# Ion‐Pairing‐Modulated Diradical Properties in Partially Conjugated Negatively Charged π‐Electronic Systems

**DOI:** 10.1002/chem.202502698

**Published:** 2025-09-30

**Authors:** Hiroto Kobayashi, Takashi Kubo, Shinya Sugiura, Yohei Haketa, Hiromitsu Maeda

**Affiliations:** ^1^ Department of Applied Chemistry, College of Life Sciences Ritsumeikan University Kusatsu 525–8577 Japan; ^2^ Department of Chemistry, Graduate School of Science The University of Osaka Toyonaka 560–0043 Japan

**Keywords:** π‐electronic anions, cross‐conjugated systems, diradical properties, ion pairs, quinones

## Abstract

A quinonoidal dipyrrolyldiketone catecholate‐boron complex, with two pyrrole‐quinonemethide moieties bridged by a six‐membered cross‐conjugated unit, was synthesized to modulate the diradical character of the dianion formed upon deprotonation. The dianionic species exhibited near‐infrared absorption and electron spin resonance (ESR) signals, confirming the diradical properties. Variable‐temperature (VT) ESR spectra suggest the thermal excitation from the ground‐state singlet diradical to the triplet diradical, providing singlet–triplet energy gaps modulated by coexisting cations and bridging boron moieties.

## Graphical abstract

A quinonoidal dipyrrolyldiketone boron complex with an orthogonally arranged catechol unit in the dianionic form exhibited diradical properties, as observed from the singlet–triplet energy gaps (Δ*E*_ST_) that were modulated by coexisting countercations. Δ*E*_ST_ values were controlled by the bridging boron moieties in the dianion diradicals.

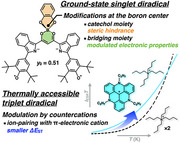

## 1 Introduction

Two fundamental characteristics of electrons, charge and spin, are crucial for the development of functional π‐electronic materials.^[^ ^1^, ^2^ ^]^ Charged π‐electronic systems have garnered attention as building blocks for materials exhibiting unique properties derived from electron‐deficient and electron‐rich states.^[^ ^3^, ^4^ ^]^ Interactions between π‐electronic cations and anions via electrostatic and dispersion forces modulate electronic states and ion‐pairing assemblies in solution and solid states. Therefore, the molecular design of π‐electronic ion pairs is essential for controlling their interactions and fabricating functional ion‐pairing assemblies and materials.^[^ ^5^ ^]^ Appropriate control of geometries and electronic states of charged π‐electronic systems results in modulation of *^i^* π–*^i^* π interactions and electron‐transfer behaviors. Electron spin is essential for designing π‐electronic materials with controllable magnetic functionalities. In particular, diradical systems with two unpaired electron spins serve as versatile platforms for tuning magnetic properties through molecular design.^[^ ^6^, ^7^, ^8^, ^9^, ^10^, ^11^, ^12^, ^13^, ^14^, ^15^ ^]^

**Figure 1:**
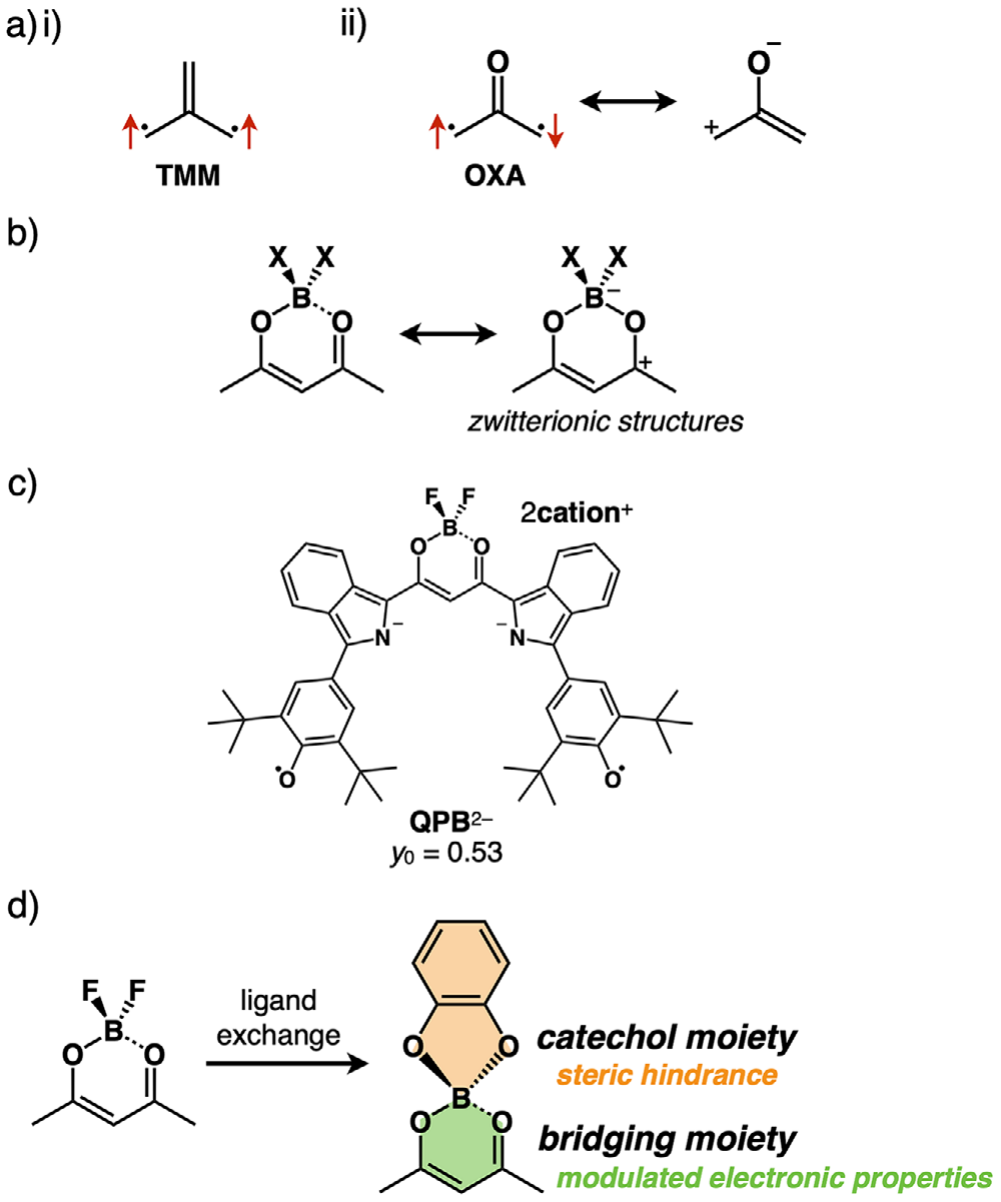
a) Bridging units for tuning diradical properties: i) trimethylenemethane (TMM) and ii) oxyallyl (OXA) diradicals, b) resonance structures of boron‐bridged 1,3‐diketonate units, c) **QPB** ^2−^ showing countercation‐dependent diradical properties, and d) a conceptual diagram of boron‐modifications.

Among various design elements in diradicals, the electronic states of the bridging moieties play a crucial role in modulating their magnetic properties. Cross‐conjugated bridging moieties effectively induce diradical properties by allowing moderate interactions between two electron spins. Representative non‐Kekulé‐type diradical species include trimethylenemethane (TMM) and oxyallyl (OXA), which exhibit open‐shell triplet and singlet states, respectively, depending on the absence and presence of a polarized unit (Figure 1a).^[^ ^14^, ^15^, ^16^, ^17^, ^18^, ^19^, ^20^, ^21^, ^22^ ^]^ Polarized cross‐conjugated systems include boron‐bridged 1,3‐diketonate units,^[^ ^23^ ^]^ which show conjugated states derived from zwitterionic resonance forms comprising a propenyl cation and a borate anion (Figure 1b). Boron complexation enhances contributions from conjugated states and modulates the electronic states and structures of the bridging six‐membered ring units, depending on the moieties attached to boron.^[^ ^24^, ^25^ ^]^ As a charged diradical species, the dianion **QPB** ^2−^ of **q**uinonoidal di**p**yrrolyldiketone **b**oron complex (**QPB**), with two pyrrole‐quinonemethide moieties bridged by a 1,3‐propanedione BF_2_ complex unit, exhibited ground‐state singlet diradical properties modulated by coexisting countercations (Figure 1c).^[^ ^26^ ^]^ The pyrrole‐β‐benzo‐fused structure^[^ ^27^ ^]^ in **QPB** stabilizes the system through enhanced π‐conjugation extending through the pyrrole β‐positions. **QPB** is the oxidized form of the corresponding di**p**yrrolyldiketone **b**oron (BF_2_) complex (**PB**) and has been used in anion complexation and ion‐pairing assemblies.^[^ ^28^, ^29^, ^30^ ^]^ The BF_2_ unit in **PB**s can be replaced with various diols, such as catechols, naphthalenediols, and BINOLs, which are orthogonally introduced into the dipyrrolyldiketone units.^[^ ^31^, ^32^ ^]^ Thus, boron modification in **QPB** ^2−^ by the introduction of diol units modulates the electronic and magnetic properties. In this study, a catechol‐containing derivative of **QPB** ^2−^ with countercation‐dependent diradical properties was synthesized (Figure 1d).

## 2 Results and Discussion

**Figure 2:**
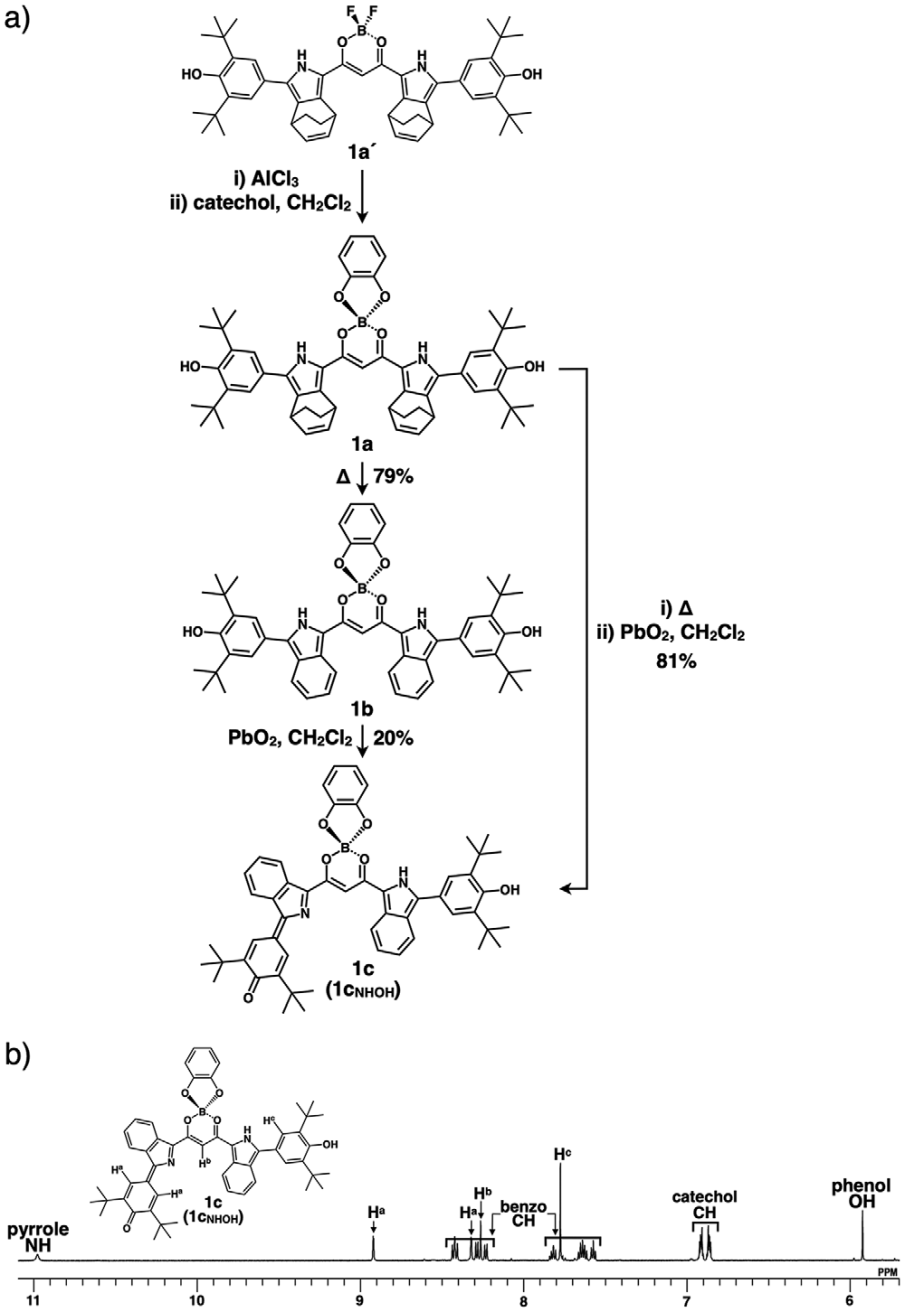
a) Synthesis of dipyrrolyldiketone catecholate–boron complexes **1a**–**c** and b) ^1^H NMR spectrum of **1c** (**1c**_NHOH_) in CD_2_Cl_2_ (1.0 mM) at 20 °C.

The catecholate–boron complex as a **QPB** derivative was investigated as a quinonoidal π‐electronic system capable of forming a negatively charged diradical. Although various synthetic pathways for the catechol complex **1a** were examined, the catechol unit was eliminated during the coupling reaction to introduce 3,5‐di‐*tert*‐butyl‐4‐hydroxyphenyl moieties.^[^ ^33^ ^]^ However, ligand exchange of 3,5‐di‐*tert*‐butyl‐4‐hydroxyphenyl‐substituted bicyclo **PB** ^[^ ^26^ ^]^ **1a´** with catechol afforded **1a** in 38% yield.^[^ ^34^, ^35^, ^36^ ^]^ Subsequently, benzo‐fused **1b** was obtained in 79% yield by eliminating ethene units of **1a** under vacuum at 160 °C. Quinonoidal **1c** was obtained in 20% yield by oxidizing **1b** with excess PbO_2_^[^ ^37^ ^]^ for 5 min in CH_2_Cl_2_ (Figure 2a). Longer reaction times resulted in lower yields of **1c**, likely because of further oxidation (dehydrogenation). Notably, **1c** was obtained from **1a** in 81% yield without isolation of **1b**.

The proton positions of the pyrrole N and terminal O in **1c** are critical for diradical properties, as observed in **QPB**,^[^ ^26^ ^]^ as various tautomeric forms and conformations are possible, depending on pyrrole inversion for the latter. For example, **1c**_NHOH_ is the tautomer possessing one NH and one OH groups on the same side, whereas **1c**_NHNH_ bears two NH groups (Figure S21). According to the theoretical study (CAM‐(U)B3LYP/6–31+G(d,p)),^[^ ^38^ ^]^ the closed‐shell singlet‐state **1c**_NHOH_ with the most stable conformation is more stable than the open‐shell singlet‐state **1c**_NHNH_ by 4.71 kcal/mol (Figures S22, S23). The tautomeric form and conformation of **1c** were confirmed using ^1^H NMR spectroscopy. The ^1^H NMR of **1c** in CD_2_Cl_2_ exhibited signals corresponding to less symmetrical conformation with a single pyrrole inversion. The pyrrole NH and phenol OH signals were observed at 10.84 and 5.81 ppm, respectively (Figures 2b and S52, S53), suggesting the formation of the closed‐shell singlet‐state **1c**_NHOH_. In addition, the bridging CH signal of **1c** observed at 7.66 ppm in CD_2_Cl_2_ was shifted downfield compared to that of **1b** (0.52 ppm), suggesting hydrogen bonding with the inverted pyrrole imine‐N (Figure S52). The corresponding CH signal of **QPB** appeared at 8.03 ppm, suggesting electronic modulation of the bridging moiety introduced in **1c**. The nucleus‐independent chemical shift (NICS)^[^ ^39^ ^]^ of **1c** at the center of the boron‐containing six‐membered ring (2.11 ppm) was larger than that of **QPB** (1.63 ppm), suggesting that the introduced catechol moiety altered the electronic states of the bridging units via the inductive effect of the oxygen atoms (Figure S29).

The electronic properties of **1c**, in the form of **1c**_NHOH_, along with **1a**,**b** as references, were examined by UV/vis absorption spectra (Figure S4). **1c** in CH_2_Cl_2_ displayed a green color with absorption maxima (λ_max_) at 431 and 615 nm, extending to 900 nm. In contrast, **1b** in CH_2_Cl_2_ exhibited blue color with λ_max_ at 606 nm, which was red‐shifted compared to the λ_max_ of **1a** (527 nm) showing red color. The UV/vis absorption spectra of **1a**–**c** were correlated with the theoretical spectrum via time‐dependent density functional theory (TD‐DFT) for the polarizable continuum model (PCM) (CH_2_Cl_2_).^[^ ^38^ ^]^ The observed λ_max_ values at 431 and 615 nm for **1c** were attributed to the theoretical absorptions at 410 and 552 nm, respectively, originating primarily from the HOMO–2‐to‐LUMO and HOMO–1‐to‐LUMO transitions, respectively (Figure S46). According to the theoretical study, HOMO and LUMO of **1c**_NHOH_ were localized mainly at the catechol moiety and inverted quinonemethide‐benzopyrrole unit, respectively (Figure S35). Absorption in the near‐infrared (NIR) region was caused by the intramolecular HOMO‐to‐LUMO charge transfer. The observed λ_max_ values were also correlated well with the respective HOMO–LUMO energy gaps of 4.69 and 3.82 eV for **1b** and **1c**_NHOH_, respectively, as calculated at CAM‐B3LYP/6–31+G(d,p) (Figures S34, S35). The smaller gap for **1c**_NHOH_ can be ascribed to the more decreased LUMO level than the HOMO. In contrast to **1b**, with the fluorescence emission maximum (λ_em_) (quantum yield, Φ_FL_) of 606 nm (0.35), in CH_2_Cl_2_, **1c** exhibited no fluorescence emission (Figure S4).

**Figure 3:**
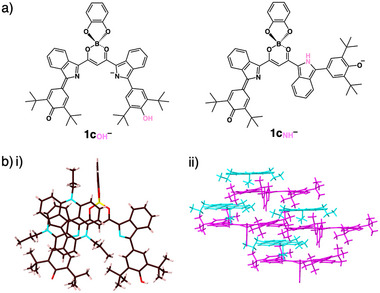
a) Most stable conformations of **1c**_OH_^–^ and **1c**_NH_^–^ as tautomers of **1c** ^–^ and b) single‐crystal X‐ray structure of TATA^+^‐**1c**_OH_^–^ as i) a top view of the ion pair (one of the independent structures) and ii) a packing structure (magenta: **1c**_OH_^–^: cyan: TATA^+^). In b) i), atom color code: brown, pink, yellow, blue, and red refer to carbon, hydrogen, boron, nitrogen, and oxygen, respectively.

The electronic state of **1c** can be modulated by the deprotonation of pyrrole NH and phenol OH. Deprotonated **1c** ^−^ adopts two tautomers that are deprotonated at NH or OH, and the corresponding structures are labelled **1c**_OH_^−^ and **1c**_NH_^−^, respectively. The most stable conformations of **1c**_OH_^−^ and **1c**_NH_^−^ are a doubly pyrrole‐inverted structure as a closed‐shell state and a singly pyrrole‐inverted structure as an open‐shell singlet state, respectively, with **1c**_OH_^−^ favored by 2.85 kcal/mol (Figures 3a and S26).^[^ ^38^ ^]^ Therefore, **1c** ^−^ in the following discussion is regarded as **1c**_OH_^−^. Formation of the monoanionic species **1c** ^−^ was confirmed upon deprotonation by the changes of the UV/vis absorption spectra (Figure S54a). In CH_2_Cl_2_, the λ_max_ of **1c** at 615 nm decreased upon the addition of tetrabutylammonium hydroxide (TBAOH) (1.6 equiv), while a broad absorption band of **1c** ^−^ at 786 nm appeared simultaneously. ^1^H NMR (CDCl_3_) showed the OH signal at 5.37 ppm, and ^1^H–^1^H NOESY confirmed correlation between OH and *tert*‐butyl protons, further verifying the deprotonation species (Figure S57).

The monoanion species serves as a crucial intermediate in the stepwise formation of dianion ion pairs. Ion pair of **1c** ^−^ with a TBA cation was obtained by adding TBAOH to a MeOH solution of **1c**. Similar to TBA⁺‐**QPB** ^−^, TBA⁺‐**1c** ^−^ exhibited no significant stacking interactions in solution (Figure S58). In contrast, ion‐pair metathesis between a Na^+^ ion pair of **1c** ^−^ (Na^+^·**1c** ^−^), prepared by treating **1c** with NaOH, and desired cation Cl^−^ salts in CH_2_Cl_2_ provided the corresponding ion pairs. Actually, Na^+^·**1c** ^−^ was converted to TATA^+^‐**1c** ^−^, the ion pair with 4,8,12‐tripropyl‐4,8,12‐triazatriangulenium cation (TATA^+^),^[^ ^40^, ^41^ ^]^ as a π‐electronic cation. In the ^1^H NMR of TATA^+^‐**1c** ^−^ in CDCl_3_ (1.0 mM), the signals of both **1c** ^−^ and TATA^+^ were shifted upfield from those of TBA^+^‐**1c** ^−^ and TATA^+^‐Cl^−^, owing to the shielding effect of stacking (Figure S58). The quinonemethide‐CH signals of **1c** ^−^ in TATA^+^‐**1c** ^−^ appeared at 8.35 and 7.95 ppm, whereas the corresponding signals in TBA^+^‐**1c** ^−^ appeared at 8.60 and 8.18 ppm. The bridging CH signals of TATA^+^‐**1c** ^−^ and TATA^+^‐**QPB** ^−^ were 7.77 and 7.66 ppm, respectively, suggesting that stacking interactions influence the electronic environments of the ion‐pairing structures.

The exact structure and ion‐pairing assembly mode of TATA^+^‐**1c** ^−^ were elucidated using single‐crystal X‐ray analysis (Figure 3b).^[^ ^42^, ^43^, ^44^ ^]^ The C–O bond lengths for the quinonemethide and phenol moieties were 1.25 and 1.37 Å, respectively, consistent with the tautomeric form **1c**_OH_^−^ in solid and solution states (Figure S12). The planarity and distortion of the anionic structure of the crystal were related to the ion‐pairing assembly mode. The dihedral angle between the pyrrole and quinonemethide units in **1c** ^−^ was estimated to be 11.4°, whereas that between the pyrrole and phenol units was 22.2° (Figure S12). In contrast, the angles in TATA⁺‐**QPB** ^−^ were 19.4° and 23.6°, indicating a more planar geometry for **1c** ^−^. Furthermore, **1c** ^−^ and TATA^+^ showed *^i^* π–*^i^* π interactions with stacking distances of 3.45 and 3.48 Å, shorter than those observed in TATA^+^‐**QPB** ^−^ (3.53 and 3.66 Å)^[^ ^26^ ^]^ (Figure 3b and S11). The packing arrangements in these ion pairs included alternately stacked anions and cations, exhibiting the contribution from charge‐by‐charge assemblies facilitated by negative‐charge delocalization in the π‐planes. In TATA^+^‐**1c** ^−^, the cation was slightly offset from the center of the boron‐containing six‐membered ring of **1c** ^−^ due to steric effects induced by the catechol moiety, resulting in the formation of π‐stacked ion pairs (*π‐sips*).^[^ ^5^ ^]^ Hirshfeld surface analysis supported the close stacking of **1c** ^−^ and TATA^+^ (Figure S16).^[^ ^45^ ^]^ In addition, CH–π interactions between the alkyl chains of TATA⁺ and the catechol unit of **1c** ^−^ were also observed, as confirmed by NCI analysis (Figure S18).^[^ ^46^, ^47^, ^48^, ^49^ ^]^

**Figure 4:**
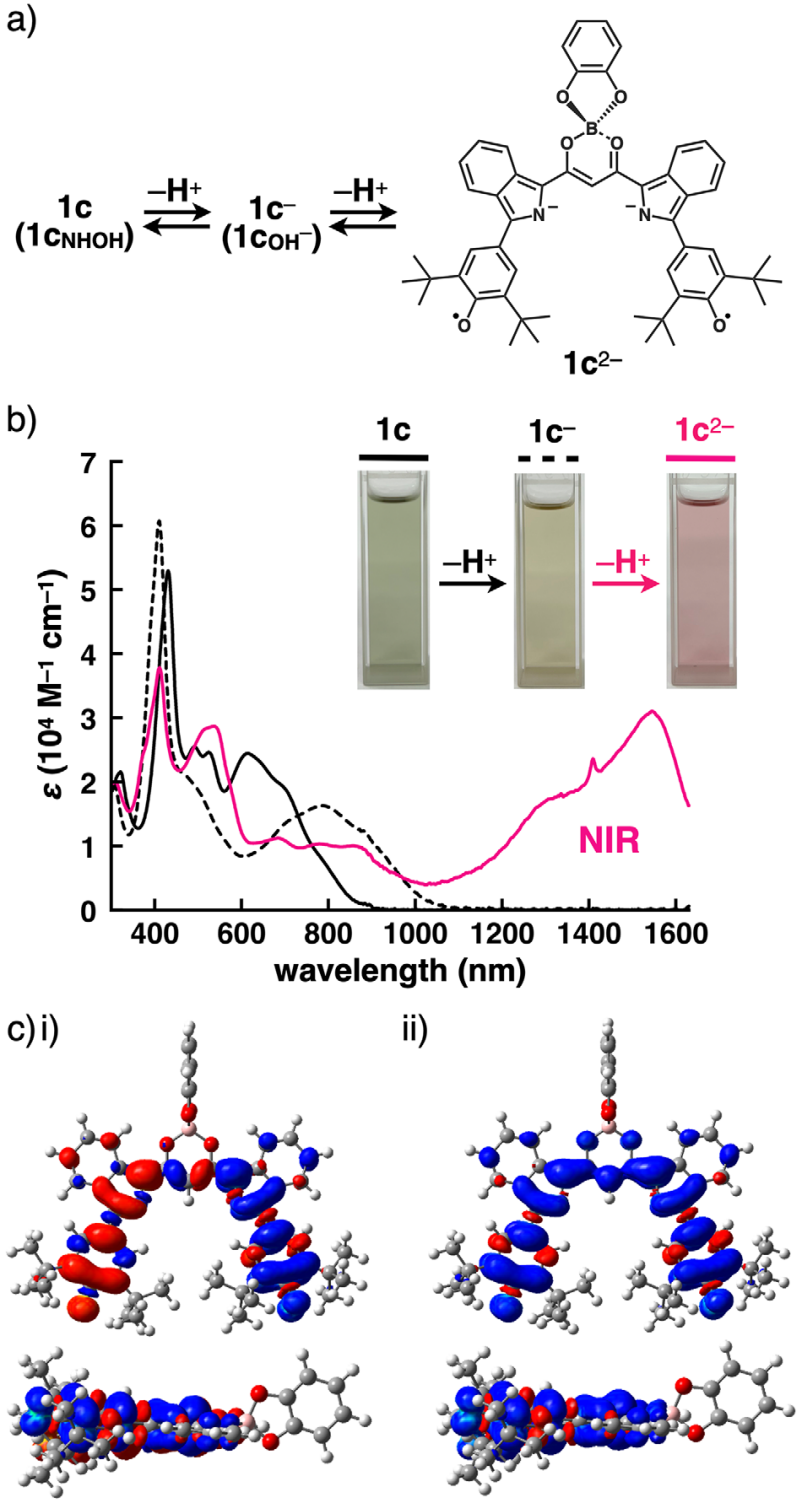
a) Deprotonation of **1c** to yield **1c** ^2−^, b) UV/vis absorption spectral change of **1c** in CH_2_Cl_2_ (0.01 mM) upon the addition of TBAOH (30 equiv) along with the corresponding photographs under visible light, and c) theoretically estimated spin densities of **1c** ^2−^ in i) singlet and ii) triplet states calculated at CAM‐UB3LYP/6–31+G(d,p).

Further deprotonation behavior yielded the dianion **1c** ^2−^ (Figure 4a), which has a diradical resonance structure (Figure S56), as revealed by theoretical and spectroscopic analyses. Theoretical calculations indicated that the most stable conformation corresponds to a doubly pyrrole‐inverted structure, similar to **1c**_OH_^−^, with the singlet state being more stable than the triplet state by 1.52 kcal/mol (Figure S27). This conformation was also associated with diradical character, supported by a theoretically estimated *y*_0_ value of 0.51 calculated at CAM‐UB3LYP/6–31+G(d,p).^[^ ^38^ ^]^ In CH_2_Cl_2_ (0.01 mM), **1c** ^2−^ exhibited a broad absorption band at ∼1500 nm upon the addition of excess TBAOH (12.8 equiv) (Figures 4b and S54b). At 20 °C in CD_2_Cl_2_, ^1^H NMR signals disappeared except for those of the catechol moiety upon the addition of TBAOH (3 equiv), whereas at −50 °C, the sharp ^1^H NMR signals assigned as the singlet diradical of **1c** ^2−^ were observed, suggesting thermal excitation from the ground‐state singlet diradical to the triplet diradical (Figure S56). Furthermore, the singlet and triplet spin densities were delocalized on the diketone moiety without spin distribution at the catechol unit because of its orthogonal arrangement (Figures 4c and S49).

**Figure 5:**
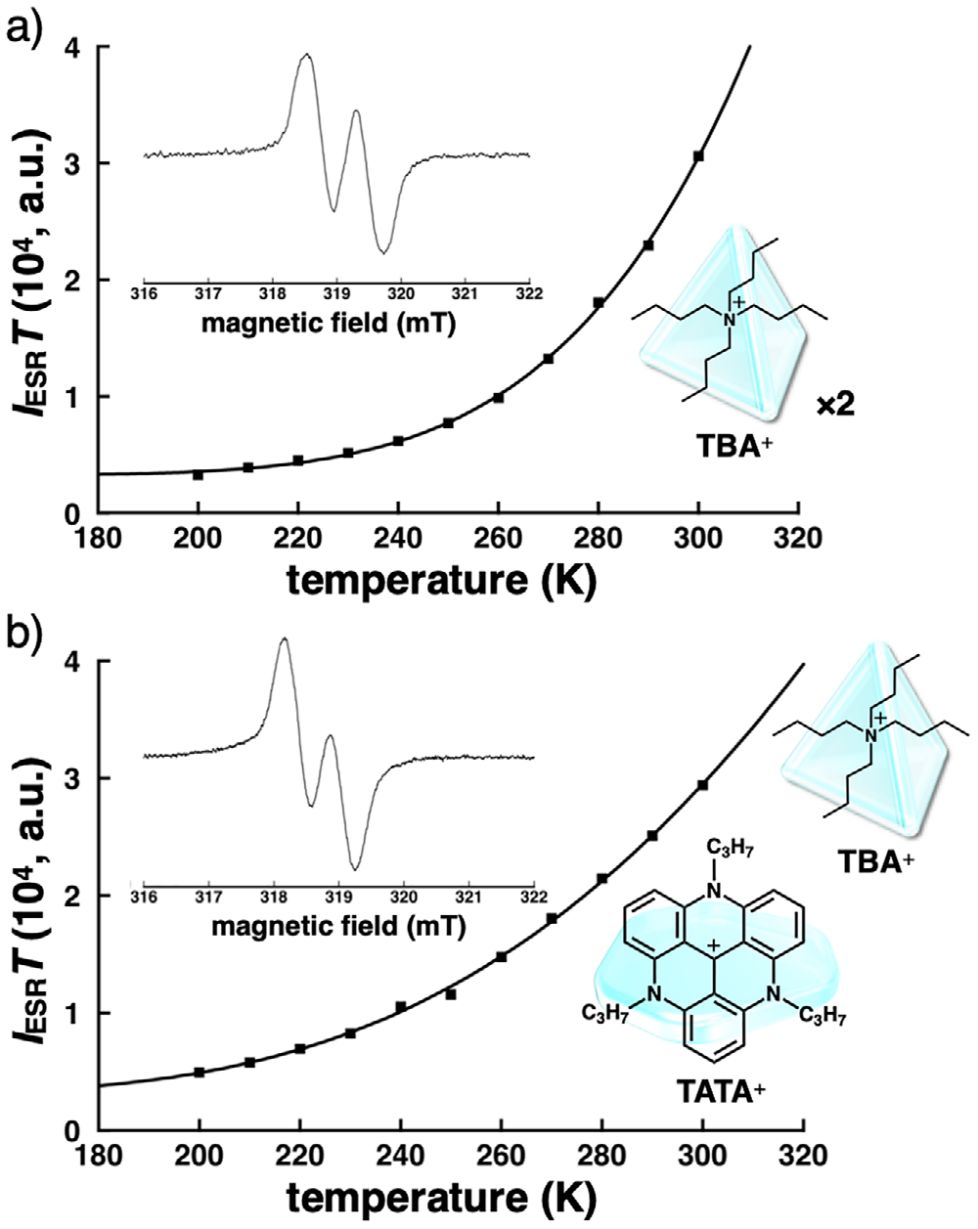
ESR spectra (insets, 250 and 300 K, respectively) and corresponding temperature‐dependent *I*_ESR_ *T* plots of a) TBA^+^_2_–**1c** ^2–^ and b) TATA^+^‐TBA^+^‐**1c** ^2–^ in toluene.

The diradical properties of **1c** ^2−^ was further examined by electron spin resonance (ESR) spectra in toluene using a quartz capillary tube.^[^ ^50^ ^]^ ESR spectra of TBA^+^_2_‐**1c** ^2−^ and TATA^+^‐TBA^+^‐**1c** ^2−^, prepared by the addition of TBAOH (2 and 1 equiv) to **1c** and TATA^+^‐**1c** ^−^, respectively, were examined to investigate the effects of coexisting countercations on the diradical properties (Figures 5 inset and S59).^[^ ^51^, ^52^, ^53^ ^]^ These experimental results indicated that two unpaired electrons interact through the extended and partially conjugated π‐unit, stabilizing the singlet diradical as the more stable state. Thermodynamic stability of the singlet diradical was examined by temperature‐dependent ESR spectral changes in the ion pairs (Figures 5 and S60). The signal intensities *I*_ESR_ increased upon heating from −73 °C to r.t., suggesting that the more stable singlet diradicals were converted to the triplet diradicals. The *I*_ESR_ *T *− *T* plots were fitted using the Bleaney‐Bowers equation for the two‐site Heisenberg Hamiltonian *H* = −2*JS*_1_·*S*_2_, which was described as

$$I_{ESR}T=\frac{C_{1}}{3+\exp\left(-\frac{2J}{RT}\right)}+C_{2}$$

(*J*: exchange interaction constant, *S*_1,2_: operators for the spins of two unpaired electrons, *C*_1_: constant, *C*_2_: constant derived from radical impurities, *R*: gas constant).^[^ ^54^, ^55^ ^]^ The fitting curves provided the singlet–triplet energy gaps (Δ*E*_ST_) of TBA^+^_2_–**1c** ^2−^ and TATA^+^‐TBA^+^‐**1c** ^2−^ as −5.4 and −3.2 kcal/mol, respectively, suggesting the effects of countercations (Figures 5 and S60). Lower‐temperature VT measurements in toluene enabled reliable estimation of Δ*E*_ST_ values even for TBA^+^_2_–**1c** ^2−^ in contrast to the TBA^+^ ion pair of **QPB** ^2−^, for which Δ*E*_ST_ was inaccessible owing to thermal instability.^[^ ^56^ ^]^ The |Δ*E*_ST_| value of TATA^+^‐TBA^+^‐**1c** ^2−^ was smaller than that of TATA^+^‐TBA^+^‐**QPB** ^2−^ (−6.4 kcal/mol), suggesting that modulated electronic states and geometries around the boron center substantially affect singlet–triplet energy gaps. The smaller |Δ*E*_ST_| value of TATA^+^‐TBA^+^‐**1c** ^2−^ compared to those of TBA^+^_2_‐**1c** ^2−^ and TATA^+^‐TBA^+^‐**QPB** ^2−^ may arise from the less effective π‐conjugation and the resulting spin localization.^[^ ^57^ ^]^

## 3 Conclusion

This study demonstrated that boron modification with an orthogonally oriented catechol unit in quinonoidal dipyrrolyldiketone boron complexes resulted in diradical properties in the dianion state, featuring a distinct electronic structure compared to that of the BF_2_ analog (**QPB** ^2−^). The dianion exhibits near‐infrared absorption and thermally activated ESR signals consistent with a ground‐state singlet diradical. Modifications of the boron moiety led to a narrower singlet–triplet energy gap Δ*E*_ST_, suggesting the effects of modulated electronic states. The increased steric structure, along with the use of different countercations, offers a valuable strategy for tuning spin states through ion pairing. Further investigations into structurally and electronically diverse π‐electronic cations^[^ ^58^ ^]^ are in progress to elucidate their effects on diradical properties.

## 4 Experimental Section

### 4.1 Crystallographic Data

Deposition numbers 2482008–2482011 contain the supplementary crystallographic data for this paper. These data are provided free of charge by the joint Cambridge Crystallographic Data Centre and Fachinformationszentrum Karlsruhe Access Structures service.

## Conflict of Interest

The authors declare no conflict of interest.

## Supporting information



Supporting Information

Supporting Information

## Data Availability

The data that support the findings of this study are available in the supplementary material of this article.
