# Supplementary material for: Ion‐Pairing‐Modulated Diradical Properties in Partially Conjugated Negatively Charged π‐Electronic Systems
Source: Chemistry. 2025 Sep 30;31(65):e02698. doi: 10.1002/chem.202502698 (PMC12642387; doi:10.1002/chem.202502698)
Supplement: Supplementary file 1 — Supporting Information [file CHEM-31-e02698-s002.pdf]

Supporting Information

Ion-Pairing-Modulated Diradical Properties in Partially Conjugated Negatively Charged π-Electronic Systems

Hiroto Kobayashi, Takashi Kubo, Shinya Sugiura, Yohei Haketa, and Hiromitsu Maeda*

Department of Applied Chemistry, College of Life Sciences, Ritsumeikan University, Kusatsu 525–8577, Japan, Fax: +81 77 561 3729; Tel: +81 77 561 5969; E-mail: maedahir@ph.ritsumei.ac.jp and Department of Chemistry, Graduate School of Science, The University of Osaka, Toyonaka 560–0043, Japan

Table of Contents

1. Synthetic procedures and spectroscopic data S2
Figure S1–3 ^{1}H and ^{13}C NMR spectra. S4
Figure S4 UV/vis absorption and fluorescence spectra. S7
2. X-ray crystallographic data S8
Figure S5–8 Ortep drawings. S9
Figure S9–14 Packing diagrams. S11
Figure S15–17 Hirshfeld surfaces. S14
Figure S18 NCI plot. S16
3. Theoretical study S17
Figure S19–28 Optimized structures. S17
Figure S29 NICS values. S24
Figure S30–32 Electrostatic potential mapping. S25
Figure S33–43 Molecular orbitals. S26
Figure S44–48 TD-DFT calculations. S34
Figure S49 Spin density mapping. S38
Figure S50 Molecular atom labels. S39
Cartesian coordination of optimized structures S40
4. Solution-state properties S104
Figure S51 Solvent-dependent UV/vis absorption spectra. S104
Figure S52 Solvent-dependent ^{1}H NMR spectra. S104
Figure S53 VT ^{1}H NMR spectra. S105
5. Deprotonation behaviors and diradical properties S106
Figure S54,55 UV/vis absorption spectral changes upon deprotonation. S106
Figure S56–58 ^{1}H NMR spectra. S107
Figure S59,60 ESR spectra. S110

1. Synthetic procedures and spectroscopic data

General Procedures. Starting materials were purchased from FUJIFILM Wako Pure Chemical Corp., Nacalai Tesque Inc., and Sigma-Aldrich Co. and were used without further purification unless otherwise stated. NMR spectra used in the characterization of products were recorded on a JEOL ECA-600 600 MHz spectrometer. All NMR spectra were referenced to solvent. UV-visible absorption spectra were recorded on a Hitachi U-3500 spectrometer. Fluorescence spectra were recorded on a Hitachi F-4500 fluorescence spectrometer. High-resolution (HR) electrospray ionization mass spectrometry (ESI-MS) was recorded on a BRUKER microTOF using ESI-TOF method. TLC analyses were carried out on aluminum sheets coated with silica gel 60 (Merck 5554). Column chromatography was performed on Wakogel C-300 and Wakosil HC-N.

Catecholate boron complex of 1,3-bis(3-(3,5-di-*tert*-butyl-4-hydroxyphenyl)-4,7-ethano-4,7-dihydroisoindol-1-yl)-1,3-propanedione, 1a. According to the literature procedures,[S1] BF_2 complex of 1,3-bis(3-(3,5-di-*tert*-butyl-4-hydroxyphenyl)-4,7-ethano-4,7-dihydroisoindol-1-yl)-1,3-propanedione **1a′** [S2] (108 mg, 0.133 mmol) was dissolved in dry CH_2Cl_2 (5.4 mL) in the presence of $AlCl_3$ (31.2 mg, 0.234 mmol) under N_2. The resulting mixture was refluxed for 5 min prior to addition of catechol (48.0 mg, 0.436 mmol). After 5 min at r.t., the crude mixture was partitioned between water and CH_2Cl_2. The organic extract was dried over anhydrous Na_2SO_4 and was evaporated. The residue was then chromatographed over silica gel column (Wakogel C-300, eluent: CH_2Cl_2) to give **1a** (41.2 mg, 46.5 μmol, 38%) as a dark green solid. R_f = 0.75 (CH_2Cl_2). ^{1}H NMR (600 MHz, $CDCl_3$, 20 °C): δ (ppm) 8.83 (s, 2H, NH), 7.33 (s, 4H, phenol-H), 6.89–6.85 (m, 2H, catechol-H), 6.84 (s, 1H, CH), 6.79–6.76 (m, 2H, catechol-H), 6.65–6.60 (m, 4H, HC=CH), 5.41 (s, 2H, OH), 4.41–4.40 (m, 2H, C*H*CH_2CH_2), 4.23–4.21 (m, 2H, C*H*CH_2CH_2), 1.82–1.58 (m, 8H, CH_2CH_2), 1.50 (s, 36H, $C(CH_3)_3$). $^{13}C\{^1H\}$ NMR (151 MHz, $CDCl_3$, 20 °C) δ (ppm) 166.04, 154.82, 151.31, 141.90, 137.10, 136.85, 134.49, 133.80, 131.30, 124.25, 122.71, 119.53, 118.53, 109.93, 92.01, 36.17, 34.84, 34.07, 30.63, 26.74, 26.65. UV/vis (CH_2Cl_2, λ_{max}[nm] (ε, 10^5 $M^{-1}cm^{-1}$)): 527 (1.01). ESI-TOF-MS (HR): 919.4624. Calcd for $C_{57}H_{64}BClN_2O_6$ ($[M + Cl]^-$): 919.4624. This compound was further characterized as an anion complex (an ion pair) by single-crystal X-ray analysis.

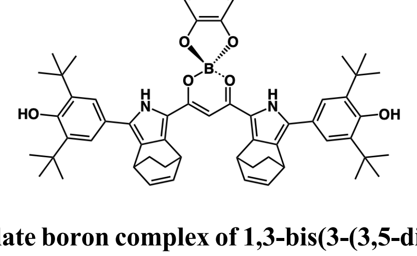

Catecholate boron complex of 1,3-bis(3-(3,5-di-*tert*-butyl-4-hydroxyphenyl)isoindol-1-yl)-1,3-propanedione, 1b. According to the literature procedure,[S2] a CH_2Cl_2 solution (5 mL) of **1a** (14.4 mg, 16.3 μmol) was evaporated and the residue was heated under vacuum at 160–170 °C for 6 h. Silica gel column chromatography (Wakosil HC-N; eluent: CH_2Cl_2) and crystallization from CH_2Cl_2/*n*-hexane afforded **1b** (10.6 mg, 12.8 μmol, 79%) as a dark purple solid. R_f = 0.73 (CH_2Cl_2). ^{1}H NMR (600 MHz, $CDCl_3$, 20 °C): δ (ppm) 10.40 (s, 2H, NH), 8.15 (d, J = 8.4 Hz, 2H, benzo-H), 8.01 (d, J = 8.4 Hz, 2H, benzo-H), 7.62 (t, J = 7.2 Hz, 2H, benzo-H), 7.52 (s, 4H, phenol-H), 7.34 (t, J = 7.2 Hz, 2H, benzo-H), 7.13 (s, 1H, CH), 6.89–6.88 (m, 2H, catechol-H), 6.79–6.78 (m, 2H, catechol-H), 5.54 (s, 2H, OH), 1.51 (s, 36H, $C(CH_3)_3$). $^{13}C\{^1H\}$ NMR could not be measured due to the less stability. UV/vis (CH_2Cl_2, λ_{max}[nm] (ε, 10^5 $M^{-1}cm^{-1}$)): 606 (1.44). Fluorescence (CH_2Cl_2, λ_{em}[nm] (λ_{ex}[nm], Φ_{FL})): 638 (606, 0.35). ESI-TOF-MS (HR): 827.4226. Calcd for $C_{53}H_{56}BN_2O_6$ ($[M - H]^-$): 827.4231.

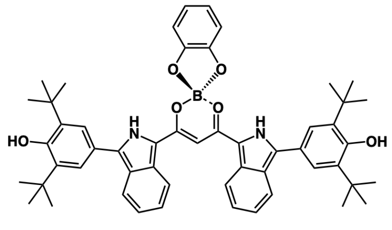

Catecholate boron complex of 1-(3-(3,5-di-*tert*-butyl-4-hydroxyphenyl)isoindol-1-yl)-3-(1-(3,5-di-*tert*-butyl-4-oxocyclohexa-2,5-dienylidene)isoindol-3-yl)propane-1,3-dione, 1c. According to the literature procedure,[S2] a round-bottomed flask placed with **1b** (5.3 mg, 6.4 μmol) in CH_2Cl_2 (1.6 mL) under N_2 was added PbO_2 (163 mg, 0.681 mmol) at r.t. The mixture was stirred at r.t. for 5 min. The reaction mixture was filtered through the celite column. The filtrates were concentrated under reduced pressure and was dried under vacuum. The residue was then chromatographed over silica gel column (Wakosil HC-N, eluent: CH_2Cl_2:*n*-hexane = 5:2) to give **1c** (1.1 mg, 1.3 μmol, 20%) as a dark green solid. Compared to the stepwise synthesis with the isolation of **1b**, the direct conversion from **1a** to **1c** provides a higher yield, following the procedure described below. A round-bottomed flask placed with **1a** (82.0 mg, 92.7 μmol) in CH_2Cl_2 (5 mL) was evaporated, and the residue was heated under vacuum at 160–170 °C for 6 h. After cooling to r.t., CH_2Cl_2 (8 mL) was added under N_2, followed by addition of PbO_2 (1.50 g, 5.50 mmol) at r.t. The mixture was stirred at r.t. for 5 min. The reaction mixture was filtered through the celite column. The filtrates were concentrated under reduced pressure and was dried under vacuum. The residue was then chromatographed over silica gel column (Wakosil HC-N, eluent: CH_2Cl_2:*n*-hexane = 5:2) to give **1c** (66.2 mg, 75.2 μmol, 81%) as a dark green solid. R_f = 0.62 (CH_2Cl_2:*n*-hexane = 3:1). ^{1}H NMR (600 MHz, $CDCl_3$, 20 °C): δ (ppm) 10.68 (s, 1H, NH), 8.76 (d, J = 2.4 Hz, 1H, quinomethide-H), 8.39 (d, J = 7.2 Hz, 1H, benzo-H), 8.26 (d, J = 9.0 Hz, 1H, benzo-H), 8.17 (d, J = 2.4 Hz, 1H,

quinomethide-H), 8.11 (s, 1H, CH), 8.09 (d, J = 9.0 Hz, 1H, benzo-H), 8.07 (d, J = 7.8 Hz, 1H, benzo-H), 7.67 (t, J = 7.8 Hz, 1H, benzo-H), 7.62 (s, 2H, phenol-H), 7.51–7.49 (m, 1H, benzo-H), 7.49–7.47 (m, 1H, benzo-H), 7.44 (t, J = 7.2 Hz, 1H, benzo-H), 6.92–6.91 (m, 2H, catechol-H), 6.84–6.82 (m, 2H, catechol-H), 5.76 (s, 1H, OH), 1.57 (s, 18H, $C(CH_3)_3$), 1.52 (s, 9H, $C(CH_3)_3$), 1.45 (s, 9H, $C(CH_3)_3$). $^{13}C\{^1H\}$ NMR (151 MHz, $CDCl_3$, 20 °C): δ (ppm) 186.62, 166.19, 162.76, 161.85, 156.97, 154.59, 152.69, 150.73, 149.78, 144.96, 138.09, 137.51, 134.71, 133.07, 130.49, 130.21, 129.58, 127.13, 126.89, 126.27, 125.47, 125.44, 124.94, 124.28, 120.86, 119.70, 119.09, 117.94, 109.39, 98.07, 36,14, 35.92, 34.45, 30.00, 29.61, 29.43 (A signal is missing probably due to the overlapping with another signal). UV/vis (CH_2Cl_2, λ_{max}[nm] (ε, 10^5 $M^{-1}cm^{-1}$)): 431 (0.53), 492 (0.25), 524 (0.25), 615 (0.24). ESI-TOF-MS (HR): 825.4074. Calcd for $C_{53}H_{54}BN_2O_6$ ($[M - H]^-$): 825.4075. This compound was further characterized as a monoanion (an ion pair) by single-crystal X-ray analysis.

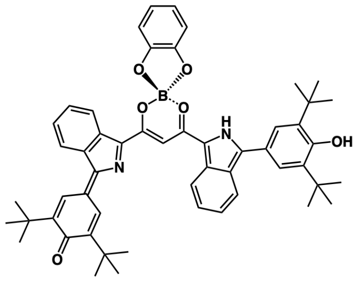

[S1] C. Tahtaoui, C. Thomas, F. Rohmer, P. Klotz, G. Duportail, Y. Mély, D. Bonnet, M. Hibert, *J. Org. Chem.* **2007**, *72*, 269–272.

[S2] S. Sugiura, T. Kubo, Y. Haketa, Y. Hori, Y. Shigeta, H. Sakai, T. Hasobe, H. Maeda, *J. Am. Chem. Soc.* **2023**, *145*, 8122–8129.

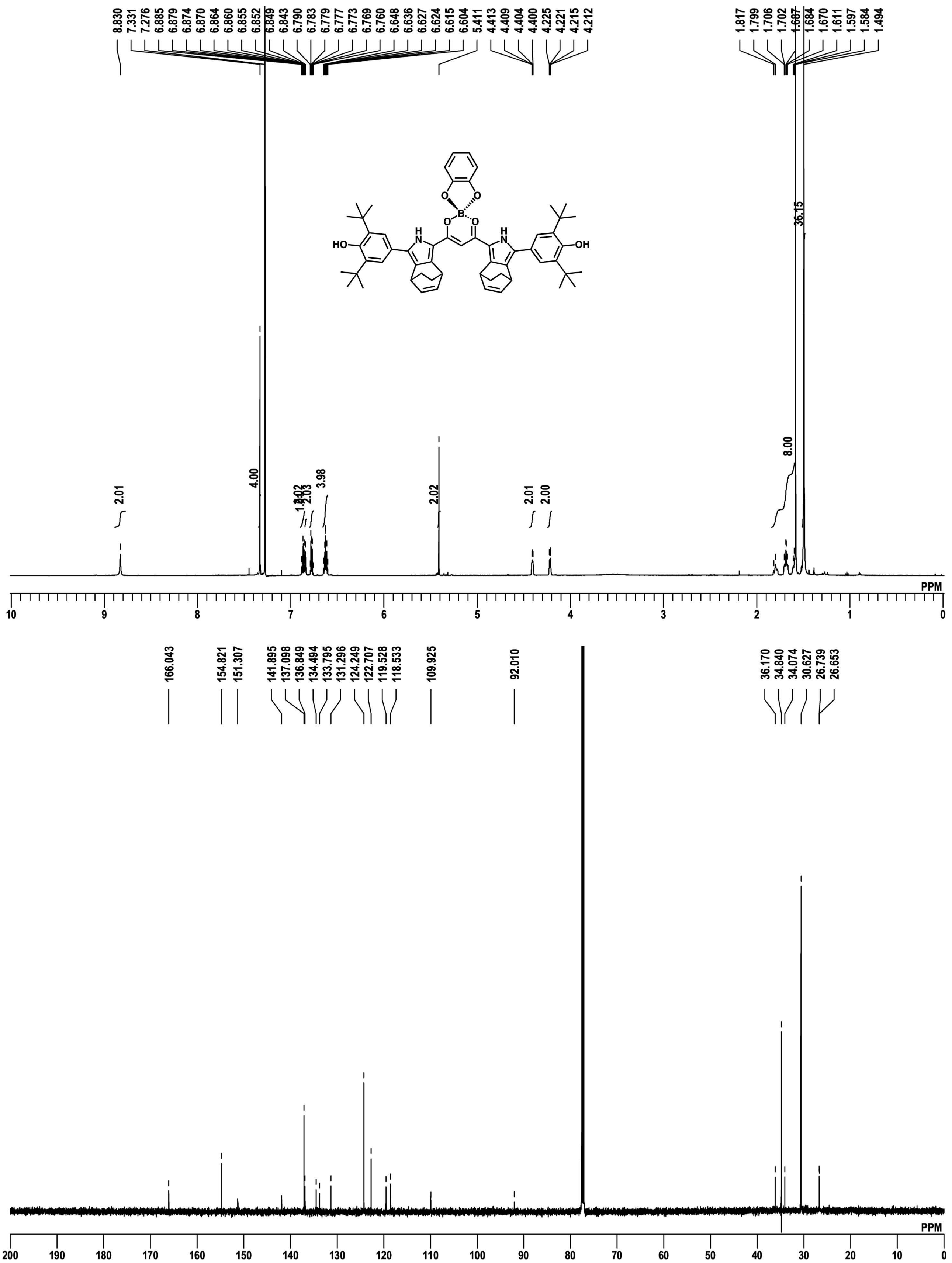

Figure S1 ^{1}H NMR (top) and $^{13}C\{^{1}H\}$ NMR (bottom) spectra of **1a** in $CDCl_3$.

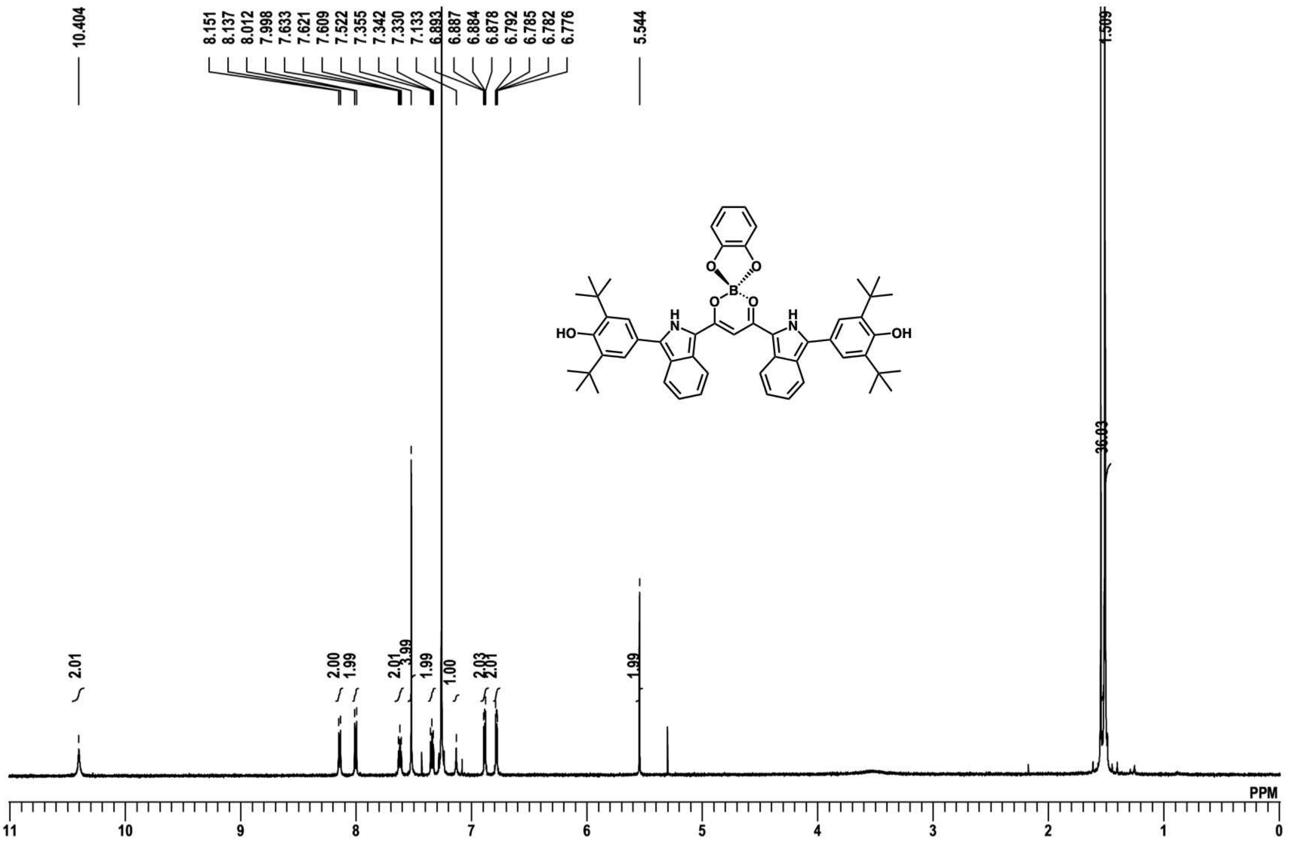

Figure S2 ^{1}H NMR spectrum of **1b** in $CDCl_3$.

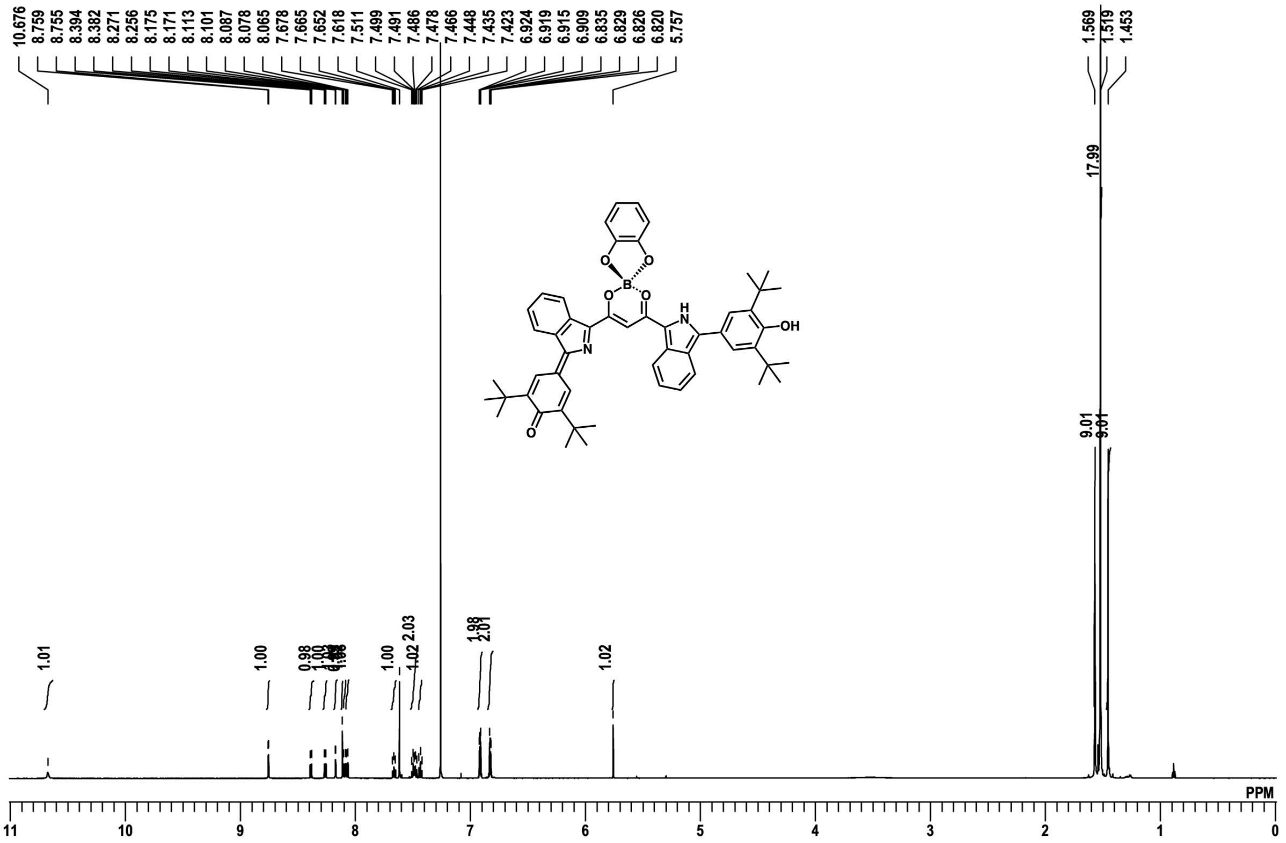

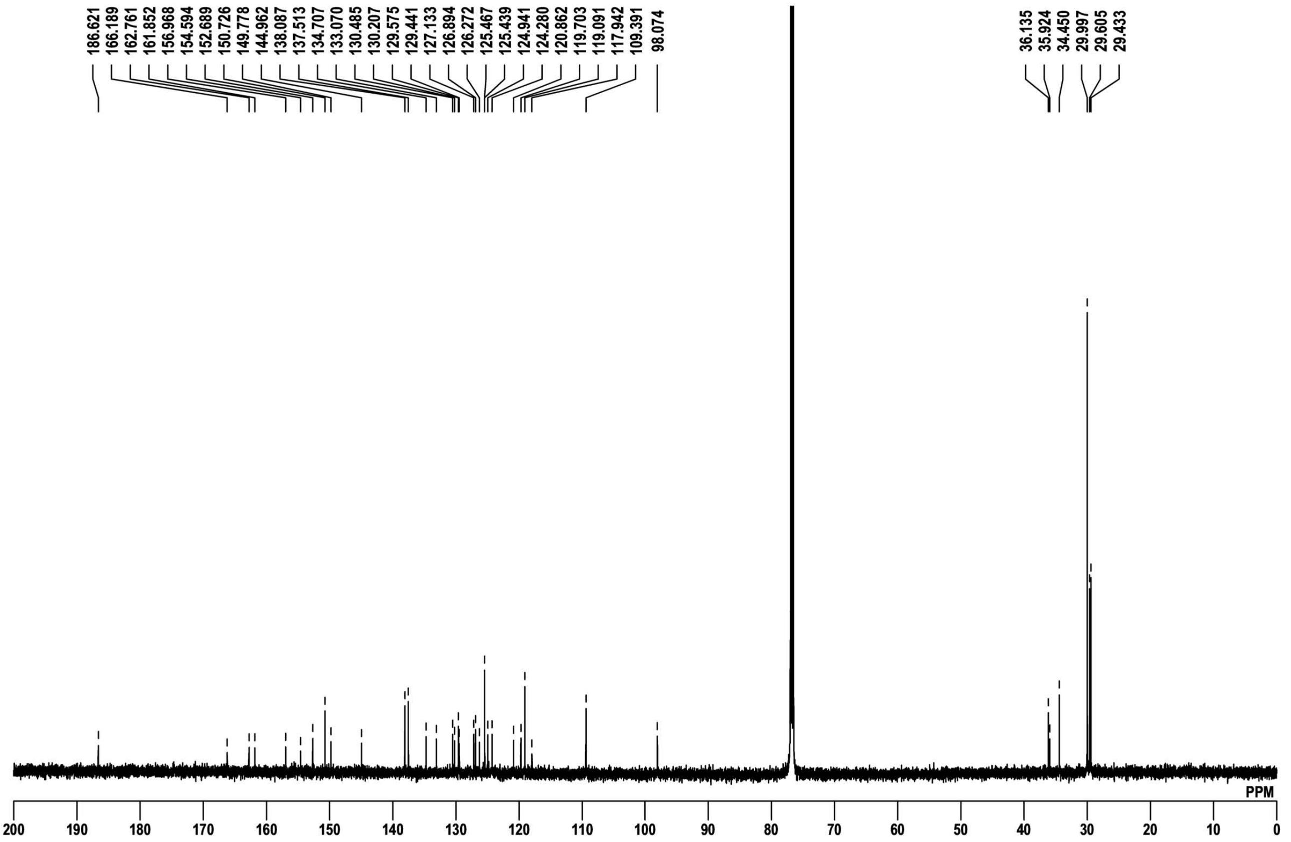

Figure S3 ^{1}H NMR (top) and $^{13}C\{^{1}H\}$ NMR (bottom) spectra of **1c** in $CDCl_3$.

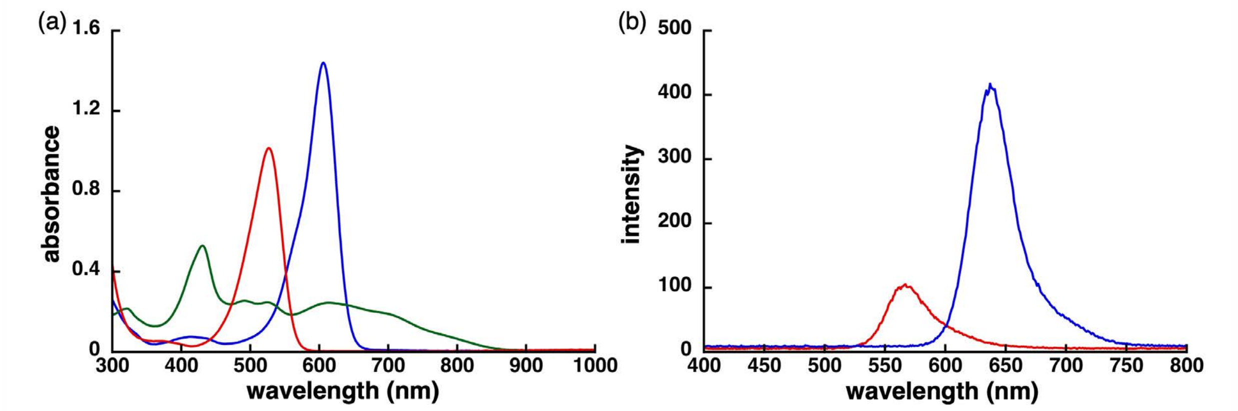

Figure S4 (a) UV/vis absorption and (b) fluorescence spectra of **1a**–**c** (red, blue, and green, respectively) in CH_2Cl_2 (1.0 $\times$ 10^{-5} M). Fluorescence spectra were measured by the excitation at their absorption maxima.

2. X-ray crystallographic data

Method for single-crystal X-ray analysis. Crystallographic data are summarized in Table S1. A single crystal of **1a** was obtained by vapor diffusion of *n*-hexane into a CH_2Cl_2 solution. The data crystal was a red prism of approximate dimensions 0.78 mm × 0.04 mm × 0.03 mm. A single crystal of $TPPAu^+$-**1a**·Cl^- was obtained by vapor diffusion of *n*-hexane into a CH_2Cl_2 solution of the 1:1 mixture of **1a** and *meso*-tetraphenylporphyrin Au^{III} complex as a Cl^- ion pair (TPPAuCl).[S3] The data crystal was a purple block of approximate dimensions 0.05 mm × 0.05 mm × 0.05 mm. A single crystal of $TATA^+$·**1c**$^-$ was obtained by vapor diffusion of *n*-hexane into a CH_2Cl_2 solution of the 1:1 mixture of **1c** and 4,8,12-tripropyl-4,8,12-triazatriangulenium chloride (TATACl).[S4] The data crystal was a brown prism of approximate dimensions 0.47 mm × 0.07 mm × 0.04 mm. A single crystal of $3C_6F_5Au^+$-**QPB**$^-$ was obtained by vapor diffusion of *n*-hexane into a CH_2Cl_2 solution of the 1:1 mixture of **QPB**$^-$, prepared by deprotonation with NaOH (1 equiv), and 5,10,15-tris(pentafluorophenyl)porphyrin Au^{III} complex as a Cl^- ion pair ($3C_6F_5AuCl$).[S5] The data crystal was a red prism of approximate dimensions 0.05 mm × 0.02 mm × 0.01 mm. The data were collected at 100 K on a DECTRIS EIGER X 1M diffractometer with Si (111) monochromated synchrotron radiation (λ = 0.81022 Å (**1a**), 0.80959 Å ($TPPAu^+$-**1a**·Cl^- and $3C_6F_5Au^+$-**QPB**$^-$), and 0.81457 Å ($TATA^+$-**1c**$^-$)) at BL40XU (SPring-8).[S6] All the structures were solved by dual-space method. The structures were refined by a full-matrix least-squares method using a SHELXL 2014[S7] (Yadokari-XG).[S8] The non-hydrogen atoms were refined anisotropically. For $TATA^+$-**1c**$^-$, the disordered solvent, presumably *n*-hexane, was removed using the SQUEEZE protocol included in PLATON.[S9] CIF files (CCDC-2482008–2482011) can be obtained free of charge from the Cambridge Crystallographic Data Centre via www.ccdc.cam.ac.uk/data_request/cif.

Table S1 Crystallographic details.

	**1a**	$TPPAu^+$-**1a**·Cl^-	$TATA^+$-**1c**$^-$	$3C_6F_5Au^+$-**QPB**$^-$
formula	$C_{57}H_{65}BN_2O_6{\cdot}C_6H_{14}$	$C_{44}H_{28}AuN_4{\cdot}C_{57}H_{65}BN_2O_6Cl{\cdot}C_6H_{14}$	$C_{28}H_{30}N_3{\cdot}C_{53}H_{54}BN_2O_6{\cdot}CH_2Cl_2{\cdot}C_6H_{14}$	$C_{39}H_9AuF_{15}N_4{\cdot}C_{47}H_{50}BF_2N_2O_4{\cdot}CH_2Cl_2$
fw	971.09	1816.21	1405.43	1844.08
crystal size, mm	0.78 × 0.04 × 0.03	0.05 × 0.05 × 0.05	0.47 × 0.07 × 0.04	0.05 × 0.02 × 0.01
crystal system	monoclinic	monoclinic	monoclinic	monoclinic
space group	*C*2/*c* (no. 15)	$P2_1/n$ (no. 14)	*Pc* (no. 7)	$P2_1/n$ (no. 14)
a, Å	31.8519(2)	18.06760(10)	12.0598(2)	25.7865(12)
b, Å	11.20780(10)	15.75940(10)	22.9541(4)	9.6585(2)
c, Å	32.2572(2)	31.0943(2)	15.1081(2)	33.2290(10)
α, °	90	90	90	90
β, °	98.5510(10)	92.3570(10)	92.6770(10)	98.463(4)
γ, °	90	90	90	90
V, Å^3	11387.48(15)	8846.13(9)	4177.69(11)	8185.9(5)
ρ_{calcd}, gcm^{-3}	1.133	1.364	1.117	1.496
Z	8	4	2	4
T, K	100(2)	100(2)	100(2)	100(2)
μ, mm^{-1}	0.094 ^{a}	2.423 ^{a}	0.182 ^{a}	2.694 ^{a}
no. of reflns	70942	107697	42978	99075
no. of unique reflns	13653	20885	13714	19075
variables	744	1111	880	1022
λ, Å	0.81022 ^{a}	0.80959 ^{a}	0.81457 ^{a}	0.80959 ^{a}
R_1 ($I > 2\sigma(I)$)	0.0882	0.0304	0.0643	0.1021
wR_2 ($I > 2\sigma(I)$)	0.2582	0.0966	0.1765	0.2301
GOF	1.025	0.834	1.024	1.068

^{a} Synchrotron radiation.

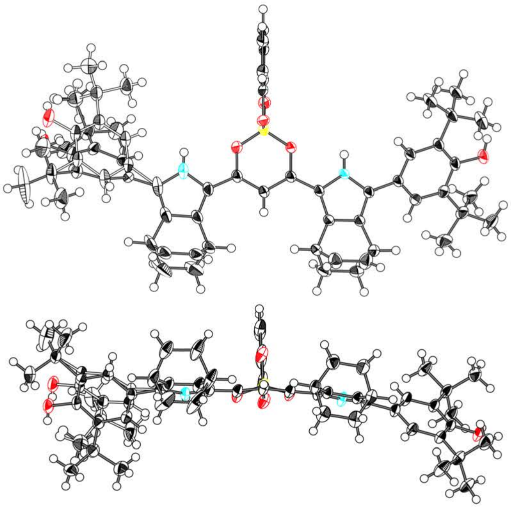

Figure S5 Ortep drawing of single-crystal X-ray structure (top and side views) of **1a** with a disordered phenyl unit in the ratio of 52:48, represented by black and white bonds for major and minor structures, respectively. Thermal ellipsoids are scaled to the 50% probability level. Solvent molecules are omitted for clarity. Atom color code: black, white, yellow, blue, and red refer to carbon, hydrogen, boron, nitrogen, and oxygen, respectively.

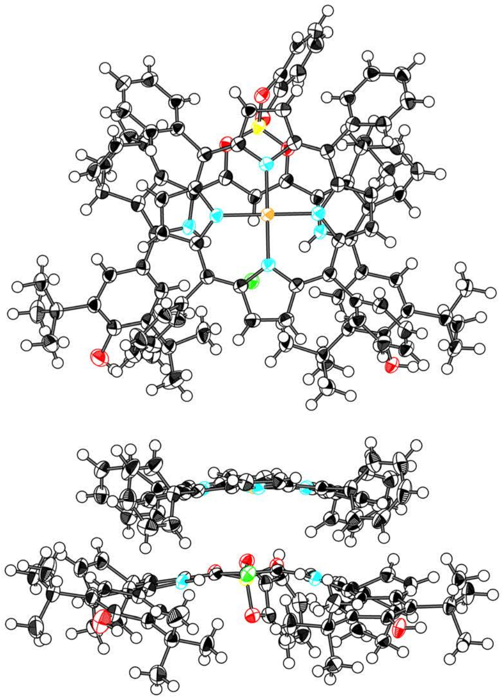

Figure S6 Ortep drawing of single-crystal X-ray structure (top and side views) of TPPAu^{+}-**1a**·Cl$^{–}$. Thermal ellipsoids are scaled to the 50% probability level. Solvent molecules are omitted for clarity. Atom color code: black, white, yellow, blue, red, yellow green, and orange refer to carbon, hydrogen, boron, nitrogen, oxygen, chlorine, and gold, respectively.

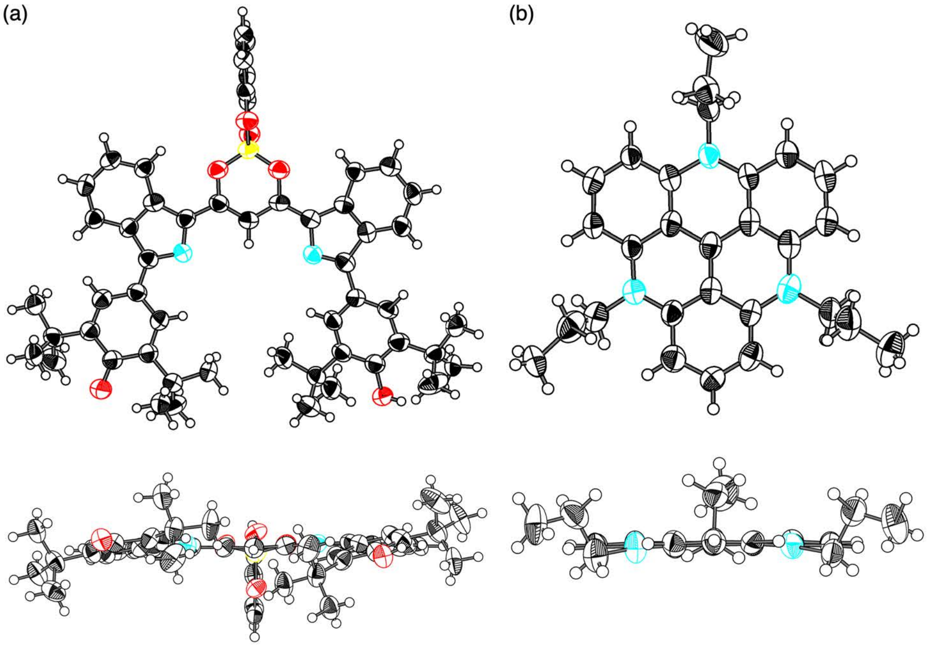

Figure S7 Ortep drawing of single-crystal X-ray structure (top and side views) of TATA$^+$-**1c**$^-$: (a) **1c**$^-$ and (b) TATA$^+$. Thermal ellipsoids are scaled to the 50% probability level. Solvent molecules are omitted for clarity. Atom color code: black, white, yellow, blue, and red refer to carbon, hydrogen, boron, nitrogen, and oxygen, respectively.

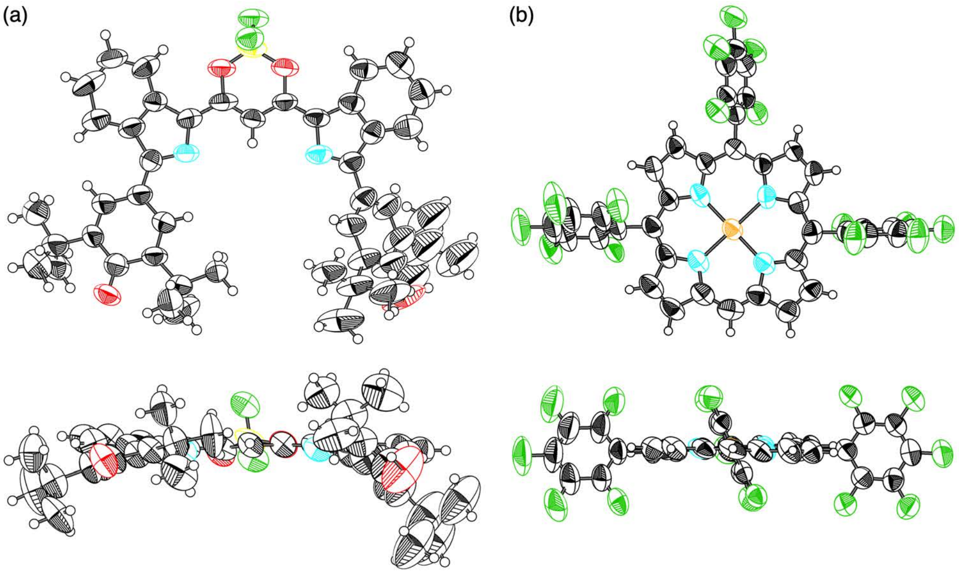

Figure S8 Ortep drawing of single-crystal X-ray structure (top and side views) of $3C_6F_5Au^+$-**QPB**$^-$: (a) **QPB**$^-$ and (b) $3C_6F_5Au^+$. Thermal ellipsoids are scaled to the 50% probability level. Solvent molecules are omitted for clarity. Atom color code: black, white, yellow, blue and red, green, and orange refer to carbon, hydrogen, boron, nitrogen, oxygen, fluorine, and gold, respectively.

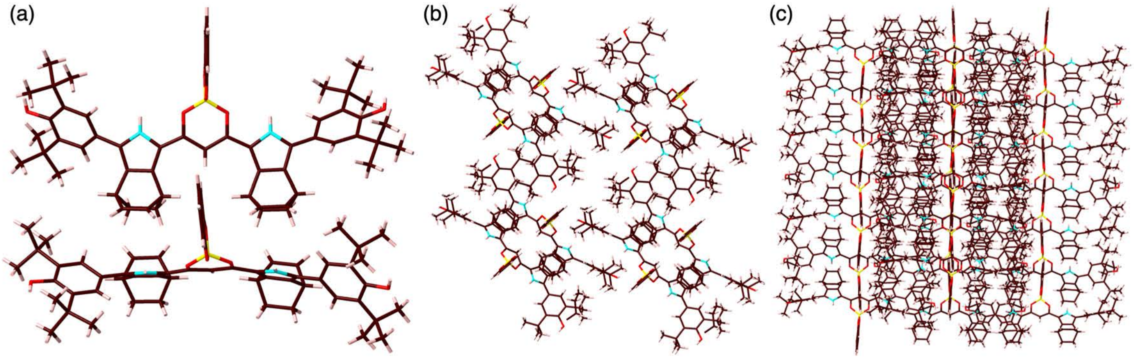

Figure S9 Single-crystal X-ray structure of **1a**: (a) dimer and (b) top and (c) side views of the packing diagram. Solvent molecule is omitted for clarity. Atom color code: brown, pink, yellow, blue, and red refer to carbon, hydrogen, boron, nitrogen, and oxygen, respectively.

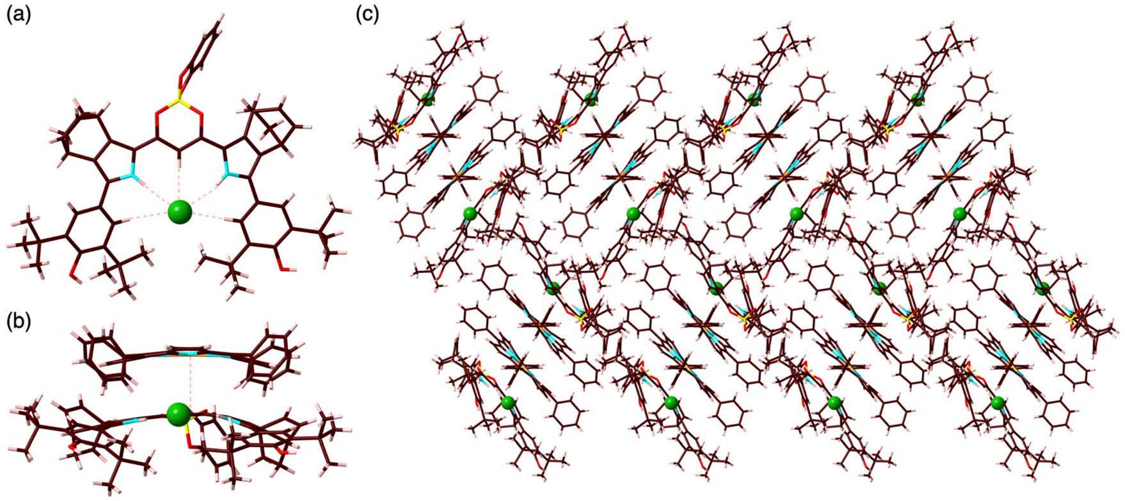

Figure S10 Single-crystal X-ray structure of $TPPAu^{+}$-**1a**$\cdot Cl^{-}$: (a) **1a**$\cdot Cl^{-}$ exhibiting interactions with pyrrole N(–H)···Cl^{-} distances of 2.40 and 2.50 Å, a bridged C(–H)···Cl^{-} distance of 2.41 Å, and phenyl C(–H)···Cl^{-} distances of 2.94 and 3.01 Å, (b) the ion pair (side view) with stacking distances of 3.60 Å estimated from the average distance of the mean plane of pyrrole-diketone unit (16 atoms) of **1a** and the mean plane of $TPPAu^{+}$ (25 atoms), and (c) packing diagram. Solvent molecules are omitted for clarity. Atom color code: brown, pink, yellow, blue, red, green (sphere), and orange refer to carbon, hydrogen, boron, nitrogen, oxygen, chlorine, and gold, respectively.

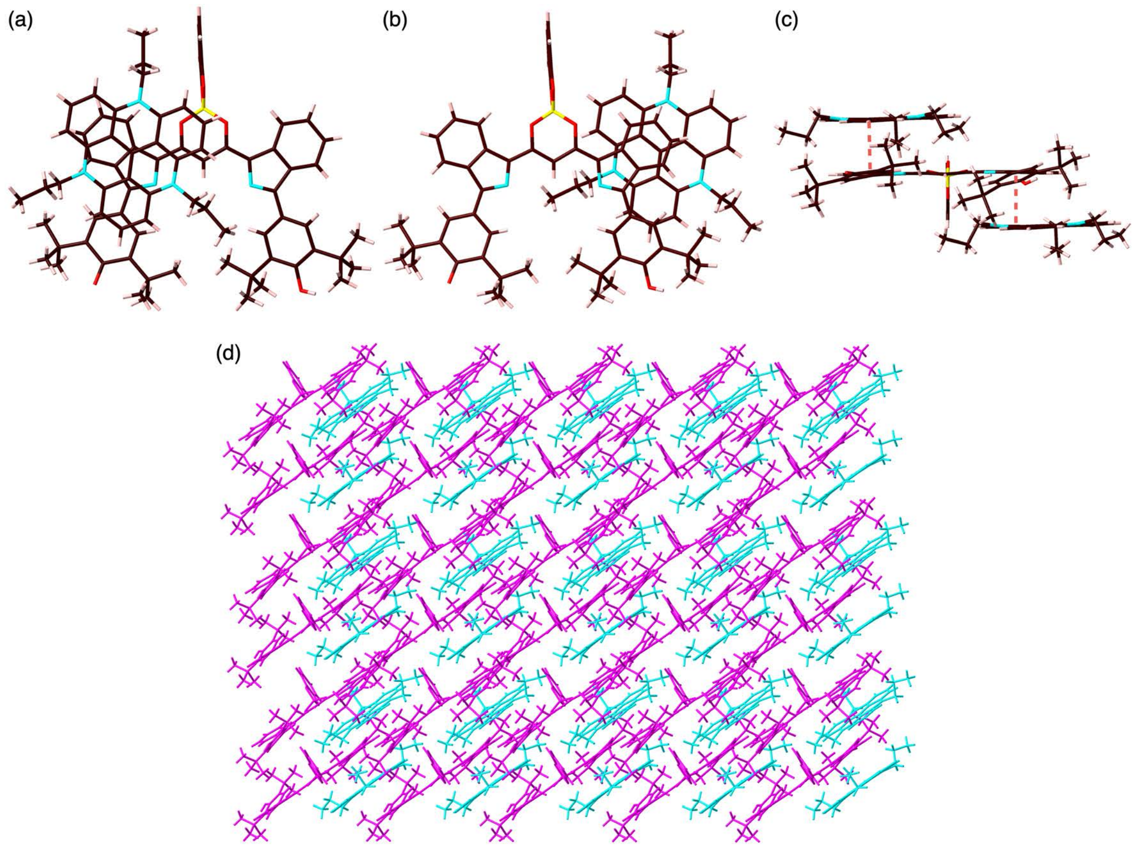

Figure S11 Single-crystal X-ray structure of TATA^{+}-**1c**$^{-}$: (a) top view of the structure where TATA^{+} is located above the diketone framework, (b) top view of the structure where TATA^{+} is located below the diketone framework, and (c) stacked structure with distances of 3.445 and 3.480 Å estimated from the average distances between the mean plane of pyrrole-diketone unit (24 atoms) of **1c**$^{-}$ and the mean plane of TATA^{+} (22 atoms), and (d) packing diagram (magenta: **1c**$^{-}$, cyan: TATA^{+}). Solvent molecules are omitted for clarity. Atom color code in (a–c): brown, pink, yellow, blue, and red refer to carbon, hydrogen, boron, nitrogen, and oxygen, respectively.

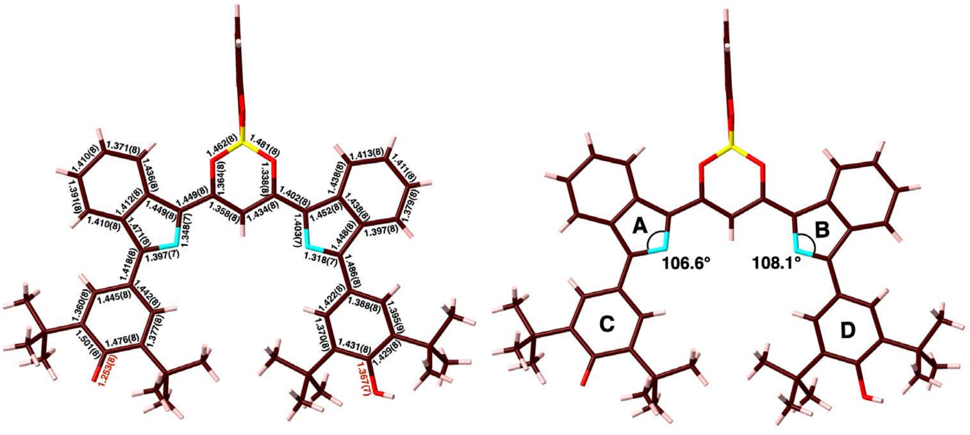

Figure S12 Top view of **1c**$^{-}$ in the crystal structure of TATA^{+}-**1c**$^{-}$ with bond lengths (Å) (left) and C–N–C bond angles in pyrrole units (right). The quinonemethide units and phenol units were characterized by the CO bond lengths. The similar C–N–C bond angles in two pyrrole units indicated that negative charge was delocalized at the core unit. Dihedral angles between pyrrole units (A,B) and quinonemethide/phenol units (C,D) of **1c**$^{-}$ were 11.40°/22.23° (A–C/B–D), suggesting that quinonemethide units formed more flat conformations than phenol units.

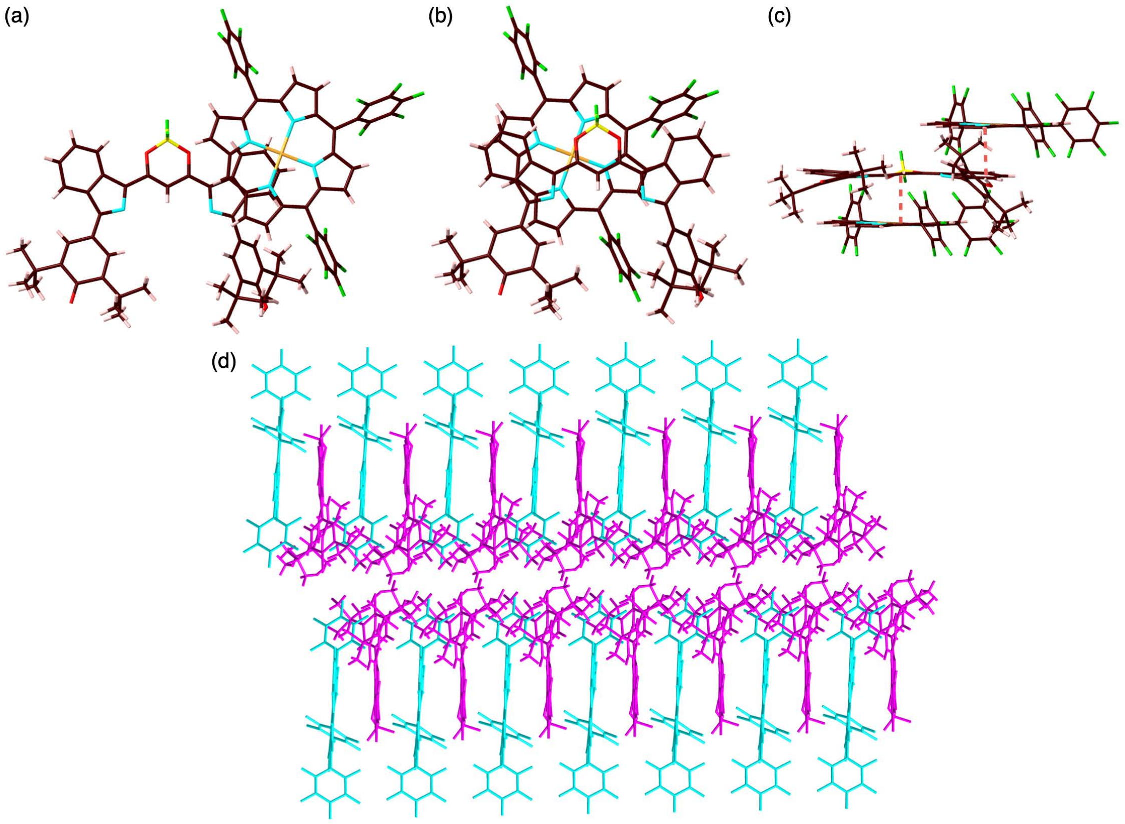

Figure S13 Single-crystal X-ray structure of $3C_6F_5Au^+$-**QPB**$^–$: (a) top view of the structure where $3C_6F_5Au^+$ is located above the diketone framework, (b) top view of the structure where $3C_6F_5Au^+$ is located below the diketone framework, and (c) stacked structure with distances of 3.326 and 3.326 Å estimated from the average distances between the mean plane of pyrrole-diketone unit (24 atoms) of **QPB**$^–$ and the mean plane of $3C_6F_5Au^+$ (25 atoms), and (d) packing diagram (magenta: **QPB**$^–$, cyan: $3C_6F_5Au^+$). Solvent molecules are omitted for clarity. Atom color code in (a–c): brown, pink, yellow, blue, red, green, and orange refer to carbon, hydrogen, boron, nitrogen, oxygen, fluorine, and gold, respectively.

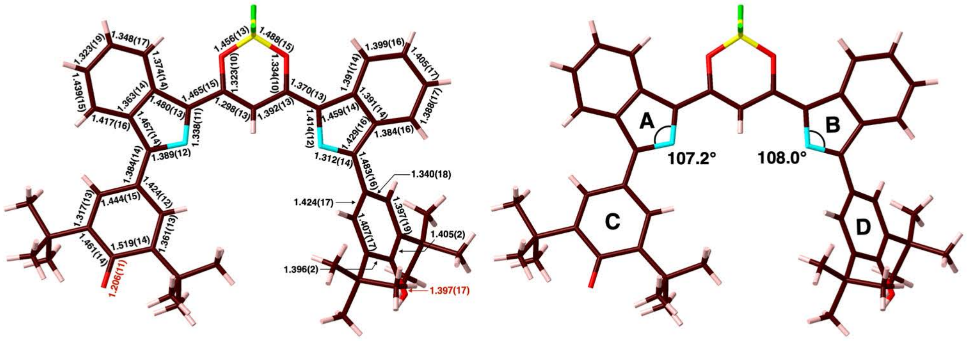

Figure S14 Top view of **QPB**$^–$ in the crystal structure of $3C_6F_5Au^+$-**QPB**$^–$ with bond lengths (Å) (left) and C–N–C bond angles in pyrrole units (right). The quinonemethide units and phenol units were characterized by the CO bond lengths. The similar C–N–C bond angles in two pyrrole units indicated that negative charge was delocalized at the core unit. Dihedral angles between pyrrole units (A,B) and quinonemethide/phenol units (C,D) of **QPB**$^–$ were 15.80°/42.86° (A–C/B–D), suggesting that quinonemethide units formed more flat conformations than phenol units.

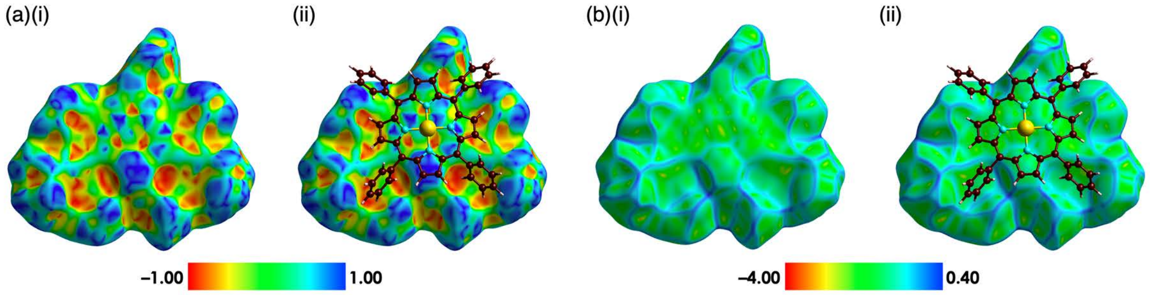

Figure S15 Hirshfeld surfaces[S10,11] of **1a**·Cl^- in the crystal structure of TPPAu$^+$-**1a**·Cl^- mapped over (a) shape-index and (b) curvedness properties: (i) only surface and (ii) surface with a ball-and-stick model of the neighboring TPPAu$^+$. Shape index is a qualitative measure of shape and is sensitive to subtle changes in surface shape, particularly in a flat region by differing by sign represent complementary bumps (blue) and hollows (red), whereas curvedness is a function of the root-mean-square curvature of the surface, and maps of curvedness typically show large regions of green (relatively flat) separated by dark blue edges (large positive curvature). The flat region on the curvedness surface suggested the characteristic mapping pattern for stacking between TPPAu$^+$ and **1a**·Cl^-. Atom color code: brown, pink, blue, and yellow refer to carbon, hydrogen, nitrogen, and gold respectively.

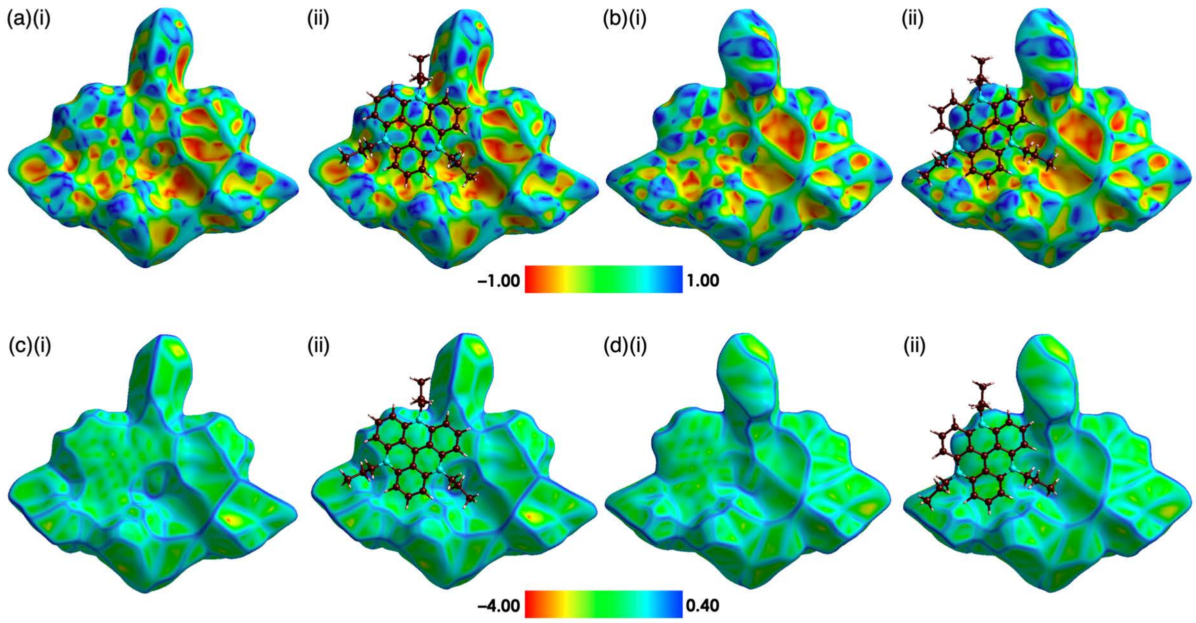

Figure S16 Hirshfeld surfaces[S10,11] of **1c**$^-$ in the crystal structure of TATA$^+$-**1c**$^-$ mapped over (a,b) shape-index and (c,d) curvedness properties: (i) only surface and (ii) surface with a ball-and-stick model of the neighboring TATA$^+$. Shape index is a qualitative measure of shape and is sensitive to subtle changes in surface shape, particularly in a flat region by differing by sign represent complementary bumps (blue) and hollows (red), whereas curvedness is a function of the root-mean-square curvature of the surface, and maps of curvedness typically show large regions of green (relatively flat) separated by dark blue edges (large positive curvature). The flat region on the curvedness surface suggested the characteristic mapping pattern for stacking between TATA$^+$ and **1c**$^-$. Atom color code: brown, pink, and blue refer to carbon, hydrogen, and nitrogen, respectively.

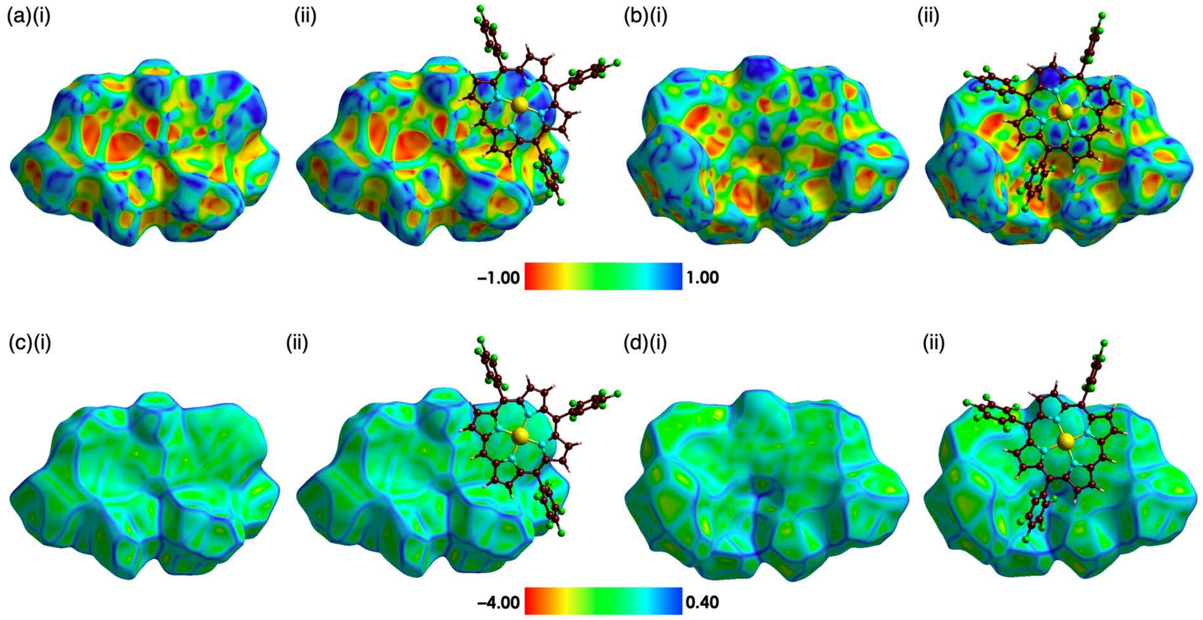

Figure S17 Hirshfeld surfaces[S10,11] of **QPB**$^{-}$ in the crystal structure of $3C_6F_5Au^+$-**QPB**$^{-}$ mapped over (a,b) shape-index and (c,d) curvedness properties: (i) only surface and (ii) surface with a ball-and-stick model of the neighboring $3C_6F_5Au^+$. Shape index is a qualitative measure of shape and is sensitive to subtle changes in surface shape, particularly in a flat region by differing by sign represent complementary bumps (blue) and hollows (red), whereas curvedness is a function of the root-mean-square curvature of the surface, and maps of curvedness typically show large regions of green (relatively flat) separated by dark blue edges (large positive curvature). The flat region on the curvedness surface suggested the characteristic mapping pattern for stacking between $3C_6F_5Au^+$ and **QPB**$^{-}$. Atom color code: brown, pink, blue, green, and yellow refer to carbon, hydrogen, nitrogen, fluorine, and gold, respectively.

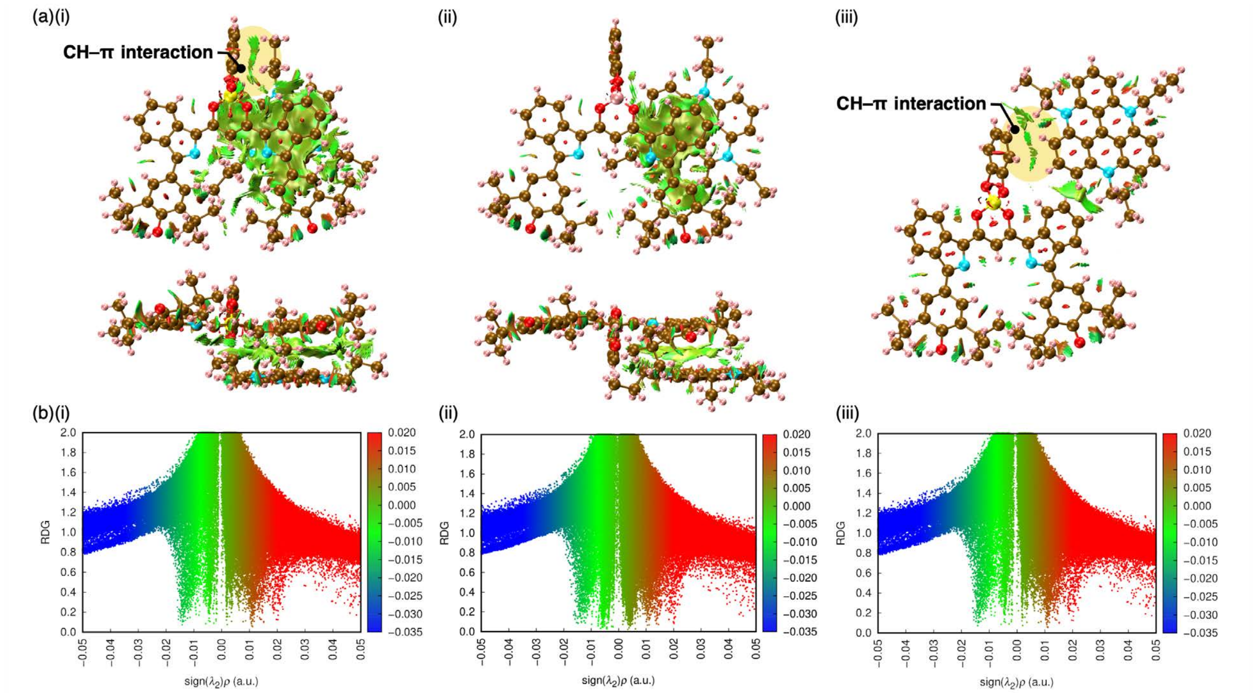

Figure S18 NCI plot[S12,13] of TATA$^+$ and **1c**$^-$ in the crystal structure of TATA$^+$-**1c**$^-$: (a) RDG isosurface and (b) RDG vs *sign*(λ_2)ρ plot, exhibiting (i) top and side views of the stacking of charged π-electronic systems, where TATA$^+$ is located above the quinonemethide framework with the support of CH–π interactions between the alkyl chains of TATA$^+$ and the catecholate unit of **1c**$^-$, with the distances of 2.92–3.16 Å that support the stacking of charged π-electronic systems, (ii) top and side views of the stacking of charged π-electronic systems, where TATA$^+$ is positioned above the phenol framework, and (iii) CH–π interactions between adjacent TATA$^+$ and the catecholate moiety of **1c**$^-$ with the distances of 3.15–3.52 Å. Atom color code: brown, pink, yellow, cyan, and red refer to carbon, hydrogen, boron, nitrogen, and oxygen, respectively.

[S3] a) E. B. Fleischer, A. Laszlo, *Inorg. Nucl. Chem. Lett.* **1969**, *5*, 373–376; b) R. Timkovich, A. Tulinsky, *Inorg. Chem.* **1977**, *16*, 962–963; c) A. M. Shachter, E. B. Fleischer, R. C. Haltiwanger, *Acta Crystallogr. Sect. C* **1987**, *43*, 1876–1878; d) C.-M. Che, R. W.-Y. Sun, W.-Y. Yu, C.-B. Ko, N. Zhu, H. Sun, *Chem. Commun.* **2003**, 1718–1719; e) R. W.-Y. Sun, C. K.-L. Li, D.-L. Ma, J. J. Yan, C.-N. Lok, C.-H. Leung, N. Zhu, C.-M. Che, *Chem. Eur. J.* **2010**, *16*, 3097–3113; f) Y. Haketa, Y. Bando, Y. Sasano, H. Tanaka, N. Yasuda, I. Hisaki, H. Maeda, *iScience* **2019**, *14*, 241–256.

[S4] a) B. W. Laursen, F. C. Krebs, *Angew. Chem. Int. Ed.* **2000**, *39*, 3432–3434; b) Y. Haketa, S. Sasaki, N. Ohta, H. Masunaga, H. Ogawa, N. Mizuno, F. Araoka, H. Takezoe, H. Maeda, *Angew. Chem. Int. Ed.* **2010**, *49*, 10079–10083.

[S5] H. Tanaka, Y. Haketa, N. Yasuda, H. Maeda, *Chem. Asian J.* **2019**, *14*, 2129–2137.

[S6] a) N. Yasuda, H. Murayama, Y. Fukuyama, J. E. Kim, S. Kimura, K. Toriumi, Y. Tanaka, Y. Moritomo, Y. Kuroiwa, K. Kato, H. Tanaka, M. Takata, *J. Synchrotron Rad.* **2009**, *16*, 352–357; b) N. Yasuda, Y. Fukuyama, K. Toriumi, S. Kimura, M. Takata, *AIP Conf. Proc.* **2010**, *1234*, 147–150.

[S7] G. M. Sheldrick, *Acta Crystallogr. A* **2008**, *64*, 112–122.

[S8] a) K. Wakita, *Yadokari-XG*, *Software for Crystal Structure Analyses*, **2001**; b) C. Kabuto, S. Akine, T. Nemoto, E. Kwon, *J. Cryst. Soc. Jpn.* **2009**, *51*, 218–224.

[S9] A. L. Spek, *Acta Crystallogr. C* **2015**, *71*, 9–18.

[S10] P. R. Spackman, M. J. Turner, J. J. McKinnon, S. K. Wolff, D. J. Grimwood, D. Jayatilaka, M. A. Spackman, *J. Appl. Cryst.* **2021**, *54*, 1006–1011.

[S11] a) M. A. Spackman, D. Jayatilaka, *CrystEngComm* **2009**, *11*, 19–32; b) J. J. McKinnon, M. A. Spackman, A. S. Mitchell, *Acta Crystallogr. B* **2004**, *60*, 627–668.

[S12] R. A. Boto, F. Peccati, R. Laplaza, C. Quan, A. Carbone, J.-P. Piquemal, Y. Maday, J. Contreras-García, *NCIPLOT4*, **2020**.

[S13] Reports for *NCIPLOT*: a) E. R. Johnson, S. Keinan, P. Mori-Sánchez, J. Contreras-García, A. J. Cohen, W. Yang, *J. Am. Chem. Soc.* **2010**, *132*, 6498–6506; b) J. Contreras-García, E. R. Johnson, S. Keinan, R. Chaudret, J.-P. Piquemal, D. N. Beratan, W. Yang, *J. Chem. Theory Comput.* **2011**, *7*, 625–632; c) M. Ranjbar, A. Nowroozi, E. Nakhaei, *Comput. Theor. Chem.* **2022**, *1216*, 113867.

3. Theoretical study

DFT calculations. DFT calculations were carried out using the *Gaussian 16* program.[S14]

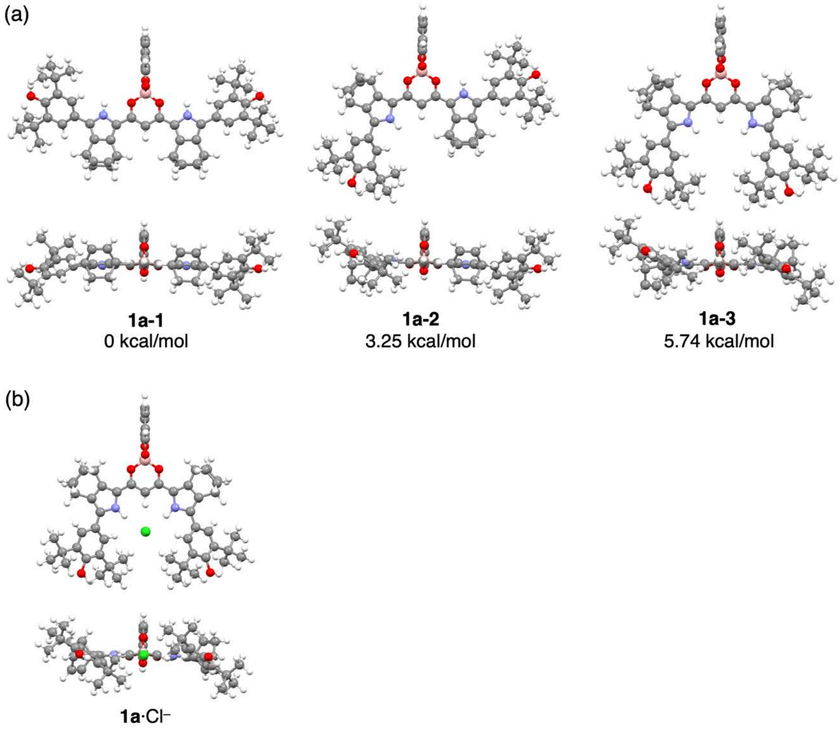

Figure S19 Optimized structures (top and side views) of (a) **1a** (**1a-1**: pyrrole-non-inverted conformation; **1a-2**: singly pyrrole-inverted conformation; **1a-3**: doubly pyrrole-inverted conformation) and (b) **1a**·Cl^- at CAM-B3LYP/6-31+G(d,p). The relative energies of pyrrole-inverted conformations are comparable to those of the corresponding α-phenyl derivative.[S15]

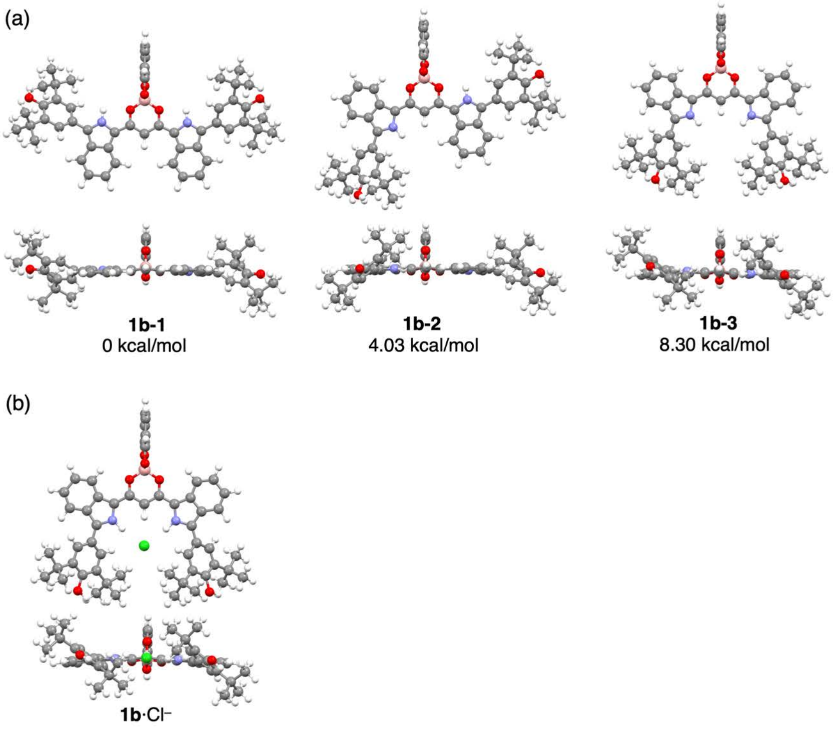

Figure S20 Optimized structures (top and side views) of (a) **1b** (**1b-1**: pyrrole-non-inverted conformation; **1b-2**: singly pyrrole-inverted conformation; **1b-3**: doubly pyrrole-inverted conformation) and (b) **1b**·Cl^- at CAM-B3LYP/6-31+G(d,p). The relative energies of pyrrole-inverted conformations are comparable to those of the corresponding α-phenyl derivative.[S15]

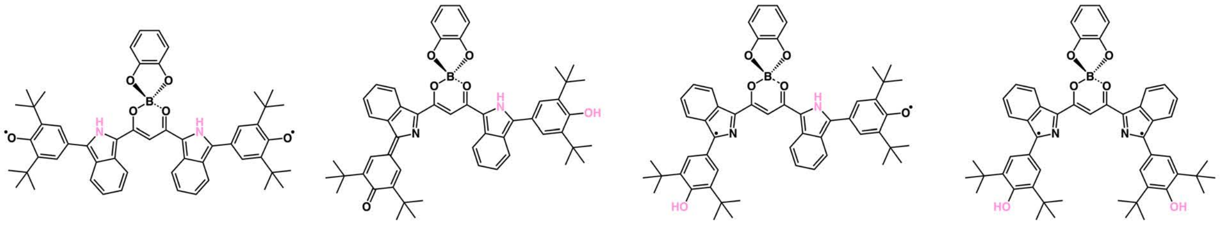

1c$_{NHNH}$ **1c**$_{NHOH}$ **1c**$_{NHOH'}$ **1c**$_{OHOH}$

Figure S21 The labels of **1c**: **1c** could form four tautomers according to the locations of protons on pyrrole N and terminal O: two NH, two OH, one NH and one OH in the one side, and one NH in the one side and one OH in the other side. Four tautomers were labeled as that with two NH (**1c**$_{NHNH}$, a pyrrole-non-inverted open-shell singlet-state conformation as a resonance structure (diradical character: $y_0 = 0.66$, Figure S22b(ii))), that with one NH and one OH in the one side (**1c**$_{NHOH}$, a singly pyrrole-inverted closed-shell conformation as a representative), that with one NH and one OH in the other side (**1c**$_{NHOH}$´, a singly pyrrole-inverted open-shell singlet-state conformation as a resonance structure ($y_0 = 0.49$)), and that with two OH (**1c**$_{OHOH}$, a doubly pyrrole-inverted open-shell singlet-state conformation as a resonance structure ($y_0 = 0.39$)). The atoms bearing a proton are described as the labels in subscript.

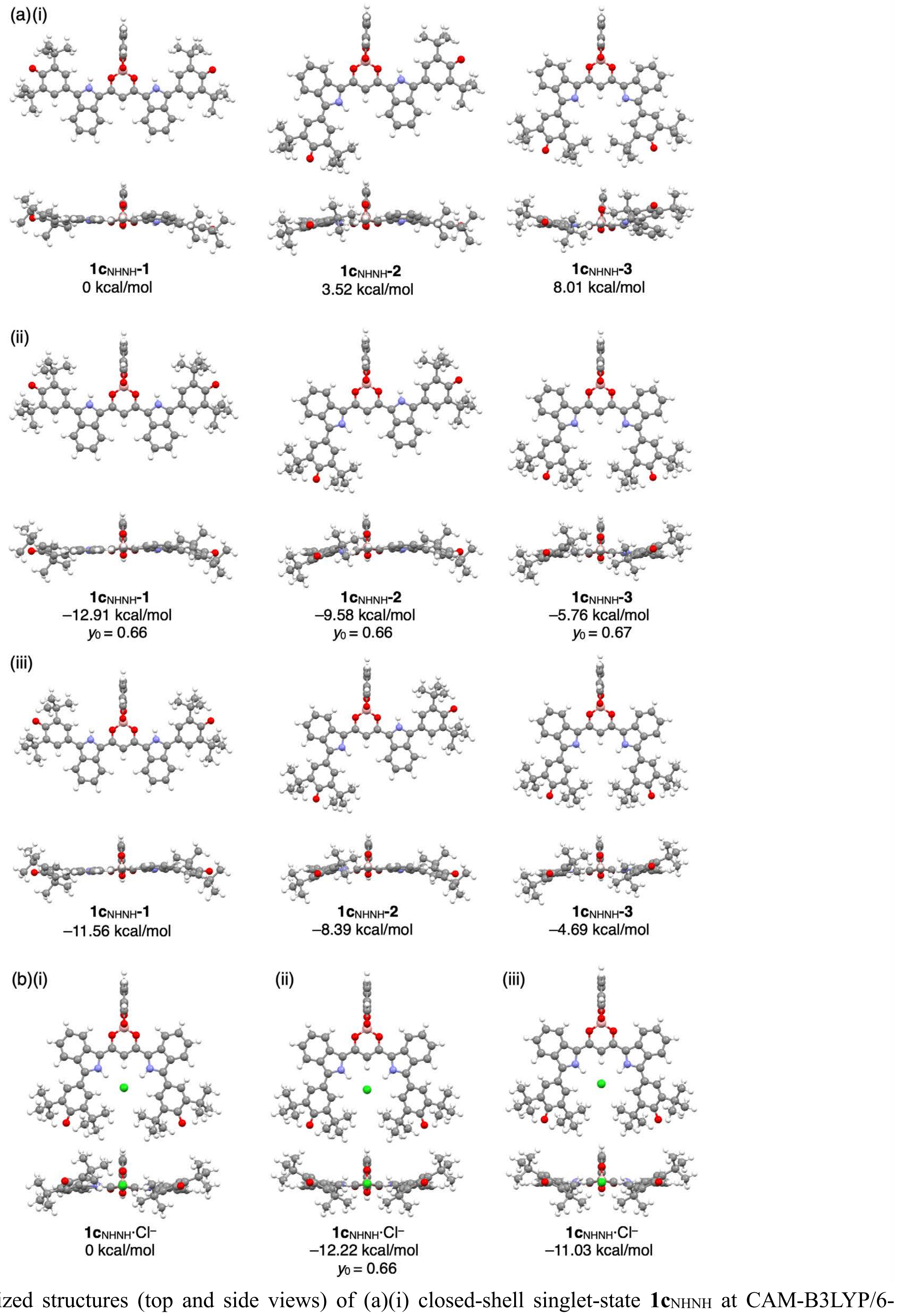

Figure S22 Optimized structures (top and side views) of (a)(i) closed-shell singlet-state **1c**$_{\mathrm{NHNH}}$ at CAM-B3LYP/6-31+G(d,p), (ii) open-shell singlet-state **1c**$_{\mathrm{NHNH}}$ at CAM-UB3LYP/6-31+G(d,p), and (iii) triplet-state **1c**$_{\mathrm{NHNH}}$ at CAM-UB3LYP/6-31+G(d,p) (**1c**$_{\mathrm{NHNH}}$**-1**: pyrrole-non-inverted conformations; **1c**$_{\mathrm{NHNH}}$**-2**: singly pyrrole-inverted conformations; **1c**$_{\mathrm{NHNH}}$**-3**: doubly pyrrole-inverted conformations) and (b)(i) closed-shell singlet-state **1c**$_{\mathrm{NHNH}}$·Cl^- at CAM-B3LYP/6-31+G(d,p), (ii) open-shell singlet-state **1c**$_{\mathrm{NHNH}}$·Cl^- at CAM-UB3LYP/6-31+G(d,p), and (iii) triplet-state **1c**$_{\mathrm{NHNH}}$·Cl^- at CAM-UB3LYP/6-31+G(d,p). The stable structure of closed-shell singlet state (**1c**$_{\mathrm{NHNH}}$**-1** in (a)(i)) was less stable by 12.9 kcal/mol than the stable structure of open-shell singlet state (**1c**$_{\mathrm{NHNH}}$**-1** in (a)(ii)). The calculations for **1c**$_{\mathrm{NHNH}}$·Cl^- were conducted as anion binding is a strategy to control tautomerism.

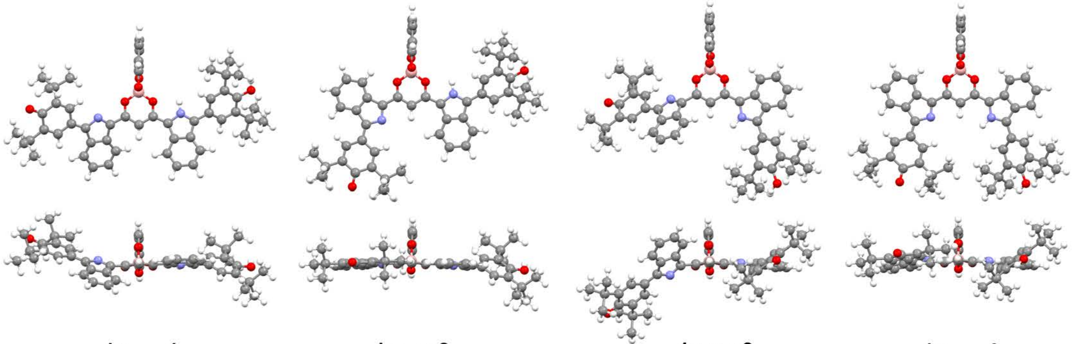

1c$_{\text{NHOH}}$**-1**
3.25 kcal/mol

1c$_{\text{NHOH}}$**-2**
0 kcal/mol

1c$_{\text{NHOH}}$**-3**
10.1 kcal/mol

1c$_{\text{NHOH}}$**-4**
6.09 kcal/mol

Figure S23 Optimized structures (top and side views) of **1c**$_{\text{NHOH}}$ (**1c**$_{\text{NHOH}}$**-1**: pyrrole-non-inverted conformation; **1c**$_{\text{NHOH}}$**-2**,**3**: singly pyrrole-inverted conformations; **1c**$_{\text{NHOH}}$**-4**: doubly pyrrole-inverted conformation) at CAM-B3LYP/6-31+G(d,p). The calculations of open-shell singlet-state **1c**$_{\text{NHOH}}$ at CAM-UB3LYP/6-31+G(d,p) provided the same results for the closed-shell singlet-state calculations. The stable structure of closed-shell singlet state (**1c**$_{\text{NHOH}}$**-2**) was more stable by 4.71 kcal/mol than the stable structure of open-shell singlet state (**1c**$_{\text{NHNH}}$**-1**, Figure S22a(ii)), supporting the formation of **1c**$_{\text{NHOH}}$ in solution.

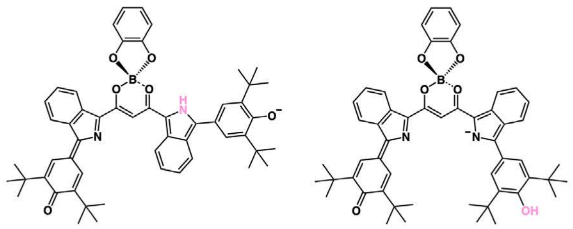

1c$_{\text{NH}}$$^-$ **1c**$_{\text{OH}}$$^-$

Figure S24 The labels of **1c**$^-$: **1c**$^-$ could form two tautomers, shown as representative conformations and resonance structures, according to the locations of protons on pyrrole N (**1c**$_{\text{NH}}$$^-$) and terminal O (**1c**$_{\text{OH}}$$^-$). The atoms bearing a proton are described as labels in subscript.

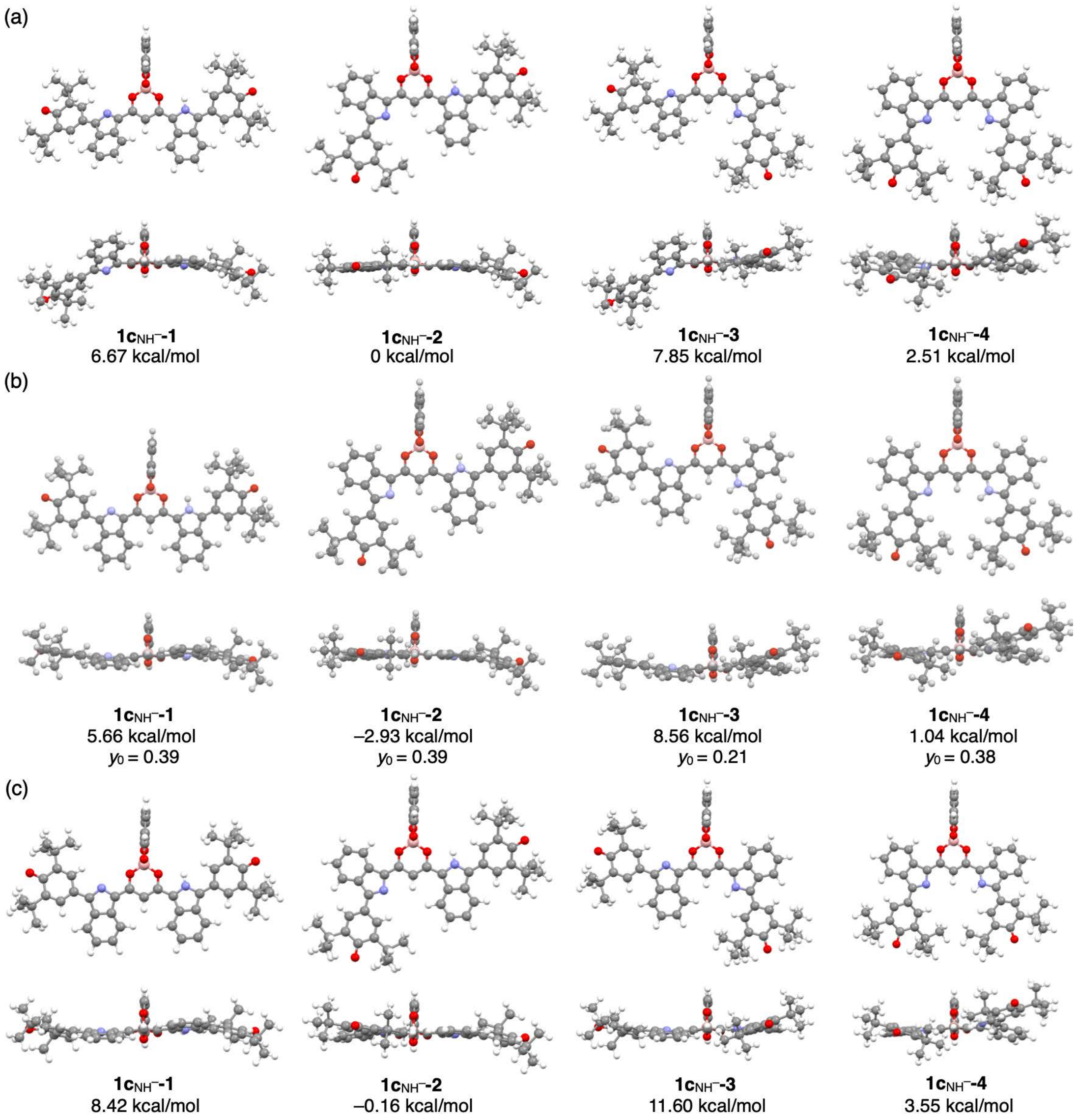

Figure S25 Optimized structures (top and side views) of (a) closed-shell singlet-state **1c**$_{NH}^{-}$ at CAM-B3LYP/6-31+G(d,p), (b) open-shell singlet-state **1c**$_{NH}^{-}$ at CAM-UB3LYP/6-31+G(d,p), and (c) triplet-state **1c**$_{NH}^{-}$ at CAM-UB3LYP/6-31+G(d,p) (**1c**$_{NH}^{-}$**-1**: pyrrole-non-inverted conformations; **1c**$_{NH}^{-}$**-2**,**3**: singly pyrrole-inverted conformations; **1c**$_{NH}^{-}$**-4**: doubly pyrrole-inverted conformations). The stable structure of closed-shell singlet state (**1c**$_{NH}^{-}$**-2** in (a)) was less stable by 2.93 kcal/mol than the stable structure of open-shell singlet state (**1c**$_{NH}^{-}$**-2** in (b)) (Table S2).

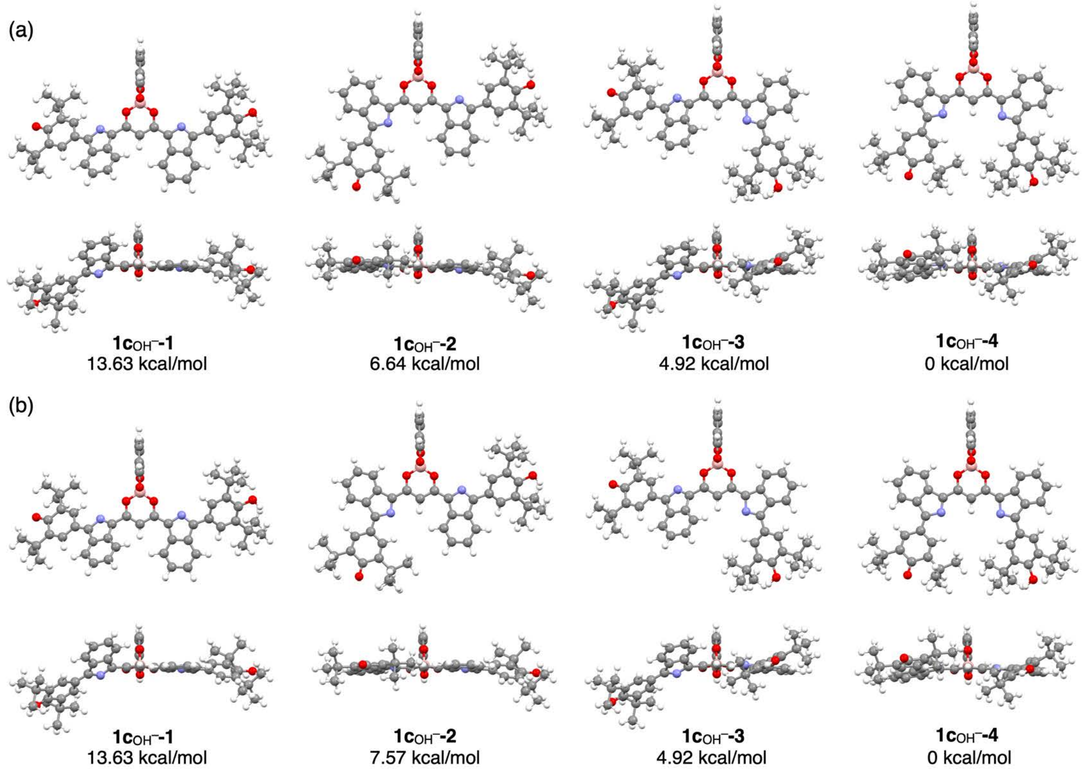

Figure S26 Optimized structures (top and side views) of (a) closed-shell singlet-state **1c**$_{OH}^{-}$ at CAM-B3LYP/6-31+G(d,p), (b) open-shell singlet-state **1c**$_{OH}^{-}$ at CAM-UB3LYP/6-31+G(d,p) (**1c**$_{OH}^{-}$**-1**: pyrrole-non-inverted conformation; **1c**$_{OH}^{-}$**-2**,**3**: singly pyrrole-inverted conformations; **1c**$_{OH}^{-}$**-4**: doubly pyrrole-inverted conformation). The calculations of open-shell singlet-state **1c**$_{OH}^{-}$ at CAM-UB3LYP/6-31+G(d,p) provided the same results for the closed-shell singlet-state calculations except for **1c**$_{OH}^{-}$**-2**. The stable structure (**1c**$_{OH}^{-}$**-4**), in the closed-shell singlet state, was less stable by 2.85 kcal/mol than the stable structure of open-shell singlet state **1c**$_{NH}^{-}$**-2** (Figure S25b and Table S2). On the other hand, **1c**$_{OH}^{-}$**-4**, in the closed-shell singlet state, is more stable by 1.78 kcal/mol than **1c**$_{NH}^{-}$**-2** in the open-shell singlet state at the level of PCM-CAM-UB3LYP/6-31+G(d,p)(CH_2Cl_2)//CAM-UB3LYP/6-31+G(d,p), suggesting that the stability of conformations and resulting electronic states were influenced by the solvent effect.

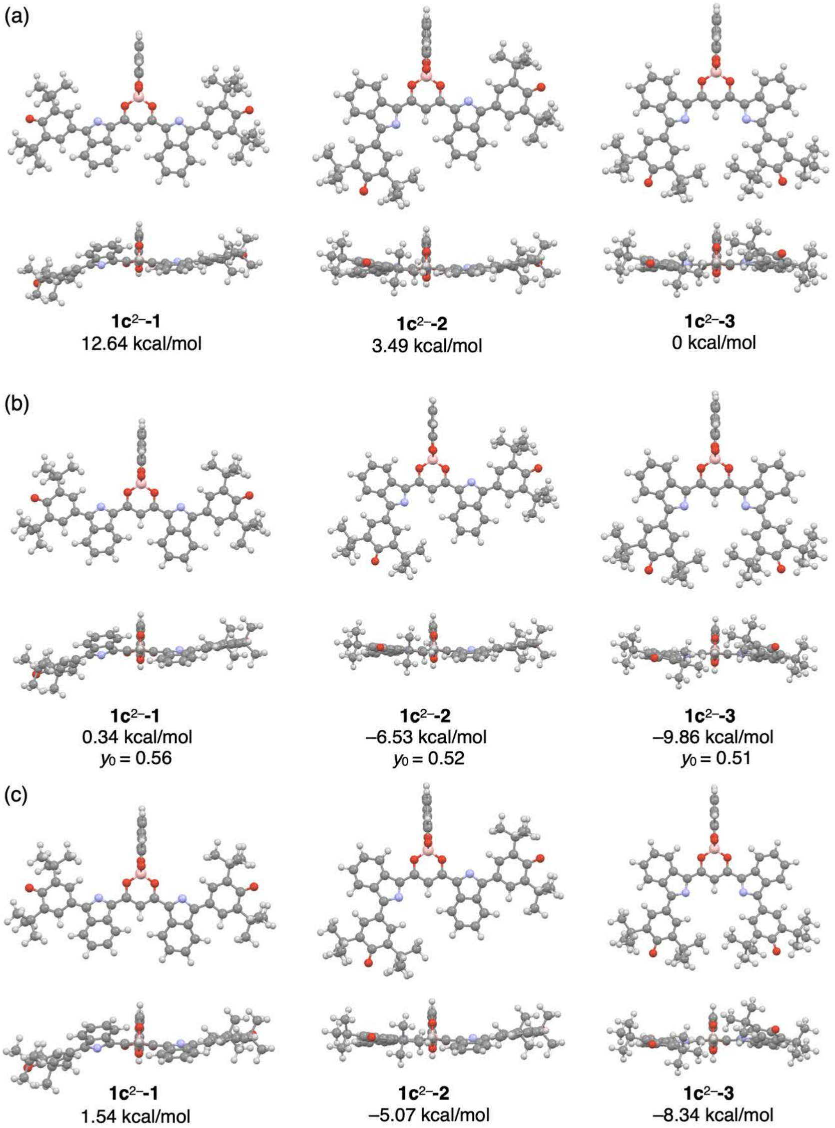

Figure S27 Optimized structures (top and side views) of (a) closed-shell singlet-state **1c**$^{2-}$ at CAM-B3LYP/6-31+G(d,p), (b) open-shell singlet-state **1c**$^{2-}$ at CAM-UB3LYP/6-31+G(d,p), and (c) triplet-state **1c**$^{2-}$ at CAM-UB3LYP/6-31+G(d,p) (**1c**$^{2-}$**-1**: pyrrole-non-inverted conformations; **1c**$^{2-}$**-2**: singly pyrrole-inverted conformations; **1c**$^{2-}$**-3**: doubly pyrrole-inverted conformations). The stable structure of closed-shell singlet state (**1c**$^{2-}$**-3** in (a)) was less stable by 9.86 kcal/mol than the stable structure of open-shell singlet state (**1c**$^{2-}$**-3** in (b)) (Table S2).

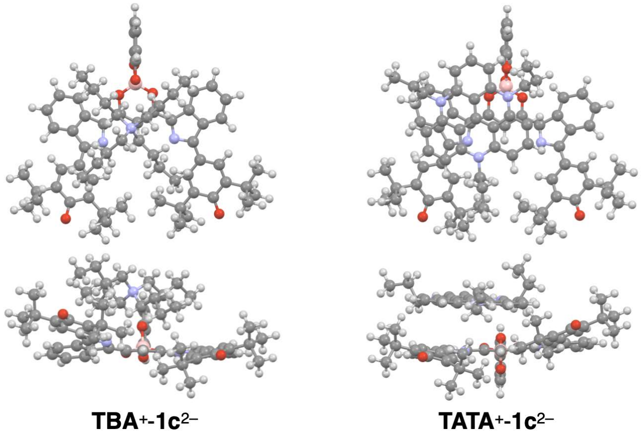

Figure S28 Optimized structures (top and side views) of open-shell singlet-state TBA^{+}-**1c**$^{2-}$ (left) and TATA^{+}-**1c**$^{2-}$ (right) at CAM-UB3LYP/6-31+G(d,p).

Table S2 Calculated energies of **1c**$_{\mathrm{NHNH}}$, **1c**$_{\mathrm{NHOH}}$, **1c**$_{\mathrm{NH}}{}^{-}$, **1c**$_{\mathrm{OH}}{}^{-}$, and **1c**$^{2-}$ for the ground-state optimized geometries at CAM-(U)B3LYP/6-31+G(d,p) (Figure S22–28). According to the theoretical studies, open-shell singlet states of **1c**$_{\mathrm{NHNH}}$ (**1c**$_{\mathrm{NHNH}}$**-1**), **1c**$_{\mathrm{NH}}{}^{-}$ (**1c**$_{\mathrm{NH}}{}^{-}$**-2**), and **1c**$^{2-}$ (**1c**$^{2-}$**-3**) are more stable than their corresponding triplet diradicals.

	heat of formation (hartree)	ΔE (kcal/mol)	<s^2> ^{a}	y_0 ^{b}
**1c**$_{\mathrm{NHNH}}$ (**1c**$_{\mathrm{NHNH}}$**-1**) (closed-shell singlet state)	–2637.361994	17.62	–	–
**1c**$_{\mathrm{NHNH}}$ (**1c**$_{\mathrm{NHNH}}$**-1**) (open-shell singlet state)	–2637.382566	4.71	1.197100	0.66
**1c**$_{\mathrm{NHNH}}$ (**1c**$_{\mathrm{NHNH}}$**-1**) (triplet state)	–2637.380421	6.06	2.176895	–
**1c**$_{\mathrm{NHOH}}$ (**1c**$_{\mathrm{NHOH}}$**-2**) (closed-shell singlet state)	–2637.395264	0 (most stable)	–	–
**1c**$_{\mathrm{NH}}{}^{-}$ (**1c**$_{\mathrm{NH}}{}^{-}$**-2**) (closed-shell singlet state)	–2636.887924	2.93	–	–
**1c**$_{\mathrm{NH}}{}^{-}$ (**1c**$_{\mathrm{NH}}{}^{-}$**-2**) (open-shell singlet state)	–2636.892589	0 (most stable)	1.074826	0.39
**1c**$_{\mathrm{NH}}{}^{-}$ (**1c**$_{\mathrm{NH}}{}^{-}$**-2**) (triplet state)	–2636.888185	2.76	2.130776	–
**1c**$_{\mathrm{OH}}{}^{-}$ (**1c**$_{\mathrm{OH}}{}^{-}$**-4**) (closed-shell singlet state)	–2636.888043	2.85	–	–
**1c**$^{2-}$ (**1c**$^{2-}$**-3**) (closed-shell singlet state)	–2636.296186	9.86	–	–
**1c**$^{2-}$ (**1c**$^{2-}$**-3**) (open-shell singlet state)	–2636.311893	0 (most stable)	1.004398	0.51
**1c**$^{2-}$ (**1c**$^{2-}$**-3**) (triplet state)	–2636.309473	1.52	2.062113	–

^{a} Spin expectation values ($s(s + 1)$). ^{b} Singlet diradical character.

Table S3 Calculated energies of **1c**$_{\mathrm{NHNH}}$·Cl$^-$ for the ground-state optimized geometries at CAM-(U)B3LYP/6-31+G(d,p) (Figure S22). According to the theoretical studies, singlet diradical (open-shell ground state) of **1c**$_{\mathrm{NHNH}}$·Cl$^-$ is more stable than the corresponding triplet diradical (open-shell ground state). As described in the caption of Figure S22, the calculations for **1c**$_{\mathrm{NHNH}}$·Cl$^-$ were conducted as anion binding, like **PB**s,[S9] is a strategy to control tautomerism and resulting diradical properties.

	heat of formation (hartree)	ΔE (kcal/mol)	<s^2> ^{a}	y_0 ^{b}
**1c**$_{\mathrm{NHNH}}$·Cl$^-$ (closed-shell singlet state)	–3097.718375	12.22	–	–
**1c**$_{\mathrm{NHNH}}$·Cl$^-$ (open-shell singlet state)	–3097.737843	0 (most stable)	1.169269	0.66
**1c**$_{\mathrm{NHNH}}$·Cl$^-$ (triplet state)	–3097.735959	1.18	2.159324	–

^{a} Spin expectation values ($s(s + 1)$). ^{b} Singlet diradical character.

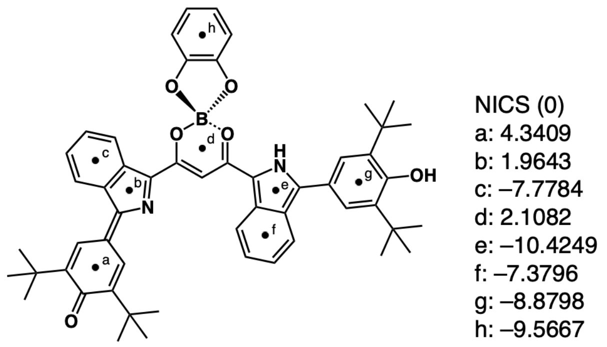

Figure S29 NICS(0) values (ppm) of closed-shell singlet-state **1c** (**1c**$_{\mathrm{NHOH}}$**-2**, Figure S23) estimated at CAM-B3LYP/6-31+G(d,p). The NICS value of **1c** at the cross-linking unit is larger than that of **QPB**, suggesting that the cross-linking unit was influenced by the catechol unit.

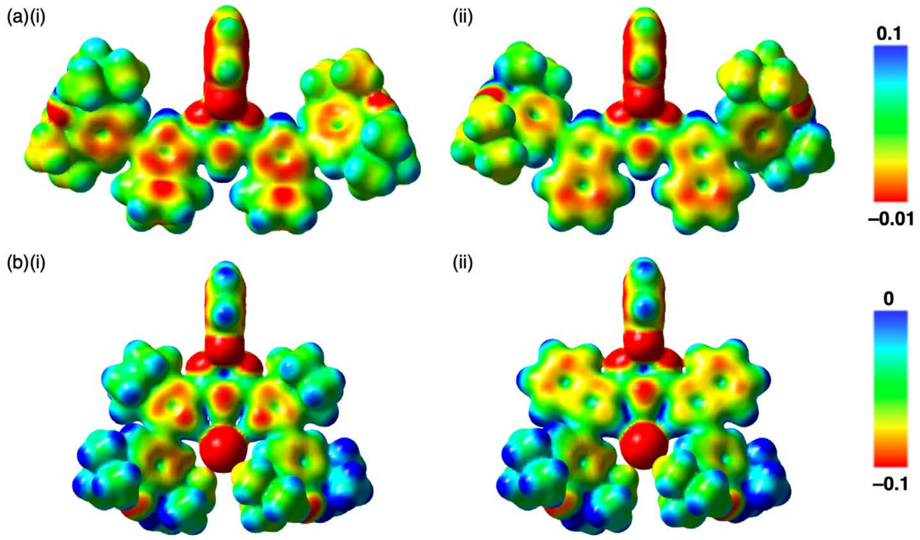

Figure S30 Electrostatic potential (ESP) mapping of (a)(i) **1a** and (ii) **1b** and (b)(i) **1a**·Cl^- and (ii) **1b**·Cl^- estimated at CAM-B3LYP/6-31+G(d,p) (δ = 0.01).

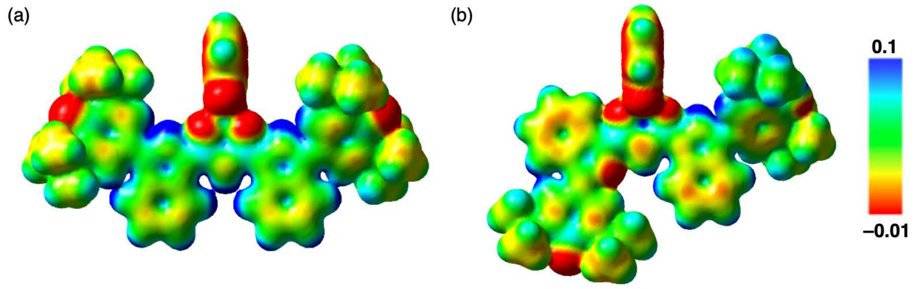

Figure S31 ESP mapping of (a) open-shell singlet-state **1c**$_{\rm NHNH}$ (**1c**$_{\rm NHNH}$**-1**, Figure S22a(ii)) and (b) closed-shell singlet-state **1c**$_{\rm NHOH}$ (**1c**$_{\rm NHOH}$**-2**, Figure S23) estimated at CAM-(U)B3LYP/6-31+G(d,p) (δ = 0.01).

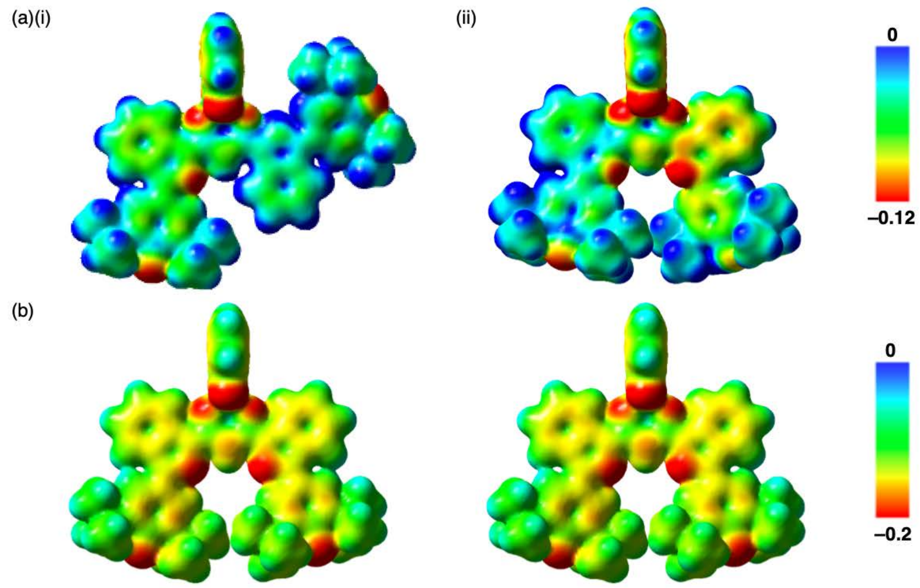

Figure S32 ESP mapping of (a)(i) open-shell singlet-state **1c**$_{\rm NH}^{-}$ (**1c**$_{\rm NH}^{-}$**-2**, Figure S25b) and (ii) closed-shell singlet-state **1c**$_{\rm OH}^{-}$ (**1c**$_{\rm OH}^{-}$**-4**, Figure S26a) and (b) open-shell singlet-state (left) and triplet-state (right) **1c**$^{2-}$ (**1c**$^{2-}$**-3**, Figure S27b,c, respectively) estimated at CAM-(U)B3LYP/6-31+G(d,p) (δ = 0.01). According to the ESP mapping of **1c**$^{2-}$, negative charge was delocalized.

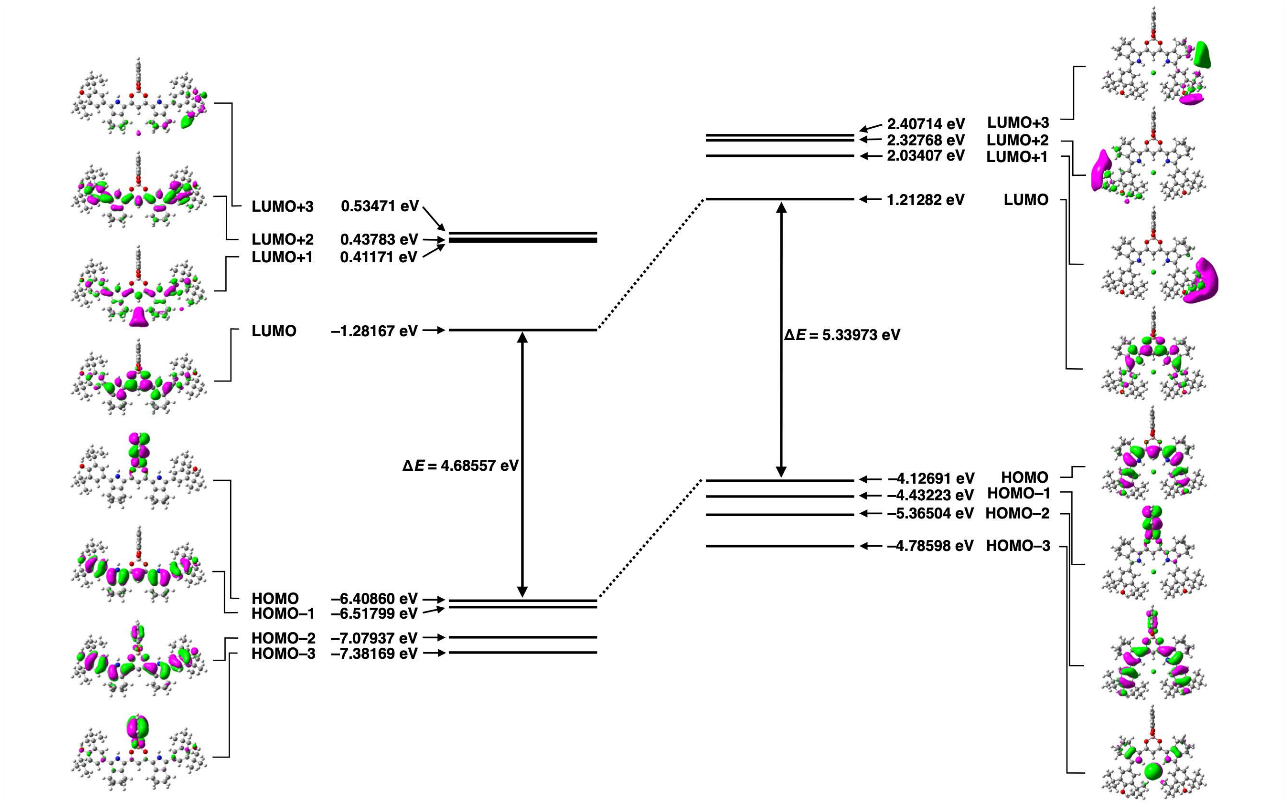

Figure S33 Molecular orbitals (HOMO/LUMO) of **1a** (left) and **1a**·Cl⁻ (right) estimated at CAM-B3LYP/6-31+G(d,p).

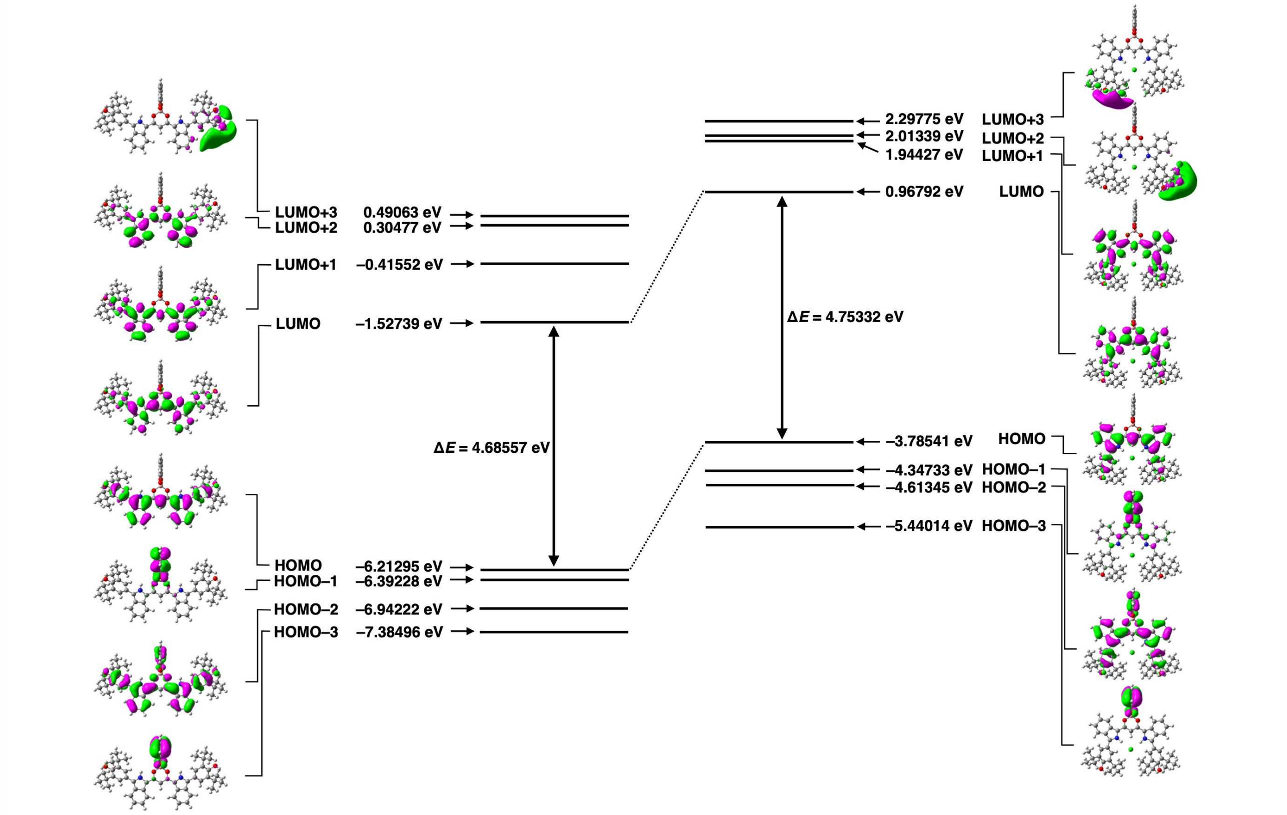

Figure S34 Molecular orbitals (HOMO/LUMO) of **1b** (left) and **1b**·Cl⁻ (right) estimated at CAM-B3LYP/6-31+G(d,p).

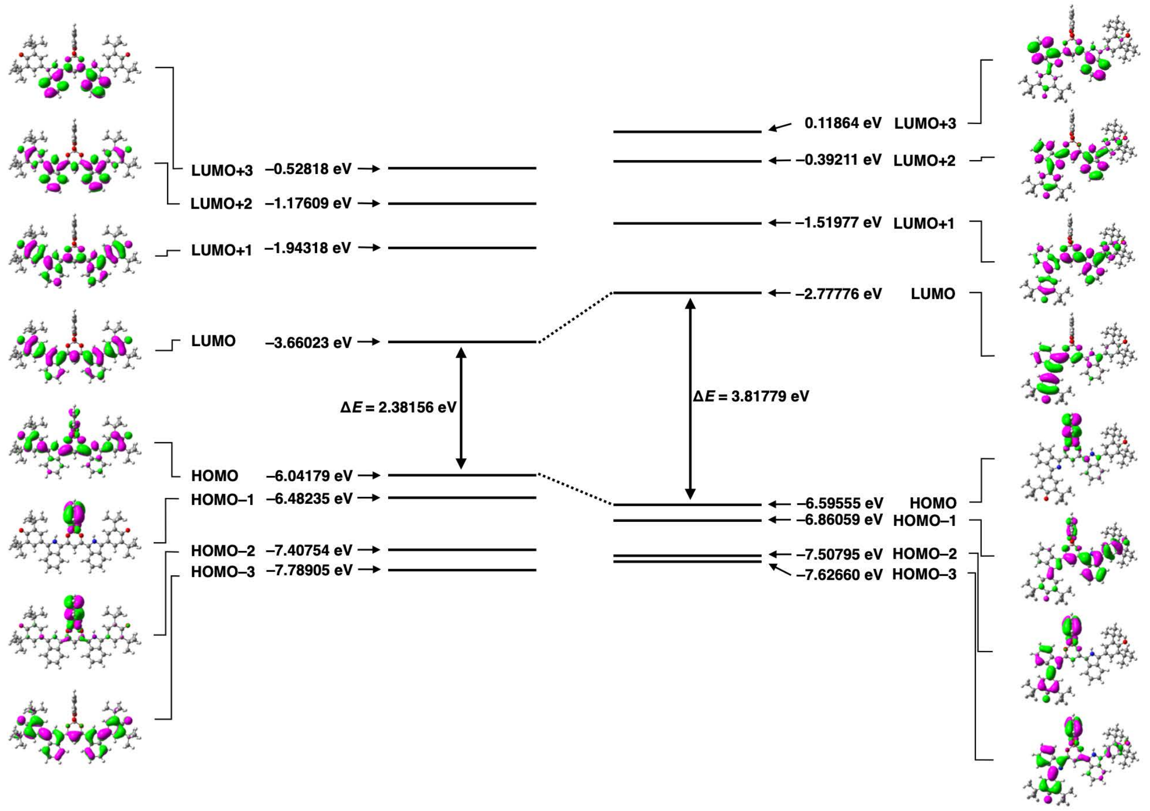

Figure S35 Molecular orbitals (HOMO/LUMO) of closed-shell singlet-state **1c**$_{\mathrm{NHNH}}$ (left) (**1c**$_{\mathrm{NHNH}}$**-1**, Figure S22a(i)) and **1c**$_{\mathrm{NHOH}}$ (right) (**1c**$_{\mathrm{NHOH}}$**-2**, Figure S23) estimated at CAM-B3LYP/6-31+G(d,p). The HOMO of **1c**$_{\mathrm{NHOH}}$ was localized at the catechol unit, whereas the LUMO of **1c**$_{\mathrm{NHOH}}$ was localized at the quinonemethide–pyrrole$_{\mathrm{N}}$ unit. Thus, the LUMO level of **1c** was more affected by the oxidation than the HOMO level.

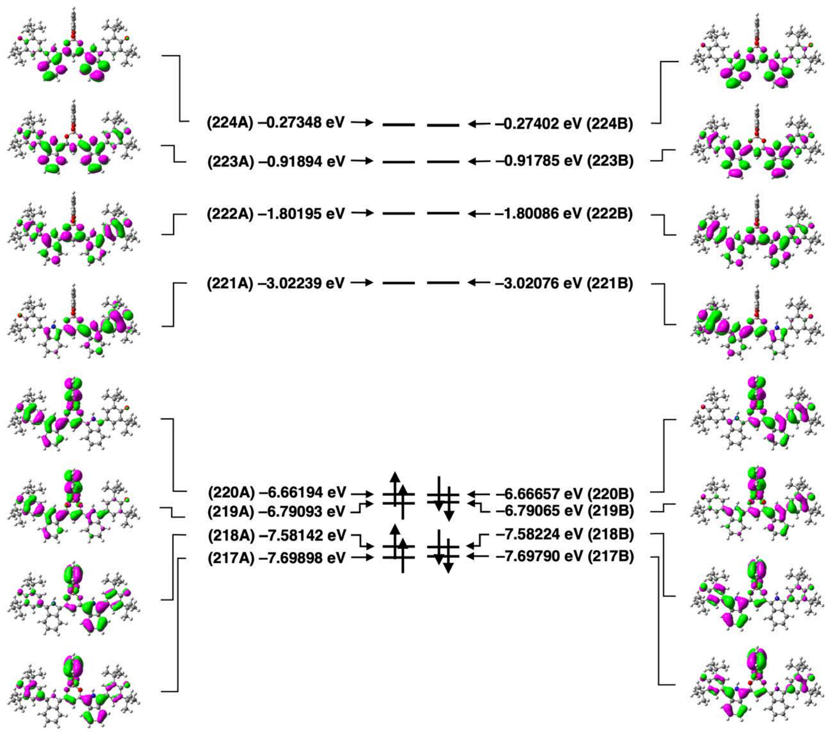

Figure S36 Molecular orbitals (HOMO/LUMO) of open-shell singlet-state **1c**$_{\mathrm{NHNH}}$ (**1c**$_{\mathrm{NHNH}}$**-1**, Figure S22a(ii)) with α-spin (left) and β-spin (right) estimated at CAM-UB3LYP/6-31+G(d,p) for the comparison to Figure S35.

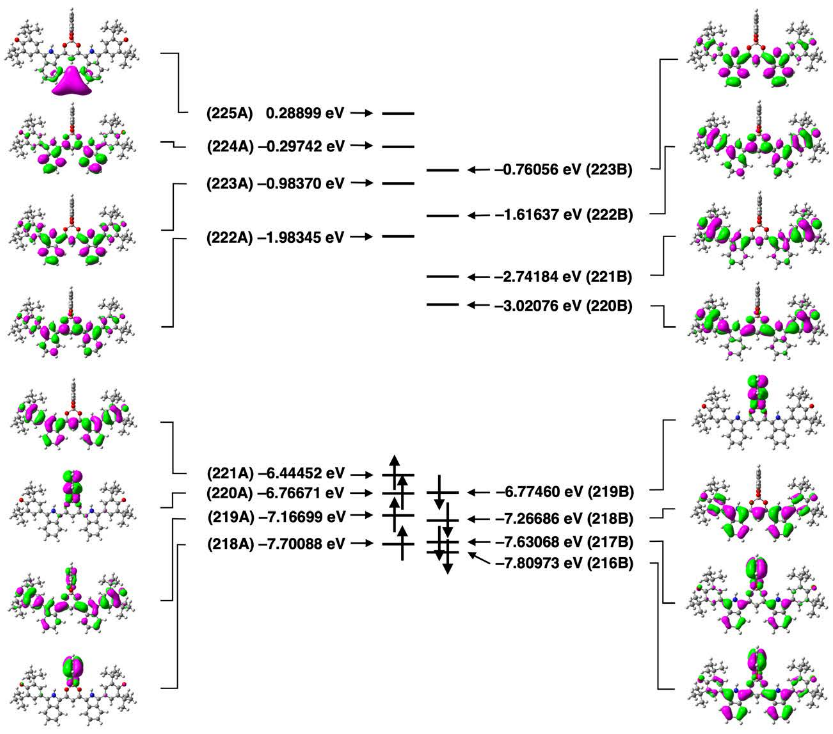

Figure S37 Molecular orbitals (HOMO/LUMO) of triplet-state **1c**$_{\mathrm{NHNH}}$ (**1c**$_{\mathrm{NHNH}}$**-1**, Figure S22a(iii)) with α-spin (left) and β-spin (right) estimated at CAM-UB3LYP/6-31+G(d,p) for the comparison to Figure S35.

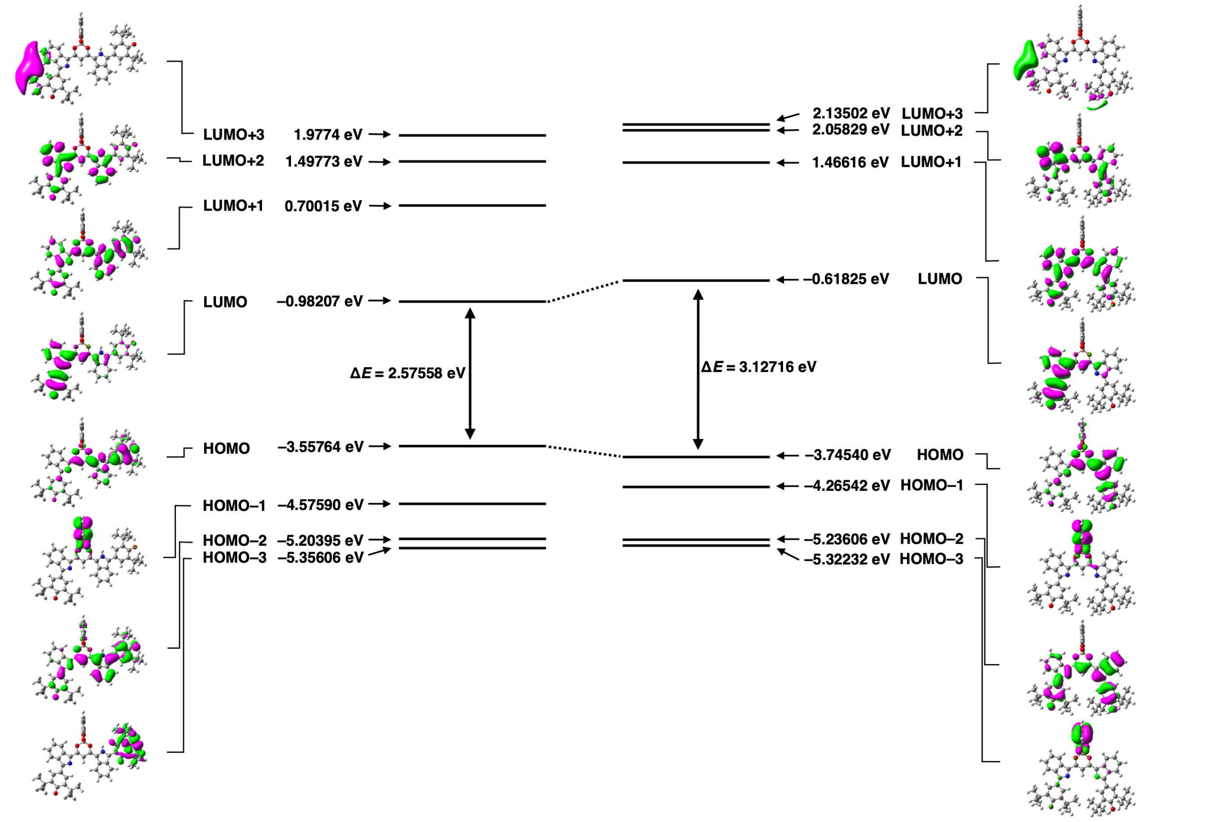

Figure S38 Molecular orbitals (HOMO/LUMO) of closed-shell singlet-state **1c**$_{NH}$$^{-}$ (**1c**$_{NH}$$^{-}$**-2**, Figure S25a) (left) and **1c**$_{OH}$$^{-}$ (**1c**$_{OH}$$^{-}$**-4**, Figure S26a) (right) estimated at CAM-B3LYP/6-31+G(d,p).

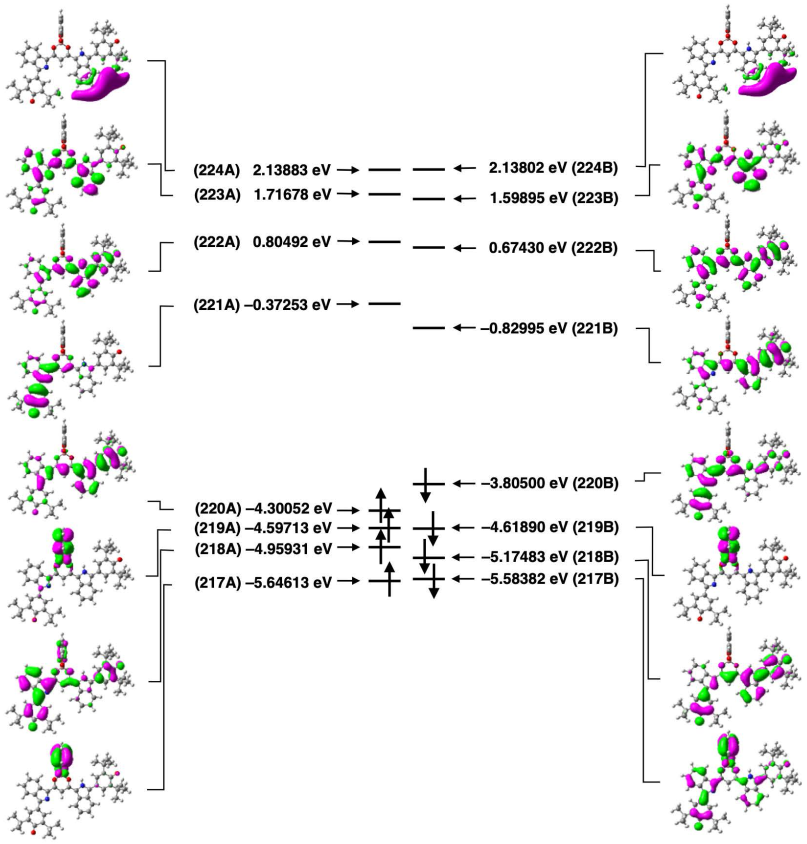

Figure S39 Molecular orbitals (HOMO/LUMO) of open-shell singlet-state **1c**$_{NH}$$^{-}$ (**1c**$_{NH}$$^{-}$**-2**, Figure S25b) with α-spin (left) and β-spin (right) estimated at CAM-UB3LYP/6-31+G(d,p) for the comparison to Figure S38.

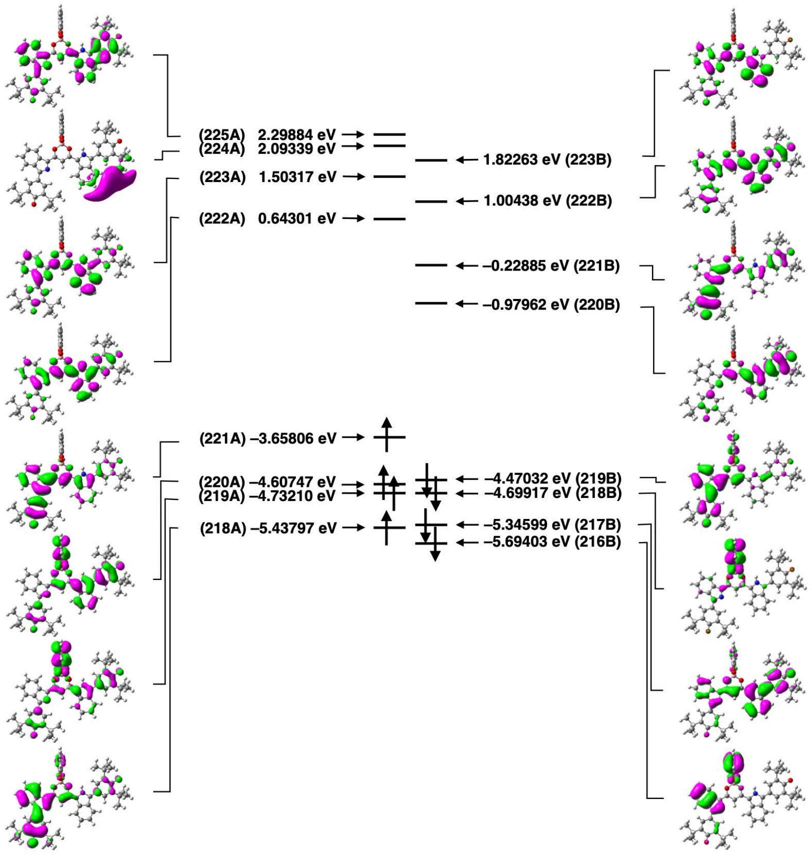

Figure S40 Molecular orbitals (HOMO/LUMO) of triplet-state **1c**$_{NH}^{-}$ (**1c**$_{NH}^{-}$**-2**, Figure S25c) with α-spin (left) and β-spin (right) estimated at CAM-UB3LYP/6-31+G(d,p) for the comparison to Figure S38.

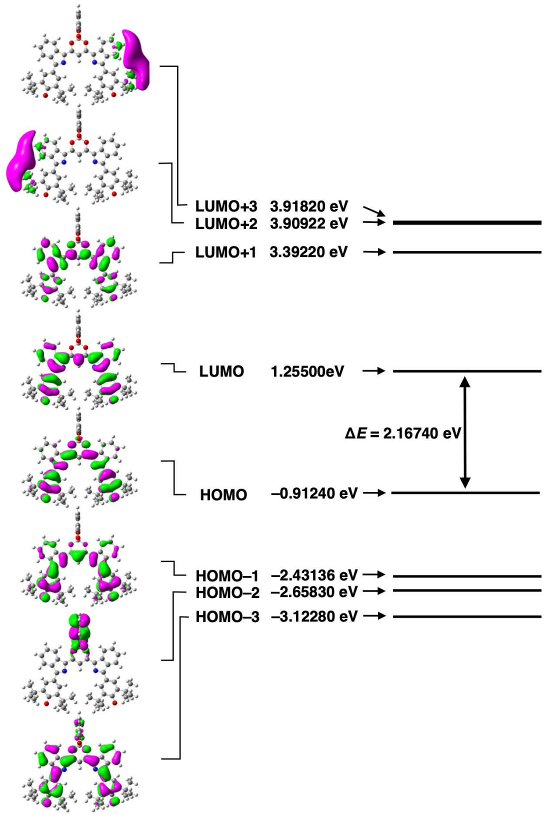

Figure S41 Molecular orbitals (HOMO/LUMO) of closed-shell singlet-state **1c**$^{2-}$ (**1c**$^{2-}$**-3**, Figure S27a) estimated at CAM-B3LYP/6-31+G(d,p).

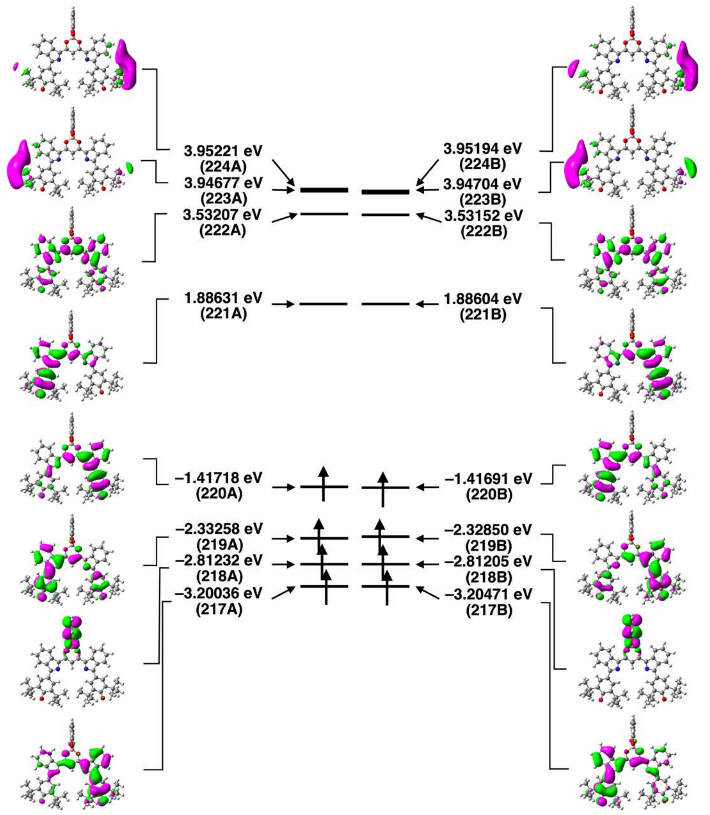

Figure S42 Molecular orbitals (HOMO/LUMO) of open-shell singlet-state **1c**$^{2-}$ (**1c**$^{2-}$**-3**, Figure S27b) with α-spin (left) and β-spin (right) estimated at CAM-UB3LYP/6-31+G(d,p) for the comparison to Figure S41.

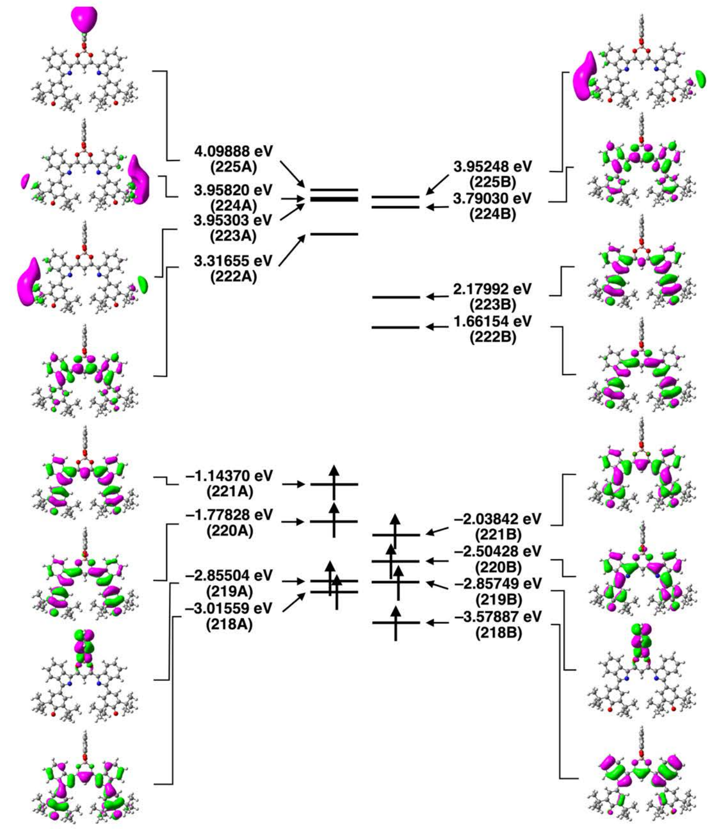

Figure S43 Molecular orbitals (HOMO/LUMO) of triplet-state **1c**$^{2-}$ (**1c**$^{2-}$**-3**, Figure S27c) with α-spin (left) and β-spin (right) estimated at CAM-UB3LYP/6-31+G(d,p) for the comparison to Figure S41.

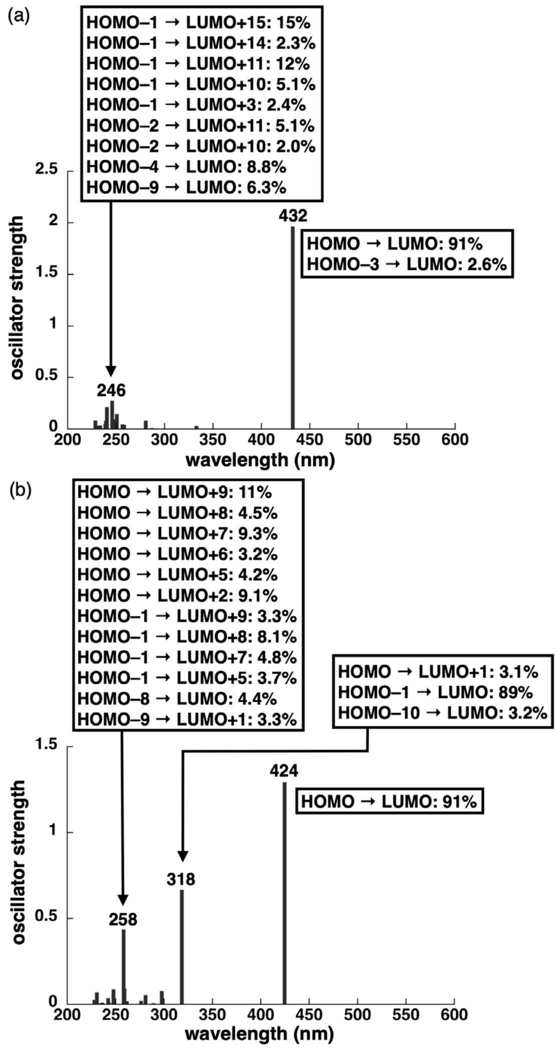

Figure S44 TD-DFT-based UV/vis absorption stick spectra of (a) **1a** and (b) **1a**·Cl^- with the transitions correlated with molecular orbitals estimated at PCM-CAM-B3LYP/6-31+G(d,p)(CH_2Cl_2)//CAM-B3LYP/6-31+G(d,p).

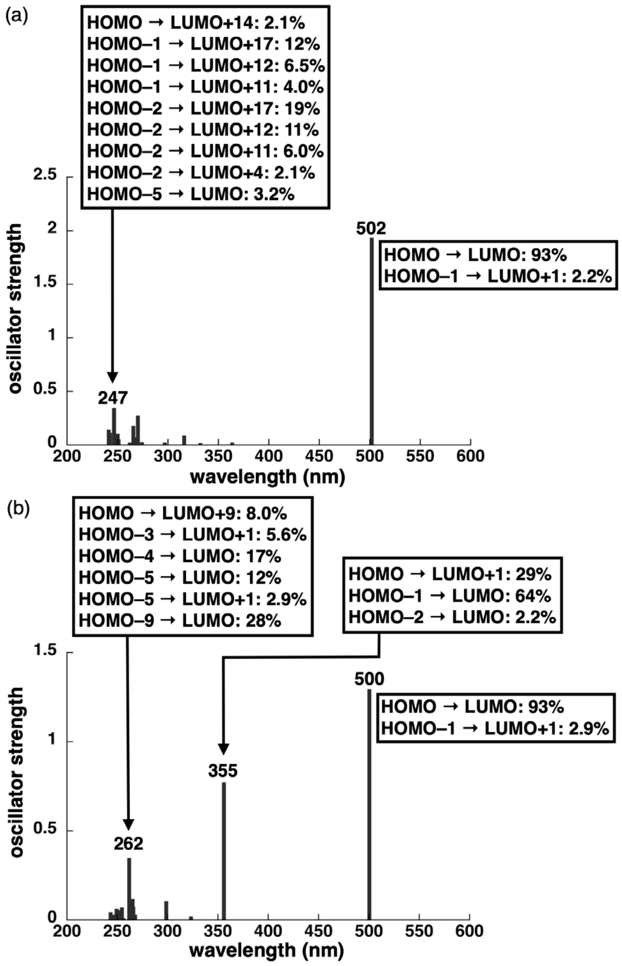

Figure S45 TD-DFT-based UV/vis absorption stick spectra of (a) **1b** and (b) **1b**·Cl^- with the transitions correlated with molecular orbitals estimated at PCM-CAM-B3LYP/6-31+G(d,p)(CH_2Cl_2)//CAM-B3LYP/6-31+G(d,p).

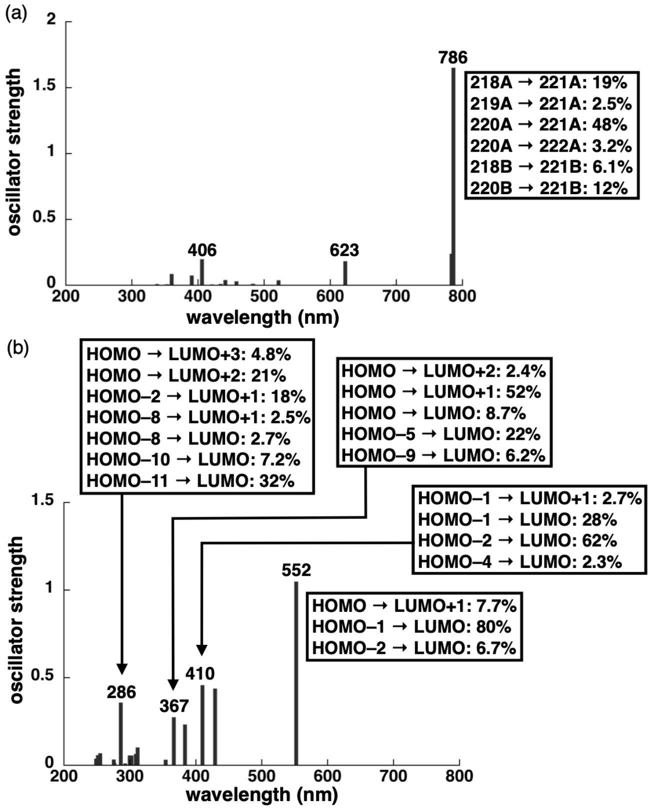

Figure S46 TD-DFT-based UV/vis absorption stick spectra of (a) open-shell singlet-state **1c**$_{\mathrm{NHNH}}$ (**1c**$_{\mathrm{NHNH}}$**-1**, Figure S22a(ii)) with the transitions correlated with molecular orbitals estimated at PCM-CAM-UB3LYP/6-31+G(d,p)(CH_2Cl_2) //CAM-UB3LYP/6-31+G(d,p) and (b) closed-shell singlet-state **1c**$_{\mathrm{NHOH}}$ (**1c**$_{\mathrm{NHOH}}$**-2**, Figure S23) with the transitions correlated with molecular orbitals estimated at PCM-CAM-B3LYP/6-31+G(d,p)(CH_2Cl_2)//CAM-B3LYP/6-31+G(d,p).

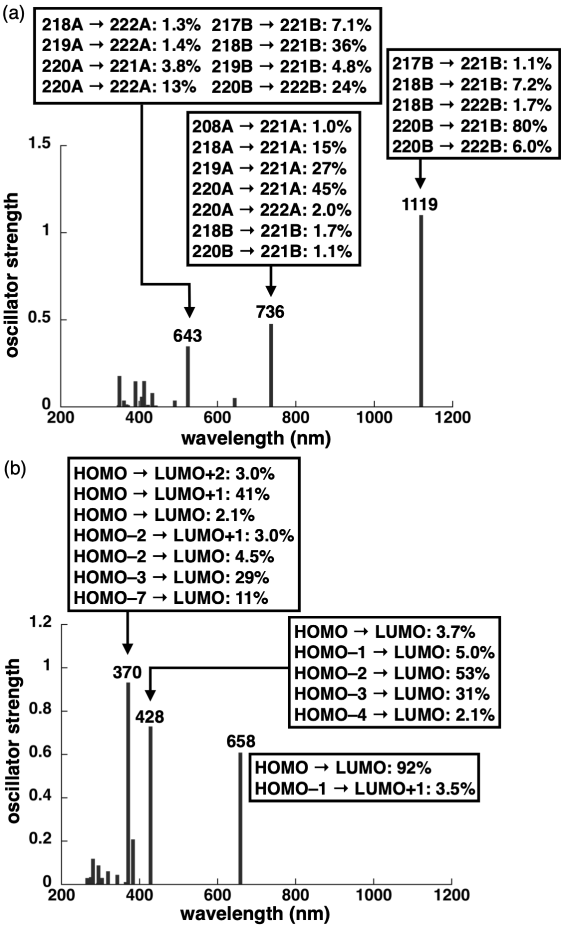

Figure S47 TD-DFT-based UV/vis absorption stick spectra of (a) open-shell singlet-state **1c**$_{NH}^{-}$ (**1c**$_{NH}^{-}$**-2**, Figure S25b) with the transitions correlated with molecular orbitals estimated at PCM-CAM-UB3LYP/6-31+G(d,p)(CH_2Cl_2)//CAM-UB3LYP/6-31+G(d,p) and (b) closed-shell singlet-state **1c**$_{OH}^{-}$ (**1c**$_{OH}^{-}$**-4**, Figure S26a) with the transitions correlated with molecular orbitals estimated at PCM-CAM-B3LYP/6-31+G(d,p)(CH_2Cl_2)//CAM-B3LYP/6-31+G(d,p).

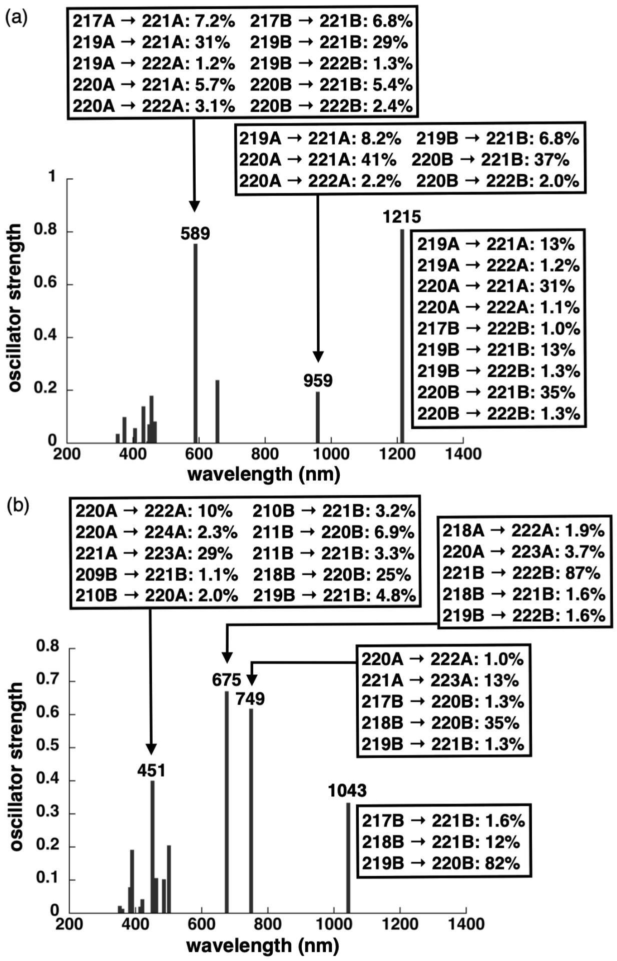

Figure S48 TD-DFT-based UV/vis absorption stick spectra of (a) open-shell singlet-state **1c**$^{2-}$ (**1c**$^{2-}$**-3**, Figure S27b) and (b) triplet-state **1c**$^{2-}$ (**1c**$^{2-}$**-3**, Figure S27c) with the transitions correlated with molecular orbitals estimated at PCM-CAM-UB3LYP/6-31+G(d,p)(CH_2Cl_2)//CAM-UB3LYP/6-31+G(d,p). Calculated excitation energies of ion species deviate from the observed values as an estimated excitation state at 1215 nm would correspond to an experimental absorption at 1546 nm (Figure S54b).

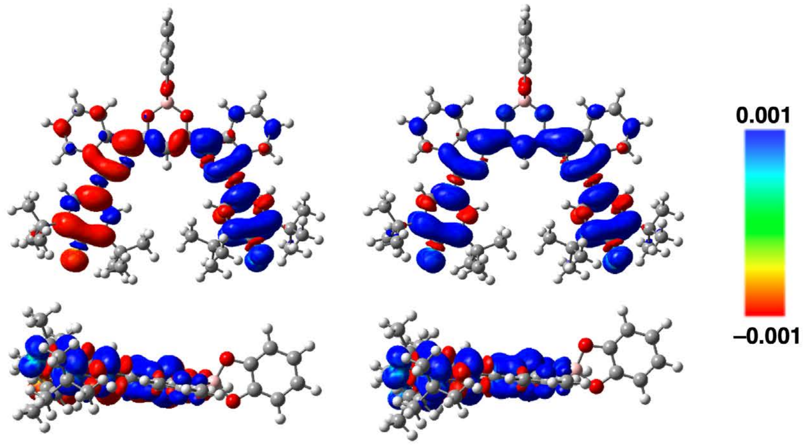

Figure S49 Spin densities of **1c**$^{2-}$ (**1c**$^{2-}$**-3**, Figure S27b,c) (top and side views) (left: open-shell singlet state, right: triplet state) estimated at CAM-UB3LYP/6-31+G(d,p) (δ = 0.001). The singlet and triplet spin densities were delocalized at terminal aryl, oxygen units and the bridging unit but the spin is not distributed at catechol unit.

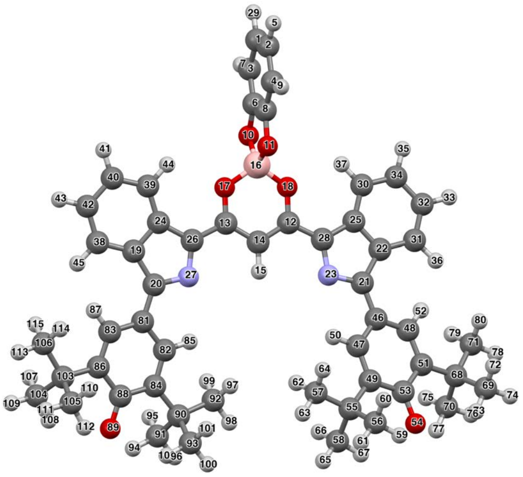

Figure S50 Molecular atom labels of **1c**$^{2-}$ (**1c**$^{2-}$**-3**) for the discussions on the spin densities that are related with ESR signals (Figure S59).

Table S4 Spin densities, excluding hydrogen atoms, of triplet-state **1c**$^{2-}$ estimated at UBLYP/6-31G(d)//CAM-UB3LYP/6-31+G(d,p). Atom labels were referred to Figure S50.

atom labels		spin densities
1	C	0.001804
2	C	-0.00059
3	C	-0.0005
4	C	0.0016
6	C	0.001422
8	C	-0.00073
10	O	-0.00004
11	O	0.004279
12	C	0.032662
13	C	0.033051
14	C	0.074455
16	B	-0.00288
17	O	0.036273
18	O	0.036051
19	C	0.027006
20	C	0.077181
21	C	0.07758
22	C	0.027059
23	N	0.033181
24	C	0.005139
25	C	0.005167
26	C	0.145228
27	N	0.033599
28	C	0.145616
30	C	0.013581
31	C	0.009105
32	C	0.020881
34	C	0.016359
38	C	0.009176
39	C	0.013443
40	C	0.01645
42	C	0.02075
46	C	0.151754
47	C	-0.00917
48	C	-0.00619
49	C	0.082345
51	C	0.111204
53	C	0.037614
54	O	0.170932
55	C	-0.00537
56	C	0.003568
57	C	0.000006
58	C	0.004167
68	C	-0.00728
69	C	0.004979
70	C	0.004732
71	C	-0.000032
81	C	0.151954
82	C	-0.00926
83	C	-0.00618
84	C	0.082201
86	C	0.11131
88	C	0.037652
89	O	0.170913
90	C	-0.00536
91	C	0.00358
92	C	0.000006
93	C	0.004149
103	C	-0.00729
104	C	0.004978
105	C	0.004743
106	C	-0.000033

Cartesian Coordination of Optimized Structures

1a-1
(estimated at CAM-B3LYP/6-31+G(d,p))
–2795.7395498 hartree
C,-0.3649247996,8.594378538,0.5007960001
C,-0.471146695,8.1856139029,1.8254589536
C,-0.2227035533,7.6590895641,-0.536878975
C,-0.4396305736,6.8244556947,2.1686342601
H,-0.5790819665,8.9279705312,2.6098388554
C,-0.1929158479,6.3254502742,-0.1904909053
H,-0.1378800516,7.9650903979,-1.5739204986
C,-0.2998143433,5.913420011,1.143396402
H,-0.5203088863,6.4932744039,3.1982668087
O,-0.0648217212,5.2441478221,-1.0054116501
O,-0.2428315889,4.5578617816,1.2177254366
C,1.1749784066,1.9841789111,-0.1518106261
C,-1.210455763,1.9023879379,-0.3589861812
C,-0.0050370738,1.2448842404,-0.1256513856
H,0.0190861129,0.178592819,0.0191431059
B,-0.0995257583,4.0813582229,-0.1512104361
O,-1.2491632732,3.2016715132,-0.5172496189
O,1.1539065556,3.2852547008,-0.297688764
C,-4.3150594003,-0.05428219,-0.6316922882
C,-4.7284061124,1.2642704615,-0.7809863539
C,4.7442112392,1.5730893455,0.0983064579
C,4.3959459925,0.2284691053,0.137472861
N,3.5797617064,2.2722363116,-0.0203957824
C,2.9985626605,0.1299801375,0.0451882281
C,-2.4836224765,1.2550291084,-0.477276709
N,-3.6087810022,2.0348235151,-0.6762119643
C,2.4894057359,1.4210503196,-0.0506371637
H,-0.3908239535,9.6526032238,0.261159049
C,6.0465253682,2.2457546332,0.1504275642
C,6.2893498579,3.3956117383,-0.6018790657
C,7.0643848187,1.7535024653,0.96252823
C,7.5077760573,4.06612687,-0.5663696841
H,5.5038132564,3.7587199839,-1.2515178449
C,8.3136156767,2.3669591728,1.0435305064
H,6.8543491388,0.8798830636,1.5631767837
C,8.5103386655,3.5278825408,0.2669588081
O,9.7019347486,4.2027481061,0.2823622157
C,7.7425800856,5.3329425625,-1.4108704383
C,8.8937949505,5.1052572577,-2.4108680365
C,6.4988198441,5.7085442946,-2.2313005178
C,8.0609715485,6.5328117004,-0.4962811572
H,9.83407044,4.8859653253,-1.9063055028
H,8.6596461299,4.2745275678,-3.0844453328
H,9.0333314086,6.0030291241,-3.0221314497
H,5.6341580352,5.9281890046,-1.5980034741
H,6.7159386483,6.6113914791,-2.8087378689
H,6.2193267633,4.9258138738,-2.9429814716
H,8.1997513321,7.4326344684,-1.1046462106
H,7.2328868625,6.7206526699,0.1945446048
H,8.9669939482,6.3711836857,0.086609145
C,9.4114188477,1.7904125658,1.9636093185
C,10.6499024914,1.3622507026,1.1436123188
C,9.7968863274,2.8034472798,3.0655633116
C,8.934737143,0.5269602464,2.6988612076
H,10.3727743957,0.5933283311,0.4171046397
H,11.1263221408,2.1673026667,0.5777453722
H,11.4102313243,0.942086816,1.8092119632
H,8.9242424052,3.0404082382,3.6802998699
H,10.5639032993,2.3723538191,3.7163570111
H,10.185511148,3.756282607,2.6963167893
H,9.7451430089,0.155543619,3.3323138236
H,8.0768697688,0.7274148311,3.3461639479
H,8.6670109463,-0.2743061856,2.0045603955
C,-6.0547253462,1.8557803838,-0.9851026828
C,-6.3841180309,3.0816016572,-0.411470753
C,-7.0114650851,1.2061384984,-1.7647017477
C,-7.6299056262,3.6797739247,-0.59459794
H,-5.6501990474,3.5664400523,0.2182413249
C,-8.2780011133,1.7373879393,-1.9852140219
H,-6.7384055725,0.2683709863,-2.2279318701
C,-8.5639833158,2.98332915,-1.3888665372
O,-9.8156233816,3.4868650812,-1.6244158544
C,-7.9515245517,5.0384658046,0.0650292923
C,-9.1260510051,4.9032028474,1.0604881281
C,-6.7630724708,5.5743495581,0.8802536858
C,-8.2503912165,6.1132911712,-1.0052169184
H,-10.0617202661,4.5505604707,0.618500699
H,-8.86769589,4.1949857188,1.852582921
H,-9.3319741878,5.8718230266,1.526219541
H,-5.8802045097,5.7461068869,0.2588623998
H,-7.0396645186,6.5339800363,1.3254185109
H,-6.4879506658,4.9020104842,1.6972274903
H,-8.4547711142,7.0728704349,-0.5207313967
H,-7.3851354604,6.2429004147,-1.6609771643
H,-9.1030532634,5.8936791245,-1.6541100799
C,-9.3102203568,0.9959888016,-2.8556230688
C,-10.5670264443,0.6618826902,-2.0272714596
C,-9.6920879562,1.8501139011,-4.081021827
C,-8.7545970421,-0.3340764352,-3.3881112903
H,-10.3077529838,0.0275800074,-1.1733753805
H,-11.0572927827,1.5596327602,-1.6527715208
H,-11.2829274771,0.1128470951,-2.6480392796
H,-8.8087306781,2.0639253031,-4.6912649808
H,-10.4064383916,1.3023725469,-4.704739771
H,-10.1473131746,2.7961186242,-3.7904258696
H,-9.5238721597,-0.8219045981,-3.9932797056
H,-7.8781033898,-0.190549464,-4.0271601023
H,-8.4880304656,-1.0233913021,-2.5809502499
C,-2.9240514643,-0.0640437788,-0.4436216442
C,2.5082134889,-1.2913126492,-0.0241002283
C,3.1320684051,-2.045801485,1.1398498882
H,1.4240607118,-1.3951469384,-0.0559309286
C,3.1609225465,-1.8895704223,-1.316423338
C,4.4597732814,-1.9524004038,1.2189257386
C,4.7039878501,-1.7813994094,-1.220522918
H,2.784339429,-1.3492919662,-2.1891833845
H,2.8500054169,-2.9330487035,-1.4155243044
C,5.0992569799,-1.1024302241,0.1332628173
H,5.1044732475,-1.1871199595,-2.0460367644
H,5.1653459569,-2.771349536,-1.2726461075
H,6.1830120855,-1.0290860663,0.229670075
C,-2.3599052304,-1.4484249786,-0.2717948231
C,-3.145774581,-2.1181102614,0.8461416995
C,-2.7398751169,-2.22005721,-1.5805774759
C,-4.46824899,-2.1041578966,0.6809472184

C,-4.2800178447,-2.219019819,-1.7638924086
H,-2.2454162553,-1.7446278909,-2.4319862243
H,-2.3593909431,-3.2423707653,-1.5078407668
C,-4.9428310964,-1.4213627444,-0.5925223163
H,-4.5556443089,-1.7565315975,-2.7157227923
H,-4.6700224729,-3.240363217,-1.7690984508
H,-6.0295451709,-1.4099154664,-0.6818843307
H,-2.6411531258,-2.5889895436,1.683240881
H,-5.1697060527,-2.5660313034,1.3679490489
H,5.0576398014,-2.4602563907,1.9688912623
H,2.5229637155,-2.6350103437,1.8173799536
H,-1.28400977,-1.4804864668,-0.1020238965
H,-3.554743385,3.0350325774,-0.8019947721
H,3.4796454761,3.2764011164,0.0176986397
H,-9.9155153021,4.3471806851,-1.2094056346
H,10.3245230395,3.7750123761,0.8750765283

1a-2

–2795.7343648 hartree

C,0.0263641859,8.8564286275,0.3311154915
C,-0.1045490235,8.4900522449,1.6658862459
C,0.11365135,7.8871783305,-0.6810639239
C,-0.1538028803,7.1391145829,2.0451044924
H,-0.17030883,9.2581321461,2.4298854911
C,0.0644238394,6.5639051638,-0.2992779093
H,0.2154931146,8.1599348435,-1.7258122445
C,-0.0674023911,6.1946272326,1.0446949766
H,-0.2560982988,6.8408614745,3.0828331352
O,0.1287991702,5.4554440233,-1.0852035433
O,-0.0907500985,4.840134407,1.1550371806
C,1.2205259223,2.1748265664,-0.122412735
C,-1.1700550966,2.1940846955,-0.3434705416
C,0.0074430626,1.4921280349,-0.0742528513
H,-0.00057402,0.4273177359,0.0950818791
B,0.0327018749,4.320985651,-0.19964451
O,-1.1479617218,3.4820010013,-0.5479849646
O,1.2572046913,3.4673936042,-0.3133967116
C,-4.6557289323,1.1140538803,-0.7160960612
C,-4.0148593512,-0.0733265456,-0.4053565348
C,4.7692604371,1.6200069876,0.1457357474
C,4.3650729892,0.2935875779,0.2325246149
N,3.6347800093,2.3620872521,0.001261202
C,2.9644821124,0.2504391685,0.1426510621
C,-2.4604609453,1.5806480683,-0.4478685425
N,-2.6822810642,0.2193345114,-0.2668718564
C,2.5098669059,1.5570900556,-0.0000527103
H,0.0618107484,9.9076007064,0.0633510328
C,6.0985450342,2.2393791152,0.1754042779
C,6.3893381245,3.3492631441,-0.6187466587
C,7.0944911074,1.7358451355,1.0074464543
C,7.6345349249,3.9694924008,-0.6054845522
H,5.6199172191,3.7205586582,-1.2829053084
C,8.3681789315,2.2993290354,1.0681566337
H,6.8477901713,0.8949780508,1.6400671318
C,8.6133673755,3.4214643297,0.249476556
O,9.8319336304,4.0462347548,0.2422108763
C,7.922325314,5.1931790525,-1.4959675124
C,9.0637608261,4.8806476932,-2.4842040796
C,6.6957954489,5.5892368151,-2.3325544819
C,8.2895776601,6.4120753335,-0.6259193016
H,9.9937883026,4.6413122364,-1.969840003
H,8.7956068361,4.0359891795,-3.1270947466
H,9.2410347379,5.7484723228,-3.1280433235
H,5.8405931919,5.8682496608,-1.7098902046
H,6.9506552184,6.4600787472,-2.942765261
H,6.3844320392,4.7928142693,-3.0152444899
H,8.4659014582,7.2820566359,-1.267044774
H,7.4694579174,6.6597234435,0.0554679765
H,9.1877259505,6.2350923012,-0.0353673871
C,9.4406692485,1.7118318565,2.0108588752
C,10.6602673687,1.2024941252,1.209181109
C,9.8680938846,2.7475307538,3.0756191866
C,8.9113049283,0.4973861014,2.7913072732
H,10.351479304,0.4188886535,0.5116961674
H,11.1689462932,1.9656528324,0.614186228
H,11.4027816566,0.7766748029,1.8910989907
H,9.0057734288,3.0446331138,3.6786805018
H,10.6149917508,2.3082562377,3.7440892723
H,10.2982272893,3.6684068828,2.6727647306
H,9.7053222431,0.115719028,3.4392547176
H,8.062609584,0.7573969711,3.4294829936
H,8.6101327443,-0.3169026491,2.126452137
C,-4.518902574,-1.4412507124,-0.2447461991
C,-3.7784830075,-2.5352884336,-0.6860270646
C,-5.7563892899,-1.6727772911,0.3559991045
C,-4.2296573445,-3.8475030335,-0.5436050016
H,-2.8375194757,-2.3467278185,-1.1857012257
C,-6.2747107968,-2.952228842,0.525419667
H,-6.3124468878,-0.8182032989,0.7154210786
C,-5.4874643199,-4.0295205909,0.066858437
O,-6.0192641355,-5.2786392295,0.2479281007
C,-3.3797961771,-5.0298818588,-1.0574595611
C,-4.1244516,-5.8015746243,-2.1706090121
C,-2.0583783429,-4.5551614932,-1.6841437733
C,-2.984372467,-5.9712509651,0.1031643513
H,-5.0848386704,-6.2272448185,-1.8681298679
H,-4.3298315745,-5.1381481985,-3.0151436933
H,-3.5037378922,-6.6276792142,-2.5310167339
H,-1.4309046317,-4.0236904752,-0.9631556886
H,-1.4942190191,-5.4258040666,-2.0298260242
H,-2.2225217575,-3.9061682527,-2.5484347743
H,-2.3702310574,-6.7934174229,-0.2768590839
H,-2.39769931,-5.425701889,0.8475344143
H,-3.8258385801,-6.4189699613,0.638634719
C,-7.644251688,-3.1718145699,1.1954796105
C,-8.612622028,-3.8665384838,0.2171729327
C,-7.48650664,-4.0144185649,2.4769611512
C,-8.2951673667,-1.8420281,1.6068463011
H,-8.7546803013,-3.2574971893,-0.6814021687
H,-8.2503217444,-4.8482127606,-0.0856324713
H,-9.5901341647,-3.993350599,0.6941092664
H,-6.8243224975,-3.5114462915,3.189026105
H,-8.4622355032,-4.1399379083,2.9578783535
H,-7.0799014813,-5.0028733413,2.2664764405
H,-9.2624863078,-2.0508861275,2.0721177799
H,-7.692583217,-1.2942620282,2.3375654052
H,-8.4799869266,-1.1887737208,0.7486592645
C,-3.6945281029,2.1386082049,-0.7437115024
C,2.4164882962,-1.1514521176,0.1240980654
C,3.0090660403,-1.88905663,1.3150057651

H,1.3286607383,-1.2115908247,0.0953877358
C,3.0423712773,-1.8219881728,-1.1457254104
C,4.3395777591,-1.8478867481,1.3913954336
C,4.5887414351,-1.7747400464,-1.0524065523
H,2.6882108194,-1.2976851476,-2.0374204802
H,2.6888665242,-2.8548379471,-1.2081885068
C,5.0126244046,-1.0645750358,0.2764049997
H,5.0128181652,-1.2274365286,-1.8982898268
H,5.0084549465,-2.7842784958,-1.0688662421
H,6.0985236403,-1.0326811384,0.3708712421
C,-4.2544983779,3.488470157,-1.0871585622
C,-5.0367314048,3.3239254541,-2.3818737417
C,-5.325523022,3.7796641228,0.0175821132
C,-5.950260753,2.3526505446,-2.3676602458
C,-6.3961340437,2.6565662694,0.0288737293
H,-4.825178872,3.8547698126,0.9864444529
H,-5.7834728924,4.7501079906,-0.1892361274
C,-6.0433303848,1.5855863828,-1.0566941337
H,-6.4416977038,2.174620182,1.0099313947
H,-7.388528064,3.0636203007,-0.1826619763
H,-6.785609478,0.7867859214,-1.0877815655
H,-4.8644594987,3.9849601654,-3.2247539728
H,-6.6138575973,2.1304838999,-3.1970216483
H,4.9164148472,-2.351776183,2.1602654697
H,2.3769620796,-2.4265745831,2.0141886672
H,-3.4993598755,4.2699697172,-1.1321595437
H,-2.0101326952,-0.4323871554,0.1033243717
H,3.57628523,3.3703755033,0.0018973982
H,-5.4237581191,-5.9518851225,-0.0897304129
H,10.4345479556,3.6182950205,0.8550832668

1a-3

–2795.7304085 hartree

C,-2.5287516424,-4.254193166,3.6968380622
C,-1.4515699442,-5.1250009106,3.5772539285
C,-2.5549419256,-3.0342674973,3.0020653157
C,-0.3550088655,-4.8128128992,2.7578146155
H,-1.4549858266,-6.0622737711,4.124513076
C,-1.474398466,-2.7364496619,2.2001902272
H,-3.3891375666,-2.3463997941,3.086858693
C,-0.3894352734,-3.6131406895,2.0795831654
H,0.4908697362,-5.4839989672,2.6560268118
O,-1.2696316699,-1.6295742749,1.4367879319
O,0.5395553722,-3.0907355776,1.236203211
C,1.8985991209,-0.3160430909,0.3807430967
C,0.8298822537,-1.417418372,-1.4710236445
C,1.9195433902,-0.7067973667,-0.9597272313
H,2.7388027807,-0.4123128114,-1.5992116074
B,0.00957145,-1.8068407636,0.7962936073
O,-0.1438347959,-1.7900006015,-0.6890807865
O,0.9444438365,-0.7057430255,1.1770897104
C,-0.0474979917,-2.4921019146,-4.8667341588
C,1.2080551065,-1.9394645191,-5.0526118916
C,4.6534650663,1.9602833593,1.0686327339
C,4.2144029363,1.7301057053,2.3602262908
N,3.8516877458,1.2175911737,0.2357203674
C,3.1103567666,0.8592710554,2.301044368
C,0.6735566223,-1.7604740954,-2.853213459
N,1.6230324079,-1.4749705235,-3.8298722943
C,2.8790350851,0.5475111724,0.9724706837
H,-3.3647728576,-4.5182361878,4.3365934434
C,5.7339740258,2.7956037815,0.535054116
C,6.4712215499,2.3918609152,-0.5788422173
C,6.0392784667,4.018519124,1.1267697716
C,7.4928213896,3.1674749704,-1.1186237864
H,6.2527112752,1.4246215797,-1.0121384896
C,7.0552938203,4.8442535764,0.6484250985
H,5.4429582767,4.3325986744,1.9715978425
C,7.7696943334,4.3947813721,-0.4818096472
O,8.782624516,5.1375356998,-1.0281779479
C,8.2906467391,2.6898921025,-2.3467260421
C,9.7811268149,2.5258639188,-1.9881851568
C,7.7958351992,1.3255795356,-2.8519172679
C,8.1303622623,3.687948057,-3.5110312009
H,10.2270316535,3.4669771728,-1.668818806
H,9.9050968545,1.7934138501,-1.1842159713
H,10.3336634423,2.1628359235,-2.8611992178
H,6.7481481724,1.3586659839,-3.1678511929
H,8.3903938252,1.0336262185,-3.7220989977
H,7.9085283534,0.5385152023,-2.1004302632
H,8.681077975,3.3259200535,-4.3857153129
H,7.0766870788,3.7843571687,-3.7930635497
H,8.5090234427,4.676381212,-3.2541077661
C,7.3554414043,6.1945048404,1.3347298231
C,8.7944797593,6.2182054395,1.8989350015
C,7.1197676742,7.3725921539,0.362501154
C,6.427400926,6.4429942249,2.5351882548
H,8.9242248064,5.4193957928,2.6342715545
H,9.5837766172,6.085896937,1.1536724221
H,8.9822119715,7.1733194519,2.3988051858
H,6.077791628,7.383823302,0.0310686931
H,7.3240897306,8.3204836757,0.8697113439
H,7.734976362,7.3499261263,-0.5408651648
H,6.6794796722,7.4060857151,2.9876290766
H,5.3756250604,6.4839132398,2.2399842613
H,6.5421121165,5.6774947403,3.3074283324
C,2.0440821576,-1.7931085201,-6.2484502618
C,2.8367829079,-0.6632764603,-6.4360379087
C,2.0635937828,-2.7897995516,-7.2243041116
C,3.6488010244,-0.499535571,-7.5582485298
H,2.7878845837,0.1233859686,-5.6948489452
C,2.840097298,-2.6920464067,-8.3739565984
H,1.4633304038,-3.6732835999,-7.057909805
C,3.6302662495,-1.5320749247,-8.5181580641
O,4.3877333144,-1.4619651619,-9.6569581547
C,4.5070388494,0.7729232389,-7.7274506728
C,4.0766861709,1.5660926764,-8.9823240326
C,4.3417641992,1.7369343544,-6.5410564706
C,6.0113743926,0.4209797532,-7.7804068073
H,4.1619492381,1.0154200777,-9.9228411372
H,3.0304660116,1.8713399456,-8.8938279289
H,4.6868310348,2.4692868197,-9.0800172061
H,4.6588226347,1.2825913834,-5.5980466654
H,4.9684786823,2.6175873695,-6.7064189362
H,3.3104526441,2.0832318494,-6.4327981379
H,6.6041095189,1.3349772584,-7.8840951339
H,6.3146114059,-0.0765013376,-6.8549867521
H,6.2990026294,-0.2440200594,-8.5990876908
C,2.8383238962,-3.8126752986,-9.4308722695
C,2.3434817154,-3.2685989505,-10.7860798513

C,4.251057043,-4.411469468,-9.5812115213
C,1.8995365061,-4.9636182809,-9.0368180378
H,1.3276916457,-2.8708910627,-10.6935590806
H,2.9893259842,-2.4788463414,-11.1678914553
H,2.3203216831,-4.0790611602,-11.5220605262
H,4.5956400132,-4.8281669513,-8.6293134539
H,4.2307134868,-5.2240839749,-10.3149042096
H,4.9745172774,-3.6684453405,-9.9146988398
H,1.9301690392,-5.7281773617,-9.8180302774
H,2.2005185595,-5.4417308337,-8.0998470397
H,0.859947432,-4.635540877,-8.9418001034
C,-0.3791154794,-2.3826937058,-3.5052232456
C,2.5937394477,0.437348876,3.6458861394
C,2.3680351659,1.7083370821,4.449311298
H,1.7151442079,-0.2015671468,3.5892766848
C,3.7975069942,-0.2891803557,4.335507443
C,3.4176571658,2.5290411313,4.5150625636
C,5.0181425368,0.6640527322,4.4085355423
H,4.0379202289,-1.1932621101,3.7699780469
H,3.488070399,-0.6027736291,5.3356660846
C,4.6494485814,2.0439346709,3.767007559
H,5.8755068621,0.2418660058,3.8767999516
H,5.3226898521,0.8261761287,5.4460972273
H,5.4885254323,2.7385224526,3.8263044801
C,-1.7345038616,-2.92949263,-3.1630690065
C,-2.7272626317,-2.2694480763,-4.1085491883
C,-1.6854795633,-4.4358056922,-3.5882786862
C,-2.4212616874,-2.3662024641,-5.402810616
C,-1.3403911076,-4.5510161777,-5.0966932937
H,-0.9438565737,-4.9540898561,-2.9751074886
H,-2.6574027428,-4.8884539799,-3.3769541149
C,-1.1330577113,-3.1207439909,-5.6977252809
H,-0.4293983138,-5.1390970547,-5.2418180896
H,-2.1443998084,-5.0524410249,-5.6420243997
H,-0.9046950912,-3.1630596762,-6.7635343091
H,-3.6253719246,-1.7930873758,-3.7296360652
H,-3.0427142521,-1.9807788091,-6.2045516958
H,3.4265813267,3.4564756733,5.0787865283
H,1.4221990833,1.8908748087,4.9483722493
H,-1.9927321245,-2.8158890812,-2.112591368
H,2.5715307126,-1.1916357538,-3.6455699457
H,3.7883997217,1.3942610587,-0.7546068152
H,4.8925550455,-0.6454630456,-9.6776079191
H,8.922402967,5.9413736182,-0.5216570922

1a·Cl⁻

–3256.0786166 hartree

C,-0.2193321178,9.1373176644,0.2632971817
C,-0.3050024272,8.7947398155,1.6074338359
C,-0.1320796811,8.1476593006,-0.7310302055
C,-0.3070207981,7.4490898379,2.0128530796
H,-0.3716946087,9.5757702206,2.3589537943
C,-0.1345428318,6.8295879827,-0.3258954343
H,-0.0648373403,8.4010414714,-1.78377735
C,-0.2210700931,6.4829469831,1.0318631881
H,-0.3736435112,7.1686038515,3.0586987961
O,-0.0606699874,5.7158633374,-1.0897119837
O,-0.2049323964,5.1397792426,1.1722164226
C,1.1358314788,2.4810330962,-0.1031122248
C,-1.2490425663,2.4315125016,-0.2741452975
C,-0.0551492384,1.776020807,-0.0020942662
H,-0.04442379,0.7106917733,0.1770867512
B,-0.1000775782,4.5760805006,-0.183001136
O,-1.2787630234,3.7269573091,-0.4811587969
O,1.1420135916,3.7765468053,-0.3134536836
C,-4.6636681036,1.1436722619,-0.5932831086
C,-3.9146091717,-0.0170713937,-0.4247446644
C,3.9000688607,0.1515645073,0.1454703692
C,4.6139607991,1.3443393789,0.0804478765
N,2.5747726025,0.4698805283,0.0825549531
C,3.6932762716,2.3936874255,-0.029813619
C,-2.499820459,1.7410114628,-0.3845633235
N,-2.6090512992,0.359919525,-0.3047124478
C,2.4184383926,1.8445342145,-0.0253524121
H,-0.219626138,10.1836664535,-0.0271341329
C,4.36819129,-1.235514858,0.2634839836
C,3.6455463114,-2.189119766,0.9812910442
C,5.5551548067,-1.6251359958,-0.3511770142
C,4.0731415609,-3.5080990234,1.1036696603
H,2.7143704777,-1.8885977887,1.4412722611
C,6.0450624383,-2.9284583353,-0.2702968873
H,6.0880252787,-0.8845741669,-0.930232297
C,5.2781256253,-3.8564699265,0.4622190858
O,5.6732481139,-5.1699040486,0.5835932504
C,3.2443846378,-4.5350697085,1.8992338612
C,4.0826393534,-5.1396539018,3.0437184826
C,2.0034867038,-3.8907776203,2.5367565274
C,2.7468059638,-5.6492694399,0.9567816817
H,4.9557718062,-5.6781792105,2.6762618012
H,4.4223604895,-4.3523788966,3.7251348787
H,3.4641521468,-5.8361730334,3.6208049055
H,1.3262701586,-3.4528280485,1.7984268233
H,1.4505897151,-4.6619276934,3.0830528134
H,2.2750883029,-3.112333513,3.2573560537
H,2.1471208739,-6.3705789923,1.5234524224
H,2.1110842764,-5.2251351606,0.1739405369
H,3.5737768114,-6.1835495888,0.4875868028
C,7.3630736043,-3.313854895,-0.9763104344
C,8.4142645421,-3.8046991487,0.0451490632
C,7.1173789221,-4.3756138381,-2.07294024
C,8.0013301026,-2.1123752268,-1.6930647631
H,8.6392900295,-3.0082574992,0.7601090549
H,8.1037643827,-4.6721331309,0.633044968
H,9.3429466266,-4.0718772144,-0.4703978981
H,6.4209033569,-3.9861141818,-2.8203953626
H,8.0588040405,-4.6196510098,-2.5764872131
H,6.6908596352,-5.3166563762,-1.7139833859
H,8.9343135172,-2.4326792129,-2.1670229243
H,7.352969666,-1.7103039697,-2.4758495321
H,8.2435261948,-1.3043706904,-0.9975253539
C,-4.3257008982,-1.4265184415,-0.3847405782
C,-3.5107341719,-2.4294387246,-0.9048133447
C,-5.5458793745,-1.7884363466,0.1860018975
C,-3.8733954635,-3.7747444572,-0.8652248086
H,-2.5612753217,-2.1478984502,-1.3381063741
C,-5.9676082946,-3.1126531835,0.2688224267
H,-6.1607759532,-1.0047110003,0.6059000743
C,-5.1018511938,-4.093025883,-0.2532329768
O,-5.520668885,-5.3992683596,-0.1337075721
C,-2.9422247341,-4.8498692246,-1.4671211628

C,-3.6691139577,-5.6667181227,-2.560064477
C,-1.7150520233,-4.222996007,-2.1495136464
C,-2.3893922637,-5.7711332069,-0.35624174
H,-4.5563026983,-6.2064219349,-2.2191520627
H,-3.9939719887,-5.0026941134,-3.366423641
H,-2.9800175619,-6.4027896921,-2.9870060489
H,-1.0955891567,-3.6455922963,-1.4585165452
H,-1.0958674956,-5.0243124416,-2.5657149373
H,-2.0070963247,-3.5697026977,-2.9773555184
H,-1.7232378372,-6.5226535533,-0.7923383196
H,-1.812784186,-5.1808549242,0.3604001215
H,-3.1514650296,-6.3118696727,0.2167377116
C,-7.3113794992,-3.4809435224,0.9253553291
C,-8.2188983757,-4.2122937607,-0.0839350028
C,-7.079644395,-4.3664458405,2.1658763188
C,-8.0768564208,-2.2348787601,1.3973722046
H,-8.4198231918,-3.5736817261,-0.9504415373
H,-7.7653337012,-5.1378086447,-0.4374790155
H,-9.178857864,-4.4545746227,0.386320313
H,-6.46192543,-3.8408632898,2.9010537644
H,-8.039687046,-4.6037317823,2.6386089732
H,-6.5832609925,-5.300666659,1.9052892859
H,-9.0262851792,-2.5485159057,1.8423499377
H,-7.5254501383,-1.6755367353,2.158814996
H,-8.3081908599,-1.5561767228,0.5707453096
C,-3.7846976909,2.2329351786,-0.5712446507
C,4.3149280761,3.7612773419,-0.0122436024
C,5.4243365068,3.764952122,-1.0517269069
H,3.5929614614,4.5646389849,-0.1448840797
C,5.0416219906,3.8581045844,1.3715929243
C,6.3089470978,2.7727258811,-0.9353972583
C,6.0656721645,2.701198477,1.5086124196
H,4.2922174762,3.8147581656,2.1662155672
H,5.5401818369,4.8292235073,1.4428494253
C,6.0384613199,1.8167482383,0.2162438863
H,5.8295089439,2.0722416335,2.3716280343
H,7.0772912964,3.0936215095,1.6528459597
H,6.7709805951,1.0108578816,0.2833585712
C,-4.4572705983,3.5667010191,-0.730058303
C,-5.3132930498,3.4838525575,-1.9846309801
C,-5.4697421481,3.6619831542,0.4605255123
C,-6.1535257895,2.4485448383,-2.021801233
C,-6.456985035,2.4650171395,0.4133987805
H,-4.9096315683,3.6702306139,1.3992260106
H,-6.0086902572,4.6113606171,0.3915960394
C,-6.101355654,1.5423528479,-0.8002986838
H,-6.4005384356,1.8802697189,1.3364326756
H,-7.488202462,2.8162742386,0.3084624112
H,-6.7900088821,0.6994391054,-0.8758277306
H,-5.2408433955,4.236809718,-2.7630600861
H,-6.8497476251,2.2603560183,-2.8334098758
H,7.1740682785,2.6537804205,-1.5810243864
H,5.4835088985,4.545174257,-1.8040707283
H,-3.7606436759,4.4027706337,-0.7349210487
H,-1.8291654125,-0.2928987661,-0.1599040929
H,1.8174126249,-0.2228078714,0.0180527094
H,-4.8012966266,-5.9908698101,-0.3691605519
H,6.4630880565,-5.3259446542,0.0620952909
Cl,0.026631071,-1.6686448101,0.0428848172

1b-1

–2638.6381421 hartree
C,-0.8681605816,-3.840877149,2.8130354022
C,0.27624151,-4.6278138778,2.7492602531
C,-0.9863964944,-2.6710709047,2.0451682099
C,1.3505599582,-4.2783213011,1.9154130715
H,0.3441491627,-5.5273821884,3.3527177205
C,0.0729159116,-2.335958955,1.2293992097
H,-1.8732274771,-2.0479887484,2.0867795285
C,1.2260143219,-3.1281873914,1.1651404806
H,2.2484006488,-4.8840370327,1.8574000838
O,0.193899071,-1.2648317496,0.4009423537
O,2.1159687787,-2.5856577579,0.2946971181
C,3.2560398021,0.2552936597,-0.7294954839
C,2.2776359928,-1.0040760205,-2.5214843032
C,3.2938844614,-0.1715136765,-2.0555388579
H,4.0738911013,0.1616630176,-2.7168046536
B,1.4916933398,-1.3734901542,-0.2258229488
O,1.3480648858,-1.4548888486,-1.7062583107
O,2.3238459749,-0.1796315613,0.0916565665
C,1.0708845924,-2.6796204789,-5.4493185018
C,4.9062668664,2.3662613871,1.6493294501
N,4.0139194237,1.4673093707,1.2021612083
C,5.2446051101,1.9707993346,-0.5896647439
C,2.1187641275,-1.4294897806,-3.8714376985
N,1.1206741365,-2.337484863,-4.1512033985
C,4.159688365,1.1850224502,-0.1387080821
H,-1.6848019031,-4.1316884973,3.4661106521
C,4.938324731,2.8042424391,3.0456458673
C,4.6806269451,1.9018255372,4.0795890678
C,5.206567956,4.1322859155,3.3686179799
C,4.679455358,2.2824811171,5.416773874
H,4.5028712599,0.8650577768,3.8251264015
C,5.2271217614,4.5853121727,4.6864308612
H,5.3720181914,4.8292597526,2.5596221564
C,4.9592680651,3.6370914335,5.6962049451
O,4.956651189,3.9868669753,7.0183937376
C,4.3957217471,1.2600690542,6.5335640043
C,3.1433298062,1.6754984082,7.3313753607
C,4.1250810495,-0.1418439086,5.9657021298
C,5.6120560152,1.1423082743,7.4736706978
H,3.2711625714,2.6423679287,7.8167331518
H,2.2688669835,1.7293151142,6.6751106146
H,2.935561154,0.9281754855,8.1040950405
H,4.981835439,-0.5385252401,5.4126717912
H,3.9283294573,-0.8268751449,6.794798951
H,3.2477374758,-0.164381556,5.3124770201
H,5.4079839598,0.3937732082,8.2462523834
H,6.4977847339,0.817232707,6.9184162167
H,5.8402554051,2.0871290626,7.9656197662
C,5.5176491479,6.0691118257,4.9998355537
C,4.3093017975,6.7323439491,5.6991421889
C,6.8004502091,6.2120728184,5.850482073
C,5.766311556,6.8845443403,3.7202417808
H,3.4286534348,6.6861417239,5.0527038495
H,4.0225556724,6.2751119431,6.6498406534
H,4.5277799364,7.7856442755,5.899982634
H,7.6580071227,5.8001082892,5.311374998
H,6.9998863179,7.2697996725,6.0476459558
H,6.7692454048,5.7049908225,6.8186960526

H,5.9693602455,7.9237291162,3.9934175797
H,6.6314717254,6.5177712774,3.161078147
H,4.8976022545,6.883408335,3.0565652672
C,0.0855493074,-3.6306016615,-5.965733704
C,-1.2111739199,-3.6525833505,-5.4552250735
C,0.4283457089,-4.5451695278,-6.9625128697
C,-2.1716139569,-4.5574889873,-5.9022446343
H,-1.4800755585,-2.9201647671,-4.7055877282
C,-0.4788554675,-5.4733308935,-7.461583064
H,1.444979491,-4.5430807282,-7.3291220787
C,-1.7786543694,-5.4605128192,-6.9123562606
O,-2.651250298,-6.3828280706,-7.4205835407
C,-3.5985038339,-4.5422306158,-5.31215552
C,-3.920650923,-5.8843508176,-4.6157999147
C,-3.7592710122,-3.4583282753,-4.2335265861
C,-4.6422864689,-4.2168293142,-6.4045034213
H,-3.8838464231,-6.7626871291,-5.2665194217
H,-3.2117210153,-6.0681123074,-3.8039963894
H,-4.9258453383,-5.8467723087,-4.1853402636
H,-3.5892229823,-2.4540616687,-4.6309132761
H,-4.7820114797,-3.4883158617,-3.8480883585
H,-3.087069021,-3.6165817817,-3.3859893204
H,-5.6449525013,-4.1992184556,-5.9666180176
H,-4.443049688,-3.2309475155,-6.8335303568
H,-4.6656334303,-4.9223217999,-7.2392481935
C,-0.0672040986,-6.4765170802,-8.555668648
C,-0.205717375,-7.9221248184,-8.0379695222
C,-0.9318846674,-6.2769355177,-9.8165919464
C,1.3979538874,-6.2865137917,-8.9780687914
H,0.4298745102,-8.0794686935,-7.1607216164
H,-1.2334187796,-8.1602669453,-7.7664304683
H,0.1152834866,-8.623900139,-8.8148448184
H,-0.8129795113,-5.2616078958,-10.2085714847
H,-0.6130579139,-6.9766765321,-10.5961778036
H,-1.9890320755,-6.4472064789,-9.6164581349
H,1.6390020219,-7.016314762,-9.7559141253
H,1.5828907428,-5.291984576,-9.3956715854
H,2.0933679045,-6.4506528081,-8.1495377392
C,2.7564371134,-1.1555924794,-5.1028073156
H,0.5436777775,-2.7215353596,-3.4133022078
H,3.2649879851,1.053531108,1.7437706158
H,-3.4986165745,-6.3293676566,-6.971411556
H,5.1626424504,4.9190631611,7.122369758
C,5.7170910543,2.7112190188,0.5410793339
C,5.8912829781,2.1318200995,-1.8380288028
C,6.8409885717,3.5603524831,0.4264759201
H,5.5556277968,1.5986140709,-2.718692384
C,7.4502757379,3.6899847696,-0.7952616048
H,7.2218575577,4.0883998941,1.2937649575
C,6.9660613797,2.9810511429,-1.9255598796
H,8.3160549419,4.3345292022,-0.9046477483
H,7.4665162581,3.108990088,-2.8801897459
C,2.093327434,-1.9443782106,-6.0968186473
C,2.4711306048,-1.8553478026,-7.4557726519
C,3.812382691,-0.2985629959,-5.4933847063
C,3.501931094,-1.0196513962,-7.8019332031
H,1.9451950517,-2.4275894551,-8.2119553619
C,4.1716917065,-0.2475524831,-6.817173874
H,4.3299227692,0.3211032409,-4.7716698592
H,3.8070054205,-0.9354925795,-8.8396050182
H,4.980735182,0.4078255806,-7.1239436291

1b-2

–2638.6317122 hartree

C,-1.4293009296,-3.0842455936,4.1201850107
C,-0.3301823435,-3.9350819724,4.0868845591
C,-1.5586586645,-2.0315746524,3.1999551711
C,0.68591001,-3.7692983963,3.1321883864
H,-0.2535630262,-4.7424267738,4.8082354317
C,-0.5563624629,-1.8763907055,2.2667538222
H,-2.4116309464,-1.3618692909,3.2143966947
C,0.550851905,-2.7329604123,2.2329041286
H,1.5470906812,-4.4275940347,3.0950718019
O,-0.4563848302,-0.9423919267,1.2833783136
O,1.3895193605,-2.3703799384,1.2276297824
C,2.6009333127,0.1920792786,-0.2487397227
C,1.4191333847,-1.2471672699,-1.7704713633
C,2.5307954611,-0.4419343729,-1.4851844007
H,3.3080415607,-0.281751708,-2.2151140821
B,0.7775464361,-1.2120225451,0.5864296618
O,0.517870138,-1.4870333325,-0.8548924713
O,1.6899323525,-0.0325188972,0.6721027559
C,1.5159113323,-2.2587609555,-5.2476207917
C,4.5485098012,2.4887080401,1.6969586045
N,3.5704350539,1.6047620386,1.436858246
C,4.6991867022,1.7646378768,-0.479398193
C,1.1657829261,-1.8298580907,-3.0438519185
N,1.9541340991,-1.59626293,-4.155676391
C,3.6031510218,1.1296923617,0.144654871
H,-2.2022476719,-3.2336563148,4.8672983901
C,4.7096812939,3.1092609493,3.0130848402
C,4.4760066562,2.3759281698,4.17810848
C,5.0809627514,4.4467009121,3.1280290267
C,4.5985124978,2.933601538,5.4458279926
H,4.2164532023,1.3294943685,4.0839809023
C,5.2285741055,5.0716441786,4.3649916043
H,5.2264841356,5.0142264169,2.2200571411
C,4.981002319,4.2902415895,5.5132905187
O,5.1010304773,4.8149898675,6.7706027245
C,4.3375437187,2.0947416303,6.7112780188
C,3.1776814534,2.7032917471,7.5248959487
C,3.9379635494,0.6520576362,6.364885475
C,5.612336988,2.0182880921,7.5751395141
H,3.4019042339,3.7150172636,7.8608484728
H,2.2609296164,2.7308284449,6.9273936784
H,2.9831401763,2.0839506734,8.4065395148
H,4.7240038733,0.1232782084,5.8173483349
H,3.7623367929,0.1016809359,7.2931031279
H,3.0139655615,0.6045021636,5.7810718065
H,5.421823447,1.3977271259,8.456859781
H,6.4314062912,1.5583363937,7.0128979123
H,5.9353103098,3.0021720687,7.9134644071
C,5.6317250495,6.5599192629,4.4472648136
C,4.5232790926,7.395783743,5.1268016557
C,6.9823011397,6.7253131926,5.1808989222
C,5.8303898636,7.1753063183,3.0522654691
H,3.5936476138,7.3231055736,4.5557971544
H,4.2845668347,7.0950835923,6.1504043013
H,4.8192858157,8.4487468559,5.1605351679
H,7.7695782526,6.1856626496,4.6472712509

H,7.2596670765,7.7832985976,5.2154570408
H,6.99455583,6.3557189532,6.2098322079
H,6.1167558789,8.224834535,3.1629619671
H,6.6260440416,6.6758867282,2.4926074811
H,4.9147469069,7.1460876613,2.4556921075
C,2.1988485219,-2.1577527228,-6.5385398341
C,3.5905261384,-2.129745429,-6.6073119498
C,1.4682336707,-2.078860707,-7.7250961646
C,4.2740630146,-2.0205526154,-7.8174072632
H,4.1508620048,-2.2338810846,-5.6873416929
C,2.0811794309,-1.9725791393,-8.9685669445
H,0.3894410209,-2.0755016714,-7.6582572193
C,3.4922213351,-1.9443678503,-8.9889576594
O,4.0693255141,-1.8400714084,-10.2245038559
C,5.8177748929,-2.0061840454,-7.8487583994
C,6.3448786758,-0.6789653235,-8.4405983487
C,6.419450222,-2.1077848003,-6.4374768207
C,6.3645883713,-3.2210822933,-8.6324821596
H,6.0257955817,-0.4739203857,-9.4659989798
H,6.0051090358,0.1656514637,-7.8347316807
H,7.4390798703,-0.6795651868,-8.4382370551
H,6.139357493,-3.0379410531,-5.9359698423
H,7.5101310372,-2.0921538827,-6.5124781434
H,6.1239455188,-1.2668695524,-5.803782585
H,7.4586386206,-3.2016698909,-8.6331049694
H,6.042455088,-4.1516120877,-8.15727233
H,6.0422741318,-3.2758351302,-9.6756731708
C,1.2459291808,-1.8796996164,-10.2595216448
C,1.5252765339,-0.5481683864,-10.9848306182
C,1.5633356363,-3.0692138585,-11.1878784403
C,-0.2613799469,-1.9261721351,-9.964675145
H,1.2723578696,0.3009845534,-10.341848995
H,2.5693060781,-0.4572671656,-11.2821752805
H,0.9054864474,-0.4826185209,-11.8850601641
H,1.3374281203,-4.0176301421,-10.6899239109
H,0.9439007083,-3.0081271969,-12.0886649846
H,2.6090199855,-3.080513209,-11.4929115831
H,-0.8082364134,-1.8607144393,-10.9092806404
H,-0.5602777241,-2.8602174005,-9.4793164108
H,-0.5882159607,-1.089797878,-9.3396596653
C,0.1533895197,-2.7176314672,-3.4647652104
H,2.712650514,-0.9321575333,-4.1874114043
H,2.8361388045,1.3253389163,2.0760670842
H,5.0268589278,-1.8285347643,-10.1518176636
H,5.3644921532,5.7374918179,6.7287091313
C,5.2980583131,2.6174336232,0.5035409317
C,5.2611288526,1.7109701148,-1.7776997418
C,6.4619735635,3.359309881,0.1989135074
H,4.8247321046,1.0936855003,-2.5533705035
C,6.9876107309,3.2780861046,-1.0649434033
H,6.9368779741,3.9720061462,0.9573444402
C,6.3771221358,2.4608755243,-2.0524671712
H,7.8815511079,3.8380311346,-1.3184906719
H,6.8113488651,2.4257651268,-3.0467463353
C,0.3769693019,-2.990560513,-4.8496425614
C,-0.4584783911,-3.8978898415,-5.5451736751
C,-0.9263666654,-3.3334723187,-2.7872475144
C,-1.4959367323,-4.4811285035,-4.8664125884
H,-0.272946036,-4.1337387582,-6.5873824971
C,-1.7294417699,-4.1922663135,-3.4930806656
H,-1.1012308071,-3.1222713135,-1.7404011622
H,-2.1497234846,-5.1806630576,-5.3769836517
H,-2.5632108987,-4.6733645637,-2.9916762417

1b-3

–2638.6249154 hartree

C,-2.2379477362,-4.3991096612,3.5776291368
C,-1.1651083175,-5.2659985612,3.4026864497
C,-2.2851594536,-3.1619829272,2.9151477617
C,-0.094268908,-4.9326579497,2.5580917847
H,-1.1518017217,-6.2168587616,3.9258806779
C,-1.2296799127,-2.8434095599,2.0881941941
H,-3.1161152302,-2.476817222,3.0429134736
C,-0.1491105695,-3.7161857688,1.9116986246
H,0.7479785988,-5.6004644951,2.4134439024
O,-1.0482403939,-1.717338116,1.3473164364
O,0.753028823,-3.1725475003,1.0535072225
C,2.1085151502,-0.4006572598,0.2393562192
C,0.9718439871,-1.4401621622,-1.6154649259
C,2.0899031341,-0.7645645241,-1.1112237169
H,2.9104261699,-0.4958307792,-1.7619081633
B,0.2108928022,-1.877251298,0.663596217
O,0.0122996326,-1.8195805723,-0.8149126221
O,1.1543307221,-0.7841118348,1.0440606325
C,1.2380924233,-1.7727550114,-5.2172420418
C,4.953112919,1.7509702934,0.9020430167
N,4.1240693104,1.0268428268,0.1169532901
C,3.3259430836,0.7732782871,2.1829455063
C,0.7657460676,-1.7372243872,-2.9942989654
N,1.6355519235,-1.3506313901,-3.9967969213
C,3.1143383726,0.416911026,0.8359449199
H,-3.0539294796,-4.6797536335,4.235964698
C,6.0850078527,2.494611332,0.3465269713
C,6.8477687559,1.9634288781,-0.695505607
C,6.4136733889,3.756390028,0.8370319085
C,7.9158347382,2.6504628107,-1.2630609626
H,6.6161351078,0.9649215076,-1.0435337008
C,7.4739695363,4.5002997921,0.3228047578
H,5.7998992924,4.1694221335,1.6246021646
C,8.2117933541,3.9236630114,-0.7324326125
O,9.2682803856,4.5782155579,-1.3044178859
C,8.7439226135,2.03152927,-2.4049915542
C,10.2122656501,1.8562491272,-1.9686917996
C,8.2215627997,0.6409482227,-2.7982674953
C,8.6649035255,2.9174869666,-3.6644362563
H,10.6792581986,2.8092059137,-1.7232693155
H,10.2782757943,1.2023110959,-1.0934426886
H,10.7849140902,1.3919176869,-2.77825962
H,7.1889697577,0.6748420585,-3.1605222466
H,8.8384497333,0.2477867809,-3.6109367755
H,8.2778241465,-0.0735949539,-1.9717462008
H,9.2357292396,2.4552275839,-4.4765607698
H,7.6271797661,3.0197805182,-3.9989864697
H,9.0684344343,3.9135664071,-3.4872864131
C,7.7975489614,5.8987693128,0.8920690426
C,9.2154861036,5.9284630808,1.5072910711
C,7.6396356155,6.9866310553,-0.1942899586
C,6.8364436704,6.2886784719,2.0270755035
H,9.2915018709,5.1964388233,2.3159419911
H,10.0268998074,5.7053960403,0.8086744199

H,9.4175883529,6.9187799105,1.9262658434
H,6.6111007715,7.0014651499,-0.5652442977
H,7.8602867968,7.9706730304,0.2303139176
H,8.2855850863,6.8584108574,-1.0668337982
H,7.1061660504,7.2815174288,2.3973258011
H,5.7979480138,6.3358418882,1.6889204656
H,6.895437617,5.5969492373,2.87172993
C,2.0079566487,-1.4758761433,-6.4268695044
C,2.6017476058,-0.2280962046,-6.6073423718
C,2.1692499387,-2.4433082753,-7.4198722084
C,3.353208989,0.0822293203,-7.7401790864
H,2.436349129,0.5315103085,-5.8543487513
C,2.9007145611,-2.2042943893,-8.577919493
H,1.7258238452,-3.4156780041,-7.2581953901
C,3.4873682172,-0.9279499753,-8.715311701
O,4.2013318992,-0.7196476611,-9.8632611511
C,3.9821169734,1.4826317619,-7.9065528766
C,3.4018442056,2.2039310471,-9.1443608658
C,3.6773430755,2.3930537008,-6.7056219417
C,5.522994427,1.3894220382,-7.9880292693
H,3.564033123,1.6862446978,-10.0933813083
H,2.32063859,2.3259151762,-9.0365761293
H,3.8478305825,3.1987005053,-9.2387581367
H,4.0823579439,1.9902010405,-5.7729390925
H,4.1438524258,3.3682273778,-6.8695250756
H,2.6046116493,2.5603703531,-6.5772382877
H,5.9515138393,2.3910377882,-8.0901106404
H,5.9229335176,0.9415457839,-7.0740635775
H,5.9039018281,0.7904458068,-8.8195238479
C,3.0653571838,-3.2966487679,-9.6515084433
C,2.4648400374,-2.8242733342,-10.9908377837
C,4.5547587905,-3.6523400991,-9.8310722003
C,2.335380879,-4.5909685712,-9.2603848006
H,1.3994719538,-2.5992806421,-10.8774265213
H,2.966308431,-1.9354102587,-11.3716952085
H,2.5614546435,-3.6190912313,-11.7376472008
H,4.9793192948,-4.0183844036,-8.8907487913
H,4.6554929988,-4.4484617788,-10.5759519961
H,5.1407512151,-2.796834046,-10.1648785305
H,2.4786647983,-5.3305779009,-10.0528550138
H,2.7257971213,-5.0245288303,-8.3348928036
H,1.2575756953,-4.4407251187,-9.1460223516
C,-0.2706292849,-2.449245668,-3.6328810869
H,2.5071052802,-0.8670672926,-3.8450468813
H,4.1879529535,1.0275076765,-0.8893423637
H,4.5670716154,0.1681384563,-9.8792741608
H,9.424773318,5.4171680425,-0.8636305139
C,4.4869718524,1.6071377255,2.2246397124
C,2.6466303508,0.4442101957,3.381709006
C,4.9815070558,2.0830956614,3.4641463621
H,1.7636750733,-0.1806393944,3.3502176979
C,4.3066205067,1.7493520788,4.6082077132
H,5.8826584605,2.6852027369,3.5060549163
C,3.1385224741,0.9371712822,4.5622582486
H,4.6674793012,2.0997845597,5.5697601358
H,2.6314315018,0.6968649752,5.4912681829
C,0.0276661532,-2.4714325072,-5.0308102889
C,-0.8625899734,-3.0874199506,-5.9441842994
C,-1.4474538198,-3.0724055832,-3.1506456172
C,-1.9942467068,-3.681666094,-5.4518146708
H,-0.6568720756,-3.0783538423,-7.0090338651
C,-2.2785382402,-3.6780127625,-4.0574246543
H,-1.6736473633,-3.058853971,-2.0925515775
H,-2.692480271,-4.1595469244,-6.1312260739
H,-3.1843535859,-4.1624531844,-3.7069638864

1b·Cl⁻

–3098.9815872 hartree
C,-0.2467431526,9.4081936587,0.2841819966
C,-0.3305120501,9.0622312592,1.6276385717
C,-0.158124829,8.4210087078,-0.7124456292
C,-0.329220615,7.7157798953,2.0300731685
H,-0.3982448539,9.8413887666,2.3810415545
C,-0.1573703256,7.1020761282,-0.3101635301
H,-0.0922833087,8.6767904347,-1.7646990806
C,-0.2420792066,6.7519960343,1.0468061713
H,-0.3942029975,7.4326925773,3.0752771554
O,-0.0816315003,5.9900829047,-1.0765835463
O,-0.2228774659,5.4087680588,1.1840582576
C,1.1232820022,2.7578155938,-0.0784417104
C,-1.2655120809,2.7033844902,-0.2462871179
C,-0.0692395895,2.0571473139,0.0422232918
H,-0.0578832968,0.9975462441,0.2457006377
B,-0.1178238615,4.8488566877,-0.1722542143
O,-1.2956926839,3.9976878268,-0.4734211321
O,1.1270212118,4.0523319928,-0.3077934022
C,-3.8771614633,0.2105585485,-0.384225115
C,3.8457179308,0.390412014,0.1393258266
N,2.5482568289,0.7514571195,0.0934023233
C,3.6974526293,2.6689166454,-0.0212293283
C,-2.5061312645,2.0077125036,-0.3574009024
N,-2.6036913336,0.6329036971,-0.2645884655
C,2.3993903059,2.1205750286,-0.0072041215
H,-0.2495683296,10.4552226472,-0.0038536174
C,4.2756945892,-1.0081352205,0.2366947776
C,3.5666651066,-1.9315914043,1.0063348137
C,5.4041958007,-1.4436783009,-0.4540524832
C,3.9498995417,-3.2651117922,1.1062613001
H,2.6822943923,-1.5938643963,1.5280055505
C,5.8464455884,-2.764995532,-0.4003392633
H,5.9249712716,-0.7278156382,-1.0733831812
C,5.0959646626,-3.66050533,0.3879848877
O,5.4488090948,-4.9858117179,0.4919943408
C,3.1362551902,-4.2575298414,1.9589897323
C,4.0222184926,-4.8879175433,3.0522517307
C,1.9620274954,-3.5648789817,2.6682238769
C,2.5392496696,-5.3556624375,1.0561970855
H,4.850412735,-5.4591116447,2.6336872636
H,4.4324128971,-4.1114122833,3.7066258009
H,3.416662799,-5.5597308033,3.6707063675
H,1.2577275546,-3.1078762556,1.9677539791
H,1.4162498769,-4.3123626977,3.2529490719
H,2.3058780023,-2.7936990156,3.3653776379
H,1.9489194928,-6.0509530694,1.6636323323
H,1.87320792,-4.910532562,0.3111009279
H,3.3144678316,-5.9235651356,0.5405858235
C,7.0968889421,-3.2035681808,-1.1930744609
C,8.1983679015,-3.7258106195,-0.242573413
C,6.7359337114,-4.2632784852,-2.2592665425
C,7.7261272506,-2.031173067,-1.9637981462

H,8.5001942277,-2.9344844088,0.4493383724
H,7.900631809,-4.5794605263,0.3718229507
H,9.0790860252,-4.0279903574,-0.8191050961
H,6.0042212423,-3.8533543568,-2.9607082404
H,7.6303840181,-4.5457302857,-2.8243744946
H,6.3011036984,-5.1848696766,-1.8620922196
H,8.610442064,-2.3890700169,-2.4998530019
H,7.0392923226,-1.6101902333,-2.7025329508
H,8.0473368585,-1.2277697013,-1.2955611544
C,-4.2442327892,-1.2079851918,-0.3290164274
C,-3.4220070607,-2.1820199417,-0.8915338716
C,-5.4202268451,-1.6105785793,0.3053651889
C,-3.7318940043,-3.5388281658,-0.8310881693
H,-2.5085881537,-1.866746769,-1.3753399839
C,-5.788007745,-2.9488138138,0.4102493416
H,-6.0377263015,-0.8494366573,0.7608409743
C,-4.9155120868,-3.8987832804,-0.1563695511
O,-5.2808106041,-5.2172255482,-0.0161301008
C,-2.791604299,-4.5812793347,-1.4744432587
C,-3.5376293093,-5.4278989479,-2.5310432197
C,-1.6209024216,-3.9117347498,-2.2134463156
C,-2.1540342765,-5.4766252125,-0.3880564834
H,-4.3871900902,-5.9992982445,-2.1483725695
H,-3.9244709953,-4.7795956912,-3.322688686
H,-2.8424408352,-6.1396906446,-2.9880231216
H,-0.9935438444,-3.3123636096,-1.5488318749
H,-0.9923215916,-4.6909304975,-2.6563993026
H,-1.973829014,-3.271968662,-3.0280946165
H,-1.480954977,-6.2044390703,-0.852716606
H,-1.5680020791,-4.8620791337,0.2999996332
H,-2.8683064578,-6.0431957826,0.2202005175
C,-7.081211714,-3.3636237055,1.1369341425
C,-8.0142608065,-4.1273989355,0.1761151167
C,-6.7528845963,-4.238925265,2.362794058
C,-7.8633567874,-2.1442704492,1.6495293593
H,-8.2824613755,-3.4975838431,-0.678508913
H,-7.5487837522,-5.0376536751,-0.2007949077
H,-8.9392288603,-4.4014869804,0.6961185745
H,-6.115392202,-3.69154325,3.0643397271
H,-7.677749926,-4.5076964461,2.8858470812
H,-6.2399052847,-5.1564300964,2.0763020538
H,-8.77573215,-2.4904649161,2.1448040898
H,-7.2924706999,-1.5640723602,2.3802612255
H,-8.1632022923,-1.4754005154,0.8370604514
C,-3.8145230718,2.4922748578,-0.559396514
H,-1.807955668,-0.0061683115,-0.1123090721
H,1.7725186378,0.0697113047,0.0667829536
H,-4.5571661651,-5.784638288,-0.2944364078
H,6.2057244684,-5.1710950824,-0.067632623
C,4.613752652,1.5775375526,0.0798229384
C,4.1805235343,3.9980670632,-0.0889775358
C,6.0056053835,1.824019995,0.1491161502
H,3.4828004519,4.8216200276,-0.1744416268
C,6.4500723874,3.1206199888,0.0853609507
H,6.7070177587,1.0048732215,0.2675993945
C,5.5364044495,4.2032885949,-0.0415307259
H,7.5142101482,3.3297371738,0.1405399814
H,5.9249197445,5.2160888123,-0.0919620519
C,-4.6831278997,1.3583751484,-0.575658392
C,-6.0686051069,1.5281143059,-0.8066391065
C,-4.342159545,3.7933848484,-0.7392521208
C,-6.5566676783,2.7986351358,-0.9820258676
H,-6.7301380525,0.669876392,-0.8577824731
C,-5.6931747307,3.9277287849,-0.9395727748
H,-3.6808755794,4.6506305966,-0.7237756496
H,-7.6166734544,2.9492014372,-1.1629776125
H,-6.1157037216,4.9182692127,-1.0795775944
Cl,0.0211872389,-1.3678822457,0.0929221643

1c$_{NHNH}$-1 (closed-shell singlet state)

–2637.3619941 hartree

C,-1.3665377394,5.8659388994,-3.6677498629
C,-1.8210359281,6.5555175169,-2.5493236409
C,-0.8059277016,4.5841175202,-3.5502852718
C,-1.7338032601,5.99160069,-1.266387838
H,-2.2505315493,7.5453696217,-2.6657236448
C,-0.723923766,4.0374667194,-2.2867254731
H,-0.4481013122,4.0370479044,-4.4158541384
C,-1.1821185324,4.7328485767,-1.1605425821
H,-2.0846408546,6.5190561449,-0.3859881197
O,-0.2275734608,2.8281623154,-1.9192885253
O,-0.9925003377,3.9881421056,-0.0382488552
C,1.4636109247,1.3421402793,0.2778615771
C,-0.7073543378,0.3728151766,-0.0508552484
C,0.664367016,0.2094907757,0.1332916545
H,1.1008222372,-0.7735525792,0.1623748911
B,-0.3852311763,2.7514095123,-0.4641663078
O,-1.2454284838,1.5922384917,-0.0925008219
O,0.9400323564,2.5644679367,0.19231185
C,-3.800041655,-1.4258419105,-0.3009356439
C,4.8687488261,2.3893430778,0.7044127885
N,3.5045036376,2.5061579328,0.5297203576
C,3.8397371399,0.2976347791,0.7715078326
C,-1.6355839989,-0.6748447572,-0.1560373999
N,-2.9452737388,-0.3427798969,-0.2825410992
C,2.8472379313,1.3193055621,0.5156220672
H,-1.4446735882,6.3227825148,-4.6491380881
C,5.7177467403,3.4818586035,0.690793619
C,5.1857224853,4.822601446,0.6880237809
C,7.1484941158,3.3355262142,0.6501602791
C,5.9604657022,5.9315213439,0.6864987425
H,4.1119111513,4.9472833719,0.7289392431
C,8.0024556506,4.3853875019,0.6325704924
H,7.5484055502,2.3378550461,0.5793255304
C,7.4407546744,5.7630944551,0.6724204644
O,8.1848790898,6.7440332324,0.6880076683
C,5.3710836747,7.3444315182,0.7124225411
C,5.8217804923,8.1223239404,-0.5411785641
C,3.8361715153,7.3220516563,0.7194509819
C,5.8357159496,8.0849027834,1.9832753618
H,6.905464614,8.2285559679,-0.5766117384
H,5.4862745941,7.6177751158,-1.4531572582
H,5.3751068916,9.1217311567,-0.5295202139
H,3.4318082003,6.8214024511,1.6050865426
H,3.4657429945,8.3504360216,0.7328282602
H,3.4229572187,6.842005907,-0.1732860242
H,5.3900627064,9.0846462834,2.0057960868
H,5.5097167078,7.5536043843,2.8836275567
H,6.9198520802,8.1891693457,2.0104152297
C,9.5211690874,4.2043242419,0.5586962073

C,10.0734803335,4.881740598,-0.7118821728
C,10.1848872621,4.8176688593,1.8088709222
C,9.9151318923,2.7213948307,0.5041338099
H,9.6249891402,4.4465183486,-1.611074811
H,9.878466976,5.9535780556,-0.7073350351
H,11.1559455951,4.7269908181,-0.7687544548
H,9.8155784844,4.33641167,2.7206069155
H,11.2677181341,4.6616147316,1.7622954451
H,9.9919322924,5.8876638588,1.8756512302
H,11.0044160235,2.6437512427,0.4508613814
H,9.5945288627,2.1772903482,1.3990765504
H,9.5086501736,2.2171866887,-0.3787134973
C,-5.1747432905,-1.2885355463,-0.3806695379
C,-5.7673154408,-0.0007883884,-0.6472256958
C,-6.0657845274,-2.3993639098,-0.1758938827
C,-7.1016793371,0.199821264,-0.743820619
H,-5.1086571184,0.8398375319,-0.8198822702
C,-7.4135949144,-2.2918524589,-0.2376548891
H,-5.6311106234,-3.3483245449,0.0901239889
C,-8.0104659113,-0.9643066308,-0.5478843549
O,-9.2314351207,-0.8301261857,-0.6354969025
C,-7.7043838512,1.5715451712,-1.0593926214
C,-8.6278057098,2.0151274465,0.0939768187
C,-6.6207759878,2.6456771504,-1.2281499543
C,-8.5062152497,1.5034726859,-2.3753338111
H,-9.4587435332,1.3234798107,0.2284384583
H,-8.0700463308,2.083757827,1.0337922347
H,-9.0341026092,3.0076086626,-0.1261099382
H,-5.9431091902,2.4269116609,-2.0594489489
H,-7.1002198865,3.6033237199,-1.4471325084
H,-6.0262129268,2.7826923896,-0.3192301264
H,-8.9123308597,2.4936405,-2.6059727577
H,-7.8610558097,1.2051981195,-3.2082970706
H,-9.3339684256,0.7988661132,-2.3023135309
C,-8.3422305578,-3.4827425931,0.0156644231
C,-9.2645793074,-3.1884734432,1.2160725658
C,-9.1924531423,-3.7625947492,-1.240678387
C,-7.5539853335,-4.7605950051,0.3368265312
H,-8.6773052151,-3.0037706024,2.1217003634
H,-9.8981037494,-2.3225380933,1.0271873263
H,-9.9082023084,-4.0539865383,1.4047180768
H,-8.5529246404,-3.9899529965,-2.1000537444
H,-9.8351763691,-4.6309199613,-1.0618787367
H,-9.8234655346,-2.9104926439,-1.4903395094
H,-8.2576331132,-5.5794125165,0.5096063106
H,-6.902979504,-5.0614842437,-0.4910572479
H,-6.9466773189,-4.6561376235,1.2419188792
C,-1.5798525075,-2.1213082684,-0.1346810186
H,-3.207043094,0.6345487775,-0.269984904
C,5.0916514015,0.9507849178,0.8876061452
C,3.7369608632,-1.0838738452,0.9524577696
C,6.2305689086,0.2048466119,1.2039563372
H,2.7826505174,-1.5914675513,0.8999697041
C,6.1181559919,-1.1681210921,1.3620155076
H,7.1943161608,0.6705298516,1.3526257931
C,4.8816772762,-1.8098677254,1.2337864641
H,7.0011527399,-1.7492459661,1.6052690683
H,4.8159752248,-2.883207237,1.3761815919
C,-2.9128209064,-2.5922786782,-0.229013518
C,-3.1538821781,-3.9669782644,-0.3047000458
C,-0.5097641609,-3.0180816772,-0.0838133615
C,-2.0836256299,-4.8463319972,-0.243310852
H,-4.1504005852,-4.365106967,-0.4319813077
C,-0.7709099962,-4.3769277042,-0.1274625059
H,0.5147947206,-2.6743204923,-0.0287222855
H,-2.2685565678,-5.9136726399,-0.2992980032
H,0.0512076419,-5.0835594291,-0.0895153061
H,2.9927423931,3.3530302693,0.3181422756

1c$_{NHNH}$-2 (closed-shell singlet state)

–2637.3563847 hartree
C,-2.4766714098,6.403919317,-2.5565576257
C,-2.7414144813,6.8933513546,-1.2824534699
C,-1.8233237815,5.1747213301,-2.7393056343
C,-2.3636964462,6.1734409366,-0.1376109866
H,-3.2473434587,7.8465376049,-1.1669781283
C,-1.4561981111,4.4736066603,-1.6097405399
H,-1.6104086122,4.78391513,-3.7284756208
C,-1.723533278,4.9674080329,-0.3264648886
H,-2.563624078,6.544384882,0.8618077638
O,-0.8203757537,3.2769461082,-1.5269553156
O,-1.2662232204,4.1003601677,0.6164024205
C,1.3278735742,1.6367422046,0.0609597667
C,-0.8242827733,0.5618931887,-0.0235604097
C,0.5573335007,0.4774378927,-0.1237111339
H,1.0257607245,-0.4662441792,-0.3566281352
B,-0.6876118263,2.9880524127,-0.0957873729
O,-1.4175079751,1.7239768832,0.2388077604
O,0.7376727439,2.8059855409,0.2760359909
C,-3.8342535199,-1.39365964,-0.0428262707
C,4.8593528445,0.7521741603,0.0194594723
N,3.5027135646,0.4883758403,-0.0195284204
C,3.6421966752,2.7297963061,0.0990379536
C,-1.7129985146,-0.5271735356,-0.1417225331
N,-3.0316572619,-0.2699099953,0.0135293576
C,2.72695534,1.6194332171,0.0484576099
H,-2.7779338879,6.9789177941,-3.4263790596
C,5.8093553886,-0.2577341309,0.0137090004
C,5.434058032,-1.6172093766,-0.2739800914
C,7.1885921826,-0.0061585141,0.3252267232
C,6.3101739125,-2.649607287,-0.2796001485
H,4.4070817155,-1.8147600728,-0.5546426097
C,8.133457597,-0.9776401658,0.3590970766
H,7.4631471857,0.9999649085,0.5976767381
C,7.7357181009,-2.3726272434,0.0444509033
O,8.5652790754,-3.2860782567,0.0516748117
C,5.8912246448,-4.0816655067,-0.6251648531
C,6.1796739533,-5.0156676782,0.5678557221
C,4.3908236749,-4.1792828009,-0.9378081888
C,6.6591021568,-4.570931302,-1.8698387506
H,7.2423252929,-5.0343632035,0.806334049
H,5.6259304677,-4.6942146287,1.4563761943
H,5.859912334,-6.0339688127,0.3228064559
H,4.108862297,-3.5719772279,-1.8045189315
H,4.1442876261,-5.2180703302,-1.1742065005
H,3.7732100241,-3.8846105691,-0.0820043515
H,6.3412927382,-5.5881842896,-2.121620566
H,6.4495290668,-3.9302393919,-2.732819824
H,7.7339035676,-4.5786138896,-1.6926836399
C,9.5916346527,-0.6867230721,0.7277512788

C,9.9896667978,-1.4865069971,1.9846379939
C,10.5158555861,-1.0665258089,-0.4472943837
C,9.8160727904,0.8011655811,1.0324358455
H,9.3556764054,-1.2143587296,2.8350868415
H,9.9063987648,-2.5594850821,1.8147519334
H,11.0262892472,-1.2573224492,2.2528632621
H,10.2591722856,-0.4929931101,-1.3442082957
H,11.5540344209,-0.8349611131,-0.1871195377
H,10.4457318429,-2.1286581011,-0.6791864129
H,10.8678096405,0.9583391028,1.2867390134
H,9.5923952339,1.4364477052,0.1687722752
H,9.2199464755,1.1444012419,1.8842495882
C,-5.2049189074,-1.3349799101,0.1313540514
C,-5.8805890404,-0.0613150766,0.2007384397
C,-6.0067344848,-2.5219062943,0.2751620439
C,-7.2173638032,0.064656381,0.3604456504
H,-5.2924280671,0.8382775362,0.079555533
C,-7.347599866,-2.4951296405,0.4526404766
H,-5.4987145142,-3.4713446499,0.2889694904
C,-8.0364898489,-1.1747210848,0.4882342227
O,-9.25714146,-1.1085793922,0.6257413153
C,-7.9143591131,1.4270081776,0.4008009338
C,-8.6409670231,1.6027501894,1.7503200495
C,-6.9149275188,2.5829955734,0.2550227425
C,-8.9274394755,1.533426339,-0.7576108933
H,-9.4127069487,0.8466614597,1.8904078223
H,-7.933179879,1.5434906495,2.583695083
H,-9.1126129024,2.590046833,1.7830470917
H,-6.3830246154,2.5562209687,-0.7012371569
H,-7.4588397825,3.5302895011,0.2938292172
H,-6.1784068815,2.6006071091,1.0647111706
H,-9.4009909728,2.5201418346,-0.7367139638
H,-8.4251335256,1.425153998,-1.7245073184
H,-9.7058253984,0.7752306395,-0.6790057089
C,-8.175789089,-3.7701236148,0.6319548981
C,-8.8892456829,-3.7493574597,1.9991502839
C,-9.2173877384,-3.8831918278,-0.5004241137
C,-7.2984015536,-5.0291065252,0.5834798498
H,-8.1631193992,-3.6846455162,2.816241366
H,-9.5782067827,-2.9089532863,2.0757498731
H,-9.4579276297,-4.6758460683,2.1291140196
H,-8.7265517666,-3.9137694933,-1.4788278033
H,-9.7865862311,-4.8109486124,-0.3819134805
H,-9.9139929394,-3.0459267981,-0.4847942064
H,-7.9320363931,-5.9101253562,0.7158831888
H,-6.7883075002,-5.1383873884,-0.379601096
H,-6.5497231567,-5.0433074837,1.3823468363
C,-1.5933023334,-1.9512644158,-0.3664623457
H,-3.3235157991,0.6760661546,0.2253270887
C,4.957619366,2.2108725448,0.0612550009
C,3.3967891869,4.1059753536,0.1270901287
C,6.040624312,3.0916222506,0.0019315661
H,2.380281037,4.474083583,0.163256523
C,5.7923961571,4.456921999,0.0323267139
H,7.0600311508,2.7424997269,-0.0872354731
C,4.4871377727,4.9599368881,0.1034164432
H,6.6287372692,5.1469065898,-0.0079940896
H,4.3275077523,6.0326154654,0.1263091715
C,-2.90196535,-2.4932370342,-0.313632921
C,-3.0930891562,-3.8542878095,-0.5681460071
C,-0.4928552472,-2.7695359928,-0.6349090856
C,-1.9915652762,-4.6558605598,-0.8264140444
H,-4.0777418718,-4.2988176422,-0.5895556978
C,-0.6992842433,-4.1205661797,-0.8534723394
H,0.5111112988,-2.3697201561,-0.6893161018
H,-2.1377081086,-5.7128462586,-1.0211370825
H,0.1465540011,-4.7664188805,-1.0628696272
H,3.1364123175,-0.4403111605,0.1040424493

$1c_{NHNH}$-3 (closed-shell singlet state)

–2637.3492306 hartree
C,-2.2610314897,6.2555925569,-2.1927554528
C,-2.5821156371,6.7339494523,-0.9274833573
C,-1.6025179103,5.026634269,-2.3572455428
C,-2.2577849056,6.0025727045,0.2264185874
H,-3.0914439504,7.6869430575,-0.8261543221
C,-1.2878778815,4.3143073055,-1.2189662315
H,-1.3473898658,4.643768159,-3.3394851733
C,-1.6116034607,4.797144544,0.0554569247
H,-2.5023639337,6.3644025483,1.2192387408
O,-0.6588085639,3.1155720049,-1.1191312474
O,-1.1985363639,3.9206911683,1.0102941182
C,1.4594809279,1.5141840798,0.5425867405
C,-0.672952911,0.4027874486,0.3397441934
C,0.7245685751,0.3486210339,0.29987845
H,1.2298819681,-0.589518612,0.1085146081
B,-0.5802293211,2.8190723477,0.3145760416
O,-1.2856712623,1.54480736,0.620871976
O,0.842059822,2.669888816,0.7330484129
C,-2.1060395026,-2.8676996265,-0.4093675859
C,5.0326934128,0.7709647191,0.605283837
N,3.6867886225,0.4472428761,0.5799492537
C,3.7276433296,2.6884510194,0.7145521769
C,-1.4945512928,-0.7212161168,0.1443063495
N,-1.0682343872,-1.9610743397,-0.2535333513
C,2.8653573659,1.5405175188,0.6094223735
H,-2.5222826905,6.8388769056,-3.069978373
C,6.0297326163,-0.1913981768,0.5673726422
C,5.723158177,-1.5827120146,0.7765206655
C,7.4002602186,0.1526897463,0.3057194139
C,6.6580623519,-2.5620114732,0.7651322994
H,4.7031975125,-1.8508201552,1.0225726042
C,8.3982518143,-0.7614275981,0.2420754705
H,7.6221818788,1.1849789812,0.0899417216
C,8.0714684963,-2.1913425873,0.4771730171
O,8.9483237715,-3.0564606018,0.433119811
C,6.3196901374,-4.0264319172,1.0564344451
C,7.1121171035,-4.5165000746,2.2857193847
C,4.8277022147,-4.2164996736,1.3621241345
C,6.6620792094,-4.8988758392,-0.1686204225
H,8.1861604711,-4.4666074434,2.1114801459
H,6.8705218451,-3.9160005547,3.1689717401
H,6.8437309344,-5.5553981963,2.5033622981
H,4.1944378706,-3.9285076559,0.5164670111
H,4.6360588975,-5.274143273,1.5616769764
H,4.5125060773,-3.655399466,2.2481934734
H,6.4001393806,-5.941473167,0.0395364895
H,6.0915977668,-4.5779354523,-1.0466605052
H,7.7242855705,-4.8498011015,-0.4050175346
C,9.8438447615,-0.3729740571,-0.0814805562

C,10.7675491055,-0.7671885518,1.0893530244
C,10.3053323576,-1.0797256519,-1.3721261274
C,9.9910655845,1.1394652317,-0.3001922745
H,10.4640778492,-0.260425656,2.0114781153
H,10.7544066686,-1.8429370708,1.260629671
H,11.7950249557,-0.4639650731,0.8630924019
H,9.6707258192,-0.7967764149,-2.2185178584
H,11.3313647706,-0.7789152906,-1.6079143027
H,10.2804151272,-2.1633810095,-1.2623325764
H,11.0367476029,1.3669799336,-0.5240453126
H,9.3922245124,1.4968629944,-1.1441970497
H,9.7180818704,1.7129003352,0.5921019062
C,-1.9087730005,-4.1452859369,-0.9072448055
C,-0.5816330934,-4.6803963396,-1.071038212
C,-3.0106172423,-4.9702192434,-1.3223456308
C,-0.3308651349,-5.9131420089,-1.5712030236
H,0.2554504528,-4.0843182047,-0.7296486479
C,-2.8570129822,-6.2103450457,-1.8451258424
H,-3.9999085778,-4.5473728987,-1.2622142393
C,-1.4840989739,-6.7593758805,-1.9868194348
O,-1.2982719469,-7.8894795413,-2.442143981
C,1.0871762997,-6.4730872247,-1.7101393025
C,1.3830684567,-6.7920983482,-3.1900376027
C,2.1451569031,-5.4700983109,-1.2308484357
C,1.2373453663,-7.7505980706,-0.8589763777
H,0.6999109319,-7.547151386,-3.5768253827
H,1.2974241632,-5.891450941,-3.807091011
H,2.4069650705,-7.1678912395,-3.2857016361
H,2.0269559754,-5.2211222372,-0.1711523691
H,3.137740719,-5.9123029362,-1.350126525
H,2.1300927277,-4.5443337817,-1.8168394977
H,2.2602042122,-8.1311109925,-0.9471210156
H,1.0483487407,-7.5379389098,0.1985209474
H,0.5498325655,-8.5288731139,-1.187729186
C,-4.0466833648,-7.0546477574,-2.3113800958
C,-3.9157582925,-7.3683554933,-3.8155391675
C,-4.1043993247,-8.3691768838,-1.5062778783
C,-5.3800828427,-6.3229913623,-2.1022157111
H,-3.8908188898,-6.4454844696,-4.4044385758
H,-3.0134078715,-7.9419583844,-4.0247543711
H,-4.7813501241,-7.9523310787,-4.1449618735
H,-4.2150070183,-8.164893192,-0.4360936855
H,-4.9710718223,-8.9567924279,-1.8264085207
H,-3.2063345679,-8.9669773864,-1.6574155168
H,-6.1960992849,-6.9639907407,-2.4465915924
H,-5.5660097588,-6.1002365425,-1.046112486
H,-5.4354441888,-5.3902624354,-2.6728401481
C,-2.9167850505,-0.8235609516,0.3360468158
H,-0.1546441888,-2.12827749,-0.6417507381
C,5.0658438645,2.2286374839,0.7039549919
C,3.4204717233,4.0487813885,0.8248511195
C,6.1079916485,3.1481852912,0.8486357251
H,2.3885824494,4.3718826251,0.8336411176
C,5.7977084077,4.4967880998,0.9542673968
H,7.1429748299,2.8406854981,0.9007942457
C,4.4709221499,4.944570165,0.9339436635
H,6.6022686448,5.2164371644,1.0636823617
H,4.2624154607,6.0055695559,1.0194535385
C,-3.3019645981,-2.1483265721,0.0236420348
C,-4.6275922136,-2.5422756289,0.2248592328
C,-3.8416637826,0.1214515691,0.7935577715
C,-5.5416357683,-1.6008333088,0.676653386
H,-4.9556516498,-3.5593721213,0.0612159638
C,-5.1575026386,-0.2812369691,0.9476943041
H,-3.5201357759,1.1297290127,1.0170035453
H,-6.5738334664,-1.8981271964,0.8295243992
H,-5.897891388,0.4308253796,1.2960433698
H,3.3599015354,-0.4976668342,0.4679451997

1c$_{NHNH}$·Cl^{-} (closed-shell singlet state)

–3097.718375 hartree

C,-2.3064951257,6.6715602938,-1.8604087041
C,-2.6043046542,6.996333221,-0.5423135554
C,-1.6589694954,5.4663431209,-2.1808323662
C,-2.2661253972,6.1277281821,0.5097119454
H,-3.1051824165,7.9332658599,-0.3179474936
C,-1.3294363704,4.6176915465,-1.1435605927
H,-1.4213369807,5.2021265484,-3.2058346442
C,-1.6308217256,4.946778048,0.188008439
H,-2.4925308577,6.3683717377,1.5431417399
O,-0.7119119868,3.4192598788,-1.2011203322
O,-1.2132533023,3.9652892403,1.020771223
C,1.4279985137,1.6091763701,0.277522798
C,-0.7029951198,0.5080110112,0.1005290584
C,0.684992612,0.4613303812,0.019757018
H,1.1847444783,-0.471824573,-0.206314243
B,-0.6021933639,2.9353011032,0.1913024529
O,-1.331650182,1.6560479558,0.3507290781
O,0.8196299232,2.7598167452,0.5657974139
C,-2.0253814082,-2.8411906048,-0.3893669884
C,4.9584468542,0.7559053927,0.2224182187
N,3.6177475927,0.4868069663,0.0634306408
C,3.721010807,2.67838575,0.6076412193
C,-1.4882463299,-0.6474566327,-0.0518744936
N,-1.014734907,-1.9252939471,-0.2006816622
C,2.832037016,1.5844838848,0.3031070758
H,-2.5766510354,7.3571924012,-2.6579584329
C,5.970805622,-0.1825525003,0.0416803013
C,5.7558992604,-1.4048225935,-0.6852366171
C,7.280825279,0.0520713907,0.580670796
C,6.7522357022,-2.2952121706,-0.9333508558
H,4.7664049327,-1.5920203196,-1.0834864169
C,8.3121061798,-0.8159353396,0.4347010509
H,7.414882736,0.9273114672,1.1963099521
C,8.0940333188,-2.0527525317,-0.3519947379
O,9.0147317726,-2.867772171,-0.5057814572
C,6.5369859615,-3.5464105286,-1.7933538374
C,6.735510731,-4.8106683876,-0.9326607425
C,5.1223681501,-3.5962506469,-2.384623509
C,7.5321264269,-3.5574869573,-2.9723018627
H,7.7423662466,-4.8516911143,-0.5160839364
H,6.0095534206,-4.834378902,-0.1138988885
H,6.5746219867,-5.7028456465,-1.5481246861
H,4.9255907939,-2.7410391441,-3.0400028315
H,5.0243690909,-4.5024993068,-2.9906487646
H,4.3411505155,-3.6201477835,-1.621689122
H,7.3417194018,-4.4358129027,-3.5990555478
H,7.3981464901,-2.66766875,-3.5973552766
H,8.5649277935,-3.5918538996,-2.6271850344
C,9.6827412393,-0.572546535,1.0757306362

C,10.0144213253,-1.7074796356,2.0652854088
C,10.7701099656,-0.5015691437,-0.0155928964
C,9.7240058563,0.7494571415,1.8556146115
H,9.2648035711,-1.7565670141,2.8621598832
H,10.048145773,-2.6713146618,1.5581476966
H,10.9892471476,-1.5207177352,2.5302114456
H,10.5640717927,0.31705888,-0.7136517863
H,11.7462222699,-0.3132824813,0.4459802168
H,10.8219906636,-1.4340916438,-0.5766989133
H,10.7203376972,0.8778296231,2.2893711085
H,9.535003371,1.613504837,1.2096542395
H,9.0027087526,0.7669680523,2.6788864672
C,-1.8242198325,-4.206131811,-0.5788670701
C,-0.5935391782,-4.8574372897,-0.2174231622
C,-2.8662220444,-5.0151335534,-1.1463860591
C,-0.4173583565,-6.2011429568,-0.3258128583
H,0.2007259759,-4.2498870763,0.199334046
C,-2.7451730081,-6.3497083311,-1.3516792648
H,-3.7516263,-4.5090231864,-1.4966916968
C,-1.4920345137,-7.021075718,-0.9347330057
O,-1.3473687959,-8.2397189716,-1.1065738607
C,0.8568696764,-6.9034579539,0.1586993446
C,1.591111126,-7.5376822091,-1.0402120765
C,1.823507053,-5.9273731958,0.8404492978
C,0.5009281611,-7.9962284058,1.1882475062
H,0.9651999089,-8.2756836584,-1.5427120957
H,1.8833284681,-6.7677591626,-1.7612277512
H,2.5043670198,-8.0324506072,-0.6911953134
H,1.3763166615,-5.4633472359,1.7259202242
H,2.7086437478,-6.4797398711,1.1715141397
H,2.159740601,-5.1261072111,0.1791545239
H,1.4222608943,-8.4629311836,1.5540307943
H,-0.0151820443,-7.5592134059,2.0502706344
H,-0.133120776,-8.768243264,0.7536284037
C,-3.8471772639,-7.1773741336,-2.0219754818
C,-3.3155316864,-7.8149964495,-3.3212393352
C,-4.3335876387,-8.2828691908,-1.0629743209
C,-5.0646158084,-6.3178407214,-2.3916526031
H,-2.9881123742,-7.0423458929,-4.0249042827
H,-2.4760300117,-8.4787410885,-3.1163027017
H,-4.11191556,-8.393992398,-3.8027227207
H,-4.7391410714,-7.8461967027,-0.1438667327
H,-5.1312587172,-8.8629910659,-1.5409460122
H,-3.5196058635,-8.9574664915,-0.7993590273
H,-5.8212550098,-6.9529863335,-2.8621545483
H,-5.5256757745,-5.8582546277,-1.5108766374
H,-4.8114564962,-5.5261269244,-3.103988065
C,-2.9275172109,-0.7266390223,-0.0869778856
H,-0.0109092318,-2.1620621112,-0.2598558296
C,5.0401270976,2.1810128881,0.5569573629
C,3.4527322281,4.0242780571,0.8753902389
C,6.1115527167,3.0649501226,0.7149623054
H,2.4296238871,4.3733288199,0.9127577014
C,5.8431270283,4.4004362015,0.9822822935
H,7.1382581747,2.7428451549,0.6095779735
C,4.5285165248,4.8746683475,1.0736892282
H,6.6710639653,5.0905134238,1.110930889
H,4.3501238495,5.9244764417,1.2826314169
C,-3.273799422,-2.0773547301,-0.3007227301
C,-4.621852706,-2.4479440135,-0.2906065146
C,-3.9001142276,0.2649288398,0.0741491713
C,-5.585872073,-1.4616968674,-0.1327824616
H,-4.9313467448,-3.480285179,-0.3759476469
C,-5.2312617887,-0.1173607756,0.0354187963
H,-3.6016793472,1.2918388786,0.2362043635
H,-6.6341868417,-1.7432126532,-0.1285903948
H,-6.0073581695,0.6313041014,0.1568460761
H,3.2432460929,-0.4657742517,-0.0783027814
Cl,2.2089114033,-2.4244696898,-0.3285490297

$1c_{NHNH}$-1 (open-shell singlet state)

–2637.3825655 hartree
C,-1.5457795334,6.1280772168,-3.3207752898
C,-1.9537077086,6.7061529775,-2.1236212521
C,-0.9678986826,4.8492518075,-3.3485640717
C,-1.8011704691,6.0299682952,-0.9030040904
H,-2.3975659594,7.6963700858,-2.12921616
C,-0.8221696909,4.1932892461,-2.1449459252
H,-0.645687519,4.3883302637,-4.2758688622
C,-1.2328152343,4.775147664,-0.9407284873
H,-2.115100071,6.4700413313,0.037139764
O,-0.2965067038,2.9595951877,-1.9100484859
O,-0.9814409995,3.9295721809,0.0977648651
C,1.4892141785,1.3100067988,0.1830669264
C,-0.6837114598,0.3437373036,-0.1100438868
C,0.692526085,0.1760454114,0.0332170374
H,1.1275859816,-0.806674764,0.0588144057
B,-0.3853449908,2.7522176106,-0.4739541366
O,-1.2146198239,1.5440435792,-0.1709040565
O,0.9741171333,2.5156519033,0.104085808
C,-3.7863787088,-1.4515597079,-0.3248028688
C,4.8879694729,2.3641027841,0.6740736529
N,3.5514085822,2.4910391105,0.4599326578
C,3.8657337976,0.2921517172,0.7099707279
C,-1.6383203824,-0.7146745891,-0.1716976032
N,-2.9529101161,-0.3779692994,-0.3247824134
C,2.8914919009,1.2956206215,0.4452976989
H,-1.674610808,6.6719260619,-4.2509618716
C,5.7609181044,3.4951903669,0.7021035118
C,5.2381959458,4.7985933955,0.9338863838
C,7.1548400399,3.3483996114,0.4695694756
C,6.0227133462,5.915925217,0.9630034453
H,4.1803394163,4.8970802813,1.1418730095
C,8.0173682075,4.4083511894,0.4682234982
H,7.5249823984,2.362676016,0.232584984
C,7.4752645318,5.7578180111,0.7320886346
O,8.227798884,6.7448863874,0.7571809865
C,5.4503399557,7.3078419265,1.2417537661
C,5.7286584716,8.2371465823,0.042024709
C,3.9306416346,7.2679313582,1.4572262351
C,6.0902348372,7.8937075807,2.5172830493
H,6.7979170544,8.3601671175,-0.1262397
H,5.2709581635,7.8420763281,-0.8708067126
H,5.2908657087,9.2217103863,0.2354345421
H,3.6494312433,6.6570337659,2.32096082
H,3.572608977,8.2829582332,1.6484390635
H,3.3956287666,6.8973053354,0.57716932
H,5.6558148591,8.8771817859,2.7236626417
H,5.8913158883,7.2524258075,3.382339863
H,7.1679006729,8.0077703551,2.4055379101

C,9.5125657994,4.2408566349,0.1862015881
C,9.9095368036,5.0573099552,-1.0607335522
C,10.331854534,4.7151359426,1.4044625381
C,9.8815647216,2.774282106,-0.0800512825
H,9.3461051226,4.7253959183,-1.9390190332
H,9.7307518849,6.121577827,-0.9118541423
H,10.9739506069,4.90978198,-1.2704963942
H,10.0713931148,4.1373499681,2.297526784
H,11.398455242,4.5650354948,1.2078200918
H,10.1621707253,5.7714198575,1.6095285231
H,10.9554666676,2.7076212184,-0.2741530338
H,9.6666705331,2.1321330234,0.7804937961
H,9.3658036066,2.3691928899,-0.9564385022
C,-5.2021871294,-1.310577339,-0.4605454253
C,-5.7538968577,-0.1231329719,-1.018434804
C,-6.0841554061,-2.3317751132,-0.0157564685
C,-7.0978536216,0.0707111548,-1.163973271
H,-5.0737410525,0.6349874107,-1.3853914779
C,-7.4432764398,-2.2236126579,-0.1088590252
H,-5.6521943682,-3.1978308313,0.461954666
C,-8.0128375041,-0.9988644775,-0.7090126698
O,-9.2417921242,-0.8686067835,-0.8273331944
C,-7.6754174408,1.3407729593,-1.7933274924
C,-8.5911449625,2.0566322897,-0.7787061477
C,-6.5729295414,2.3276090832,-2.2032369939
C,-8.4785580146,0.9812868131,-3.0603813602
H,-9.4352009198,1.4299281715,-0.4930674236
H,-8.033705554,2.3302016436,0.1231756765
H,-8.9770621947,2.9788418734,-1.2248273789
H,-5.8988123592,1.9085833496,-2.9569437357
H,-7.0358961645,3.2154366452,-2.641775835
H,-5.9766960452,2.6633775494,-1.3489015552
H,-8.8643766981,1.8975777311,-3.5186966142
H,-7.8402368064,0.4826027762,-3.7972155457
H,-9.3200446619,0.3299129169,-2.8269451281
C,-8.3839122797,-3.3175196545,0.4028185826
C,-9.3035766111,-2.7507613823,1.5037398768
C,-9.2366796366,-3.8621646905,-0.7618618041
C,-7.6097226342,-4.4991158449,1.0048221158
H,-8.7149120513,-2.3700165553,2.3449035472
H,-9.9300946295,-1.945453587,1.1219554426
H,-9.9531331328,-3.5464785611,1.8827635739
H,-8.5996946036,-4.2806678009,-1.5481955353
H,-9.8861457233,-4.6637025654,-0.3950447353
H,-9.8603435015,-3.0814576139,-1.1955976435
H,-8.3230524189,-5.2509216545,1.3531528375
H,-6.9599719679,-4.9842989399,0.2689846356
H,-7.0025110245,-4.2018542095,1.8657038893
C,-1.5916009854,-2.1352728554,-0.0890775319
H,-3.2318401709,0.5947185157,-0.350067676
C,5.1190034471,0.9586837681,0.8534119901
C,3.7880143153,-1.1045608354,0.8777812342
C,6.2706338385,0.222416749,1.194367258
H,2.8457725835,-1.6306205469,0.7930439263
C,6.1710546689,-1.1423516303,1.343171536
H,7.2205943114,0.7128568199,1.362836638
C,4.9343168616,-1.8033337832,1.179107665
H,7.0515843214,-1.7191070913,1.6051969734
H,4.8861996868,-2.8791379097,1.3117540072
C,-2.9366628413,-2.6005967332,-0.1893838596
C,-3.2013546645,-3.9840105009,-0.2131605792
C,-0.535691397,-3.0594276363,0.0374223953
C,-2.1525475495,-4.86684191,-0.0898799547
H,-4.2071593305,-4.3603726912,-0.3475740457
C,-0.8254644599,-4.4044679813,0.0450138159
H,0.4931065218,-2.7330191709,0.1185654729
H,-2.3447847359,-5.9342619584,-0.1065700091
H,-0.0198432587,-5.124907519,0.1409115303
H,3.0608713491,3.3495543344,0.2439227444

1c$_{\mathrm{NHNH}}$-2 (open-shell singlet state)
–2637.3772678 hartree
C,-2.5284159663,6.6750166109,-2.0523017742
C,-2.7256165104,7.033264479,-0.7231593531
C,-1.9062798895,5.4635963168,-2.3920732159
C,-2.3090163739,6.1953354255,0.3231133931
H,-3.2088155565,7.9758070556,-0.4870674994
C,-1.5010484929,4.647447977,-1.3577496321
H,-1.7460969335,5.1738069247,-3.4247856553
C,-1.6995687296,5.0081964571,-0.0206619231
H,-2.4562759954,6.4636786277,1.3634715315
O,-0.8827697062,3.4364264065,-1.4258421102
O,-1.2133682968,4.0380985798,0.8035072543
C,1.3392993027,1.6390431102,-0.0117902522
C,-0.8069793967,0.5635679393,-0.0698198892
C,0.5752719865,0.4859463874,-0.2251274811
H,1.0390906486,-0.4526330829,-0.4816147207
B,-0.6835066913,3.0109879996,-0.0505902686
O,-1.3873563206,1.7104327415,0.1967780303
O,0.7700342445,2.7901616908,0.2230358908
C,-3.814281669,-1.398429057,-0.0278764922
C,4.8717248702,0.7691585433,-0.0276929206
N,3.5289164078,0.5096710856,-0.0753386554
C,3.6738541975,2.7367605748,0.0648454352
C,-1.7101816546,-0.5396883613,-0.145207818
N,-3.0419360854,-0.2815396701,0.0129991312
C,2.7665752037,1.6464731175,-0.0041510819
H,-2.8592839577,7.3409967992,-2.8426520054
C,5.8368851753,-0.2783446934,-0.0541298165
C,5.4849241446,-1.5716432091,-0.5373672633
C,7.1579842754,-0.0700059316,0.4284548101
C,6.3619121924,-2.6163711877,-0.5685272778
H,4.4929375239,-1.7112539047,-0.9487587229
C,8.1018615569,-1.0572591508,0.4472220341
H,7.3938110218,0.8981713946,0.8428914814
C,7.7361743062,-2.3932940929,-0.0671373587
O,8.5660871345,-3.3161757302,-0.0792003756
C,5.9771976257,-3.9913828891,-1.1206224536
C,6.1376032132,-5.0614973845,-0.0210952346
C,4.517299778,-4.0287543532,-1.59564567
C,6.8704933006,-4.3433674749,-2.3277883022
H,7.170630558,-5.1295331056,0.3174398909
H,5.5000727775,-4.8343269535,0.8399044342
H,5.8348173046,-6.0375231939,-0.4141457086
H,4.3270832836,-3.3210816615,-2.4090220353
H,4.2932991866,-5.028838738,-1.9763798294
H,3.8144220057,-3.8264396073,-0.7800459524
H,6.5734530868,-5.3168645799,-2.731290225
H,6.7578478676,-3.6015031901,-3.1252073163
H,7.9204177026,-4.3932912314,-2.0417602728

C,9.5111308566,-0.8251417219,0.9985497513
C,9.7756062815,-1.7708728482,2.1878815949
C,10.5544864752,-1.0747579663,-0.1102103121
C,9.6976989171,0.6149771598,1.4973733601
H,9.0516179727,-1.5979798992,2.9910337143
H,9.7199511261,-2.8158025797,1.8846800982
H,10.7750104654,-1.5792894685,2.5917798856
H,10.3895561747,-0.4020436426,-0.9583778677
H,11.5577597616,-0.8789163566,0.2819729332
H,10.5164744801,-2.1034044411,-0.4667577166
H,10.7163737639,0.7303788554,1.8772220233
H,9.5646889954,1.3494772085,0.696328349
H,9.0144420381,0.8625706573,2.3158972885
C,-5.2350871289,-1.3425741971,0.1284895969
C,-5.9331550385,-0.1241667842,-0.1006593738
C,-5.9693401738,-2.4856754521,0.5426620129
C,-7.2866158219,-0.0090392938,0.043181984
H,-5.3696133797,0.7317662488,-0.4496948949
C,-7.3239349034,-2.4648849886,0.7244753397
H,-5.4166223438,-3.3841271704,0.7713677856
C,-8.0494457347,-1.2032702307,0.4670066856
O,-9.2818316066,-1.1460329651,0.6069812483
C,-8.0280057562,1.3006013873,-0.2356832359
C,-8.753565614,1.7722760224,1.0416731602
C,-7.0690177325,2.4193150047,-0.6671398854
C,-9.0503971877,1.094047984,-1.372271207
H,-9.498761415,1.0472708554,1.3670058212
H,-8.0406061478,1.9352949616,1.8565732818
H,-9.2566953317,2.7240797978,0.8433669727
H,-6.5400549524,2.1790161816,-1.5947868278
H,-7.6450357186,3.3300766531,-0.8504064874
H,-6.3303934188,2.6549667923,0.1055120361
H,-9.5560268357,2.0418125813,-1.5832958179
H,-8.5505947951,0.7704495886,-2.2912608738
H,-9.8020476494,0.3532140063,-1.1019612444
C,-8.1003950471,-3.6952631492,1.2002527668
C,-8.8069600051,-3.3895626605,2.5368260116
C,-9.1434973747,-4.0995652959,0.1377622689
C,-7.1758281007,-4.9001780799,1.4265317684
H,-8.0798131884,-3.11209277,3.3070854968
H,-9.5282856011,-2.5803808996,2.428598226
H,-9.3365344454,-4.283159604,2.8828828447
H,-8.6579186319,-4.332243818,-0.8158065979
H,-9.6745506172,-4.9972422352,0.4708555291
H,-9.8717962551,-3.3060826577,-0.0251246931
H,-7.7756865394,-5.7504087834,1.762127622
H,-6.6645867881,-5.2059739882,0.50775274
H,-6.4235994429,-4.7069136937,2.1978751142
C,-1.5901867656,-1.9452730709,-0.3315491532
H,-3.3600495836,0.657632321,0.216852847
C,4.991286774,2.1963826657,0.0395078895
C,3.4631203012,4.1293260205,0.1118688945
C,6.0999999911,3.0659790762,0.0135931407
H,2.456968316,4.5258937965,0.1393729481
C,5.8757209675,4.4237947647,0.0603987402
H,7.1119702851,2.6899309248,-0.0636729163
C,4.565765193,4.9518430659,0.1188076912
H,6.7205634881,5.1043262167,0.0425455057
H,4.430112308,6.0276034695,0.1562210855
C,-2.9091342533,-2.486315665,-0.2631897767
C,-3.1171852369,-3.8616385794,-0.4877959431
C,-0.4938270335,-2.7976912816,-0.5717901887
C,-2.0301576662,-4.6721889966,-0.7227178334
H,-4.1140490068,-4.2830679585,-0.4990056714
C,-0.7217202668,-4.1420183572,-0.7542592364
H,0.5175376478,-2.4152484608,-0.6242636825
H,-2.1785367842,-5.7325418752,-0.8964331908
H,0.1155263237,-4.8065543177,-0.940480496
H,3.1515248593,-0.4222266832,-0.0067943337

1c$_{NHNH}$-3 (open-shell singlet state)

–2637.3711812 hartree

C,-2.4211631192,6.6753153291,-1.6557128285
C,-2.6645000325,6.9716690347,-0.319012532
C,-1.7804809856,5.4837421897,-2.0292328804
C,-2.2775225307,6.088901956,0.7014178773
H,-3.1616437107,7.899922069,-0.0566490492
C,-1.4043636314,4.6233956944,-1.0201324386
H,-1.5852522349,5.2416501178,-3.0681445895
C,-1.6493227915,4.9217964118,0.32455318
H,-2.4614507573,6.3082896188,1.7473991065
O,-0.7757710996,3.4201232289,-1.1224068887
O,-1.1843634889,3.9174892241,1.1196662852
C,1.4319851611,1.6053411191,0.2487829027
C,-0.7008169552,0.4972348473,0.112750723
C,0.6901529115,0.4528620925,-0.0335927821
H,1.1856085199,-0.4629673234,-0.3238861657
B,-0.6160772713,2.9360587001,0.2378819004
O,-1.3039891744,1.6165607073,0.4044388668
O,0.834008533,2.7257794147,0.5470123968
C,-2.1196960826,-2.8257181547,-0.3404282471
C,4.9942923805,0.8555353566,0.1737619574
N,3.6627176268,0.5581775347,0.0746472604
C,3.7279398287,2.7552926305,0.4840363283
C,-1.5558724301,-0.6414975042,-0.0052489416
N,-1.1040029918,-1.917738947,-0.2203817013
C,2.8596713858,1.6523341462,0.2700233936
H,-2.7303920894,7.3747724219,-2.4256537577
C,5.9995261203,-0.1446005805,0.0215085776
C,5.7124485477,-1.3572295602,-0.6675563427
C,7.2967129694,0.0343550322,0.5740396379
C,6.6323566278,-2.3524345115,-0.831255973
H,4.7398957925,-1.4624440597,-1.1326052239
C,8.2774103752,-0.9120940478,0.4765248189
H,7.481565574,0.9361594858,1.1378203015
C,7.9785444185,-2.1653764093,-0.2464724697
O,8.8414193319,-3.0503264379,-0.3626204564
C,6.3232771169,-3.6319279092,-1.6125934455
C,6.4783129014,-4.8596174281,-0.6913974273
C,4.8873529323,-3.636284849,-2.1564062636
C,7.2784820846,-3.7576677723,-2.8172333216
H,7.4978319394,-4.9483085501,-0.3183587504
H,5.795989033,-4.7942770479,0.1627908396
H,6.2307717539,-5.7684487299,-1.2494161258
H,4.7054351049,-2.8121263208,-2.853722653
H,4.7155490422,-4.5681935728,-2.701428741
H,4.1440054743,-3.5900857501,-1.3532812681
H,7.0328333305,-4.6604753361,-3.3857036206
H,7.1729131571,-2.8996119242,-3.4891868813
H,8.3167009741,-3.8239475838,-2.4943669232

C,9.6593463267,-0.7196974731,1.1064738859
C,9.9216497999,-1.8234846384,2.1513949559
C,10.7449927191,-0.767444597,0.0113099334
C,9.7767745978,0.6351772196,1.8193022565
H,9.1673864997,-1.7955137887,2.944761003
H,9.9139372302,-2.8126777911,1.6949583812
H,10.9003470744,-1.6625338286,2.6149110068
H,10.5813516189,0.0184733768,-0.7334582355
H,11.7275776973,-0.5997812119,0.4641462262
H,10.7567265006,-1.7321257022,-0.4945860305
H,10.7786562385,0.725852962,2.2474266222
H,9.6400489838,1.4763026962,1.1318194411
H,9.0607806303,0.7354689104,2.6411152747
C,-1.8653437002,-4.2085539201,-0.5810028716
C,-0.5968405245,-4.7747291388,-0.2680516206
C,-2.8590934215,-5.0432185509,-1.1603160982
C,-0.2962198658,-6.0879892154,-0.4881882235
H,0.1369480255,-4.1463839894,0.2217493567
C,-2.645736191,-6.3650351182,-1.4331467956
H,-3.7960065307,-4.5866526664,-1.4410327958
C,-1.3319312498,-6.9512366745,-1.0974324242
O,-1.0970175021,-8.1493052003,-1.3225404077
C,1.0573443128,-6.6926015118,-0.106873042
C,1.7574336651,-7.2467178599,-1.3649412754
C,1.9913602978,-5.6501216325,0.5243656152
C,0.858082255,-7.8242143849,0.9222608244
H,1.1704744667,-8.0388656578,-1.8279965064
H,1.9197335475,-6.4530288983,-2.1019259663
H,2.735811494,-7.6533336361,-1.0892013583
H,1.5824262869,-5.2337972569,1.450721473
H,2.9422524605,-6.1282178119,0.7738873523
H,2.2141624978,-4.8280858313,-0.1643739999
H,1.832346108,-8.2326249974,1.2096195069
H,0.3729291052,-7.4460112532,1.8280971257
H,0.2523101878,-8.6309222369,0.5114215708
C,-3.7147016168,-7.2431989132,-2.0886911592
C,-3.1961565053,-7.7904054203,-3.434283358
C,-4.0763056144,-8.4146694971,-1.1520940491
C,-5.0035211945,-6.4573775058,-2.3699391064
H,-2.947128251,-6.9716440088,-4.117440947
H,-2.3127543678,-8.4128595832,-3.2961887231
H,-3.9764472562,-8.3950694848,-3.9076997324
H,-4.4582822621,-8.0442286654,-0.1950142297
H,-4.8621407103,-9.021420659,-1.6135750907
H,-3.2134967167,-9.0518180363,-0.9616375768
H,-5.7337919223,-7.1264027281,-2.8329486819
H,-5.4568309116,-6.0664784915,-1.4531198543
H,-4.8397746233,-5.624869243,-3.061455452
C,-2.9724039794,-0.7219208026,0.0549283129
H,-0.1380498126,-2.1587628758,-0.3739285375
C,5.0635602453,2.2643274089,0.4201781877
C,3.4716335626,4.1252482217,0.6974308792
C,6.1441660863,3.1636512844,0.524044456
H,2.4532267577,4.4856146669,0.7515108903
C,5.8747160128,4.497334289,0.7320121486
H,7.1686953042,2.8293054164,0.4227871203
C,4.5465461998,4.9732780879,0.8274520873
H,6.6964780175,5.2009076182,0.8149791511
H,4.3748166635,6.031706659,0.9927358734
C,-3.3308779515,-2.0844394594,-0.1554479895
C,-4.6852062012,-2.4691587371,-0.0842932355
C,-3.9600588901,0.2569709295,0.2876890915
C,-5.6354119573,-1.4997983872,0.143975882
H,-4.984333776,-3.504559167,-0.1844720509
C,-5.2754032132,-0.1436654815,0.3188494262
H,-3.6761250169,1.2888309916,0.4443760567
H,-6.6817039415,-1.7813999601,0.200278742
H,-6.0535467612,0.5914900755,0.4952683023
H,3.3219518593,-0.3871446453,0.0020564415

$1c_{NHNH}\cdot Cl^{-}$ (open-shell singlet state)

–3097.7378431 hartree
C,-2.4076722992,6.9099626359,-0.9054286718
C,-2.6792408874,7.0144470589,0.4537946341
C,-1.7567428289,5.7806471087,-1.4293598826
C,-2.3108576302,5.993256088,1.3458420905
H,-3.183447632,7.8968610715,0.8358267527
C,-1.398249573,4.7824849865,-0.5476131278
H,-1.5391227874,5.6870527454,-2.487877854
C,-1.6724910498,4.8883431853,0.8239179298
H,-2.5166472084,6.061864231,2.4087102908
O,-0.7696476094,3.6129737859,-0.8080355083
O,-1.225841751,3.7880236963,1.4766809687
C,1.4030844183,1.5665165318,0.4700698418
C,-0.7135204949,0.504687391,0.12874344
C,0.6726643362,0.4508325326,0.0853065398
H,1.1761890271,-0.465657482,-0.1860990923
B,-0.6293289045,2.9204335684,0.4763312681
O,-1.3421737408,1.6152382417,0.4385272021
O,0.8082218904,2.6940245026,0.7852053707
C,-2.0329627196,-2.7784189874,-0.6748806243
C,4.92997644,0.7144312816,0.4483365367
N,3.5979704529,0.4351965468,0.3679980514
C,3.7159558951,2.6302300105,0.8341051148
C,-1.5340273077,-0.6359244917,-0.1357437478
N,-1.0403144201,-1.8915640563,-0.3802456594
C,2.8294170788,1.5530085553,0.5679542764
H,-2.701669586,7.7116642008,-1.5760213865
C,5.9574974968,-0.2659890603,0.2751459438
C,5.7287995611,-1.6443938847,0.540684229
C,7.2465979932,0.141297252,-0.1688014185
C,6.7202940881,-2.5820602607,0.4369235979
H,4.7393792111,-1.9450806494,0.8612542141
C,8.2778216427,-0.7350516517,-0.3487255423
H,7.3775571869,1.1806787297,-0.430174663
C,8.0511839173,-2.159133781,-0.0387973034
O,8.9693665434,-2.988455236,-0.1892149013
C,6.4886545906,-4.0527152987,0.7982049671
C,7.489961126,-4.4979485935,1.884242023
C,5.0752666202,-4.2871153022,1.346900664
C,6.6596710802,-4.9284175779,-0.4600893048
H,8.5196161465,-4.4274791444,1.5353376468
H,7.3800739148,-3.8844230715,2.7852482389
H,7.2830432046,-5.5372299478,2.1623629259
H,4.2934578824,-4.0192543955,0.6324499293
H,4.9603718315,-5.3490974688,1.5848056263
H,4.8992778125,-3.7237897472,2.269594417
H,6.4812529191,-5.9781248754,-0.2019726668
H,5.9321263001,-4.6446154466,-1.2267233108
H,7.6649433788,-4.8370295939,-0.8723931625

C,9.6435943931,-0.2881428476,-0.8796682947
C,10.7411595005,-0.6053507301,0.1564253137
C,9.9570531834,-1.0097717426,-2.2059401005
C,9.68543779,1.2225563119,-1.1518596151
H,10.5450839175,-0.0844420728,1.0998109122
H,10.7966157576,-1.6755071314,0.353437458
H,11.7127028769,-0.2655662095,-0.219824961
H,9.1962309756,-0.7815693184,-2.9597067354
H,10.925178458,-0.6699811284,-2.5909010735
H,9.9961053236,-2.0895652956,-2.0655282235
H,10.6784894457,1.4902978044,-1.5251382565
H,8.9564774742,1.5265526075,-1.9094235369
H,9.5072368608,1.8097392716,-0.2450990673
C,-1.8003424412,-4.157489503,-0.9766133257
C,-0.6555999554,-4.8471020686,-0.4899622412
C,-2.7370137664,-4.8664658642,-1.7795778789
C,-0.453775223,-6.1808081186,-0.7213543206
H,0.0591529735,-4.2931169188,0.105324062
C,-2.5865795853,-6.1845357781,-2.1018059532
H,-3.5612236178,-4.3060940691,-2.194837322
C,-1.4185120734,-6.9091612034,-1.567056548
O,-1.2487403469,-8.1138038658,-1.8380887723
C,0.7409630383,-6.9361499519,-0.1305237644
C,1.6490944463,-7.4419571109,-1.2702013493
C,1.5860625884,-6.0379420573,0.781995214
C,0.2504734656,-8.1297551014,0.7150916743
H,1.1127497839,-8.1236858382,-1.9308804002
H,2.0310011428,-6.6016034119,-1.8580345807
H,2.5091848745,-7.9708548225,-0.8449328299
H,1.0106922332,-5.6751685962,1.6404907973
H,2.4250179957,-6.6215824263,1.1733059192
H,1.9979129577,-5.1711895276,0.2599496846
H,1.1134541874,-8.6324859102,1.1654230541
H,-0.3962887766,-7.7856342708,1.5296950321
H,-0.299234946,-8.8513290143,0.1117331497
C,-3.5757281787,-6.9186479702,-3.0126553575
C,-2.8514718636,-7.4342573645,-4.2726527134
C,-4.2090220344,-8.1039128856,-2.2557878646
C,-4.7167365235,-6.0011751517,-3.4756824928
H,-2.410023731,-6.6031014183,-4.8324704864
H,-2.0612463367,-8.1373296147,-4.0110662193
H,-3.568509087,-7.9395990797,-4.9294677745
H,-4.7453725645,-7.7536546135,-1.3672983029
H,-4.9301821866,-8.6130534993,-2.9051103228
H,-3.449921619,-8.8211739853,-1.9452615982
H,-5.3942023847,-6.57123772,-4.1185232642
H,-5.3060445763,-5.6201950311,-2.6352519428
H,-4.3518439761,-5.1485621509,-4.056718405
C,-2.948347931,-0.7129013243,-0.2405085825
H,-0.033566074,-2.1261801214,-0.4088605447
C,5.0370436834,2.1123070354,0.7581538609
C,3.4867308381,3.9859154222,1.1452787042
C,6.1308158777,2.9506455652,1.0551151396
H,2.4748918261,4.3659562481,1.1941463893
C,5.8904286032,4.2721371631,1.3609010397
H,7.1442099373,2.5698415392,1.0687050988
C,4.5754235349,4.7900280787,1.3939388839
H,6.7251688698,4.9268885654,1.5910757992
H,4.4233158925,5.8370772091,1.6363291083
C,-3.2693958425,-2.0545311255,-0.5814373439
C,-4.6210589843,-2.4429551503,-0.6787235864
C,-3.9618694159,0.2492930415,-0.0555333177
C,-5.6000569038,-1.4920366915,-0.4917769605
H,-4.8983948272,-3.4712274159,-0.8734099925
C,-5.2715997089,-0.1497600032,-0.1935463128
H,-3.7005756946,1.2679663017,0.1987575887
H,-6.6442530792,-1.7798452766,-0.564521678
H,-6.0689941132,0.5735436973,-0.0549929629
H,3.2202366423,-0.4939161225,0.1160981529
Cl,2.2073362129,-2.4544916444,-0.393756083

1c$_{NHNH}$-1 (triplet state)

–2637.3804213 hartree
C,-1.5779736894,6.1677623918,-3.2268278714
C,-1.9784706896,6.7293091884,-2.0192813588
C,-0.9982601807,4.8904745859,-3.2756090158
C,-1.8164300927,6.0376257346,-0.8086945878
H,-2.4239393876,7.7187412334,-2.0088409442
C,-0.8433423112,4.2194030588,-2.0816249179
H,-0.681718095,4.4422638607,-4.2110333134
C,-1.2463438467,4.7844438052,-0.8671464858
H,-2.1245239338,6.4647882585,0.1392692709
O,-0.3139578898,2.983059454,-1.8661732325
O,-0.9860382683,3.9251196435,0.1582982735
C,1.4879552307,1.3110482169,0.1977859639
C,-0.6849207569,0.3448687273,-0.0884503277
C,0.6930664107,0.1770153721,0.0384932763
H,1.1299132923,-0.8051506381,0.0489033079
B,-0.3923765627,2.7577532556,-0.4339596103
O,-1.2160706895,1.5422407759,-0.1407216352
O,0.9719382926,2.5139822695,0.1317730247
C,-3.7863477781,-1.4526086009,-0.3178010202
C,4.8889594458,2.3650156687,0.6792492207
N,3.5561485847,2.4952431005,0.467455196
C,3.8660057768,0.2982270932,0.7188854085
C,-1.6437768323,-0.7157966659,-0.1465078951
N,-2.9585346651,-0.3794494839,-0.3134147321
C,2.8950439377,1.2985158982,0.4578863942
H,-1.7141671806,6.7236470658,-4.1487837523
C,5.7685481583,3.5015589732,0.7064011103
C,5.2574046931,4.7930632979,1.0009376798
C,7.1478152249,3.3571763322,0.4119287831
C,6.0460681706,5.9104095387,1.0293427949
H,4.2091700768,4.8828610068,1.2569034072
C,8.0136195035,4.4173405031,0.4071068483
H,7.5035218114,2.3766691655,0.13268291
C,7.4853031548,5.7570892475,0.7324905585
O,8.2412839071,6.7439313471,0.7544772455
C,5.4882951726,7.2934783261,1.373023831
C,5.7172331562,8.257922821,0.1906435372
C,3.9789399389,7.2477440983,1.6512763941
C,6.1825403669,7.8409984611,2.6371897838
H,6.7786641766,8.385587331,-0.0186334007
H,5.2210712302,7.8902668532,-0.7135002438
H,5.2893066986,9.2363398318,0.4314782412
H,3.7335313304,6.611783677,2.5077680201
H,3.6310371859,8.2568988289,1.8870623239
H,3.4068313065,6.9031850604,0.7840708413
H,5.7579803376,8.8175494712,2.8913838659
H,6.0203986836,7.1738646839,3.4902707031

H,7.2545088949,7.9588955162,2.4828207521
C,9.4954730814,4.2558298971,0.0597717383
C,9.8433803144,5.1078825069,-1.1780650913
C,10.3647260122,4.6934516462,1.257031486
C,9.8493007871,2.7970765906,-0.2639468315
H,9.2432028462,4.8031316096,-2.0417154841
H,9.6744671386,6.1676843357,-0.9907981189
H,10.8977217481,4.9640636109,-1.4357226376
H,10.1393856521,4.0903732939,2.1428909976
H,11.4220091535,4.5475311597,1.0128246676
H,10.2062274433,5.7436021314,1.4992544761
H,10.9143450982,2.7344858635,-0.5029757467
H,9.6671503512,2.1300053849,0.585011575
H,9.2976302776,2.418797363,-1.1303201314
C,-5.2087973659,-1.3137562165,-0.4739079086
C,-5.7486209263,-0.159583282,-1.1002785972
C,-6.0920304889,-2.3075045961,0.0181713429
C,-7.0933458245,0.0301800013,-1.2652025607
H,-5.0611587958,0.5738903647,-1.5024252037
C,-7.452326531,-2.202246635,-0.0925023935
H,-5.6630381512,-3.1481982218,0.5426591406
C,-8.0125590565,-1.0103397132,-0.7599138857
O,-9.2421518672,-0.8829805398,-0.8933295591
C,-7.662171872,1.2662917128,-1.9656177349
C,-8.5822568819,2.038400028,-0.9971375669
C,-6.5534912733,2.2262148584,-2.4204522209
C,-8.4582403705,0.841601145,-3.2169614481
H,-9.4305941198,1.4305560884,-0.6847032346
H,-8.0302623014,2.3577804008,-0.1070603653
H,-8.9614322706,2.9368058454,-1.4946223179
H,-5.8757797781,1.764825847,-3.1456347849
H,-7.0104938266,3.0904820944,-2.9094362465
H,-5.9619249512,2.6057591633,-1.581370541
H,-8.8373194106,1.733170569,-3.726735059
H,-7.8170848277,0.3014170355,-3.9213560303
H,-9.3037761363,0.20683816,-2.9542521462
C,-8.398675786,-3.2673507898,0.4665460242
C,-9.3262084962,-2.643957026,1.5297450285
C,-9.2430832454,-3.8693348573,-0.6758649101
C,-7.6313039034,-4.4177868151,1.1341392975
H,-8.743665581,-2.2209954378,2.354825248
H,-9.9488828924,-1.8589111332,1.1022230251
H,-9.9793296861,-3.4188871423,1.9440085425
H,-8.6006250619,-4.3273817546,-1.435233543
H,-9.8959822648,-4.6509998276,-0.2739923568
H,-9.8626672737,-3.1110774823,-1.1530995943
H,-8.3488148445,-5.1500994224,1.5139714541
H,-6.9759284417,-4.9403272682,0.429641852
H,-7.0308971294,-4.0780616266,1.9839376185
C,-1.5973661612,-2.1305841312,-0.0545897816
H,-3.2417183722,0.5918550813,-0.3483966437
C,5.1214563218,0.9661768005,0.8581687465
C,3.7933037187,-1.1014159614,0.886651735
C,6.2780817907,0.2311304718,1.1933218678
H,2.8521529114,-1.6299876861,0.8044196532
C,6.1808607924,-1.1317028042,1.3424028582
H,7.2275084649,0.7258878342,1.3547929513
C,4.9416369781,-1.7952766074,1.1832251098
H,7.0626021377,-1.7085702277,1.6001001263
H,4.8972149654,-2.8713051892,1.3158686185
C,-2.9435160777,-2.5969294021,-0.1665183142
C,-3.2115740763,-3.9819582856,-0.1809329511
C,-0.5443914502,-3.0586690005,0.0951762419
C,-2.1670779913,-4.8637563294,-0.038265815
H,-4.2182224887,-4.3549041701,-0.3217349507
C,-0.8386129915,-4.4007943833,0.1100684746
H,0.4837226168,-2.733981422,0.1913819382
H,-2.3593738736,-5.9312589145,-0.0478304202
H,-0.0366072172,-5.1227394076,0.2239895042
H,3.0686816008,3.3565963923,0.2551951738

$1c_{NHNH}$-2 (triplet state)

–2637.3753678 hartree
C,-2.5376156892,6.7047427431,-1.9578608443
C,-2.7238144964,7.046070733,-0.622634503
C,-1.9201783812,5.4969605657,-2.3182168991
C,-2.3004616364,6.1943222805,0.4095689298
H,-3.2035496897,7.9862592735,-0.3707164041
C,-1.5083755711,4.6673310485,-1.2974499465
H,-1.768438625,5.2202205339,-3.3557448971
C,-1.6956754572,5.0109306937,0.0454901223
H,-2.4390526425,6.4495230424,1.4543841939
O,-0.8925807413,3.4556087922,-1.3855967026
O,-1.2044635099,4.0289026262,0.8530459979
C,1.3419071908,1.6428791894,-0.0082379613
C,-0.8026427326,0.5659442531,-0.0583687929
C,0.5774178128,0.491694775,-0.2333224795
H,1.0393748947,-0.4432372065,-0.5055474866
B,-0.6826299464,3.014163219,-0.0189832539
O,-1.3811592847,1.7085223034,0.2183666581
O,0.7748425052,2.7888081916,0.2394395221
C,-3.8099397668,-1.3979041599,-0.015225034
C,4.8725600205,0.7740832341,-0.0339657984
N,3.5323505419,0.5159726512,-0.0781840608
C,3.6795444396,2.7403342491,0.0684853149
C,-1.7086929061,-0.5406005377,-0.1263687911
N,-3.042670334,-0.2816444388,0.0255732161
C,2.7730198533,1.6547021589,-0.0007923742
H,-2.8735318533,7.381443748,-2.7368584062
C,5.8402811664,-0.280144908,-0.0681916641
C,5.496667893,-1.5545665571,-0.5975374309
C,7.1468775639,-0.0858249697,0.450996526
C,6.3738625093,-2.6013109651,-0.6374673424
H,4.514033442,-1.677812239,-1.0359324873
C,8.0904635752,-1.0759396495,0.4629103755
H,7.372490279,0.8710283535,0.8970937816
C,7.7345300506,-2.3947555405,-0.09751921
O,8.5642681182,-3.3195609163,-0.115909795
C,5.9999969145,-3.9587958282,-1.2378032438
C,6.1329836262,-5.0606531287,-0.1662531305
C,4.5516312704,-3.9789911843,-1.747788236
C,6.9210856732,-4.2775807529,-2.4333497103
H,7.1577850953,-5.1412844922,0.1938153193
H,5.4762241836,-4.8568848576,0.6861106297
H,5.837238915,-6.0239559627,-0.5944130683
H,4.381442842,-3.2476600107,-2.5444028654
H,4.3348490718,-4.9670813205,-2.1624374528
H,3.8300272142,-3.7985956091,-0.9435582634
H,6.6314458304,-5.2378800696,-2.8722386298
H,6.8289722939,-3.5123234156,-3.2110314925

H,7.9638311586,-4.3389113403,-2.1241770047
C,9.4867863897,-0.8614122565,1.0524002462
C,9.7230617026,-1.8406420619,2.2204744382
C,10.5548915368,-1.0817141435,-0.0389719344
C,9.6631317562,0.5639162272,1.595518002
H,8.9807524779,-1.6896425283,3.0112057992
H,9.6740387023,-2.8765477438,1.8867325331
H,10.7128718007,-1.6613586607,2.6525972785
H,10.4096554141,-0.3858709636,-0.871933343
H,11.5489763956,-0.8975812831,0.3812223315
H,10.5246024781,-2.1001915516,-0.424292238
H,10.6730638004,0.6670498454,2.0013338412
H,9.5486861034,1.3209278151,0.8128561518
H,8.9617815983,0.7894371508,2.4050034671
C,-5.2394127261,-1.3429639961,0.1325235323
C,-5.9415374619,-0.1450819601,-0.1631171033
C,-5.9625390846,-2.4683770115,0.60150631
C,-7.2983303191,-0.0323502551,-0.0280147557
H,-5.380554125,0.6943171592,-0.5543720226
C,-7.3200423727,-2.4489779471,0.7770090065
H,-5.4017603257,-3.3497124094,0.8749389868
C,-8.052083314,-1.2080839566,0.4540693314
O,-9.2869680413,-1.1526261147,0.5880519863
C,-8.0476032902,1.2561519805,-0.3749655935
C,-8.7794109743,1.787440072,0.8751931273
C,-7.0950378388,2.3578246111,-0.8611282434
C,-9.0658860814,0.9849222825,-1.501707248
H,-9.5206372854,1.0748769841,1.2352069437
H,-8.0696484509,1.9963310311,1.6823638815
H,-9.2881373674,2.7246100705,0.627628774
H,-6.562086699,2.0735278696,-1.7739263013
H,-7.6768568834,3.2539066583,-1.09221656
H,-6.3600238494,2.6379434885,-0.0999976636
H,-9.5768950746,1.9173789461,-1.762174419
H,-8.5618281482,0.6177602903,-2.4018044999
H,-9.8134590875,0.2541181157,-1.1952564108
C,-8.0886831827,-3.6602837717,1.3103804014
C,-8.8029493286,-3.2940481011,2.6275842344
C,-9.1244661173,-4.1248742684,0.2654707633
C,-7.1556425544,-4.8450923515,1.5996286497
H,-8.0811576612,-2.9722865585,3.3855334923
H,-9.5305086595,-2.4974398608,2.4764279489
H,-9.3262496222,-4.1738170451,3.0159750894
H,-8.6334568627,-4.4004556582,-0.6737685694
H,-9.6496130838,-5.0092842599,0.6406983532
H,-9.858345048,-3.3462464816,0.0611568368
H,-7.7504225086,-5.6824144413,1.974426724
H,-6.6376579597,-5.1918038107,0.6994210358
H,-6.4083705836,-4.6079859781,2.3634486088
C,-1.5872680158,-1.9423525521,-0.3035500294
H,-3.3662023491,0.6571755521,0.2230587049
C,4.9970687177,2.1952980907,0.0388382823
C,3.4769152122,4.1361938147,0.1221238883
C,6.1113158275,3.0614954803,0.0169029508
H,2.4731996342,4.5388722038,0.1529613603
C,5.8929025459,4.4179029598,0.0697210676
H,7.1210944558,2.6790195355,-0.0616094279
C,4.5824934664,4.9515413386,0.1314778617
H,6.7396954281,5.0960573325,0.0542689859
H,4.4524556466,6.0278617765,0.1738353895
C,-2.9084140498,-2.4837511908,-0.2384055712
C,-3.1177063267,-3.8625392398,-0.4524104403
C,-0.4897199774,-2.8013289955,-0.5288652893
C,-2.0315650615,-4.6747190983,-0.6743872164
H,-4.1163966012,-4.2806573253,-0.4631167668
C,-0.7200851085,-4.1448170676,-0.7014548601
H,0.5231607733,-2.4217679167,-0.5742426819
H,-2.1786397073,-5.7364981744,-0.8403495074
H,0.1168468355,-4.813151065,-0.8755034859
H,3.15320789,-0.4161620343,-0.0174479897

1c$_{NHNH}$-3 (triplet state)

–2637.3694671 hartree
C,-2.458347228,6.7042831363,-1.5783704686
C,-2.6931577463,6.9794447666,-0.2355642781
C,-1.8167387846,5.5206589517,-1.97458858
C,-2.2964452582,6.0827166083,0.7687190968
H,-3.191261281,7.902038701,0.0442848713
C,-1.4311589008,4.6466638488,-0.9810685991
H,-1.6280337509,5.2948915103,-3.0183406848
C,-1.6674097533,4.9236010933,0.3694867181
H,-2.4736866346,6.2856375769,1.8191355206
O,-0.7993319727,3.4464307137,-1.1056183747
O,-1.1934785666,3.9082409699,1.1456735625
C,1.4219334932,1.615735166,0.2312369959
C,-0.7080585792,0.5049687518,0.0912114009
C,0.6819619694,0.4660940182,-0.06805832
H,1.1778729234,-0.4426401035,-0.3784602547
B,-0.629487103,2.9436436881,0.2444993956
O,-1.3114205858,1.6175996411,0.3957670664
O,0.8243387045,2.7302774001,0.5402513848
C,-2.1133579141,-2.8207205362,-0.3669491479
C,4.9817504198,0.8624540118,0.1676385805
N,3.6518647614,0.5669230126,0.0734508118
C,3.7217272981,2.766413616,0.4577001972
C,-1.5647843708,-0.636645992,-0.0270091645
N,-1.1042520298,-1.9097709275,-0.2435484772
C,2.8528246449,1.6661507696,0.2578051405
H,-2.775140264,7.4144438897,-2.335291816
C,5.9870045471,-0.1479072323,0.0285261092
C,5.7034184765,-1.3530045357,-0.6705110654
C,7.272080911,0.0218189435,0.6061392466
C,6.6197305385,-2.3560204635,-0.8191954778
H,4.7383589589,-1.4484029831,-1.1530205142
C,8.2496915392,-0.9313493582,0.5219696355
H,7.4513987004,0.9215347363,1.1755137954
C,7.9553356568,-2.1788778928,-0.2107225825
O,8.8157982142,-3.0693249043,-0.315612386
C,6.3144327434,-3.6321204984,-1.6073090749
C,6.4475060506,-4.8623296789,-0.6859466358
C,4.8868780865,-3.6265482973,-2.1729036318
C,7.2873498861,-3.7619394081,-2.7973042812
H,7.4608786147,-4.9589289895,-0.2984336825
H,5.7534156016,-4.7935552543,0.1584306615
H,6.2014187877,-5.7684015653,-1.2490347925
H,4.72057815,-2.8003460285,-2.8716266751
H,4.717662247,-4.5565930442,-2.7218950903
H,4.1316312432,-3.5766999209,-1.3811201811
H,7.0448014033,-4.6622622456,-3.3710061041
H,7.1976466418,-2.9021643485,-3.4693426343

H,8.319977191,-3.8352672125,-2.4583901097
C,9.6213710482,-0.7492692145,1.1765273128
C,9.8592976054,-1.8589829762,2.2210965074
C,10.7255252641,-0.7987331226,0.1000196149
C,9.7338926957,0.6019503783,1.8970913907
H,9.0912415035,-1.8305006883,3.0010869944
H,9.8546761369,-2.8461383751,1.7602405619
H,10.8303678178,-1.704920272,2.702604664
H,10.5789833442,-0.0092207551,-0.6444935023
H,11.7009015198,-0.6378603801,0.5705219264
H,10.7409809371,-1.7616104677,-0.409203529
H,10.7286635023,0.685049583,2.3429507816
H,9.6137720205,1.4467935938,1.2111498705
H,9.0042445421,0.7028531894,2.7066785293
C,-1.8479554287,-4.2076810815,-0.6075421419
C,-0.5904592364,-4.7712010554,-0.2579273814
C,-2.8218906606,-5.037026479,-1.2217004019
C,-0.2796534439,-6.0836294268,-0.4789970373
H,0.1256832114,-4.1433647847,0.2580382692
C,-2.5983301779,-6.3587352107,-1.4949103433
H,-3.7507994214,-4.5789047846,-1.5265273036
C,-1.2948679142,-6.9437107064,-1.1230784648
O,-1.0514097774,-8.141528927,-1.347521465
C,1.0644035326,-6.6867523321,-0.0636295934
C,1.8001839175,-7.2327143221,-1.3048440318
C,1.9781878593,-5.6451919932,0.5981603229
C,0.8397220419,-7.8242034621,0.9538298223
H,1.2285483977,-8.0243680582,-1.7874737304
H,1.980122432,-6.4350054703,-2.0333500354
H,2.7719705221,-7.637587316,-1.0043990684
H,1.5432514614,-5.2343402127,1.5150027174
H,2.9229803493,-6.1225012955,0.8712373292
H,2.2177788216,-4.8190735456,-0.0800234271
H,1.8067776171,-8.2315837295,1.2659013131
H,0.3287152527,-7.4519680704,1.8478255638
H,0.2476531483,-8.6302170919,0.5221627905
C,-3.6463709663,-7.2346157672,-2.1859182104
C,-3.0875797995,-7.7747715026,-3.5182637679
C,-4.0338714658,-8.4110569497,-1.2659524619
C,-4.927250882,-6.4487478605,-2.5013362705
H,-2.8188683359,-6.9524887832,-4.1896215191
H,-2.2083428458,-8.3975483793,-3.3570700842
H,-3.8532426676,-8.3772419028,-4.0176212104
H,-4.4438697603,-8.0457599177,-0.3185507223
H,-4.8054418113,-9.0159768871,-1.7531483379
H,-3.1763550012,-9.0484674603,-1.0537165237
H,-5.6426124562,-7.1163965339,-2.9889406979
H,-5.4079427945,-6.0625700441,-1.5966115202
H,-4.7441205194,-5.612763182,-3.1836797899
C,-2.9774930538,-0.7240337486,0.025769976
H,-0.1352958646,-2.1467631663,-0.3854552163
C,5.0571173444,2.2692297084,0.3969806523
C,3.4736762004,4.142066433,0.6570909631
C,6.1434672808,3.165783158,0.4914112926
H,2.4574655382,4.5091599536,0.7095189207
C,5.8804277679,4.5009661608,0.6851597413
H,7.1660119215,2.8235814811,0.3936986352
C,4.5521034683,4.9843596902,0.7765933772
H,6.7046789971,5.2025092195,0.7602247808
H,4.3863603114,6.0454192062,0.9310654435
C,-3.3266912748,-2.090257752,-0.188099896
C,-4.6806388733,-2.4850466462,-0.1262963145
C,-3.9764477148,0.2476852525,0.2525921411
C,-5.6375364964,-1.5237534417,0.0967608733
H,-4.9700521498,-3.5230477207,-0.2308299564
C,-5.2870311556,-0.1630864832,0.2751548809
H,-3.7024802197,1.2819952922,0.4115572611
H,-6.6824762674,-1.8115321535,0.1465108945
H,-6.0721206972,0.5657989316,0.4470512616
H,3.3079445904,-0.3784928019,0.014234573

$1c_{NHNH}$·Cl⁻ (triplet state)

–3097.7359586 hartree
C,-2.4270357689,6.9259555576,-0.8382725855
C,-2.6929444869,7.017468618,0.523050455
C,-1.7766468078,5.802655033,-1.3754243637
C,-2.3192921699,5.9889040297,1.4042181493
H,-3.1968752203,7.8955459534,0.9152424947
C,-1.4130718671,4.7973571949,-0.5041286539
H,-1.5633579728,5.7190294203,-2.4356287782
C,-1.6815032423,4.8900329093,0.8691970107
H,-2.5206375817,6.0474075863,2.468503405
O,-0.7834728548,3.6306185973,-0.7777535626
O,-1.230095597,3.7839742441,1.509798125
C,1.3961641108,1.5826678733,0.451130286
C,-0.7180228975,0.5219838716,0.1094446671
C,0.6669272492,0.4798729254,0.0287465701
H,1.1703891392,-0.4267995899,-0.2719572599
B,-0.6376099223,2.9286429602,0.4988713893
O,-1.3458705153,1.6197056089,0.4526838901
O,0.8022205808,2.6974055855,0.7997994799
C,-2.0278336178,-2.7661812018,-0.6809405531
C,4.9183643573,0.7186253926,0.441821433
N,3.5877348004,0.4467396079,0.3557462707
C,3.7148559439,2.6373584913,0.8277403383
C,-1.5421622711,-0.6229261381,-0.1472952986
N,-1.0405391841,-1.8752191024,-0.392328367
C,2.825918641,1.568500396,0.5586923039
H,-2.7251609795,7.7333404771,-1.50012561
C,5.9439981876,-0.2734725784,0.2689469782
C,5.7143900597,-1.6422830474,0.5669230909
C,7.2234743973,0.1222559538,-0.20539263
C,6.7002519885,-2.5876969873,0.4596741701
H,4.7295710723,-1.9314241542,0.9109613507
C,8.2499319968,-0.7619024292,-0.3861016988
H,7.352468261,1.1574250846,-0.4850039642
C,8.0227384934,-2.1783243274,-0.0470745131
O,8.935609789,-3.0149147444,-0.1992713997
C,6.4667662325,-4.0508095649,0.848186042
C,7.4846587904,-4.4852645492,1.9231098929
C,5.0616197244,-4.2679510513,1.4249195764
C,6.6103982209,-4.9465108855,-0.399503849
H,8.5082610536,-4.4265779834,1.5547460089
H,7.3947033675,-3.8571975281,2.8162570828
H,7.2765395713,-5.5187895731,2.2209904882
H,4.269558126,-4.0059349385,0.7195254439
H,4.9445403934,-5.325506692,1.68079399
H,4.9054240723,-3.6898195405,2.3420017851
H,6.4296649836,-5.9908969806,-0.1222308686
H,5.8715944301,-4.6694776699,-1.1577513032

H,7.609028619,-4.8681774641,-0.8302487378
C,9.6084276971,-0.3290485534,-0.9460962927
C,10.7212590209,-0.6327511126,0.0777360159
C,9.8981148751,-1.0743052257,-2.2647526552
C,9.6507577706,1.1766967803,-1.2445259038
H,10.5416620281,-0.0959729062,1.0154729318
H,10.7770518774,-1.6997164837,0.2912770221
H,11.6875629194,-0.3019521388,-0.3193474536
H,9.1255489505,-0.8570665041,-3.0097309746
H,10.8605017208,-0.7435401773,-2.6712834184
H,9.9369935342,-2.1516313807,-2.1065161002
H,10.6388137033,1.4346208432,-1.637425765
H,8.9112416767,1.4701436571,-1.9959337792
H,9.4880699857,1.7798477,-0.3454390631
C,-1.7847557158,-4.1508850418,-0.9803157296
C,-0.655652952,-4.8380483472,-0.4625610093
C,-2.6985310906,-4.8554430283,-1.8093213504
C,-0.4434324299,-6.1715728567,-0.6947565773
H,0.0409239714,-4.2836573591,0.1532828436
C,-2.5387503171,-6.1743959473,-2.1300237896
H,-3.5134006847,-4.293786625,-2.2416766769
C,-1.3841581775,-6.8975952618,-1.5672571029
O,-1.2049212891,-8.1022295297,-1.8376537418
C,0.7390113011,-6.9243379948,-0.0772421022
C,1.6766842941,-7.4220360332,-1.1964835164
C,1.5579569592,-6.0257062629,0.8587079253
C,0.2327429503,-8.123074001,0.7516395256
H,1.1596243942,-8.104119451,-1.8720203796
H,2.0686520899,-6.5779745615,-1.7721925592
H,2.5286890166,-7.948308653,-0.7521745497
H,0.9604022074,-5.6688427222,1.7044312601
H,2.3897882255,-6.6072183271,1.2679783761
H,1.9783592702,-5.1555668672,0.3490852633
H,1.0864790845,-8.623898238,1.2212460399
H,-0.435454909,-7.7848473358,1.5512550975
H,-0.2988215083,-8.8445407277,0.1321038463
C,-3.5046207983,-6.9076241752,-3.0659361421
C,-2.7492656058,-7.4197622779,-4.309051569
C,-4.1546182909,-8.0951959906,-2.3269911606
C,-4.6350121416,-5.9902076815,-3.5544879998
H,-2.2943649889,-6.5871009303,-4.8556636166
H,-1.9655891142,-8.1234567472,-4.0300403935
H,-3.4499634832,-7.9233146446,-4.9845636861
H,-4.7120345872,-7.7476110292,-1.4505165203
H,-4.8599508399,-8.6030073448,-2.9944343317
H,-3.4026338341,-8.8128830274,-2.0005367988
H,-5.2964366281,-6.5597872401,-4.2142200031
H,-5.2443730694,-5.6108207277,-2.7278322081
H,-4.256977102,-5.1364362872,-4.1252523516
C,-2.9519252494,-0.707288634,-0.2498188638
H,-0.0324280286,-2.1075351919,-0.4105368932
C,5.0346278704,2.1101639105,0.7525446787
C,3.4977139288,3.997459733,1.1384462336
C,6.1361771699,2.9422376985,1.0479719152
H,2.4892875076,4.3868678659,1.1874543686
C,5.9055383747,4.2636085878,1.3532309329
H,7.1466583199,2.5527327203,1.058344291
C,4.5916890278,4.791453169,1.3864881636
H,6.7442828668,4.9134628051,1.5828009551
H,4.4482192051,5.8398601095,1.6285550196
C,-3.2648236705,-2.0535705831,-0.5889415438
C,-4.6154846407,-2.4517607104,-0.6899297959
C,-3.9758124889,0.2480707914,-0.0694963855
C,-5.5999464378,-1.5085861838,-0.5065002122
H,-4.883324579,-3.4825933899,-0.8862491327
C,-5.2803009244,-0.1612374034,-0.2091563271
H,-3.7240746867,1.2696971899,0.1832294994
H,-6.6423392975,-1.8024697702,-0.5810297025
H,-6.08370364,0.5560782965,-0.0737295011
H,3.2050137591,-0.4833138179,0.1126639234
Cl,2.2004567006,-2.4484085357,-0.3700729903

1c$_{NHOH}$-1 (closed-shell singlet state)
–2637.385555 hartree
C,-2.4852358834,6.9799356907,-0.2766908985
C,-2.7865080019,7.0260299497,1.0800200374
C,-1.7961086243,5.888775196,-0.8294339387
C,-2.4115109711,5.9830062919,1.9418317423
H,-3.3169377048,7.8825177712,1.4836632582
C,-1.4325134955,4.8695908252,0.0244305594
H,-1.5511188807,5.843272004,-1.8849697783
C,-1.7357588551,4.9163557024,1.3900343012
H,-2.6365430014,6.0091692756,3.0023883492
O,-0.7684405731,3.7184962302,-0.2648751626
O,-1.2728993049,3.7969506249,2.0106366245
C,1.3613971514,1.6284438327,0.9383493988
C,-0.7449469111,0.5882265533,0.4695709644
C,0.6662996126,0.5385525502,0.4624020141
H,1.1900534079,-0.3334209079,0.1090009654
B,-0.648337641,2.9979487752,0.99013948
O,-1.3524968039,1.6702695113,0.8792727702
O,0.7805584609,2.7267061652,1.3121148325
C,-3.6797500831,-1.3316396931,-0.1980075516
C,4.8747739022,2.4040397325,0.959068938
N,3.5098064668,2.7051108938,0.8484555624
C,3.6996607913,0.4719994368,1.3566897765
C,-1.6002330013,-0.4731993899,0.0987284482
N,-2.964632052,-0.2596910581,0.1642043115
C,2.8345629381,1.619145269,1.0483676176
H,-2.7828138319,7.8008273932,-0.9212756162
C,5.7933876357,3.400444121,0.7421040448
C,5.3252484252,4.745204544,0.4580185185
C,7.2216120032,3.171054485,0.7778772163
C,6.1588006701,5.7838501648,0.2492920532
H,4.2527660255,4.8694247293,0.4312580302
C,8.1270326879,4.1492676173,0.5764148066
H,7.5613603369,2.1659477779,0.9621749982
C,7.6301903703,5.5381190245,0.3063280131
O,8.4286798865,6.4533581611,0.1325321127
C,5.6508088563,7.1989087936,-0.0393456438
C,6.1583179948,7.6703462391,-1.4173590401
C,4.1169318783,7.2491283803,-0.0620750715
C,6.1379402316,8.1677582399,1.0573856105
H,7.2464742442,7.719812643,-1.4498329233
H,5.8119015301,6.9980040221,-2.2091650353
H,5.7605915458,8.668021027,-1.6297581821
H,3.6787953471,6.9597362676,0.8975845753
H,3.7978312315,8.2736123819,-0.2726362998
H,3.693466144,6.6065604162,-0.8396809687
H,5.7405787273,9.168910237,0.8612329003

H,5.7762045104,7.8527386901,2.0416327484
H,7.225615147,8.2286314999,1.0858993074
C,9.6355017177,3.894184869,0.610815894
C,10.2640719785,4.257450373,-0.7501599922
C,10.2864173894,4.7330046727,1.7299419724
C,9.9523547706,2.4182322821,0.8911278999
H,9.8194961955,3.6631710292,-1.5552766985
H,10.1324327893,5.3138032994,-0.9813120704
H,11.3366772645,4.0392015704,-0.7261188008
H,9.8579347705,4.47970723,2.7052381709
H,11.3592196282,4.5178412414,1.7694547921
H,10.1545366747,5.8002651447,1.5565012216
H,11.0372563587,2.2834469449,0.90494207
H,9.5712349245,2.0930281956,1.8647345332
H,9.5507771121,1.7558383158,0.1173735223
C,-5.1417829157,-1.3197369067,-0.2177319716
C,-5.8307960292,-0.1703630131,-0.60215286
C,-5.8721163459,-2.4456864556,0.1652778003
C,-7.2217406097,-0.1074982907,-0.6132150688
H,-5.2588170993,0.6874172202,-0.9303984396
C,-7.2620807505,-2.4570445142,0.1773641005
H,-5.3295194545,-3.3210746608,0.4927188249
C,-7.9171494064,-1.2702427765,-0.2172822403
O,-9.281701683,-1.3083390253,-0.197684434
C,-7.9453859002,1.1827143112,-1.0560748908
C,-8.7990204454,1.7583328187,0.0970969153
C,-6.9517615797,2.2913271306,-1.4405305109
C,-8.8077514043,0.9254711945,-2.312613521
H,-9.5874897958,1.0951085347,0.4642812559
H,-8.164672127,1.9941900137,0.9557881481
H,-9.2847694156,2.6829873606,-0.228200605
H,-6.3160694902,2.0015231173,-2.281546995
H,-7.5101490804,3.1802245899,-1.7455930567
H,-6.3119431473,2.582270647,-0.6031386471
H,-9.3013085138,1.8524712728,-2.6193230511
H,-8.1772595652,0.588583164,-3.140042404
H,-9.588141687,0.1703639629,-2.185862093
C,-8.0421239785,-3.7106812318,0.6162430895
C,-8.904147588,-3.3995881503,1.8560781474
C,-8.9304941507,-4.2184976851,-0.5371892612
C,-7.0979626415,-4.8608667631,0.9994048997
H,-8.2760439341,-3.0674386304,2.68882686
H,-9.6452269346,-2.6273852907,1.6535422452
H,-9.4311415969,-4.3050144322,2.1742590731
H,-8.3212370726,-4.4707697551,-1.4111348669
H,-9.457698419,-5.1253102417,-0.2233545451
H,-9.6722775327,-3.4790788552,-0.8363396362
H,-7.6972356763,-5.7250031521,1.2987344363
H,-6.4680660689,-5.1763475919,0.1618125532
H,-6.4518001353,-4.6038508859,1.8441470647
C,-1.4340806374,-1.8077124146,-0.3536076044
H,-3.3400985973,0.6168555586,0.5054224028
H,-9.6484246591,-0.4570507406,-0.450724757
C,5.0162321968,0.9729075317,1.3002215244
C,3.4337104088,-0.8440293574,1.7153745529
C,6.0810899974,0.1211235825,1.5894333053
H,2.4165594669,-1.2087608424,1.8013385725
C,5.8150225768,-1.2042519133,1.9257378425
H,7.1101390403,0.4540134197,1.5768559311
C,4.5073938151,-1.6844107728,1.9938056709
H,6.6423473077,-1.8688718545,2.1520799202
H,4.3258912371,-2.7157083689,2.2776905136
C,-2.7445330126,-2.3449406476,-0.5421436959
C,-2.9239476233,-3.6518454072,-1.0429094102
C,-0.3103612884,-2.6131210655,-0.6433121888
C,-1.8136695546,-4.4117543755,-1.3158975436
H,-3.919551879,-4.0422334,-1.2212176338
C,-0.5126712464,-3.8907633389,-1.1082145284
H,0.6992393297,-2.2426840934,-0.5158739222
H,-1.9269662053,-5.4186064468,-1.7030918445
H,0.3458809077,-4.5149966196,-1.3344789647

1c$_{NHOH}$-2 (closed-shell singlet state)

–2637.3952641 hartree

C,-2.3837249423,6.8883541418,-1.7433593039
C,-2.6560397411,7.1344057987,-0.4021734273
C,-1.7272774603,5.7146203136,-2.14636062
C,-2.2833252196,6.2170536274,0.5932595876
H,-3.162818645,8.0504565098,-0.1161153634
C,-1.3657961067,4.8196331708,-1.1621010467
H,-1.5066807475,5.5128736538,-3.1888886935
C,-1.6397324476,5.0678236936,0.1879210215
H,-2.487790371,6.3983262383,1.6427973248
O,-0.7287649419,3.6256710805,-1.2955307122
O,-1.1836792203,4.0397001054,0.9545568847
C,1.3882019197,1.6771314061,0.1700829318
C,-0.7406651084,0.6225775605,-0.0827948812
C,0.6673803796,0.5435144811,-0.113149727
H,1.1884721192,-0.382178947,-0.2885992064
B,-0.6004091698,3.0832708984,0.0478887405
O,-1.3249696907,1.7733674012,0.1422300728
O,0.8327712691,2.8396432776,0.3809205609
C,-3.711102768,-1.3375493622,-0.3608583632
C,4.8770763678,0.8064662039,0.2745397836
N,3.510382129,0.5384279388,0.1187705465
C,3.7389244724,2.7821829965,0.5703629007
C,-1.6172732449,-0.4709990895,-0.2444933464
N,-2.9781278738,-0.22839486,-0.2037453942
C,2.8557624022,1.6478370777,0.2821585797
H,-2.679832775,7.614525262,-2.4935912852
C,5.77855881,-0.2218688454,0.1452995043
C,5.2956027429,-1.5579645866,-0.1492347647
C,7.2053385157,-0.0378470211,0.2903574079
C,6.1104511956,-2.622654491,-0.2893683357
H,4.225568459,-1.6540350451,-0.251725492
C,8.0942731742,-1.0449921082,0.1669412069
H,7.5602451105,0.9551516383,0.506865455
C,7.5806137123,-2.4187147438,-0.1340097963
O,8.3605842266,-3.3597667762,-0.2503129599
C,5.5840170367,-4.0264774929,-0.6002786193
C,5.9690377489,-4.9988201504,0.5330896974
C,4.0536550522,-4.0364866566,-0.7243216071
C,6.1662935645,-4.5251445128,-1.938379998
H,7.0503542973,-5.086467857,0.6340702043
H,5.5523566377,-4.6655055595,1.4892632931
H,5.5605255013,-5.9917180174,0.3178959301
H,3.7033322452,-3.3911035427,-1.5359868611
H,3.7229292005,-5.0551231965,-0.9464581433
H,3.5647824407,-3.7253960234,0.2043295078
H,5.7588576047,-5.5151254214,-2.1684892029

H,5.8914092497,-3.8516496637,-2.7567897168
H,7.2524251005,-4.6014718967,-1.8977237805
C,9.6021244205,-0.8343314754,0.3233622489
C,10.1354964554,-1.6881386063,1.4920964467
C,10.3271755025,-1.2208538227,-0.9821312728
C,9.9389544365,0.6326348571,0.6263299298
H,9.6383685129,-1.4181034431,2.4296074049
H,9.9858539756,-2.7517264465,1.3101390281
H,11.207717198,-1.505588639,1.6173374918
H,9.9674271833,-0.6156784709,-1.8207675064
H,11.4004637579,-1.0350418442,-0.8717293378
H,10.1825144214,-2.2734757336,-1.2224650083
H,11.0225975435,0.7351651676,0.7289856222
H,9.6227912092,1.3031368661,-0.1793937738
H,9.4888453989,0.9739042554,1.5642400762
C,-5.1728212205,-1.3088218013,-0.3675893121
C,-5.853703535,-0.2376329011,-0.9443314737
C,-5.9110607301,-2.3386058091,0.217761372
C,-7.2439508663,-0.1599713148,-0.9532709899
H,-5.2761099196,0.5402380251,-1.4259929492
C,-7.3007601699,-2.3297342499,0.2449816454
H,-5.3742538292,-3.1482543481,0.6912033121
C,-7.9474018749,-1.2245383429,-0.3497652576
O,-9.3120404646,-1.2415857109,-0.311663852
C,-7.9587761183,1.0395934863,-1.6124421517
C,-8.7939106502,1.8215933,-0.5727645388
C,-6.9577196865,2.049939411,-2.1965607146
C,-8.837065905,0.5736717785,-2.7957231336
H,-9.5856775268,1.2440467748,-0.0871855568
H,-8.1480078323,2.1977580705,0.2251802366
H,-9.2728009474,2.6800215917,-1.0529432226
H,-6.3342514449,1.6073280237,-2.9780794243
H,-7.5099499723,2.8773218565,-2.6499303714
H,-6.305935817,2.4767526885,-1.4295649478
H,-9.3242636188,1.4372710884,-3.2581892513
H,-8.2189411166,0.0873757353,-3.5553852987
H,-9.6238544806,-0.1374039414,-2.5300124616
C,-8.089311634,-3.4747474577,0.9082218382
C,-8.9351122975,-2.9359543324,2.0792700508
C,-8.994845928,-4.1701095333,-0.1280740472
C,-7.1532923162,-4.5489831525,1.4836259676
H,-8.2949330892,-2.4679930557,2.8339075222
H,-9.6699494333,-2.2033684401,1.7478111224
H,-9.4684301975,-3.7632292956,2.5587815551
H,-8.3972207146,-4.5829201759,-0.9472410701
H,-9.528790452,-4.9990932504,0.3477729106
H,-9.7314501136,-3.4871016367,-0.5492703699
H,-7.7584232586,-5.3378605412,1.9385369353
H,-6.535777962,-5.017568487,0.7110320907
H,-6.4953461869,-4.1525348621,2.2627796558
C,-1.4743504002,-1.8687244082,-0.4491924262
H,-3.3386421523,0.6998255215,-0.0198931745
H,-9.6724879689,-0.4458922337,-0.7117762802
C,5.0429370474,2.2472558233,0.5666908134
C,3.4929497842,4.1281724612,0.8130344475
C,6.1236477875,3.0915831557,0.8131292248
H,2.4814401229,4.5122399609,0.8111275309
C,5.8801419996,4.442055377,1.0565999131
H,7.1470704163,2.7419988028,0.8234760496
C,4.5842402753,4.9568646294,1.0574317917
H,6.7192037456,5.1029232126,1.2487273181
H,4.4244932014,6.0125291122,1.2497635502
C,-2.793562348,-2.4105051138,-0.5266329807
C,-2.9962466976,-3.7834100678,-0.7816358026
C,-0.3641824956,-2.7296452633,-0.5913998846
C,-1.8987138674,-4.5965850409,-0.9200754571
H,-3.9986707706,-4.1847387511,-0.8789558137
C,-0.5888899039,-4.0671401556,-0.815793347
H,0.6510230527,-2.358573373,-0.5294066647
H,-2.0293962647,-5.6551371518,-1.1174809032
H,0.2592526095,-4.7353964809,-0.9253668878

1c$_{NHOH}$-3 (closed-shell singlet state)

–2637.3790923 hartree

C,-2.8214649059,6.6234746757,-3.186438322
C,-2.9959737248,7.0854287145,-1.8866309726
C,-2.1322517427,5.42805022,-3.4442864239
C,-2.4888239273,6.371816528,-0.789238051
H,-3.5314316834,8.0134529501,-1.7138129497
C,-1.6379683436,4.7349621493,-2.3605319086
H,-1.9885824502,5.0580098951,-4.4535349604
C,-1.8142513861,5.1996119892,-1.0525535851
H,-2.6164650361,6.7223487883,0.2290104876
O,-0.9434717721,3.564275895,-2.3479457792
O,-1.2383325623,4.3391729194,-0.1680590222
C,1.4047066173,2.0255653129,-0.8670050066
C,-0.669258532,0.8313982926,-0.690522982
C,0.7309043846,0.8269135531,-0.9250518314
H,1.2873956505,-0.0937166316,-1.0178779667
B,-0.6701128288,3.2782130882,-0.9511006386
O,-1.2953089501,1.9620739031,-0.5681389395
O,0.8029013923,3.1638826601,-0.7234456704
C,-1.8592142512,-2.5679419354,-0.4087532369
C,4.9066525887,2.6917779139,-0.3444706833
N,3.5329691154,2.908226744,-0.1532915328
C,3.7732522054,1.2278109037,-1.7019330086
C,-1.4456021551,-0.3375034661,-0.5302847875
N,-0.9180760049,-1.6200647005,-0.5726720109
C,2.8823833888,2.074468442,-0.8976942058
H,-3.2224230777,7.1943649318,-4.0177976152
C,5.8045376097,3.4398777665,0.374005323
C,5.3114591941,4.4698672922,1.2709395012
C,7.2358416057,3.2474233411,0.2818429897
C,6.1250646868,5.257640086,2.0021221198
H,4.2375755096,4.5768832637,1.31221528
C,8.1220856799,3.9859091278,0.979215667
H,7.5939414863,2.4671703738,-0.3682255509
C,7.6000532443,5.0565281019,1.8899011807
O,8.3809271709,5.7510937537,2.5327712745
C,5.5916396149,6.3476896192,2.9354448852
C,6.1147434326,7.7276734335,2.4873454293
C,4.0577513188,6.3990750489,2.9165799721
C,6.0358844814,6.0648859814,4.3850606816
H,7.2019503355,7.7814431496,2.5385619541
H,5.7984088149,7.948387272,1.4626764225
H,5.6993887789,8.5035162993,3.1386467412
H,3.6088526305,5.4601953921,3.2544134041
H,3.7203000124,7.1875224941,3.5950021188
H,3.6632347594,6.6297338422,1.9227059867
H,5.6195176273,6.82930324,5.0491085362

H,5.663545196,5.0918348249,4.7222190784
H,7.1211258462,6.0784869447,4.482827555
C,9.6337189126,3.7701250017,0.8776951515
C,10.3154412576,5.0524507739,0.3574817517
C,10.2080139792,3.3944648369,2.2591013942
C,9.9777709768,2.6329341596,-0.0948655249
H,9.9256822256,5.3280779858,-0.6279186161
H,10.1653517166,5.8885985556,1.0392521667
H,11.3913378302,4.8773012964,0.2550355013
H,9.7416936126,2.4792841412,2.6387481275
H,11.283377786,3.209693954,2.1688514437
H,10.0551347781,4.1921735504,2.9849818312
H,11.0639890065,2.5150081474,-0.1347196479
H,9.5567667892,1.6743278535,0.2256069493
H,9.635365742,2.8444400235,-1.113160469
C,-1.5299049322,-3.9935747271,-0.4122977491
C,-0.3674076759,-4.4561896316,0.2019114004
C,-2.3699135216,-4.9138264262,-1.041253451
C,-0.0152020173,-5.8044308705,0.2053823137
H,0.2572432862,-3.7428923129,0.7237580378
C,-2.0838529445,-6.2736042669,-1.0773151552
H,-3.2550172965,-4.5406261983,-1.5367832906
C,-0.8948422525,-6.6965661716,-0.4440423133
O,-0.6391559121,-8.0371812814,-0.4924499332
C,1.2739770437,-6.2779619809,0.9110719539
C,2.2464048385,-6.9307314595,-0.0978287671
C,2.0432964372,-5.1058958588,1.5422894891
C,0.9435242876,-7.2444581681,2.0711764632
H,1.8517432059,-7.8095914216,-0.6147303902
H,2.5243912753,-6.2120919025,-0.8737819774
H,3.1598330322,-7.2438313025,0.416561627
H,1.4556845628,-4.5916484833,2.3075337147
H,2.9447610148,-5.4905450423,2.0266269633
H,2.3624579394,-4.3737888413,0.7951282626
H,1.8665730403,-7.5578262495,2.5679853512
H,0.3137702633,-6.7436675813,2.8115118724
H,0.4132432723,-8.1530856964,1.77396157
C,-3.0256995247,-7.2638396036,-1.7880195703
C,-2.2880438887,-7.9712013334,-2.9424032353
C,-3.5654442969,-8.3021447477,-0.7839347806
C,-4.2419593797,-6.5502355981,-2.3983705442
H,-1.9291287766,-7.241335436,-3.6751462548
H,-1.4373228851,-8.5518972999,-2.5879194366
H,-2.9758346883,-8.6504474937,-3.4563335602
H,-4.116888503,-7.8084221758,0.0226762377
H,-4.2557642839,-8.9817615676,-1.2939661393
H,-2.7665294282,-8.8962518423,-0.3420481307
H,-4.8787478191,-7.2928166191,-2.88661703
H,-4.8509656542,-6.0473782644,-1.640914272
H,-3.955475046,-5.8167022334,-3.1579344314
C,-2.83454825,-0.4968939851,-0.3111359043
H,0.048486454,-1.837417091,-0.7635273711
H,0.1829735257,-8.2438023012,-0.0406338724
C,5.0799082726,1.6205923806,-1.3487451142
C,3.5341029593,0.2718324711,-2.6814697836
C,6.1642164691,1.0161381282,-1.9830352721
H,2.5235781311,0.0160393618,-2.980410311
C,5.9253908914,0.0398038414,-2.9478591168
H,7.1885594072,1.2871634565,-1.7657616087
C,4.6268233528,-0.327964499,-3.3002707464
H,6.7682761193,-0.4297114329,-3.4444114388
H,4.4686600265,-1.0740647573,-4.0719366178
C,-3.0936237432,-1.8971323091,-0.2355016699
C,-4.3996487357,-2.3725778605,0.0227446173
C,-3.8903450329,0.4290577026,-0.1570118018
C,-5.4092253935,-1.4555092284,0.168702181
H,-4.5953553933,-3.435126576,0.1148242547
C,-5.1523191873,-0.0621351941,0.0716203975
H,-3.6955867284,1.4920242737,-0.2148742279
H,-6.4206974513,-1.7933095838,0.3684112134
H,-5.9780262635,0.6319025672,0.1914965219

1c$_{NHOH}$-4 (closed-shell singlet state)
–2637.3900793 hartree
C,-2.3923011175,6.7939233429,-1.4752316025
C,-2.6599343347,7.0217770365,-0.1299083439
C,-1.7310979455,5.6293092724,-1.8958338876
C,-2.2776231257,6.0944274807,0.8523606576
H,-3.1727857131,7.9301553764,0.1694481925
C,-1.3596671881,4.7248866372,-0.9242739663
H,-1.5168582074,5.4403281638,-2.9420048541
C,-1.6290370787,4.9545058653,0.4295923467
H,-2.4807636314,6.2600884433,1.90471399
O,-0.7156302718,3.5360620668,-1.0752273688
O,-1.1631387661,3.9190078892,1.1816122335
C,1.4506667804,1.6282236397,0.3192145052
C,-0.6657839666,0.5296617246,0.0962179141
C,0.7496070082,0.4882179813,0.0193413326
H,1.3009827306,-0.4145143025,-0.1963623314
B,-0.5732973641,2.9833465356,0.2603881571
O,-1.2670561011,1.6566289149,0.3468523632
O,0.8711356452,2.766628749,0.5825312716
C,-1.9906363681,-2.79691784,-0.3612605728
C,4.9718622445,0.8784777106,0.2693652688
N,3.6116483523,0.5747946063,0.1102539928
C,3.7693524249,2.7699360239,0.7817655371
C,-1.4933773744,-0.6044187233,-0.0399847307
N,-1.0065042331,-1.8871265469,-0.2501100839
C,2.9217977482,1.6368051791,0.3970667842
H,-2.6983382058,7.5264194036,-2.2152275784
C,5.9100618117,-0.0914882765,0.0145778223
C,5.4773409992,-1.397213907,-0.4463630819
C,7.32975114,0.1269125291,0.1795163544
C,6.3328829449,-2.3996525161,-0.7303981149
H,4.4111616721,-1.5200248186,-0.5608162247
C,8.2564168765,-0.8204009288,-0.0737623996
H,7.647601557,1.0944766598,0.5280335235
C,7.7948417959,-2.1613712461,-0.5503438821
O,8.6085581545,-3.050227889,-0.7872384912
C,5.8615897789,-3.7673893,-1.2318958231
C,6.2877521667,-4.8707090532,-0.2424342521
C,4.3328633123,-3.8202164446,-1.3605999651
C,6.4582103292,-4.0545571737,-2.6246677478
H,7.3719190913,-4.9270188966,-0.1508120682
H,5.8608204147,-4.6889041662,0.7495695339
H,5.9201535286,-5.8404896094,-0.5943584096
H,3.9541542222,-3.0818967281,-2.0740603864
H,4.0412975221,-4.8095811768,-1.7245846051
H,3.8364738441,-3.6603151864,-0.397805104
H,6.0901441165,-5.0191403463,-2.9896536584

H,6.154179483,-3.2865818146,-3.3433676841
H,7.5465741237,-4.0919700122,-2.5922470388
C,9.7557765289,-0.5736928526,0.11012107
C,10.3268313386,-1.5503958659,1.1585683794
C,10.4893669064,-0.7593550713,-1.2340312179
C,10.0372077371,0.8535273807,0.6009971451
H,9.8243026593,-1.4231103222,2.1230872526
H,10.2172402383,-2.5864997231,0.8403546891
H,11.3918667474,-1.3445531906,1.3067422537
H,10.1029819123,-0.064859444,-1.9872226486
H,11.5552041912,-0.5481516496,-1.0999193627
H,10.3842594102,-1.7766969144,-1.6091325884
H,11.1164952026,0.9837989833,0.7169107367
H,9.6922557327,1.6099955969,-0.1113939145
H,9.5783510067,1.0529561353,1.5748486435
C,-1.7167430017,-4.2178167321,-0.5766159164
C,-0.6664130617,-4.8489115755,0.0875797808
C,-2.5035610066,-4.9646435474,-1.4548861439
C,-0.3767587517,-6.1994044953,-0.0966476258
H,-0.0865329167,-4.272994367,0.7970126397
C,-2.2711810871,-6.3143301274,-1.6918241572
H,-3.2989826397,-4.4583642055,-1.9833710036
C,-1.1971102047,-6.9121298641,-0.9970493924
O,-0.9944768964,-8.2396925683,-1.2435790032
C,0.7786410912,-6.8686605276,0.6789145451
C,1.8542706875,-7.4111930907,-0.2897147473
C,1.502670497,-5.8707894783,1.5975816884
C,0.2442375412,-7.988095166,1.6009737525
H,1.5018035744,-8.1703294463,-0.9935505998
H,2.2663509925,-6.596246897,-0.8908083734
H,2.6738350598,-7.8592175424,0.2798833486
H,0.8359197757,-5.4500491236,2.3551076689
H,2.308320215,-6.3900081935,2.1234015802
H,1.9572417362,-5.0503393509,1.0354135602
H,1.0732534552,-8.4436559158,2.1507965091
H,-0.4577075618,-7.5732943576,2.329530955
H,-0.2822417061,-8.7940527789,1.0831709061
C,-3.1480985133,-7.1096956457,-2.6772301835
C,-2.2905260506,-7.6598300755,-3.834381517
C,-3.8612814445,-8.2646775969,-1.9458043698
C,-4.2398941483,-6.227967056,-3.3030483345
H,-1.8054868995,-6.8422860839,-4.377255406
H,-1.5205763147,-8.3448689242,-3.4815755752
H,-2.9297752279,-8.1981584321,-4.5415865173
H,-4.4963271709,-7.8785953462,-1.1419470737
H,-4.5033556061,-8.8041048368,-2.6496211369
H,-3.1548020993,-8.9739616259,-1.5164777755
H,-4.8342370476,-6.8362379608,-3.9901507933
H,-4.9251767446,-5.8213005764,-2.5530057832
H,-3.8229126417,-5.3973797196,-3.8803994884
C,-2.903888621,-0.7253083298,-0.0067110708
H,-0.0311141359,-2.1156276492,-0.3737294292
H,-0.2370642285,-8.5657378817,-0.7507036171
C,5.0911087354,2.2876268419,0.7044878116
C,3.4793084673,4.0752707672,1.159864166
C,6.1455310251,3.1450765989,1.0114859814
H,2.4548485683,4.4197155464,1.2093902474
C,5.8581028494,4.4547682167,1.3920875502
H,7.1813260484,2.8376948828,0.964624845
C,4.5447981363,4.9166974064,1.4672546641
H,6.6765478634,5.1256359244,1.6323601918
H,4.3507317732,5.9416561795,1.7650193586
C,-3.2152875066,-2.1013204001,-0.2091606477
C,-4.5564352282,-2.5455441844,-0.1848163675
C,-3.9395640356,0.2140388023,0.191446301
C,-5.5467158116,-1.6157245725,0.0092638345
H,-4.7951555182,-3.5964220741,-0.3050931121
C,-5.2353998914,-0.2424229967,0.1891649505
H,-3.7053692098,1.2595034353,0.3438801617
H,-6.5849637915,-1.9296372705,0.0329569958
H,-6.046565869,0.4631156168,0.3378936459

1c$_{NHOH}$´-1 (open-shell singlet state)
–2637.3671885 hartree
C,2.540888219,6.4987157346,2.8957500343
C,2.7857720984,6.9753928921,1.6126704004
C,1.8726176714,5.2814199485,3.1013740257
C,2.3730842701,6.2552078774,0.4804786602
H,3.3027875234,7.9203699943,1.4804098139
C,1.4715131158,4.5817986868,1.9836946618
H,1.673291092,4.900465169,4.0970331411
C,1.7184610227,5.0613913862,0.6925481093
H,2.5554953315,6.6175700755,-0.5252327635
O,0.8156907653,3.3909065339,1.9191854057
O,1.2284100767,4.1906072161,-0.2323285237
C,-1.3762003802,1.7691858608,0.2514138253
C,0.7488629652,0.6599068713,0.226949271
C,-0.6431513254,0.5872289497,0.3329513559
H,-1.1438276895,-0.3639486538,0.3571915396
B,0.6459917889,3.1039058755,0.5055558329
O,1.3552676224,1.8159449279,0.1643375405
O,-0.7933244243,2.9253399835,0.1723132364
C,3.7105396505,-1.3554428126,-0.0149699808
C,-4.7844758304,2.5122723685,-0.4138180204
N,-3.468269786,2.7895187857,-0.3960291167
C,-3.7664643441,0.7358361568,0.6176384567
C,1.6229380641,-0.4647886236,0.1450613996
N,2.9656443115,-0.2239473614,0.0945732157
C,-2.8269880995,1.7633595541,0.1688698208
H,2.8687654099,7.0753762684,3.7546847728
C,-5.706555863,3.44083433,-1.0262798009
C,-5.2740917358,4.7512915236,-1.2905010812
C,-7.0080549206,3.082150272,-1.3965442098
C,-6.094341934,5.7008955705,-1.8738463428
H,-4.2595048706,4.9952913111,-1.0115785974
C,-7.8836971477,3.9792870613,-1.9957797606
H,-7.3244139214,2.0626081437,-1.2467159973
C,-7.4062018371,5.2925547416,-2.2152504734
O,-8.1962391335,6.2449029747,-2.7838737018
C,-5.5914032557,7.1329004415,-2.1352486655
C,-6.4439044724,8.152047927,-1.3531641806
C,-4.1360694257,7.308929514,-1.6756058737
C,-5.6344067107,7.4483874594,-3.6439017717
H,-7.4888245793,8.1352840043,-1.6616499626
H,-6.3955479649,7.9497971872,-0.278406632
H,-6.0539626916,9.1613087628,-1.5206896264
H,-3.4494808841,6.6422218554,-2.2048617089
H,-3.8221434731,8.3356222628,-1.8833360001
H,-4.017283693,7.1413052565,-0.6013478328
H,-5.2429422504,8.4557686788,-3.8189166998

H,-5.0079137672,6.7451859499,-4.2017847349
H,-6.646936343,7.4025689098,-4.0439620218
C,-9.3051421252,3.5376560545,-2.4061563083
C,-10.3786900322,4.3402377627,-1.6355903774
C,-9.5041672567,3.6666338085,-3.9334872966
C,-9.5614741416,2.0597287984,-2.0684747782
H,-10.2609060321,4.191007618,-0.5587982389
H,-10.3564315731,5.4206583088,-1.8034579962
H,-11.3769384083,3.993680099,-1.919677337
H,-8.7849998592,3.0345154539,-4.4615146504
H,-10.5110543693,3.3368528914,-4.207186377
H,-9.3777633283,4.6786163774,-4.3270762823
H,-10.5751861603,1.7929868032,-2.3796424934
H,-8.8713880654,1.3927126392,-2.59223156
H,-9.4878723998,1.8664390704,-0.9944768438
C,5.1371764306,-1.3171350395,-0.0911354491
C,5.8483744027,-0.1689769033,0.3583933992
C,5.8688025123,-2.4032954616,-0.6423980275
C,7.2093716828,-0.0729649854,0.3022556736
H,5.2858363234,0.6401558876,0.8064331824
C,7.2309389671,-2.3946651205,-0.7505318296
H,5.3104950154,-3.2383958609,-1.0367753825
C,7.9687363531,-1.210716294,-0.261389882
O,9.2077286429,-1.1709164348,-0.3250639774
C,7.9621988959,1.1566543123,0.8160031055
C,8.767779725,1.7942463646,-0.3351139313
C,7.0050210656,2.2253050407,1.3629072273
C,8.9149822126,0.7501460058,1.9591252721
H,9.5145489089,1.1051783788,-0.7279637203
H,8.1050413376,2.0994706105,-1.1515123145
H,9.2790245102,2.6899920035,0.0318265911
H,6.4209601254,1.8637447225,2.2150992761
H,7.589369863,3.0806946692,1.7117276725
H,6.3151496481,2.5965309245,0.5985376989
H,9.4279371167,1.6397580097,2.3385052895
H,8.3578807233,0.3060375286,2.7906740951
H,9.6647539719,0.0370079955,1.6182580928
C,8.0054840418,-3.5584028627,-1.3741716593
C,8.7927489975,-3.0688169956,-2.6068727292
C,8.9778175358,-4.1564030535,-0.3362136232
C,7.068490533,-4.683768005,-1.8362960958
H,8.116443393,-2.6516174922,-3.3602443857
H,9.5247583548,-2.3093232725,-2.3346046148
H,9.3208675455,-3.9130213754,-3.0620170747
H,8.4341269718,-4.520921467,0.5417063178
H,9.5069642888,-5.0064777583,-0.7791683356
H,9.7130140976,-3.421015283,-0.0117532677
H,7.6671945827,-5.4884602362,-2.2715131657
H,6.5003650251,-5.1147061716,-1.0052917068
H,6.3647122885,-4.3484410347,-2.6046814666
C,1.4614042064,-1.8781706338,0.0820753739
H,3.3133291396,0.7264508451,0.0656116695
C,-5.0397247746,1.2242254017,0.2444593279
C,-3.6444316592,-0.4396286894,1.3529311101
C,-6.1886487577,0.5252710699,0.605339951
H,-2.6823511489,-0.7949381318,1.7030489118
C,-6.0570973295,-0.6673988497,1.313299507
H,-7.1787424,0.8993532106,0.3772421026
C,-4.7994071178,-1.1444268786,1.6835353707
H,-6.9460825959,-1.2196940195,1.5996104653
H,-4.7206199911,-2.0623104763,2.2568349447
C,2.7694304802,-2.4401598399,-0.0105429487
C,2.9335105454,-3.8391958387,-0.0135509638
C,0.3326947272,-2.7211416723,0.1067707
C,1.816053649,-4.6423498019,0.02062486
H,3.9178214695,-4.2890042299,-0.0222520362
C,0.5204279357,-4.0838072008,0.0675727837
H,-0.6715984752,-2.319914373,0.1536525007
H,1.9305406767,-5.7210317066,0.0173121292
H,-0.3413888455,-4.7427703507,0.0839041572
H,-9.070531248,5.894371161,-2.9734525196

1c$_{NHOH}$´-2 (open-shell singlet state)

–2637.3797542 hartree

C,-2.4329899299,6.8322856726,-1.6435606808
C,-2.7162739947,7.0986662091,-0.3085258387
C,-1.7690299356,5.6550618855,-2.0228796565
C,-2.3474468212,6.1989882804,0.7041839162
H,-3.2301957154,8.0163557388,-0.0411647299
C,-1.4111734075,4.7776518108,-1.021670002
H,-1.5412508495,5.4369359745,-3.0605328429
C,-1.696347877,5.0461445705,0.3219467405
H,-2.5616924787,6.3956311593,1.7489806771
O,-0.767967727,3.5842051734,-1.1311570405
O,-1.2422509562,4.0317931671,1.1086599146
C,1.3573565468,1.6766241113,0.3510909219
C,-0.7687074631,0.6095303663,0.0955364854
C,0.6237757001,0.5306675984,0.0610603099
H,1.1517059683,-0.3887857684,-0.12606483
B,-0.6476721942,3.0652732552,0.222090234
O,-1.3707718072,1.751707404,0.3304976982
O,0.781549359,2.8293910745,0.5712493007
C,-3.7493655919,-1.3774884556,-0.1415005377
C,4.7748929028,0.7455807357,0.4022520249
N,3.4701903291,0.4836838103,0.2691910686
C,3.6915264032,2.7350847428,0.757049366
C,-1.6524533775,-0.4941131282,-0.0725719457
N,-2.993403529,-0.2523078161,-0.0156080601
C,2.7908156808,1.6354767625,0.4564135384
H,-2.7279653576,7.5443609381,-2.4075885775
C,5.7590747166,-0.3047446794,0.2196821958
C,5.3594792182,-1.6450913549,0.3172986687
C,7.0950662941,-0.0303463424,-0.0857768932
C,6.2419560563,-2.6991428941,0.1485504728
H,4.319517016,-1.8300504096,0.5416624139
C,8.0355812343,-1.0366109029,-0.28026177
H,7.3915824218,0.9982495728,-0.2156318484
C,7.5858193526,-2.3691294678,-0.145978277
O,8.436337221,-3.423075062,-0.3029182101
C,5.7724535327,-4.160120396,0.2786730113
C,6.5179022954,-4.8636245084,1.4302566034
C,4.270770786,-4.2422559743,0.5936395001
C,6.0002010515,-4.9176559946,-1.0447982395
H,7.59310856,-4.8989839244,1.2581563484
H,6.3354711473,-4.3484511428,2.3787419855
H,6.1537960719,-5.8913310717,1.5316029444
H,3.6607018275,-3.792823544,-0.1959525814
H,3.9840132774,-5.2946061684,0.6739728058
H,4.0196775605,-3.7599289604,1.5428387312
H,5.6324989941,-5.9446278888,-0.948977056

H,5.4507006833,-4.4392634706,-1.8618956597
H,7.0544884176,-4.9579639961,-1.316031296
C,9.4969801392,-0.6893981629,-0.6379339856
C,10.4679827916,-1.1850566638,0.4583065938
C,9.8784939084,-1.2655152092,-2.020472128
C,9.7129886419,0.8296478143,-0.7369971934
H,10.2214662437,-0.7230401813,1.4182005776
H,10.4646996375,-2.2661535623,0.6238594022
H,11.4937055762,-0.9050775663,0.2000861653
H,9.2297821316,-0.8470749136,-2.7948272245
H,10.9115744573,-0.998412007,-2.2628516548
H,9.7989816033,-2.3529241773,-2.1004231697
H,10.7571003133,1.0255541075,-0.9957651753
H,9.09193554,1.2837250683,-1.5137486487
H,9.5113216648,1.3351761619,0.2116011757
C,-5.173154136,-1.3387882963,-0.0942252423
C,-5.8660202225,-0.1123021884,-0.3101089309
C,-5.9291524736,-2.507655764,0.1979004124
C,-7.2262294925,-0.0135507814,-0.2702461923
H,-5.2868854179,0.7662070931,-0.5640329166
C,-7.2925533089,-2.5046753404,0.2717784655
H,-5.3883505308,-3.4140433601,0.4218711018
C,-8.0093347733,-1.2348519533,0.0239177139
O,-9.2483949975,-1.1933321029,0.0648805111
C,-7.9568383139,1.3051412597,-0.5351890052
C,-8.7887044354,1.6993647166,0.7029525428
C,-6.9781966597,2.452440949,-0.8238153644
C,-8.8811777068,1.1566793484,-1.761234964
H,-9.550012432,0.9525648458,0.925496803
H,-8.1459762326,1.8205734457,1.581098446
H,-9.2843526004,2.6577047814,0.5176782189
H,-6.373557872,2.2687843145,-1.7175414074
H,-7.547234962,3.3686335573,-1.0017497262
H,-6.3068744337,2.6483885192,0.0182075627
H,-9.3781866428,2.1116872753,-1.9597631824
H,-8.3047767994,0.888665305,-2.6528518516
H,-9.644302041,0.3970934465,-1.5951274751
C,-8.0921396876,-3.7632494612,0.6181962646
C,-8.9073554032,-3.5330525415,1.9071457127
C,-9.0417919436,-4.1173615522,-0.5450212144
C,-7.1763246786,-4.9727126112,0.8555389388
H,-8.2476034192,-3.2922555758,2.747253266
H,-9.6259733515,-2.7236940338,1.7832349333
H,-9.4539054322,-4.4468933445,2.161985097
H,-8.4782702145,-4.2965830276,-1.4667160828
H,-9.588886652,-5.0344817218,-0.3033068036
H,-9.7625959596,-3.3207561822,-0.7251275412
H,-7.7923828493,-5.8430232737,1.0972739583
H,-6.5900864189,-5.2267632417,-0.0337527717
H,-6.4902940261,-4.8155507214,1.6939114341
C,-1.5000385978,-1.8974011094,-0.2801306744
H,-3.3348311362,0.6795717081,0.1836356483
C,4.982239622,2.1639443489,0.737338714
C,3.4940218624,4.0784701851,1.0577316281
C,6.0891286798,2.9406482422,1.0627527375
H,2.4974999928,4.501243758,1.0688494965
C,5.8920965288,4.2889623336,1.3640230134
H,7.0880701626,2.5247224174,1.109440745
C,4.6153017116,4.8518605111,1.3534730073
H,6.7471041033,4.9072519168,1.617749007
H,4.4915895757,5.9032210943,1.5912931141
C,-2.8117620982,-2.45295164,-0.32938513
C,-2.9814360141,-3.8236544714,-0.6007931173
C,-0.3740086208,-2.7244638528,-0.4497496229
C,-1.8653740475,-4.614340661,-0.7657250576
H,-3.9665083722,-4.2596918308,-0.7029131577
C,-0.567834991,-4.0682400809,-0.6799574395
H,0.63117796,-2.3251796069,-0.4099972524
H,-1.9847596646,-5.6721585341,-0.9743697298
H,0.2917634361,-4.7168171664,-0.8124673713
H,9.3281772167,-3.1159412341,-0.4861901276

1c$_{NHOH}$´-3 (open-shell singlet state)

–2637.3614393 hartree

C,2.0679401348,6.4942893244,2.2043726024
C,2.515624277,6.9147379318,0.9568076721
C,1.3770641067,5.281769688,2.3556731407
C,2.2917367749,6.1406196856,-0.1926999688
H,3.0465539907,7.8569363486,0.8662781789
C,1.1618354522,4.5291422499,1.2213394742
H,1.0217606031,4.9435381714,3.3228736453
C,1.6125473397,4.9523559357,-0.0339603449
H,2.6339907529,6.4582941948,-1.1714587152
O,0.5265546145,3.3311577339,1.1070264813
O,1.2785427652,4.0363353055,-0.9845909334
C,-1.3939942754,1.6524643257,-0.8292956999
C,0.6959371129,0.5390143169,-0.4169549992
C,-0.6984464576,0.4768819857,-0.5609268325
H,-1.2343096923,-0.4560938197,-0.4906130556
B,0.5803435828,2.9838442151,-0.3004220581
O,1.317389261,1.679530373,-0.4729946924
O,-0.7954715801,2.8014102204,-0.8486562654
C,2.0201782974,-2.818719037,0.0367978158
C,-4.8247197742,2.5248690573,-1.1508407307
N,-3.5286599004,2.7748132529,-0.8809276732
C,-3.6849458812,0.5783193598,-1.5655124285
C,1.5237925489,-0.6118431099,-0.248735219
N,1.0350902943,-1.8882451126,-0.1501541919
C,-2.8251259072,1.6636474991,-1.0959979418
H,2.2533278528,7.1115139135,3.0775921165
C,-5.7975934436,3.5807918735,-0.9959809919
C,-5.4093410278,4.7673133405,-0.348144848
C,-7.1073360872,3.4913432505,-1.484228445
C,-6.2748779429,5.8292305854,-0.1575169948
H,-4.3896727137,4.8182140908,0.0036834622
C,-8.0288032533,4.519941965,-1.3363943934
H,-7.3991978532,2.6063333851,-2.0238657252
C,-7.5910535589,5.6820192227,-0.6584126706
O,-8.425979746,6.737734314,-0.4566127154
C,-5.8151974495,7.1088691878,0.565814157
C,-6.6559498976,7.3373451732,1.8378471421
C,-4.3457868743,7.0090725274,1.0028636636
C,-5.9297117095,8.3255937138,-0.3743627716
H,-7.712510018,7.4787028383,1.6118692373
H,-6.557419182,6.4883187179,2.5218748269
H,-6.2956227112,8.229831515,2.3596883259
H,-3.6674301542,6.878237686,0.1552049155
H,-4.0638153707,7.9370688353,1.5078203396
H,-4.1773646095,6.1890838482,1.7068326079
H,-5.5670183635,9.2213525746,0.1400939593

H,-5.3131979684,8.1810629434,-1.2671708122
H,-6.9573436898,8.5063168117,-0.688551635
C,-9.4584588143,4.3838037644,-1.9032977079
C,-10.5105375594,4.4455799708,-0.7721153145
C,-9.7320817837,5.4613577491,-2.9771583497
C,-9.6678193578,3.0289961086,-2.5996614722
H,-10.340469106,3.6385086212,-0.0542344863
H,-10.5159054631,5.37611721,-0.1977847682
H,-11.5139816779,4.3223240445,-1.1906867928
H,-9.0241931837,5.3574100972,-3.8039774183
H,-10.7428706668,5.3391763981,-3.3781297714
H,-9.6483961922,6.4923327914,-2.6231925947
H,-10.689805356,2.9799000994,-2.9852606935
H,-8.9908499862,2.8938561156,-3.4476414439
H,-9.5382435489,2.1901154585,-1.9098412508
C,1.7209270308,-4.2029210183,0.1960804664
C,0.4681023658,-4.7252084787,-0.2369384813
C,2.6479698442,-5.0856771453,0.8146714447
C,0.1228731278,-6.0377209373,-0.0964147729
H,-0.2149399172,-4.058868629,-0.7489142672
C,2.387259177,-6.412316037,1.0098580284
H,3.5688557946,-4.6647280079,1.1880659969
C,1.092062473,-6.9524270281,0.5469793884
O,0.8178899528,-8.1538243019,0.6951877749
C,-1.2141322509,-6.5901279543,-0.5971368066
C,-2.0115804624,-7.1817424431,0.5834961293
C,-2.0793859023,-5.4968648419,-1.2410816775
C,-0.9690039635,-7.6793369406,-1.6614524867
H,-1.4758914802,-8.0084772925,1.0482758697
H,-2.2070660605,-6.4179210399,1.3434385927
H,-2.9774239832,-7.5509516389,0.2235216036
H,-1.5973919288,-5.0503155292,-2.1170453402
H,-3.0193697034,-5.9417357984,-1.5785900204
H,-2.3333349283,-4.7007280533,-0.5331727866
H,-1.9298459332,-8.0513282987,-2.0318201851
H,-0.4156134413,-7.2729540262,-2.514480146
H,-0.4094695866,-8.5177889881,-1.2485261012
C,3.3835094338,-7.3433974092,1.7057303531
C,2.7492182413,-7.9389453347,2.9793074679
C,3.7960139536,-8.4792710845,0.7466249013
C,4.6606125776,-6.6007426491,2.1241285981
H,2.4615055068,-7.1464164469,3.6779880763
H,1.8683365439,-8.5349441566,2.7432117994
H,3.4779972627,-8.581323215,3.4841567594
H,4.2606135706,-8.0747120635,-0.1587130361
H,4.5302954327,-9.1245412069,1.239738953
H,2.9388129497,-9.0867364319,0.4587192315
H,5.3383734941,-7.3063413527,2.6120492943
H,5.1933118904,-6.1799672222,1.2650298043
H,4.4580077874,-5.7963450166,2.8382367511
C,2.9373923837,-0.7128661207,-0.1563681378
H,0.0545827562,-2.109540512,-0.0760530438
C,-4.9885675805,1.1274669127,-1.5776747071
C,-3.4596358898,-0.7278944198,-1.9900994234
C,-6.0726797859,0.3298572155,-1.9376246726
H,-2.4588911712,-1.1384452134,-2.0543011012
C,-5.8433368532,-0.9874682759,-2.3281772726
H,-7.0894026885,0.6997356321,-1.9131642048
C,-4.5492778156,-1.5068215982,-2.3702716758
H,-6.682690542,-1.6135922958,-2.6121806253
H,-4.3895633933,-2.5270225178,-2.7036190062
C,3.2552723656,-2.0902338443,0.0156683299
C,4.6050268382,-2.4921572,0.0632052728
C,3.9523388482,0.2613987953,-0.2337131833
C,5.5844192559,-1.5269639143,-0.013276404
H,4.8824922214,-3.5357366848,0.1366232299
C,5.2600456772,-0.1581911834,-0.1506853074
H,3.6953263471,1.3047729046,-0.3597377397
H,6.6277000906,-1.8226193002,0.0238764339
H,6.0602945828,0.5723722177,-0.2047328111
H,-9.2969100888,6.5590160226,-0.8211204338

$1c_{NHOH}$´-4 (open-shell singlet state)

–2637.3603416 hartree

C,2.6037666385,6.2037832506,2.4518791332
C,2.9270840359,6.7079740898,1.197341129
C,1.8787394648,5.0090021268,2.5877635822
C,2.5384543144,6.0376867558,0.0260538992
H,3.487037772,7.6343588605,1.1178879332
C,1.5013059847,4.3565232835,1.4327966442
H,1.6186616144,4.6076685739,3.5613193421
C,1.8275633188,4.8654169871,0.1687424307
H,2.7819170901,6.421543431,-0.9587819312
O,0.8020762919,3.2013042846,1.3051180088
O,1.3471472186,4.0484262242,-0.8055616626
C,-1.3883836756,1.7134315375,-0.4343635511
C,0.707620382,0.5307504796,-0.3119326245
C,-0.669855573,0.4981469627,-0.2758527525
H,-1.2492207002,-0.4097744035,-0.1775614755
B,0.6905234051,2.9506458056,-0.1349596487
O,1.3724179363,1.6638606485,-0.4806522119
O,-0.7333701952,2.8540635168,-0.54100457
C,2.0236471241,-2.8553757105,0.1176853175
C,-4.7769097686,0.8416806538,-0.540524435
N,-3.5051810956,0.5260760684,-0.4666760962
C,-3.6623544251,2.8437273683,-0.640460926
C,1.4993014881,-0.6483098393,-0.2218476316
N,1.023517594,-1.8856261061,0.0448057414
C,-2.773998913,1.7041368201,-0.5103705618
H,2.9138013572,6.740395995,3.3429965725
C,-5.8283657489,-0.1762618215,-0.5280907214
C,-5.5880103614,-1.4333070582,-1.0875124549
C,-7.074436777,0.0734573457,0.0442291551
C,-6.5535804754,-2.4308233591,-1.1145619634
H,-4.6128683605,-1.6008971437,-1.5220306274
C,-8.0836034281,-0.8868682037,0.0769357324
H,-7.239675399,1.0335827729,0.5102608212
C,-7.800451073,-2.1324959725,-0.5217385054
O,-8.7327537728,-3.1323496415,-0.5570482054
C,-6.2732401617,-3.7942598707,-1.7737003523
C,-7.2455286091,-4.0345239287,-2.9458865396
C,-4.8498637017,-3.8600621939,-2.3481749089
C,-6.3998433091,-4.9289246419,-0.7377111034
H,-8.2833299019,-4.0574680982,-2.6154227121
H,-7.1394576652,-3.2486719607,-3.7005263459
H,-7.0169097369,-4.9925362055,-3.4247495474
H,-4.0881598223,-3.7299484944,-1.5738650643
H,-4.695323908,-4.8436748702,-2.8009049828
H,-4.6838550954,-3.1094469628,-3.1264397316
H,-6.1630494023,-5.8884465844,-1.2096481764

H,-5.6959389382,-4.7760771624,0.086655322
H,-7.4054399433,-4.9910022189,-0.3234401418
C,-9.4394475375,-0.5798600016,0.7491910648
C,-10.5942373173,-0.6443809135,-0.2767416059
C,-9.6962156737,-1.5373150754,1.9349624238
C,-9.4755839567,0.8408063469,1.3360638955
H,-10.4284648132,0.081658002,-1.0773973524
H,-10.7274269144,-1.6158976608,-0.7612450373
H,-11.5414153406,-0.3991992775,0.213350863
H,-8.9103831967,-1.4235613026,2.6867352351
H,-10.6540959346,-1.2977259358,2.406865259
H,-9.7227493495,-2.5971198955,1.6682660511
H,-10.4492339528,1.0090256428,1.8045751082
H,-8.7114632439,0.9876938826,2.1039096707
H,-9.346251297,1.6067062507,0.5664135626
C,1.7586333907,-4.1625919338,0.4664627093
C,0.4011024854,-4.6373655857,0.6113450982
C,2.8224714611,-5.098086022,0.7363654152
C,0.0898013122,-5.9066123008,0.9539804386
H,-0.4097172174,-3.9527096658,0.4000727907
C,2.6094052801,-6.3841305992,1.0925365274
H,3.832695981,-4.7282976775,0.6938061644
C,1.2045510122,-6.8695077787,1.2012007038
O,0.9653820617,-8.0382520932,1.4940342463
C,-1.354730217,-6.3941609199,1.0854915259
C,-1.6074045508,-6.9015778315,2.5204145734
C,-2.3611892404,-5.2702462403,0.8055473488
C,-1.6231381445,-7.5267152863,0.0729768057
H,-0.9679008955,-7.7491859323,2.7635139794
H,-1.4315249804,-6.1059698432,3.2518511451
H,-2.6512012958,-7.2171222474,2.6150598893
H,-2.2752800259,-4.8818380705,-0.2143247247
H,-3.3744688818,-5.6648322237,0.9137922235
H,-2.2609368518,-4.4381725146,1.5103248516
H,-2.6661533824,-7.8481665319,0.1558290315
H,-1.4613520679,-7.1786919016,-0.9525102784
H,-0.9814548961,-8.3875628957,0.2566997356
C,3.7557537455,-7.3504623416,1.4006852589
C,3.6381228217,-7.8647279164,2.8499872242
C,3.7182777518,-8.5390114536,0.4178035778
C,5.1243314809,-6.6699631639,1.2566026662
H,3.6762393787,-7.0342625221,3.5625808113
H,2.7115216196,-8.4165592987,3.0041776179
H,4.4775544674,-8.5321469447,3.0694042231
H,3.8133942654,-8.1921994879,-0.6164520273
H,4.5587692481,-9.2092524872,0.6245221845
H,2.7932982615,-9.1063789989,0.5130333198
H,5.9086324552,-7.3956719162,1.4871410547
H,5.3019434373,-6.3143371277,0.2360984811
H,5.2444348985,-5.8299808324,1.9484649988
C,2.9217679356,-0.7770186402,-0.3968587897
H,0.0667594162,-2.0579030859,0.3104709219
C,-4.9610671663,2.2993824395,-0.6634935222
C,-3.4484720552,4.2142730775,-0.7578267824
C,-6.0656927278,3.1244594103,-0.839302396
H,-2.4440604629,4.6183983393,-0.741166543
C,-5.853299037,4.4984084005,-0.9554966604
H,-7.0706049572,2.7233226913,-0.9046585685
C,-4.5643134278,5.0351089823,-0.9078705128
H,-6.7028105093,5.1599429416,-1.0920691953
H,-4.4290211092,6.1077761442,-1.0012482417
C,3.2578780976,-2.1387199928,-0.2125972595
C,4.5785980727,-2.5439327197,-0.4126713596
C,3.8785554372,0.1858114796,-0.7296861596
C,5.5281689875,-1.584206096,-0.7429128278
H,4.8850225026,-3.5777995804,-0.3405709211
C,5.1888740143,-0.2351138959,-0.8912339605
H,3.5839707113,1.2191900611,-0.8550502645
H,6.5567932452,-1.8943840792,-0.8947581317
H,5.9569046225,0.4867043487,-1.1464898536
H,-9.5559575538,-2.8403493915,-0.1574719535

1c$_{OHOH}$-1 (open-shell singlet state)

–2637.3524735 hartree
C,-1.7510478722,7.0788787738,-2.7468472871
C,-2.243206982,7.4891801997,-1.5128500725
C,-1.1474005754,5.8211452281,-2.9042828456
C,-2.152901858,6.6594565312,-0.3837820167
H,-2.7048580218,8.4667726066,-1.4168064779
C,-1.0635465082,5.0142026872,-1.7903849895
H,-0.7590302113,5.4895667489,-3.8610315724
C,-1.5588015301,5.4271915232,-0.5481646649
H,-2.5318428842,6.9681895167,0.5842892
O,-0.5308344927,3.7667933301,-1.6858040149
O,-1.3556674919,4.4563058545,0.3830304027
C,1.1571287066,1.9138985664,0.3346790473
C,-0.9647808695,0.9503240786,-0.2378108303
C,0.4037753436,0.788855992,-0.0092016684
H,0.852550835,-0.1880742758,-0.0158408338
B,-0.6974529506,3.3768371421,-0.2990781853
O,-1.5122513128,2.1199825596,-0.2159230701
O,0.6397295206,3.09678993,0.3221610826
C,-3.7545584684,-1.2440965453,-0.3569688943
C,4.592652213,2.535150567,0.9850470381
N,3.3454937821,2.887486322,0.6217893139
C,3.2907741754,0.6659509145,1.2496381951
C,-1.8549769779,-0.1848329087,-0.4251472779
N,-3.1587512168,-0.0510610544,-0.1748481542
C,2.5539269464,1.8188138838,0.7300912598
H,-1.8325661336,7.7392004098,-3.604356144
C,5.6619063845,3.5016905339,0.8993132503
C,5.3356947113,4.8595649791,0.7391378458
C,7.0148677582,3.1426832186,0.9375254589
C,6.2979323449,5.849187244,0.6461852773
H,4.285099691,5.1059507164,0.6932724846
C,8.0350292231,4.0805154332,0.8408855782
H,7.2698175363,2.09769471,1.0047319634
C,7.6507100646,5.4354698377,0.7055331449
O,8.579819846,6.4263567569,0.6157964231
C,5.9028997441,7.3288434794,0.4832662623
C,6.4588274656,7.8883563955,-0.8416133652
C,4.3767193062,7.500823561,0.4443057749
C,6.4262454413,8.1579279874,1.6731581211
H,7.547592773,7.8583080006,-0.8751238383
H,6.0686166708,7.3209609772,-1.6924253271
H,6.1416806519,8.9293732808,-0.9612351849
H,3.8976094156,7.162141871,1.3673819957
H,4.1440256017,8.5626101377,0.32533325
H,3.9191119208,6.9693447178,-0.3946968744
H,6.1082572039,9.1996365653,1.5617248077

H,6.0138062882,7.7821470429,2.6150225764
H,7.5137307466,8.1393594203,1.739840695
C,9.5147794102,3.6407508162,0.8704449638
C,10.2280467793,4.0117158351,-0.4496912339
C,10.2482862594,4.2473392507,2.0887869683
C,9.6524215873,2.1159624035,1.0124157011
H,9.7406148516,3.5154498792,-1.2933068518
H,10.2376800828,5.0803288391,-0.6800609073
H,11.2701069092,3.6793822585,-0.4161562258
H,9.767923568,3.9256588023,3.016911215
H,11.2868416276,3.9032123921,2.1065402201
H,10.2735012106,5.3402803963,2.1148628641
H,10.7136800834,1.8529966658,1.0256736306
H,9.2135632892,1.7504472315,1.9451917405
H,9.192713958,1.5820665532,0.176293595
C,-5.1695701036,-1.3809128368,-0.1042861882
C,-5.9425019044,-0.216775834,0.0266516301
C,-5.8051987563,-2.6222907507,0.0420226298
C,-7.3061719622,-0.2540742957,0.2679617947
H,-5.4226512811,0.7244556793,-0.0733638423
C,-7.1642450708,-2.7363205201,0.2924886417
H,-5.2080730719,-3.5185380903,-0.0049955429
C,-7.9009737904,-1.5330269369,0.392517895
O,-9.2350522868,-1.6696630156,0.6261330149
C,-8.1167784236,1.0538697203,0.3926035885
C,-8.7516531646,1.1770993215,1.796514473
C,-7.2199924588,2.2904627053,0.2191360283
C,-9.1930747539,1.1444622901,-0.712976303
H,-9.4541807327,0.3798570987,2.0568481787
H,-7.9728698287,1.1721997857,2.5638841469
H,-9.2967860833,2.1225325629,1.8739821265
H,-6.7468414705,2.3208719525,-0.7655484816
H,-7.8322327681,3.1910452315,0.317973343
H,-6.4350965172,2.3404229614,0.9778810314
H,-9.7351404871,2.090702327,-0.6245615391
H,-8.7232658759,1.1152350461,-1.6998328178
H,-9.9399533608,0.3451089681,-0.697284542
C,-7.8320182706,-4.1139578868,0.4615201562
C,-8.4578445536,-4.2395810761,1.8649426431
C,-8.9080969986,-4.3253911858,-0.6224611785
C,-6.8156561895,-5.2573545905,0.3165039084
H,-7.6935782181,-4.1212637463,2.6396976994
H,-9.235626538,-3.4953547124,2.0319691513
H,-8.9039213651,-5.232678516,1.9823367908
H,-8.4652205237,-4.2666443068,-1.6219066639
H,-9.3533601927,-5.319494978,-0.5105944144
H,-9.704933059,-3.5857387997,-0.5553797034
H,-7.3350922981,-6.2109487109,0.444853371
H,-6.3494584222,-5.2718858411,-0.673790818
H,-6.0274215854,-5.2104457126,1.074113875
C,-1.5365803956,-1.5476462904,-0.853560343
H,-9.6572780848,-0.8077979829,0.6841326772
C,4.6146172725,1.1326130765,1.4220191264
C,2.9343524691,-0.6201962466,1.6440370225
C,5.5782370652,0.3037686956,1.990108221
H,1.9142451868,-0.9776019132,1.5709012343
C,5.2210056504,-0.9922593543,2.3567150092
H,6.5868944724,0.6479933031,2.1803072287
C,3.914610765,-1.4493565254,2.1849601964
H,5.9647806872,-1.6463095216,2.7998253728
H,3.6516183019,-2.4550237137,2.496028892
C,-2.7719494834,-2.2351924773,-0.8144742283
C,-2.850492694,-3.5561032549,-1.2472824202
C,-0.3890873848,-2.1770047508,-1.326995529
C,-1.6914556604,-4.1885957512,-1.6923079414
H,-3.7897311316,-4.0937667572,-1.2728062147
C,-0.4751106044,-3.5072412428,-1.7319121929
H,0.5540236502,-1.6518393688,-1.4194439657
H,-1.7416175304,-5.2180817414,-2.0312584349
H,0.4113822186,-4.0111143102,-2.1027831275
H,9.4690992559,6.0665202889,0.6699129655

$1c_{OHOH}$-2 (open-shell singlet state)

–2637.3641427 hartree

C,-2.3128866752,7.0065963958,-1.7972843464
C,-2.5466634341,7.3558135885,-0.4718464526
C,-1.7152897416,5.780177672,-2.1289539019
C,-2.1926440909,6.4936600314,0.5783691287
H,-3.0099007247,8.3098949801,-0.2413392715
C,-1.3717647921,4.9403749452,-1.0913671177
H,-1.5273744388,5.4961970639,-3.1586491144
C,-1.6070041917,5.2922020979,0.2430580798
H,-2.3700918571,6.7540922343,1.6160714926
O,-0.786685662,3.7147325354,-1.1522609672
O,-1.1776678351,4.3017543915,1.0708551963
C,1.3230161759,1.8381753039,0.356953903
C,-0.8446802894,0.8248001208,0.2538661469
C,0.542268759,0.7012358929,0.1572443192
H,1.0311043054,-0.237935827,-0.0341271252
B,-0.6546188559,3.2634742477,0.2224879225
O,-1.3987837389,1.9845141405,0.4230910393
O,0.7921648664,3.0111345697,0.5404127125
C,-3.6608656229,-1.3049100285,-0.0902665336
C,4.7147490038,0.8031873636,0.3398419954
N,3.3956891076,0.5779976233,0.2672224637
C,3.7053993098,2.842865639,0.6190923097
C,-1.7390403585,-0.3157303592,0.1830271839
N,-3.0412176387,-0.1135120573,-0.0323247447
C,2.7617618014,1.7572853979,0.4118881188
H,-2.5951416424,7.6909546668,-2.5910159717
C,5.6527880264,-0.2900806077,0.1816716861
C,5.2008863092,-1.6089095538,0.3375374178
C,6.9940359734,-0.0834435283,-0.1551051961
C,6.0362981374,-2.7039860955,0.197308341
H,4.1579090693,-1.7418637202,0.5828337386
C,7.8890529579,-1.1342577252,-0.323799573
H,7.3306162468,0.9257683325,-0.3297564122
C,7.3875814992,-2.4412562307,-0.1304218761
O,8.1913177894,-3.5342395959,-0.2591624967
C,5.5093645328,-4.1380916178,0.3908532189
C,6.2461295734,-4.8288989846,1.5556492574
C,4.0113941706,-4.1449845872,0.7341115238
C,5.6811072706,-4.9528188825,-0.9068183845
H,7.3155565886,-4.9164731281,1.3670447924
H,6.1029552589,-4.2715254272,2.4868911724
H,5.8413227442,-5.8357163054,1.7021110746
H,3.4049294085,-3.7015845606,-0.0614959901
H,3.6842864832,-5.1811040577,0.8600511155
H,3.7999558368,-3.6172466411,1.6689714051
H,5.2736436161,-5.9593788259,-0.7661273265

H,5.1361129168,-4.4828550653,-1.7316472841
H,6.7277059601,-5.0468966479,-1.1941894029
C,9.3573090296,-0.8621786612,-0.7168286045
C,10.3251933486,-1.3554393811,0.383216016
C,9.6916064338,-1.5062880798,-2.0814554044
C,9.633534427,0.6416692213,-0.8782606693
H,10.1144237492,-0.8463201447,1.3276730511
H,10.2795261285,-2.4281519932,0.59145788
H,11.3571591864,-1.1293021199,0.0984741174
H,9.0464585181,-1.0926399302,-2.8613251622
H,10.7300056949,-1.2903109144,-2.3505298749
H,9.5682662514,-2.5919764971,-2.1174448252
H,10.6805410483,0.7841420472,-1.159264935
H,9.0193597414,1.0908621167,-1.6632979593
H,9.4677319393,1.1909745225,0.0527940316
C,-5.0875461903,-1.347806649,-0.31424151
C,-5.7412321744,-0.1595275995,-0.6785334882
C,-5.8589135061,-2.5092474562,-0.1636633807
C,-7.1052302586,-0.1018148139,-0.9115664364
H,-5.125548137,0.722475479,-0.7716572865
C,-7.2291066086,-2.5258203079,-0.3749846066
H,-5.3751808483,-3.4158767827,0.1587221504
C,-7.8340916412,-1.3060208088,-0.7575802313
O,-9.1780900906,-1.3478944769,-0.9680297834
C,-7.7759350246,1.22841815,-1.3167748737
C,-8.8162217979,1.6653289565,-0.2602832133
C,-6.7514447483,2.3711971286,-1.4056110897
C,-8.4205142516,1.1179929951,-2.7172072533
H,-9.64301166,0.9648653182,-0.1089882951
H,-8.3336433201,1.7961288358,0.7119935932
H,-9.2574601929,2.6235447368,-0.5503314518
H,-5.9832349802,2.1774294989,-2.1584233365
H,-7.2679198585,3.2915003255,-1.6917724746
H,-6.2549753178,2.5558021771,-0.4498177904
H,-8.8638954431,2.0783640162,-2.9967866874
H,-7.661322746,0.8665512767,-3.4628098907
H,-9.2083120548,0.3638457621,-2.8048065614
C,-8.0490128102,-3.8167494392,-0.1913110324
C,-9.1076372439,-3.6285670583,0.9136784504
C,-8.7280228257,-4.2144788119,-1.5173340097
C,-7.1600706967,-4.9968506966,0.2313328329
H,-8.6301519118,-3.376232047,1.8658501391
H,-9.8190365997,-2.8416281162,0.666014838
H,-9.6642676604,-4.5610540211,1.0539049543
H,-7.9797569458,-4.3786123761,-2.2994090926
H,-9.2822604924,-5.1490632356,-1.381632425
H,-9.4252251942,-3.4521542252,-1.8628137565
H,-7.7850694986,-5.8863527549,0.3488288384
H,-6.3997558013,-5.230562889,-0.52071273
H,-6.6625837875,-4.8188379914,1.1896697635
C,-1.4423616251,-1.7463931679,0.2977248337
H,-9.507817985,-0.478728175,-1.213694248
C,4.9770458863,2.2305184543,0.5870714529
C,3.5574240048,4.2060218063,0.8487885247
C,6.1169455063,2.9903012069,0.8279204086
H,2.5744587597,4.6591942293,0.8701776772
C,5.9699540892,4.3589890155,1.0583171166
H,7.1051315889,2.5486193195,0.8631055895
C,4.7108537125,4.9598854283,1.0604997164
H,6.8514360323,4.9635875234,1.2457606262
H,4.6264047841,6.0262478058,1.2418867287
C,-2.6860594901,-2.3902908052,0.0980117981
C,-2.758601188,-3.7807690293,0.095999682
C,-0.2948782979,-2.4880251627,0.5614444524
C,-1.6000709684,-4.5170994015,0.334863569
H,-3.6844371998,-4.3067565564,-0.0964269435
C,-0.3851894363,-3.8780648973,0.57743148
H,0.6577202272,-2.0162126403,0.7666056264
H,-1.6477114246,-5.6010701289,0.3367061504
H,0.5031683067,-4.4676868649,0.7790187678
H,9.091209101,-3.2720148889,-0.4708073503

$1c_{OHOH}$-3 (open-shell singlet state)

–2637.3693771 hartree

C,-2.4936328391,6.7382363133,-1.1541623072
C,-2.6888774137,6.9375266372,0.2079986257
C,-1.8669342896,5.5774775986,-1.6344674663
C,-2.2657473733,5.9842461874,1.1480070725
H,-3.1761730137,7.8434383115,0.5540344209
C,-1.4550467524,4.6473177508,-0.7042532191
H,-1.7092810447,5.410299735,-2.694344465
C,-1.6516922998,4.8481657421,0.6669995962
H,-2.4121769745,6.127742523,2.2129833841
O,-0.8313922917,3.4576334619,-0.9150456787
O,-1.1594292249,3.7917375239,1.3698641513
C,1.4221879551,1.5111322108,0.3893436161
C,-0.7005468415,0.4251764502,0.2314193429
C,0.6896692906,0.3519493351,0.1375177566
H,1.184224844,-0.5823497173,-0.0773919996
B,-0.6206557632,2.8720983365,0.3988806735
O,-1.3092883035,1.5486868226,0.4919628691
O,0.837902325,2.6510957724,0.6369541943
C,-2.0603890309,-2.7966555431,-0.3574387006
C,4.8776121414,0.7250575318,0.2844973207
N,3.5836149012,0.3900135643,0.2618997201
C,3.7237318567,2.6837363078,0.5687722721
C,-1.5436198428,-0.7310790714,0.0784530054
N,-1.037326534,-1.9406214075,-0.2339590138
C,2.8611514494,1.5219049276,0.4107543525
H,-2.8301650832,7.4901957429,-1.8606953736
C,5.9160390056,-0.277499731,0.1346001781
C,5.6543748696,-1.6057600506,0.4942361745
C,7.1765928912,0.040655553,-0.3778176182
C,6.6119120305,-2.6028206842,0.392243506
H,4.6681173294,-1.8258251512,0.8765487968
C,8.1762069595,-0.9133064753,-0.5373175833
H,7.3567017273,1.0540998008,-0.7016118051
C,7.8721388777,-2.2318248851,-0.1316848674
O,8.7926837898,-3.2334004117,-0.2323384643
C,6.311171403,-4.0452018775,0.8394381045
C,7.2685212877,-4.4676518018,1.971919765
C,4.8815103317,-4.1764580277,1.3851909988
C,6.4365207758,-5.0147511853,-0.3526736814
H,8.3096980437,-4.45904049,1.651764646
H,7.1647982275,-3.7993115885,2.8326136222
H,7.0209855938,-5.481382403,2.3038979397
H,4.1268089462,-3.9321778965,0.6322801247
H,4.7151018226,-5.2121772423,1.6945310274
H,4.7140294555,-3.5425510405,2.2607846298
H,6.190937592,-6.0315004706,-0.0276021217

H,5.7387839418,-4.7370834051,-1.1492549142
H,7.4443909965,-5.0248237921,-0.7663188406
C,9.5439569429,-0.5272793631,-1.1409800476
C,10.6840747242,-0.756090265,-0.1222138408
C,9.8063201386,-1.3025435373,-2.4520503751
C,9.6016320934,0.9639435853,-1.5109381795
H,10.5144751531,-0.1557106374,0.7757904485
H,10.8031349767,-1.7902438854,0.2133952573
H,11.6395259392,-0.450760991,-0.5593380834
H,9.0306305003,-1.0719801021,-3.1873605999
H,10.7716385804,-1.0056796577,-2.8732423321
H,9.821669251,-2.3903559744,-2.3444537473
H,10.5829878829,1.1881401615,-1.9377857653
H,8.8498025698,1.2317182639,-2.2580388834
H,9.4704471642,1.6088760022,-0.6375559338
C,-1.831207541,-4.1714652241,-0.7520768093
C,-0.5539575122,-4.7196047227,-0.6279882768
C,-2.8444247325,-4.9756234278,-1.295502831
C,-0.2510512525,-6.0287550449,-0.9906164197
H,0.2160405743,-4.076772069,-0.2220724985
C,-2.6203496924,-6.2835359325,-1.6880141822
H,-3.8190713287,-4.5419941386,-1.4506741772
C,-1.3103986189,-6.8023659195,-1.5146541711
O,-1.1498330299,-8.0982908404,-1.8967840676
C,1.2202857724,-6.4663670948,-0.761764027
C,2.1462224645,-5.5262988321,-1.5599210948
C,1.5385112119,-6.3551015685,0.7426489286
C,1.5983339297,-7.9000241015,-1.1821797054
H,1.9444922015,-5.6002723961,-2.6328755064
H,2.0225113627,-4.481007076,-1.2716793618
H,3.193185926,-5.7965828407,-1.389654215
H,0.8930448344,-7.01753405,1.327573697
H,2.5789430488,-6.6388220236,0.9299186527
H,1.399735323,-5.3391749817,1.1170508746
H,2.6614301287,-8.0527514679,-0.9787898727
H,1.0752371944,-8.6685715271,-0.6003471494
H,1.4736544709,-8.0762735323,-2.2570238814
C,-3.7529969677,-7.133155785,-2.2972355212
C,-3.3884670901,-7.5711468954,-3.7300167948
C,-4.0350045055,-8.3671174449,-1.4163363407
C,-5.0664523941,-6.340536512,-2.3912720309
H,-3.2209409073,-6.697759137,-4.3683831805
H,-2.4932647639,-8.1912087593,-3.7514731028
H,-4.2143669263,-8.1470767121,-4.1603150305
H,-4.3256178965,-8.060644403,-0.4062555915
H,-4.8634309489,-8.9419843383,-1.843225044
H,-3.168044323,-9.0220090652,-1.34161696
H,-5.8355207396,-6.9820844845,-2.830005476
H,-5.4296576276,-6.02427975,-1.408358624
H,-4.9743007501,-5.4575356116,-3.0307933571
C,-2.9930317858,-0.77993667,0.2051720822
C,5.0357798178,2.1725968979,0.4929312473
C,3.48458452,4.0362404236,0.7853714828
C,6.1259525119,3.0149900814,0.6755259111
H,2.473330795,4.4171514657,0.8437935379
C,5.8869491004,4.3733521297,0.891762905
H,7.1430630384,2.6417873893,0.6745978607
C,4.5870106291,4.877128488,0.9375172392
H,6.7268156815,5.0457631385,1.033853825
H,4.4292673235,5.9370684543,1.1074533814
C,-3.335085362,-2.1227836388,-0.0587331677
C,-4.6567941387,-2.5387105066,0.0583425118
C,-3.9567875833,0.1634994488,0.5426437434
C,-5.6252908554,-1.5926840797,0.3984135747
H,-4.9490339032,-3.5715492098,-0.0842728744
C,-5.2825061207,-0.2606029276,0.6283311855
H,-3.6800129383,1.1910624292,0.7393875932
H,-6.6610654066,-1.902862245,0.4910490584
H,-6.0556783107,0.4544164193,0.8894517804
H,9.6238427105,-2.8971136936,-0.5777135185
H,-0.2426205,-8.3859569851,-1.7636026357

$1c_{NH}^{-}$-1 (closed-shell singlet state)

–2636.8772968 hartree
C,-2.622476001,6.8263195976,-2.6642842432
C,-2.8003776092,7.263128365,-1.3569686805
C,-1.9964727951,5.5985826037,-2.9382991639
C,-2.359495603,6.4897405733,-0.2697598896
H,-3.2883187349,8.214640683,-1.16865686
C,-1.565786735,4.8448635676,-1.8662805805
H,-1.8533910114,5.2460305063,-3.9542609675
C,-1.7458924458,5.286625668,-0.5465604196
H,-2.4932118265,6.8180557063,0.755454111
O,-0.9460854861,3.6433498653,-1.8756108595
O,-1.2461872702,4.3788624412,0.3248949305
C,1.3200816034,1.9745706914,-0.4143841759
C,-0.8049423016,0.8424850559,-0.3027985574
C,0.6110915735,0.8104312776,-0.5038320488
H,1.1211913835,-0.1292276121,-0.6240867469
B,-0.7010923865,3.2915409277,-0.466938659
O,-1.3899000449,2.0193704267,-0.126358282
O,0.7534829859,3.1436539212,-0.2232726475
C,-3.688768121,-1.2452997864,0.0623053925
C,4.8396758171,2.5591257543,0.0541345425
N,3.4824372948,2.8490686417,0.2125623507
C,3.6560124211,1.0199920915,-1.1710516573
C,-1.6247793533,-0.2666233959,-0.2308642477
N,-2.9935328424,-0.0909209062,-0.0197753263
C,2.7887698578,1.9699932249,-0.4444236116
H,-2.9726632746,7.4395799591,-3.4891228785
C,5.7742671266,3.3196107515,0.7147277149
C,5.3336239496,4.4604419903,1.4913893995
C,7.1890989111,3.0327127066,0.6853676328
C,6.1840831514,5.272473446,2.1554657429
H,4.2668008431,4.6320248127,1.4982535058
C,8.1068476522,3.7878914541,1.3262931151
H,7.5105307798,2.1604577348,0.1410917218
C,7.6413450493,4.9799392672,2.0968559463
O,8.4573634854,5.7002069534,2.6750157323
C,5.6989494738,6.4829632935,2.9599164743
C,6.30391703,7.7782639028,2.3819388851
C,4.1707935019,6.6162364777,2.9076768184
C,6.1073655311,6.3320417049,4.439193043
H,7.392253757,7.771469541,2.4410985054
H,6.0081529091,7.9089562562,1.3357804859
H,5.9298014443,8.6403683109,2.9447600867
H,3.665032863,5.7427867163,3.3295374289
H,3.8688410113,7.4880744323,3.4954795936
H,3.8023104714,6.7627659411,1.8883059931
H,5.7316422327,7.1867654255,5.0120707904

H,5.6714022124,5.4243209933,4.8695057153
H,7.1906232037,6.2888122359,4.5520010844
C,9.6035016804,3.4629884888,1.2949387162
C,10.38399168,4.6284840855,0.6538097702
C,10.1215329545,3.2127573726,2.7256841692
C,9.8934047637,2.2012690492,0.4692568361
H,10.03968332,4.80814229,-0.3701258669
H,10.2653048644,5.5465420093,1.2282139142
H,11.4496502311,4.3778259619,0.6110400882
H,9.5889142325,2.3759832001,3.1897956621
H,11.1860504078,2.95616457,2.6918603364
H,9.9972936811,4.096059738,3.351273436
H,10.9701704287,2.0087329592,0.4778076563
H,9.3984683385,1.3159800482,0.8814673569
H,9.5899629698,2.3158567173,-0.5765776875
C,-5.0848526062,-1.3028461183,0.2798567159
C,-5.9036577896,-0.1615327865,0.0524885171
C,-5.7236963312,-2.4716588222,0.773545198
C,-7.2545768223,-0.1531167831,0.2587612324
H,-5.4339163064,0.7280999814,-0.3518251741
C,-7.0667219091,-2.542644119,1.0223711334
H,-5.1021255822,-3.3200091344,1.0197048339
C,-7.9101107252,-1.3706975601,0.7584485848
O,-9.1469350001,-1.4058681177,0.9548268958
C,-8.1109883211,1.0859959832,-0.0345512931
C,-8.8191586353,1.5506093703,1.2538712385
C,-7.2731691091,2.2639136932,-0.5515972662
C,-9.1642930654,0.7563006167,-1.1109825766
H,-9.4568129735,0.7602029517,1.6497710999
H,-8.0840648899,1.8271574969,2.0175508843
H,-9.434929652,2.4333716324,1.0438503299
H,-6.7656515837,2.0298340799,-1.4930446781
H,-7.9318769404,3.1175887319,-0.7393694642
H,-6.5202649214,2.5870234406,0.1745418633
H,-9.7800367861,1.6406762788,-1.3143813808
H,-8.6772487975,0.4625807556,-2.0473926773
H,-9.8112636367,-0.0566701304,-0.7812657406
C,-7.7177299116,-3.8123577061,1.5877637155
C,-8.3888920796,-3.5066214525,2.9413146901
C,-8.7735121399,-4.3466506369,0.5992552298
C,-6.6965417948,-4.9350174537,1.8222341069
H,-7.6458001162,-3.1643300021,3.6699451626
H,-9.1487607678,-2.7335777208,2.8266358392
H,-8.8613795038,-4.4127384311,3.3399177291
H,-8.306499637,-4.6095968113,-0.3564358371
H,-9.2454387446,-5.249823406,1.0052950627
H,-9.5426043847,-3.5958857375,0.418004562
H,-7.2138676761,-5.8127435312,2.2230951241
H,-6.2026195415,-5.2415273512,0.8936744617
H,-5.9258286765,-4.6481275474,2.5452737571
C,-1.4285100261,-1.6877678544,-0.305356341
H,-3.3720609743,0.8327060928,0.1342189858
C,4.9754319631,1.3948061231,-0.851994541
C,3.3906450211,0.002201548,-2.0775483532
C,6.0409610767,0.7146266042,-1.4364270745
H,2.3749552875,-0.242368853,-2.3647633531
C,5.7738568237,-0.3288957875,-2.321561421
H,7.0721928237,0.9795602057,-1.2450572404
C,4.4647426698,-0.6803524272,-2.6442606382
H,6.601801302,-0.8610517363,-2.7791043823
H,4.2795331134,-1.4797693255,-3.3543604028
C,-2.7003120621,-2.2984582055,-0.1405512018
C,-2.8376791312,-3.6865885178,-0.263531181
C,-0.2970732775,-2.4904549828,-0.522357319
C,-1.7133907853,-4.461339105,-0.4856956105
H,-3.8105719131,-4.1566151705,-0.207794179
C,-0.4490261663,-3.8623082806,-0.5999417531
H,0.689748891,-2.0590884692,-0.6312169526
H,-1.8107174661,-5.5378760817,-0.5798949206
H,0.4247378102,-4.4852829165,-0.7657623983

1c$_{NH}$$^{-}$-2 (closed-shell singlet state)

–2636.8879235 hartree
C,-2.0566010151,6.8784461039,-2.3801867529
C,-2.3772789543,7.2431227046,-1.077948895
C,-1.4583522132,5.6379379033,-2.6583136329
C,-2.1124388756,6.3813519411,0.0000402103
H,-2.8420774589,8.2056018375,-0.8869046694
C,-1.2010593292,4.7966146896,-1.5954473424
H,-1.206031225,5.3410009112,-3.6707497309
C,-1.5249694815,5.1658957442,-0.2806104773
H,-2.3608096098,6.6518101088,1.020870517
O,-0.640640631,3.5674324568,-1.6109465751
O,-1.1780972901,4.1817213763,0.5821075484
C,1.3672648409,1.698120318,0.0476680631
C,-0.7900261167,0.6656152842,-0.2052334484
C,0.6332296518,0.5736383605,-0.186018913
H,1.1462793706,-0.3653189506,-0.3079675702
B,-0.5861449961,3.1135131558,-0.2096998658
O,-1.3533024024,1.8597515862,-0.036889859
O,0.8211665665,2.8952198056,0.2020648168
C,-3.7749746627,-1.2998769111,-0.375784784
C,4.8429098142,0.8000586917,0.2293382071
N,3.4877109612,0.5273977864,0.071875943
C,3.7106812161,2.7926703313,0.4115100602
C,-1.6548507767,-0.4029037099,-0.3320393962
N,-3.0295416733,-0.1753221177,-0.292742145
C,2.8209405722,1.6459778766,0.1694972275
H,-2.2726533409,7.5588921213,-3.1983227606
C,5.7579986039,-0.2285685666,0.1655829238
C,5.2906972592,-1.5770054365,-0.0686307639
C,7.1778103866,-0.0348901273,0.3221526885
C,6.1154144622,-2.6448112192,-0.1451013792
H,4.2221986326,-1.6842953137,-0.1815707177
C,8.0736302485,-1.0458084554,0.2610018994
H,7.5286796566,0.9679740777,0.4965965924
C,7.5765069865,-2.4300732906,0.0201157355
O,8.3675624854,-3.3756153734,-0.0397053799
C,5.5957760796,-4.0646565867,-0.3952640798
C,5.9669974345,-4.9805028198,0.7883097738
C,4.0676404007,-4.0880964264,-0.5411900602
C,6.1989823656,-4.6271785939,-1.6980264969
H,7.0475348524,-5.04954805,0.9123717517
H,5.5290002793,-4.6053953141,1.7192481111
H,5.5711045597,-5.9869855067,0.6134965086
H,3.7265514885,-3.4836741258,-1.3873572765
H,3.7426087429,-5.1177774706,-0.7180730474
H,3.5629043903,-3.7328475318,0.3624655392
H,5.8035284548,-5.6319904536,-1.8831509295
H,5.9280863746,-3.9979980781,-2.5524026779

H,7.285473658,-4.687759364,-1.6381447627
C,9.5790963246,-0.8174782297,0.4311196335
C,10.1003950001,-1.6104980488,1.646639191
C,10.3288453273,-1.2613655376,-0.8412716175
C,9.9061709338,0.6637900799,0.6688296741
H,9.5914318808,-1.2930722435,2.5628903313
H,9.9469721458,-2.6810720576,1.5145847952
H,11.1723253706,-1.4242996478,1.7758266233
H,9.9836860093,-0.6935094953,-1.7117367425
H,11.4015231348,-1.0733970603,-0.7211749613
H,10.1816067643,-2.323219928,-1.0358638597
H,10.9882151813,0.7765958639,0.7834978798
H,9.5983696557,1.2933688118,-0.172402982
H,9.4381854483,1.0474864451,1.5812740274
C,-5.1864974359,-1.3021364668,-0.3382771994
C,-5.9220579478,-0.1055726884,-0.5729086296
C,-5.9319611074,-2.4707677825,-0.0229115594
C,-7.2857981875,-0.04234441,-0.5340591017
H,-5.3682426914,0.7860278119,-0.8441559894
C,-7.2966483505,-2.4906935865,0.0528209028
H,-5.3826198241,-3.3661546693,0.227437673
C,-8.0498179057,-1.2589888308,-0.2166690708
O,-9.3008118744,-1.2437698355,-0.174939578
C,-8.0457959606,1.2572819521,-0.8304597832
C,-8.8898667706,1.6621923046,0.3947073243
C,-7.1004202857,2.4248673856,-1.146436104
C,-8.9682610961,1.0622041408,-2.0502329152
H,-9.6060379989,0.8798132843,0.6457263943
H,-8.2456605304,1.8427502469,1.2621399927
H,-9.4355805499,2.5891811378,0.1817841409
H,-6.4889361349,2.2345073691,-2.0343875263
H,-7.693604367,3.3229338187,-1.3450819858
H,-6.4319128049,2.6543895945,-0.3103823299
H,-9.5143504976,1.9902809748,-2.2574872627
H,-8.3803906614,0.8116466253,-2.9400980992
H,-9.6866091596,0.2630540032,-1.8672776164
C,-8.0668987228,-3.7634545771,0.4297368851
C,-8.8881079253,-3.5260632966,1.7124748869
C,-9.0125394915,-4.1657331119,-0.7197309021
C,-7.1313206724,-4.9521029179,0.6939747502
H,-8.2280094193,-3.2783158488,2.5509865649
H,-9.5953269683,-2.7089162766,1.5702872208
H,-9.4437120007,-4.4342244163,1.9762112687
H,-8.4415511598,-4.3785010716,-1.6303174738
H,-9.5679069069,-5.0720567327,-0.4490849831
H,-9.7219700777,-3.365629346,-0.9306044173
H,-7.7312190384,-5.8295653354,0.9561558712
H,-6.5386000841,-5.2126781323,-0.1896420712
H,-6.4457636625,-4.7612913096,1.5262196309
C,-1.5052884968,-1.8258360419,-0.4814517018
H,-3.3870098558,0.7540228621,-0.1241331843
C,5.0127332007,2.2581383296,0.450733631
C,3.463727264,4.1483753293,0.5811176411
C,6.0919175127,3.1113184703,0.6647576754
H,2.4503926392,4.5259539413,0.5458267293
C,5.8488211758,4.4745212513,0.8357714475
H,7.114528943,2.7609880174,0.7036374672
C,4.5545499317,4.9888561274,0.7952151496
H,6.6876185751,5.1431629685,1.0026376422
H,4.3929487843,6.0534091885,0.9302613276
C,-2.8094361439,-2.3847784662,-0.5218054653
C,-2.9811930556,-3.7544278933,-0.7569951563
C,-0.3850469016,-2.659432745,-0.6177414937
C,-1.8660614124,-4.5624325167,-0.8906044966
H,-3.9690206957,-4.1839736281,-0.8574098473
C,-0.5762306766,-4.0153407825,-0.8089403705
H,0.6213878641,-2.2628237274,-0.5859211277
H,-1.9900577766,-5.6251431662,-1.0711948212
H,0.2881992962,-4.6636918667,-0.9144466146

1c$_{\mathrm{NH}}$$^{–}$-3 (closed-shell singlet state)
–2636.8754146 hartree
C,-2.8259819479,6.8820541252,-1.9103852107
C,-2.9379916026,7.1836956616,-0.5583775658
C,-2.1812897259,5.7094675012,-2.3394157054
C,-2.4098290003,6.324361854,0.4202685082
H,-3.4397794826,8.0958107211,-0.2496161708
C,-1.6644732664,4.8708620801,-1.3736094189
H,-2.0879796853,5.4628413644,-3.391748459
C,-1.7785098032,5.1759360193,-0.0081640515
H,-2.4894255057,6.5478747807,1.4788524039
O,-1.0108117248,3.6998934281,-1.5370703482
O,-1.2012506577,4.2068120544,0.7393520501
C,1.3842536659,1.9707186641,-0.3625812757
C,-0.7088951725,0.7697267442,-0.2629385119
C,0.7009012534,0.8011360099,-0.534047445
H,1.2402937923,-0.1074903945,-0.7546092651
B,-0.6749189907,3.2176586177,-0.1858561864
O,-1.307066877,1.9015760438,0.0666689428
O,0.7970189322,3.0992613855,-0.0422336311
C,-1.9102439848,-2.6298108342,-0.5531722516
C,4.9031888844,2.6154271303,0.0380383862
N,3.543704414,2.8422787381,0.2746247905
C,3.7175041794,1.173108163,-1.2977943086
C,-1.4664783645,-0.3831223102,-0.2808894195
N,-0.9682321866,-1.658173932,-0.5896917187
C,2.852490998,2.0151322551,-0.447115393
H,-3.24150761,7.5603500836,-2.649594291
C,5.8377876391,3.3397278553,0.7367773535
C,5.3911679497,4.3764385497,1.6456741173
C,7.2597908628,3.1154572699,0.6193677585
C,6.2403932401,5.1491289113,2.3560476544
H,4.3202658065,4.5029214481,1.7138579929
C,8.1772579095,3.8376803897,1.2970959543
H,7.5879292889,2.3195015777,-0.0279704614
C,7.7035092632,4.9237457363,2.207321296
O,8.517484439,5.6134041145,2.8237110153
C,5.7485510132,6.2490319359,3.3026166207
C,6.2791852634,7.6208652346,2.8396079346
C,4.2157352725,6.3242903913,3.3276766996
C,6.2286494286,5.9626390755,4.7397280444
H,7.3685955026,7.652674721,2.8529710307
H,5.9320046901,7.846853853,1.8260036569
H,5.8997170628,8.404083436,3.504854536
H,3.7619267969,5.3918726516,3.6760245882
H,3.9092651837,7.1176424118,4.0157879255
H,3.7963150735,6.5600582921,2.3454002363
H,5.847949009,6.7371438486,5.4143661157
H,5.8464642021,4.9979065482,5.0895775362
H,7.3167326632,5.9528537021,4.8028003415

C,9.6819223403,3.580848412,1.1687950687
C,10.3876313401,4.8381271385,0.6212022903
C,10.2745062889,3.2060941258,2.5422695793
C,9.9797897673,2.424388243,0.2036689958
H,9.9892966223,5.1083194663,-0.362456487
H,10.2625061539,5.6861037138,1.2936265229
H,11.4585795408,4.637813015,0.5065523309
H,9.7953025862,2.3045294672,2.9381711869
H,11.3449258167,2.9985072308,2.4364973403
H,10.1473558336,4.0141912307,3.2618788658
H,11.0621515516,2.2771877934,0.1463003452
H,9.5380567134,1.4812962899,0.5412880382
H,9.6240561651,2.6334436764,-0.8105671561
C,-1.6245160664,-3.9838790239,-0.858661241
C,-0.2885696051,-4.4675892439,-0.8361112234
C,-2.6371646579,-4.8969291065,-1.2513117219
C,0.0527625915,-5.7521061013,-1.1591274245
H,0.4902486681,-3.795598765,-0.4910954121
C,-2.3801526887,-6.1950367013,-1.6028668451
H,-3.6480805976,-4.5233289996,-1.3280997943
C,-1.0008575258,-6.6911307239,-1.5676442643
O,-0.7266720006,-7.8760713039,-1.8738716211
C,1.5029034882,-6.2485559237,-1.0865209215
C,1.9622828335,-6.7318413873,-2.4766806347
C,2.4750572697,-5.1485433014,-0.6364638196
C,1.61431663,-7.4073923675,-0.076018615
H,1.3203900263,-7.5372757895,-2.8333047395
H,1.9294435742,-5.9091785223,-3.1997723258
H,2.9957514228,-7.0954445111,-2.424812898
H,2.2414698564,-4.7746677752,0.3659280574
H,3.4901071113,-5.5571401188,-0.6055082029
H,2.4856293337,-4.2997939863,-1.3290504919
H,2.6486509312,-7.7695372143,-0.0325731055
H,1.3319588352,-7.0704733629,0.9274644269
H,0.9627724126,-8.2318507189,-0.3656052012
C,-3.4984773747,-7.1456285558,-2.0520075688
C,-3.2320273793,-7.6357764736,-3.4889345419
C,-3.5718368645,-8.3567391509,-1.1007532321
C,-4.8765275756,-6.4682925772,-2.0431647249
H,-3.2183335796,-6.790971822,-4.1864119317
H,-2.2744091124,-8.1534185206,-3.5461304026
H,-4.0265872007,-8.322561663,-3.8053396066
H,-3.8031925227,-8.0303351591,-0.0807454575
H,-4.3650229006,-9.0415536156,-1.4248860785
H,-2.6231194514,-8.8933389652,-1.0900910532
H,-5.6332141445,-7.1900334247,-2.3674566725
H,-5.1590274198,-6.1244544346,-1.0422612161
H,-4.921743313,-5.6143421457,-2.7270545012
C,-2.8551120262,-0.5842378924,0.0041534748
H,-0.039605887,-1.829982969,-0.937229306
C,5.0371152502,1.5533364542,-0.9858996617
C,3.4466859211,0.2419975216,-2.2913413344
C,6.0983920395,0.9632895605,-1.6677634355
H,2.4281799292,-0.002952269,-2.5679903016
C,5.8269726539,0.0038683248,-2.6422733972
H,7.1290654165,1.2340868622,-1.4818528062
C,4.5170292731,-0.351221441,-2.9569627825
H,6.6515949206,-0.4600461522,-3.1738514723
H,4.327930276,-1.084907814,-3.7336445716
C,-3.137429204,-1.9643251831,-0.1499571529
C,-4.4158075767,-2.455643684,0.1513612969
C,-3.8562712754,0.311576283,0.410136039
C,-5.3960296146,-1.5653150098,0.5491180661
H,-4.6372044322,-3.5137973731,0.097304811
C,-5.117549436,-0.1909182258,0.6662275788
H,-3.6270775263,1.3635299537,0.5208737628
H,-6.3899693584,-1.9325205661,0.7839633712
H,-5.9066577186,0.4862247845,0.9788782561

$1c_{NH}^{-}$-4 (closed-shell singlet state)

–2636.8839312 hartree
C,-2.6425242499,6.6876068318,-0.5105191042
C,-2.6474137874,6.8232878988,0.8726664289
C,-2.0775923083,5.5571780484,-1.1250321628
C,-2.0874198872,5.8331654107,1.6981294263
H,-3.0902937015,7.7049874191,1.3260210719
C,-1.5294234118,4.5887262941,-0.3090253438
H,-2.069785604,5.4386806423,-2.2033850935
C,-1.5347193303,4.7265495386,1.0883674879
H,-2.0852296163,5.9259066496,2.7790578654
O,-0.9351637381,3.4287749808,-0.6616550734
O,-0.9439807829,3.6565898921,1.6679480448
C,1.5039140796,1.4923270579,0.1745717768
C,-0.6178300108,0.351510098,0.2241425973
C,0.7871002684,0.3591740286,-0.0644747295
H,1.3074159961,-0.5183777177,-0.4215243065
B,-0.518746695,2.7696753778,0.592689832
O,-1.1587782845,1.4513225099,0.7355028308
O,0.9558381411,2.6162528683,0.6077770858
C,-2.0672297292,-2.8578687526,-0.6111547625
C,5.019265919,0.918859248,-0.3674581892
N,3.7050301602,0.5063601649,-0.1312918929
C,3.7210347617,2.8075338672,-0.2068753778
C,-1.4433908677,-0.7376322663,0.0457714937
N,-1.0521149237,-1.9630357675,-0.5155580249
C,2.9501687492,1.5646885144,-0.0437016397
H,-3.0821002067,7.4642056197,-1.1292303921
C,6.0226032391,-0.0162870191,-0.4741605887
C,5.7238315631,-1.4139160696,-0.2443608569
C,7.3872363425,0.3309282846,-0.791485464
C,6.6623641717,-2.3852608648,-0.277286865
H,4.6924462305,-1.6380334236,-0.0151122099
C,8.3827409624,-0.578649565,-0.8697169092
H,7.603446277,1.3657403755,-0.9948510265
C,8.0650809413,-2.011282404,-0.6014955074
O,8.9533606777,-2.8646247691,-0.6526647836
C,6.3338118474,-3.853243753,0.0124706964
C,7.1498583559,-4.3481470116,1.2239461395
C,4.8479642697,-4.0410981004,0.3452535338
C,6.6533610364,-4.7216187024,-1.2209625436
H,8.2212243216,-4.2973616918,1.0310482381
H,6.9231153626,-3.7500837943,2.1129924641
H,6.8843043039,-5.3878202244,1.4437673351
H,4.1992725131,-3.7467764572,-0.4845652253
H,4.6567878429,-5.0987284476,0.5469975636
H,4.5512071927,-3.4752074153,1.2337332706
H,6.3920322427,-5.7647843846,-1.0133626854
H,6.0653451654,-4.3966349361,-2.0854654373
H,7.7115289779,-4.6736734331,-1.4777540845
C,9.8214832998,-0.1915671016,-1.2259781331

C,10.7709715333,-0.5617464609,-0.0684507616
C,10.2612480665,-0.9172075675,-2.513628145
C,9.9588575602,1.3175499094,-1.4735549697
H,10.4828294027,-0.0402172251,0.850421065
H,10.7628328717,-1.6345193121,0.1214417017
H,11.7933518181,-0.2590060677,-0.3202772255
H,9.6076267035,-0.6507261185,-3.3508667832
H,11.2818130713,-0.6166874358,-2.7751684304
H,10.2401855755,-1.9988686802,-2.3846544069
H,10.998932041,1.5447859448,-1.725020358
H,9.3378920226,1.6564456596,-2.3091034497
H,9.7008667935,1.9049482983,-0.586215134
C,-1.9261098517,-4.1294400534,-1.2130000756
C,-0.6428011543,-4.7059603205,-1.4240736158
C,-3.0496959809,-4.8587082255,-1.6870978758
C,-0.4535732327,-5.9155570318,-2.032144548
H,0.221860591,-4.1787026791,-1.0351550361
C,-2.9478319204,-6.0686905661,-2.3162457526
H,-4.0220512877,-4.3980517873,-1.5944925793
C,-1.6231516944,-6.6680209169,-2.5106232828
O,-1.4898592908,-7.7822821948,-3.0673040131
C,0.9428330767,-6.5239137711,-2.2149704013
C,1.2294787331,-6.7406263399,-3.7144241961
C,2.0489492822,-5.6164770651,-1.6609047708
C,1.0339313643,-7.8720341024,-1.4724982596
H,0.4883292585,-7.4070912361,-4.1551826427
H,1.208708492,-5.7859664082,-4.2515544962
H,2.2258250359,-7.1796621564,-3.8449730978
H,1.9420947697,-5.4434142079,-0.5850807348
H,3.0206784534,-6.0941341473,-1.8198041992
H,2.0765088494,-4.6452422707,-2.1669532763
H,2.0303441603,-8.3087317726,-1.6105712906
H,0.8734006869,-7.7304851546,-0.3980129111
H,0.286854692,-8.5701353176,-1.8499614386
C,-4.1829783841,-6.8119947145,-2.8429785676
C,-4.071183999,-7.0123961841,-4.3672487361
C,-4.304774044,-8.1845328654,-2.1512177826
C,-5.4837506198,-6.0422108788,-2.5724111764
H,-4.0248317765,-6.0451120026,-4.8795391155
H,-3.1764387654,-7.5834822118,-4.6151811747
H,-4.9505950128,-7.5509882732,-4.7407749428
H,-4.4266870728,-8.0600033051,-1.0694998085
H,-5.1835528733,-8.7197074251,-2.5312495136
H,-3.4157856925,-8.7874502036,-2.3361749608
H,-6.328931354,-6.6164445572,-2.965488377
H,-5.6581706632,-5.8939147352,-1.5012253752
H,-5.4943543399,-5.0632071379,-3.0626545226
C,-2.82834649,-0.9011120512,0.3796998355
H,-0.1702089957,-2.1026099451,-0.9813560064
C,5.0499557244,2.4008126034,-0.4276689848
C,3.3509250275,4.1457191527,-0.1877623464
C,6.0335071626,3.3686206807,-0.61893063
H,2.3205856418,4.424337841,-0.0106122002
C,5.6666215756,4.714102713,-0.6013717053
H,7.0743961002,3.1207437429,-0.7763619159
C,4.3441254574,5.100867563,-0.392243852
H,6.4301207324,5.4709499297,-0.7523278072
H,4.0851488223,6.1546376326,-0.3830283219
C,-3.2165999373,-2.2086221367,0.0012257377
C,-4.5032988045,-2.6727930021,0.3080338293
C,-3.7362054157,-0.0377536177,1.011263061
C,-5.3919218821,-1.8152391495,0.9311640134
H,-4.8010427131,-3.6896823894,0.088046982
C,-5.011898386,-0.5039218075,1.2675538353
H,-3.427224684,0.9613701592,1.2886313385
H,-6.3914191231,-2.1627535371,1.1723862959
H,-5.7300427159,0.1502018154,1.7526843867

$1c_{NH}^{-}$-1 (open-shell singlet state)

–2636.8789061 hartree
C,-0.2544026541,5.7195537829,-1.6559307899
C,-0.33630 45544,6.0644919777,-0.3120752393
C,-0.1393113518,4.3766339338,-2.05292 18098
C,-0.306627191,5.0800335363,0.6902998962
H,-0.4226987397,7.10900 46606,-0.0283421549
C,-0.110723699,3.4155936188,-1.0637548091
H,-0.071 1324399,4.0949602905,-3.0983256883
C,-0.1937105475,3.7646125489,0.2930 237533
H,-0.3667089346,5.3355807242,1.7428814477
O,-0.0087885077,2.074 1765126,-1.1950779835
O,-0.1479706758,2.6544595946,1.0665338435
C,1.27 34801692,-0.5445489501,0.216391265
C,-1.1291620998,-0.6491041952,0.005 9998466
C,0.0976085359,-1.2769416281,-0.0717798502
H,0.1643672086,-2.3 101961381,-0.3591331662
B,-0.0344396488,1.5195161309,0.1666898976
O,-1 .2246570642,0.6345293831,0.3296565451
O,1.1887247851,0.7442153138,0.44 46434021
C,-4.680202834,-1.229189954,-0.1946661637
C,4.750489506,-1.17 78015783,0.2596231626
N,3.6647045294,-0.3825849425,0.2696074896
C,2.92 04893428,-2.5637677188,0.3222550674
C,-2.3939461201,-1.2825545205,-0.1 985765525
N,-3.5185353211,-0.5268516911,-0.0394864592
C,2.5586740633,- 1.145243515,0.2685881964
H,-0.277277279,6.4970627774,-2.4135755077
C,6 .0480388068,-0.5961592774,0.1916551742
C,6.1761316545,0.8197258868,0.3 377612624
C,7.2296017741,-1.3547525689,-0.0409159127
C,7.3766806006,1. 4622464892,0.3037995111
H,5.2521175175,1.3603213249,0.4877791407
C,8.4 722995276,-0.7921156626,-0.0987184516
H,7.1242210371,-2.4139074802,-0. 2109044939
C,8.601557014,0.666632495,0.0945898227
O,9.7236654821,1.208 7123151,0.0728975352
C,7.4892546016,2.9807616156,0.4792093337
C,8.1315 262651,3.6098495307,-0.7737326361
C,6.113685628,3.6331323061,0.6754746 774
C,8.3453092606,3.3071611488,1.7195692008
H,9.1372112397,3.22304584 47,-0.9385579456
H,7.5244313386,3.4044223246,-1.6616891104
H,8.1888724 491,4.6974488397,-0.6517707775
H,5.6057679287,3.2640091681,1.571254484 9
H,6.2442510414,4.7135586947,0.7909926681
H,5.4520972813,3.4696715197 ,-0.1799832273
H,8.4047721048,4.3936343248,1.8508592048
H,7.8911441819 ,2.8852330875,2.6224977299
H,9.3557143194,2.9112422282,1.6171838863
C, 9.7307845754,-1.625268231,-0.3655333689
C,10.4299460867,-1.133393271,- 1.6489063098

C,10.7005186518,-1.5136519072,0.8285285575
C,9.4037085549 ,-3.1133688869,-0.5579840331
H,9.7622843429,-1.2260527993,-2.512128123 7
H,10.7347119954,-0.0915895978,-1.5533752196
H,11.3192313093,-1.74391 32776,-1.8435582563
H,10.226958753,-1.8799214984,1.7457357107
H,11.590 5548886,-2.1254495644,0.6411696564
H,11.0107471671,-0.480830313,0.9842 265939
H,10.3318023542,-3.6614199167,-0.7475096618
H,8.940582652,-3.55 06361533,0.3326238103
H,8.7405638636,-3.2828635105,-1.4122340848
C,-5. 9558884421,-0.6322885345,-0.0514876252
C,-6.1021315944,0.7863636327,-0 .0686861491
C,-7.1285440861,-1.4150081994,0.1461342081
C,-7.304801001, 1.4152792547,0.0699594717
H,-5.219084636,1.3877120093,-0.2447886668
C, -8.3698215456,-0.8699928921,0.3052655153
H,-7.0096370419,-2.4844217648 ,0.2257477597
C,-8.5160314161,0.5954099037,0.2607397483
O,-9.636277755 7,1.1287867403,0.3850817834
C,-7.4337800076,2.9418974964,0.0164583254
C,-8.0227780084,3.4639606146,1.3427988871
C,-6.07646642,3.6286210451,- 0.1905909895
C,-8.3486048732,3.3521831595,-1.1551922982
H,-9.013783207 ,3.0482164777,1.523295658
H,-7.3716869873,3.2023409829,2.1836395273
H, -8.1000180188,4.5563114946,1.306464083
H,-5.6053913461,3.3424360667,-1 .1361162092
H,-6.224584378,4.7120023407,-0.2170107026
H,-5.3753297278, 3.4173570571,0.6227804003
H,-8.4262529654,4.4443456029,-1.1966750231
H ,-7.9318617206,3.0096888969,-2.1083913563
H,-9.3482062088,2.9341392797 ,-1.0384523077
C,-9.611268719,-1.7366431097,0.5467396097
C,-10.2534663 496,-1.3764857942,1.9013499118
C,-10.633826113,-1.5171349026,-0.586629 0157
C,-9.2708468047,-3.2336589044,0.5788016158
H,-9.5488118299,-1.550 8679864,2.7214876059
H,-10.5620539007,-0.3316096952,1.9217593667
H,-11 .1335661539,-2.0064985921,2.0737421468
H,-10.2021851252,-1.7928475085, -1.5548505362
H,-11.5144374559,-2.1476326613,-0.4184151225
H,-10.95094 62786,-0.4755770171,-0.6293802736
H,-10.1878956951,-3.8048354513,0.752 2489132
H,-8.8449949102,-3.5788927776,-0.3692209628
H,-8.5720600191,-3 .4817638906,1.384129363
C,-2.836989283,-2.598892596,-0.5203732386
H,-3 .4323599012,0.4409717138,0.2430364318
C,4.3366058864,-2.5871671619,0.3 149826428
C,2.2021445676,-3.7542652474,0.4606028076
C,5.0145153529,-3. 8037246066,0.4297443327
H,1.1223096665,-3.7599697816,0.5393280402
C,4. 2875084554,-4.9826771427,0.5331220912
H,6.0948821668,-3.8524654756,0.4 688108081
C,2.8900958157,-4.9571014387,0.5511378728
H,4.8113395836,-5. 9292368082,0.6233971682
H,2.335440788,-5.8841742313,0.6600123223
C,-4. 262991624,-2.5714630144,-0.52428146
C,-4.9788956379,-3.7257566865,-0.8 893616989
C,-2.15179873,-3.786630412,-0.83811912
C,-4.2872390418,-4.88 00390072,-1.192611001
H,-6.0585954328,-3.7185101607,-0.9593392316
C,-2 .8785212227,-4.9121662675,-1.1589123252
H,-1.0700661246,-3.8250974775, -0.8510459932
H,-4.8350002414,-5.7733770666,-1.4747551692
H,-2.3590900 275,-5.8320746874,-1.4074449285

1c$_{NH}^{-}$-2 (open-shell singlet state)

–2636.8925888 hartree
C,2.5275348587,6.8079824165,1.7847435147
C,2.77337306 23,7.1103617973,0.450628737
C,1.8750188324,5.6182152801,2.1492695552
C ,2.3763763414,6.2345837039,-0.5742109929
H,3.2794748303,8.0356123292,0 .1923735875
C,1.4882817256,4.7628904815,1.1382051103
H,1.6766440323,5. 3715775881,3.1868836228
C,1.7367381105,5.0690275897,-0.2087597633
H,2. 5618636863,6.4582091954,-1.6194412318
O,0.8545462756,3.5735599946,1.23 74051461
O,1.2674512533,4.0820749315,-1.0079975635
C,-1.3694831435,1.7 475334843,-0.2543532341
C,0.7537496201,0.6311776285,-0.0916728328
C,-0 .6204317303,0.5724201543,-0.0297243741
H,-1.1567328625,-0.34555826,0.1 408865391
B,0.6811831387,3.0745162711,-0.1366155417
O,1.3881783979,1.7 81829001,-0.3084367228
O,-0.7562834706,2.8984310234,-0.4458196606
C,3. 7265906526,-1.404682278,0.0429029095
C,-4.7867899025,0.8420820307,-0.2 599755305
N,-3.468842808,0.5747709558,-0.1595395844
C,-3.6836019836,2. 8342030839,-0.5574065829
C,1.6312651623,-0.4898235951,0.0291366505
N,2 .9746788395,-0.2656575396,-0.0384921274
C,-2.7799464787,1.7209449059,- 0.3192958476
H,2.8430000945,7.4994174589,2.5602568257
C,-5.7330215434, -0.2093327116,-0.1061233243
C,-5.2610309907,-1.5487337187,0.0538670575
C,-7.139997015,0.0022339113,-0.0902390278
C,-6.0901808356,-2.61897109 21,0.1999235808
H,-4.187800197,-1.6703456783,0.0464658842
C,-8.0440409 321,-1.0111645743,0.0550521556
H,-7.4985095449,1.0139969948,-0.1792030 301
C,-7.5476784837,-2.3930423266,0.1993517051
O,-8.3439951737,-3.3450 020028,0.3214418169
C,-5.5545120771,-4.0462217353,0.3614725572
C,-6.05 62848749,-4.9337827761,-0.7951290093
C,-4.0194021709,-4.0835645634,0.3 443705658
C,-6.0202457545,-4.6367443324,1.7075225074
H,-7.1448992894,- 4.9852860427,-0.8053668226
H,-5.713669685,-4.5415622454,-1.7586051643
H,-5.6572061098,-5.94860497,-0.6838825431
H,-3.5867437571,-3.498275537 7,1.1618374519
H,-3.6871165764,-5.1196322133,0.4648302304
H,-3.6102210 29,-3.7114138702,-0.5999552161
H,-5.6198009367,-5.6501961212,1.8259819 116
H,-5.6527938353,-4.0304116667,2.5422814961
H,-7.1078647508,-4.6822 589994,1.7602207313
C,-9.5550422264,-0.75413494,0.077278975
C,-10.2352 396368,-1.5031311466,-1.0866104419
C,-10.152605493,-1.227064488,1.4178 418856

C,-9.8859539832,0.7375825912,-0.0765108637
H,-9.8383663837,-1.1 623708925,-2.0488946373
H,-10.0776688967,-2.5782024965,-1.004499359
H, -11.312837861,-1.3027096018,-1.076622933
H,-9.6967467922,-0.6879091198 ,2.2551282273
H,-11.2300446213,-1.0264283722,1.4346669315
H,-9.9931953 261,-2.295408878,1.5616782598
H,-10.9724051295,0.8666974857,-0.0530132 083
H,-9.4653991142,1.3390019414,0.7357576237
H,-9.527789071,1.1419895 175,-1.028815745
C,5.1401399591,-1.3877210577,-0.0207497825
C,5.862930 5368,-0.1700303427,0.1521562841
C,5.890654562,-2.5678160603,-0.2880250 476
C,7.2237708618,-0.0940002237,0.0966833282
H,5.3022382665,0.7272511 997,0.3818990646
C,7.2528916685,-2.5817433261,-0.3721637665
H,5.342583 9245,-3.4762264738,-0.4835428692
C,7.9929431117,-1.3244607184,-0.16764 96935
O,9.2386905555,-1.3007869909,-0.220420683
C,7.97317348,1.2251385 822,0.3170654822
C,8.7996597694,1.573626581,-0.9375145204
C,7.01560196 98,2.3956391616,0.5815022155
C,8.9082235214,1.1019818234,1.5370777728
H,9.5361356943,0.7990511863,-1.1499818964
H,8.1473128407,1.6890724294, -1.8095883111
H,9.322276574,2.5240512435,-0.7819305346
H,6.4159129235, 2.2460813862,1.4847974462
H,7.599606491,3.3089945154,0.727163565
H,6.3 370352336,2.5752861317,-0.2582231352
H,9.4316419334,2.0512000412,1.697 4678194
H,8.3337713481,0.8789415508,2.4425317169
H,9.6473148306,0.3152 312909,1.3875625584
C,8.0324005104,-3.8630193136,-0.6905524298
C,8.840 8709148,-3.6818244586,-1.990802744
C,8.9884660283,-4.1989931494,0.4719 501232
C,7.1022983247,-5.0688687672,-0.8873868839
H,8.1744909122,-3.46 78288867,-2.8332512821
H,9.5567383332,-2.8658372757,-1.8951145397
H,9. 3872250964,-4.6040367631,-2.2193751197
H,8.4277553167,-4.3569024248,1. 3995498023
H,9.5348817582,-5.1221742474,0.2472777601
H,9.7071857898,-3 .3955225536,0.6305146193
H,7.7065931992,-5.9535267843,-1.1099269156
H, 6.5189140971,-5.2893868422,0.0127430248
H,6.4100132952,-4.92531596,-1. 7231537931
C,1.4646622994,-1.8965864545,0.1922964107
H,3.3171472501,0. 6704199199,-0.210853192
C,-4.9830114238,2.2800752954,-0.5268808801
C,- 3.4699464544,4.1918054811,-0.7938499901
C,-6.0824985055,3.1120055077,- 0.7571787636
H,-2.4630994314,4.5894212486,-0.8118610516
C,-5.868396925 1,4.4656009828,-0.9929850123
H,-7.0981954852,2.7389051007,-0.769506883 7
C,-4.576548203,5.0028682003,-1.0076806703
H,-6.7210088096,5.11390709 75,-1.1715864348
H,-4.4380078524,6.0635025643,-1.193560837
C,2.7687260 639,-2.4727437064,0.210425833
C,2.9131393032,-3.8532997722,0.437437871 9
C,0.3262180161,-2.7101868612,0.3431371637
C,1.7844557397,-4.63231666 34,0.5873984031
H,3.8900262768,-4.311475576,0.5175453975
C,0.495708689 1,-4.0646688214,0.5289215415
H,-0.6714755331,-2.2911787024,0.320594981 9
H,1.8902587929,-5.6982697203,0.7616046928
H,-0.3756198763,-4.7009335 847,0.645523947

$1c_{NH}^{-}$-3 (open-shell singlet state)
–2636.8742895 hartree
C,-2.2099485989,6.9446034481,1.7030596052
C,-2.420806 3119,7.3633891291,0.3947536811
C,-1.6704843879,5.6768532841,1.97934883 81
C,-2.1003370161,6.5308240541,-0.6912658151
H,-2.841200251,8.3463927 417,0.2047583779
C,-1.358416501,4.8646148192,0.9087964543
H,-1.5037680 443,5.3375749928,2.9961881041
C,-1.57162656,5.2883135319,-0.4124750167
H,-2.2627504132,6.8436575188,-1.7172923585
O,-0.8342675266,3.61979848 53,0.9210169538
O,-1.1878963557,4.3249466797,-1.2819031461
C,-0.955247 6554,0.7923031961,-0.6200757098
C,1.2581812976,1.7696901822,-0.5943933 811
C,0.4339585053,0.6491631772,-0.4829395541
H,0.8493659581,-0.326274 4624,-0.2897275682
B,-0.6948464332,3.211941365,-0.4867231557
O,0.73665 38737,2.9664297283,-0.806644285
O,-1.4785319599,1.9884574593,-0.754262 8548
C,4.7424742419,0.7417621592,-0.2994758863
C,-3.7818808221,-1.3348 869495,-0.4067942345
N,-3.1592153978,-0.1330503131,-0.4415295401
C,-1. 5524554024,-1.7521922064,-0.7694685555
C,2.6714472428,1.7051459556,-0. 5384231199
N,3.3873556071,0.547398029,-0.3166869734
C,-1.8492202022,-0 .3219738261,-0.6131828473
H,-2.4667946199,7.6033567776,2.5272329208
C, -5.1749442986,-1.3913598324,-0.1657055718
C,-5.9180387142,-0.167241195 8,-0.1266916234
C,-5.8795346984,-2.6105252444,0.0633681352
C,-7.261611 4428,-0.1233541064,0.079753861
H,-5.3421224266,0.7341933896,-0.2817298 247
C,-7.2230950832,-2.6585960095,0.2859319965
H,-5.3103314234,-3.5250 914485,0.0892840927
C,-7.9901426949,-1.3925477368,0.2845082974
O,-9.22 1261277,-1.4036769635,0.4595482742
C,-8.0371232914,1.198353085,0.10259 44187
C,-8.7265420804,1.381470909,1.4698028437
C,-7.111067218,2.402514 3217,-0.1179478723
C,-9.0964345438,1.2085937909,-1.0179594022
H,-9.440 6929637,0.5810882175,1.6635500524
H,-7.9854522489,1.3960334993,2.27587 52668
H,-9.2592404839,2.3388999252,1.4879481213
H,-6.60890274,2.365246 6833,-1.0890493826
H,-7.7061971613,3.3203666176,-0.0889655254
H,-6.343 4140737,2.482129444,0.6570383614
H,-9.631517647,2.1649109767,-1.009782 8944
H,-8.6208168731,1.0997886322,-1.9984808874
H,-9.8189079762,0.4029 707267,-0.887027922
C,-7.9594245899,-3.9775837902,0.5438321318
C,-8.6374875905,-3.9440342743,1.9283934322
C,-9.0220947085,-4.2113254201,-0.5 492145925
C,-7.0041737605,-5.1798879907,0.5250310804

H,-7.8940480634,- 3.7990873082,2.7194141408
H,-9.3705131839,-3.139932686,1.9864675575
H, -9.1457856381,-4.8969920389,2.1144802872
H,-8.55446984,-4.2591490334,- 1.5384735348
H,-9.531950839,-5.165046654,-0.3711670076
H,-9.7632914668 ,-3.4127286291,-0.5515741675
H,-7.5758146226,-6.0931645704,0.716105683 5
H,-6.5120737119,-5.2984520352,-0.4458863145
H,-6.233095457,-5.106417 2645,1.2985637904
C,5.6531239948,-0.311143131,-0.0619521081
C,5.240902 0695,-1.6720988155,-0.1676833259
C,7.0013189196,-0.0641362962,0.322764 6211
C,6.0724737943,-2.7284180284,0.0637180871
H,4.2292904259,-1.87383 91595,-0.4994769995
C,7.8975274823,-1.0599673806,0.5854192396
H,7.3009 356243,0.9633177487,0.4623444508
C,7.4679484713,-2.4617099727,0.457346 7486
O,8.2610261543,-3.400345859,0.676100884
C,5.60588047,-4.180705090 2,-0.0928664349
C,5.752838777,-4.9264139284,1.2488246896
C,4.131948952 3,-4.2731758928,-0.5138499212
C,6.4437545613,-4.8905717905,-1.17514299 22
H,6.7912755368,-4.9317098144,1.5789037419
H,5.1397199863,-4.4527096 722,2.022986223
H,5.4122474435,-5.9621099928,1.136839057
H,3.948978121 6,-3.7987039311,-1.483706553
H,3.8531177448,-5.3270145347,-0.607718326 9
H,3.4620599166,-3.8242119234,0.2270300556
H,6.1040379174,-5.92656775 7,-1.2873180064
H,6.3267503662,-4.3913230785,-2.1430632627
H,7.5003421 524,-4.8948723707,-0.9085695318
C,9.3350590894,-0.7597546585,1.0276954 733
C,9.5987983043,-1.3671772539,2.4198969183
C,10.3317599667,-1.34833 58495,0.0088377085
C,9.6065670115,0.7486163859,1.1213960817
H,8.918317 5128,-0.9379813139,3.163157863
H,9.4638039147,-2.4484660057,2.40489156 91
H,10.6249940886,-1.1447046683,2.734455349
H,10.1784945079,-0.904652 068,-0.9809117643
H,11.3584075528,-1.1263962641,0.3224368882
H,10.2143 497451,-2.4288202278,-0.070213639
H,10.642389691,0.9066210439,1.437039 8036
H,9.4785350908,1.2500860976,0.1562586047
H,8.9607302442,1.2403414 612,1.85594723
C,3.6404001533,2.7357224041,-0.7121766689
H,2.962753481 9,-0.3231403426,-0.0417445144
C,-2.798706792,-2.4079896124,-0.63258099 32
C,-0.4063937225,-2.4828559503,-1.0853091448
C,-2.8772195451,-3.7927 347448,-0.7923817942
H,0.5443511258,-1.9977854447,-1.26668927
C,-1.721 7170992,-4.5137385925,-1.0707272467
H,-3.8182916621,-4.3230138149,-0.7 309141023
C,-0.4953083331,-3.8631886324,-1.219606649
H,-1.7796126792,- 5.5905504466,-1.1944310401
H,0.3942499022,-4.4360577258,-1.4623584287
C,4.9275634574,2.1488581999,-0.5757782901
C,6.072544616,2.9366691701,- 0.7826171622
C,3.4939482045,4.1035508096,-1.0043637039
C,5.9153462044, 4.2789247433,-1.0676618666
H,7.0666011012,2.5107791315,-0.7436569825
C ,4.6351016685,4.8602484038,-1.1674556669
H,2.5036786869,4.529558134,-1 .0987583816
H,6.7943405485,4.8956859155,-1.2263312452
H,4.5474666462,5 .9189634448,-1.3897945054

1c$_{NH}^{-}$-4 (open-shell singlet state)

–2636.8862741 hartree
C,0.595518443,8.6345839077,1.9358029209
C,0.455735248 9,9.1050773963,0.6355494678
C,0.5551680612,7.2582677959,2.2161544554
C ,0.2699210132,8.2174272216,-0.4381925597
H,0.4893006737,10.1730849131, 0.4424072854
C,0.3727619306,6.3923128869,1.1576345876
H,0.6620814658,6 .8790006151,3.2268765547
C,0.2317244983,6.8683393971,-0.1555029324
H,0 .1581652974,8.5709147372,-1.4577994415
O,0.2997422418,5.0440062124,1.175890059
O,0.0657898722,5.834985508,-1.0134521786
C,-1.2320610242,2.55 95746989,-0.2893763662
C,1.1632555718,2.440319728,-0.5132435476
C,-0.0 442309867,1.7952942036,-0.3227995364
H,-0.1344770075,0.7202837899,-0.2 554905179
B,0.1026645778,4.6135735809,-0.219372197
O,1.2223049387,3.75 68492701,-0.6617327972
O,-1.1723349634,3.8743876603,-0.3507264186
C,3. 9324419908,0.0580563058,-0.4300305322
C,-4.0097087599,0.3767843929,-0. 1167446577
N,-2.6928179614,0.6323574,-0.2471362104
C,-3.7866253941,2.6 586127943,-0.0752232681
C,2.4242625885,1.7734347879,-0.6023842557
N,2. 6178575742,0.443731403,-0.3247421263
C,-2.5127995298,1.969616461,-0.21 00413239
H,0.7375660952,9.3382812125,2.7505245452
C,-4.495322498,-0.96 16696482,-0.1206462494
C,-3.6612562261,-2.0113236946,-0.60825756
C,-5. 7946328095,-1.3026030494,0.3489338312
C,-4.0750539417,-3.3076718252,-0 .6914190139
H,-2.6788807655,-1.7136243121,-0.9469466694
C,-6.276484049 4,-2.5805021114,0.3378681489
H,-6.3948973274,-0.5141772702,0.775214131 9
C,-5.4250221885,-3.6544891525,-0.2080578555
O,-5.8376563019,-4.83059 71671,-0.2521050162
C,-3.1875366393,-4.4093719917,-1.280399409
C,-3.88 05798652,-5.0531685474,-2.4983323342
C,-1.8379541605,-3.8552283617,-1. 7559856183
C,-2.9044369413,-5.487621855,-0.2152452514
H,-4.8258903987, -5.5151355137,-2.2147562509
H,-4.0738856632,-4.3030712551,-3.272715138 1
H,-3.2289503542,-5.8213693513,-2.9300797233
H,-1.2675648623,-3.40735 37511,-0.9374326952
H,-1.238291292,-4.6738512304,-2.1654648621
H,-1.95 78593095,-3.1042675384,-2.5429770542
H,-2.2570182846,-6.2645687169,-0. 6376356912
H,-2.388167909,-5.0522419613,0.6468486152
H,-3.8290239986,- 5.9515786278,0.1280456643
C,-7.6653798065,-2.9313742665,0.882867964
C, -8.5340104411,-3.5383512254,-0.2377083677
C,-7.5395705574,-3.938064980 3,2.0440862396
C,-8.4003160741,-1.6943463696,1.4192042959
H,-8.6522899 519,-2.8271890684,-1.0623017221

H,-8.0872927618,-4.4529836846,-0.62651 59697
H,-9.5316522398,-3.7708495497,0.1522629674
H,-6.9440184523,-3.51 42595745,2.8596722358
H,-8.534216533,-4.1727573068,2.4402283231
H,-7.0 685428076,-4.8626000881,1.7111465261
H,-9.3817295419,-1.9977177297,1.7 965271592
H,-7.8618138618,-1.219812084,2.2454915131
H,-8.5665184458,-0 .9445255923,0.6389367249
C,4.3769282543,-1.2441194408,-0.1058020461
C, 3.4621887051,-2.3311602339,0.0213158629
C,5.7530289537,-1.5077101332,0 .1534658143
C,3.8479049924,-3.5968843423,0.3550949116
H,2.4180003647,- 2.148802815,-0.2030127864
C,6.2251777049,-2.7394908988,0.5010654253
H, 6.4347608895,-0.6722419847,0.1249047738
C,5.2774606908,-3.8612759986,0 .6074944916
O,5.6716497778,-5.0052658854,0.9077925507
C,2.8472662966,- 4.7529191182,0.461278528
C,2.8657263338,-5.3329619942,1.890183558
C,1. 4123453669,-4.3002953701,0.1629530175
C,3.2064677779,-5.8578538401,-0. 5528584147
H,3.8524093431,-5.719369176,2.1439788377
H,2.5884156551,-4. 5665242569,2.6217650052
H,2.1373789517,-6.1478799939,1.9662254485
H,1. 3048989731,-3.9090278039,-0.8533577776
H,0.7380223757,-5.1565507173,0. 2523007978
H,1.0635301976,-3.5385265806,0.8680955844
H,2.479449902,-6. 6743804413,-0.4808656657
H,3.1729893328,-5.4683914046,-1.5760237497
H, 4.2021121102,-6.2571897067,-0.3617898435
C,7.7082201658,-2.9840051397, 0.8036267753
C,7.8776039899,-3.4898037173,2.2501565253
C,8.2798949198, -4.0251631899,-0.1799301096
C,8.5435483283,-1.7037110873,0.6592937368
H,7.4997272131,-2.7505775796,2.9645104579
H,7.3432747726,-4.4274038371 ,2.401440819
H,8.9401406381,-3.6522690741,2.4641821115
H,8.1919015138, -3.6700058656,-1.2123693633
H,9.3429959312,-4.1889869407,0.0304723716
H,7.7542094292,-4.975469095,-0.0911922584
H,9.5898690144,-1.9327800718 ,0.883046965
H,8.5071561142,-1.3016163661,-0.3586333104
H,8.2231923037 ,-0.9200712643,1.3532429542
C,3.6964644086,2.2951761359,-0.9647551597
H,1.903012077,-0.1422640599,0.0778302003
C,-4.7597720055,1.6382215011, -0.0115599959
C,-4.1422236799,4.0072269861,-0.0281829804
C,-6.11302696 35,1.9803169616,0.0598097375
H,-3.3806518445,4.774449784,-0.0802855042
C,-6.4648806084,3.3241888375,0.1072859962
H,-6.8947352321,1.231229855 6,0.0578772343
C,-5.4895775568,4.3279081577,0.0720629309
H,-7.51422122 55,3.597659544,0.1647485794
H,-5.7911100793,5.3702344616,0.1099949852
C,4.6372838573,1.2320239005,-0.8817066743
C,5.9671295268,1.4541595196, -1.2849381225
C,4.0875990065,3.5798435834,-1.3895438277
C,6.3353356453 ,2.7168605601,-1.7016796304
H,6.6953907558,0.6539480817,-1.2987732054
C,5.4043025032,3.776570695,-1.7422887171
H,3.356874587,4.3765574107,-1 .4328204995
H,7.3595823292,2.896202442,-2.0133026813
H,5.7299270212,4. 7585511784,-2.0702905618

1c$_{NH}$$^{-}$-1 (triplet state)

–2636.8745074 hartree
C,-2.5932778725,6.6328148357,-1.5100250087
C,-2.7862344326,6.9248838197,-0.164910074
C,-1.9355014204,5.4569875049,-1.908460403
C,-2.3293282305,6.0527470139,0.8375990467
H,-3.2945448969,7.8411564447,0.1196076811
C,-1.4898794415,4.6057716197,-0.9189030158
H,-1.7744416433,5.2200066069,-2.9547231821
C,-1.6851986526,4.9009163698,0.4386780736
H,-2.46949978,6.2706295175,1.8909786879
O,-0.8390123428,3.427277401,-1.050704343
O,-1.1651915008,3.9177731939,1.2115676658
C,1.4418944745,1.586325813,0.3510767965
C,-0.7068796357,0.5009910372,0.1810960142
C,0.6518302092,0.4257547292,0.0699942897
H,1.1294544483,-0.4893858652,-0.2285071536
B,-0.6096207913,2.925700677,0.3095258222
O,-1.3257855371,1.6277230273,0.4952865278
O,0.8282702928,2.719481402,0.5653486807
C,-3.745021358,-1.4472607969,-0.0409656488
C,4.8550244758,2.4505043464,0.4454389829
N,3.5500205855,2.726697773,0.4455005284
C,3.7654175315,0.4318257703,0.4534912423
C,-1.6281714857,-0.6062091745,-0.0023213018
N,-2.9535631565,-0.3405977013,0.1019976378
C,2.851448832,1.5632273163,0.4177640239
H,-2.9522223882,7.3230068547,-2.2674909374
C,5.8103427561,3.5273665668,0.4141058059
C,5.3597006467,4.8531565813,0.6689865517
C,7.1812678561,3.3310243876,0.1114495001
C,6.1970618673,5.931931929,0.6744878935
H,4.3024243932,4.9598270467,0.8681722521
C,8.0893932779,4.3562660689,0.0860884746
H,7.5035740255,2.334012286,-0.1443225759
C,7.6265154559,5.7203049028,0.3922662788
O,8.4331120155,6.6762208475,0.4030475232
C,5.6907287705,7.3483755726,0.9688662932
C,5.9710318569,8.269134198,-0.236063608
C,4.1779808375,7.3694370397,1.2292081641
C,6.3908390839,7.9095680744,2.2229917228
H,7.0397134376,8.3360975439,-0.4400288248
H,5.4635611435,7.8957252821,-1.1317627561
H,5.5881519724,9.2750030859,-0.0287361769
H,3.9007341628,6.7651595794,2.0979678932
H,3.8644648114,8.3990245283,1.428443309
H,3.6052646284,7.0091644342,0.3699110861
H,6.010484917,8.9145666349,2.4394338116
H,6.1841771008,7.2782975215,3.0938870157
H,7.4698204344,7.9662905654,2.0790459811
C,9.5645450242,4.1252957819,-0.2600339084
C,9.9509104903,4.945782125,-1.5072158455
C,10.4561637049,4.5390815842,0.9284834817
C,9.8589600381,2.6496642398,-0.5673194214
H,9.3419318119,4.6494485728,-2.367951058
H,9.8114102721,6.0120943129,-1.3312112694

H,11.0021883649,4.7643227006,-1.7594364966
H,10.2104081217,3.9497763405,1.8184697798
H,11.5089379835,4.3564569475,0.6832181439
H,10.3274522417,5.5954632559,1.1626176293
H,10.9207236736,2.5402651166,-0.8094476986
H,9.6490952288,2.0017190275,0.2898628309
H,9.2854849018,2.2844117812,-1.4250368682
C,-5.1654798028,-1.3783359581,0.0476367895
C,-5.8419645376,-0.1325645913,-0.0842068306
C,-5.9462741467,-2.5399289388,0.2955517009
C,-7.2001737545,-0.0102960841,-0.0043594488
H,-5.251266217,0.7484893265,-0.3002883286
C,-7.308652441,-2.5106756161,0.398469572
H,-5.4226879225,-3.4696454229,0.4568222924
C,-8.004866956,-1.2219291436,0.2386690152
O,-9.2482781804,-1.1578809127,0.3108984848
C,-7.9053175841,1.3392351966,-0.1766290633
C,-8.7001717138,1.6803604726,1.1004928508
C,-6.9098234858,2.4816196701,-0.4235352917
C,-8.8617356868,1.2823340208,-1.3851740798
H,-9.4650185866,0.9306241042,1.3012519921
H,-8.0324396223,1.7440587311,1.9660796058
H,-9.1856944603,2.6549572401,0.9805272231
H,-6.3290625979,2.3367840059,-1.3397280054
H,-7.4625180176,3.4186473954,-0.5351953051
H,-6.2135133064,2.6145024522,0.4101484804
H,-9.3488136264,2.2551546319,-1.5133751151
H,-8.3098379837,1.0608666496,-2.3048292203
H,-9.6300840709,0.5224147761,-1.2450019966
C,-8.1270055202,-3.7726660347,0.6920234115
C,-8.9115689919,-3.6006995188,2.0082952926
C,-9.1084962767,-4.0429791666,-0.4666171838
C,-7.2347750777,-5.0133692593,0.8423328411
H,-8.2281198659,-3.4282544915,2.8464633128
H,-9.6055383568,-2.7630561505,1.9449548328
H,-9.4806791771,-4.5126704104,2.2205889337
H,-8.5661713161,-4.1885875282,-1.4069768388
H,-9.6782416127,-4.9563481914,-0.2619277996
H,-9.8062182153,-3.2153298186,-0.5901681964
H,-7.8654875112,-5.8831765426,1.0486461097
H,-6.6708364915,-5.2278313408,-0.0713949198
H,-6.527613502,-4.9164030696,1.6721583779
C,-1.5100031585,-1.993857065,-0.2608285386
H,-3.2576205093,0.5960698526,0.3381414662
C,5.0648833547,1.0039465405,0.4731238806
C,3.617544612,-0.9582220733,0.5511366666
C,6.1914060117,0.1802322562,0.5852221067
H,2.6404087327,-1.4224998558,0.6002675319
C,6.0273426017,-1.1948079599,0.6536508452
H,7.1901521354,0.5935922072,0.6489277015
C,4.7452062889,-1.7596427762,0.637363321
H,6.8984954501,-1.8367819556,0.7418253799
H,4.6313467381,-2.8368642794,0.7158874018
C,-2.8375226557,-2.5268463396,-0.2912619417
C,-3.0310073646,-3.8891335059,-0.6023744046
C,-0.4014768809,-2.8369011249,-0.4897476708
C,-1.933562374,-4.6890275288,-0.8243877594
H,-4.024801354,-4.3090583789,-0.6902626388
C,-0.6209098823,-4.1665993709,-0.7582376028
H,0.6097272041,-2.451217564,-0.4663589115
H,-2.0762266558,-5.7377902122,-1.0645051794
H,0.2235636394,-4.8241646079,-0.9372810477

1c$_{NH}$$^{-}$-2 (triplet state)

–2636.8881846 hartree
C,-2.7448665283,6.9632632627,-1.1600896476
C,-2.8655270113,7.1303072133,0.2144938241
C,-2.1176238831,5.8276070683,-1.6993271886
C,-2.3634369515,6.1680180945,1.1069206541
H,-3.3536448916,8.0165179252,0.608473924
C,-1.6274808562,4.8870857977,-0.8172976235
H,-2.0154828673,5.6867032567,-2.7700492248
C,-1.7494379793,5.0563358184,0.5701260168
H,-2.4497742771,6.2864621011,2.1817793395
O,-0.9934879417,3.7243971898,-1.0912365976
O,-1.1958086602,4.0051306037,1.2205742488
C,1.3888054205,1.807614194,0.0217595253
C,-0.7279337178,0.6732633523,-0.0557528818
C,0.6201561985,0.6367112964,-0.251676417
H,1.1442340935,-0.2533033963,-0.5550703919
B,-0.6869932017,3.1003192399,0.2026916306
O,-1.3603266347,1.7820645201,0.3117723579
O,0.7769731576,2.9177867252,0.3563090823
C,-3.7110082495,-1.3705113189,-0.1673597648
C,4.780844627,0.9238228071,-0.3144373815
N,3.4676230902,0.660117465,-0.3316406003
C,3.7158376269,2.8778638892,0.2412795772
C,-1.6236803978,-0.4577024594,-0.1970377571
N,-2.9533832958,-0.2357309297,-0.0521971355
C,2.793140543,1.7962618855,-0.018502084
H,-3.1394184755,7.7204360753,-1.8308663531
C,5.7178111079,-0.1142707118,-0.6420029938
C,5.2498571711,-1.4531659475,-0.772563922
C,7.0964134568,0.1319495136,-0.866497187
C,6.0684524894,-2.5025078428,-1.0742219576
H,4.1919555362,-1.5984492976,-0.6085658958
C,7.9884570925,-0.8591873544,-1.1786897252
H,7.4401315976,1.1523777119,-0.8160273948
C,7.5028597919,-2.2448934238,-1.2831047104
O,8.2910967915,-3.1788766919,-1.548788468
C,5.5386826116,-3.9358370823,-1.1964826713
C,6.2211030061,-4.8430694197,-0.1531654374
C,4.0237542879,-4.0066545777,-0.9548996902
C,5.8104364328,-4.4805616058,-2.6131960843
H,7.2999361336,-4.8707315355,-0.305580875
H,6.0183836817,-4.4836677878,0.8613766008
H,5.827423963,-5.8629869804,-0.2340089026
H,3.4655949052,-3.4084231978,-1.6819957315
H,3.695237624,-5.0460601122,-1.0561119508
H,3.7517668601,-3.6688523008,0.049805903
H,5.4138985277,-5.4987388905,-2.7014063546
H,5.3139004577,-3.8597584809,-3.3665659231
H,6.8791101747,-4.50125037,-2.8259342222
C,9.4718688184,-0.5650849347,-1.4290419569
C,10.3397216826,-1.3242156051,-0.4047429939
C,9.8628476731,-0.9936764201,-2.8577834983
C,9.7932658894,0.9301313845,-1.2897498974
H,10.0909500942,-1.0145466503,0.6159867422
H,10.1913099196,-2.4004116627,-0.4895077451
H,11.3985817007,-1.0985727087,-0.5767220246

H,9.2714569739,-0.4468548077,-3.5999419645
H,10.9203891808,-0.7672253988,-3.0364736933
H,9.703800682,-2.0620420776,-3.0021104041
H,10.8600244551,1.085198741,-1.4789358101
H,9.2386628226,1.5402498418,-2.0095985425
H,9.5804244622,1.304089559,-0.2830777375
C,-5.1280501114,-1.3491686364,-0.0367614984
C,-5.8499468079,-0.1257043486,-0.1391673743
C,-5.8643080538,-2.5368846737,0.2255093841
C,-7.2081428344,-0.0488663244,-0.0182725746
H,-5.2956952099,0.7760992921,-0.3659507978
C,-7.2230034522,-2.5527045418,0.3693138439
H,-5.3068573494,-3.4503231644,0.3638219071
C,-7.9658307289,-1.2871475029,0.2399434611
O,-9.2081826055,-1.2637068621,0.3499384291
C,-7.9621694336,1.2778250244,-0.1597846222
C,-8.7289297694,1.5851264223,1.1427539465
C,-7.0128619867,2.4542099629,-0.4282501456
C,-8.9521822918,1.1970845207,-1.3394699459
H,-9.4610963068,0.8082422256,1.3613272517
H,-8.0376322474,1.6664913137,1.9881545444
H,-9.2509402241,2.5432244473,1.044055369
H,-6.4556162035,2.3338127859,-1.3624883135
H,-7.5994820315,3.3730526382,-0.5165072287
H,-6.2964555624,2.6045098817,0.3852645295
H,-9.4753346309,2.153770341,-1.4459911994
H,-8.4211894055,0.9997044052,-2.2767767861
H,-9.6898095941,0.4106644881,-1.1816121651
C,-7.9905103362,-3.842828472,0.6782871755
C,-8.7397281826,-3.7046796524,2.0188590874
C,-8.9977488931,-4.1378801591,-0.4518236299
C,-7.05443149,-5.0547452414,0.7924230681
H,-8.036755172,-3.5157708955,2.8371169731
H,-9.4613537096,-2.8890476607,1.9823779485
H,-9.2729643912,-4.6356794076,2.2417136497
H,-8.4797860234,-4.2607707535,-1.4090105294
H,-9.5318234468,-5.0701397923,-0.2361703523
H,-9.7246177739,-3.332010019,-0.5486329477
H,-7.6500228777,-5.9460445517,1.011238561
H,-6.51184492,-5.2447555806,-0.1394444353
H,-6.3257387596,-4.9401832029,1.6011122567
C,-1.4650016399,-1.8432268956,-0.4481481583
H,-3.283475893,0.6889053841,0.1940777695
C,5.0064540269,2.3243483463,0.0605207262
C,3.5415628151,4.2131483002,0.6175763223
C,6.1322537072,3.1246218554,0.2941971804
H,2.5451818662,4.6166229471,0.7494767136
C,5.9540155095,4.4488812319,0.6687149401
H,7.139703569,2.737200238,0.2089095835
C,4.6695364336,4.9916428017,0.822708339
H,6.8244313468,5.0723466568,0.8505160422
H,4.5590633189,6.0314954821,1.1154412635
C,-2.7732177012,-2.4207570985,-0.4388380297
C,-2.9262266214,-3.7913225189,-0.7344761602
C,-0.3315048738,-2.6482466184,-0.6898296639
C,-1.8060045178,-4.5540690019,-0.9758342975
H,-3.9058848209,-4.2476645237,-0.7944592058
C,-0.5109538356,-3.9875490282,-0.9411285251
H,0.6663343408,-2.2285496064,-0.6809444259
H,-1.9175952834,-5.6093895006,-1.20356463
H,0.3526993677,-4.6174827901,-1.1275560363

1c$_{NH}^{-}$-3 (triplet state)

–2636.8694381 hartree

C,-2.542507179,6.7789038877,-1.0107560992
C,-2.7206990169,6.9388157315,0.3583832127
C,-1.8837156109,5.65099678,-1.5282548026
C,-2.2472992586,5.9769091412,1.2667772942
H,-3.2298010934,7.8204746382,0.7358474818
C,-1.4217159028,4.7111970755,-0.6306722642
H,-1.7344611956,5.5166038987,-2.5942652868
C,-1.6019544354,4.8729858702,0.751348295
H,-2.3755225607,6.0913767054,2.3378581206
O,-0.7659553936,3.5556003706,-0.8829326207
O,-1.0673022315,3.823702301,1.4197417341
C,1.5443759728,1.6140839396,0.289264981
C,-0.6059231565,0.5315302694,0.0676514859
C,0.7546370825,0.4837943752,-0.08930393
H,1.2425613059,-0.3872572519,-0.4942327794
B,-0.5184172645,2.926467557,0.4196927012
O,-1.2253268467,1.612520689,0.4868345651
O,0.9258214223,2.7101977406,0.6372475872
C,-2.031672284,-2.7436884268,-0.7396892178
C,4.9513014706,2.5015469055,0.4092807146
N,3.6438554566,2.7595205759,0.4666646261
C,3.8816442202,0.4819921547,0.2079301247
C,-1.4824710258,-0.5980249877,-0.1838936264
N,-1.0237485942,-1.8391615305,-0.5032019168
C,2.9562950685,1.5985410631,0.3215087999
H,-2.9138526858,7.5367227357,-1.6940174418
C,5.894659133,3.5873136109,0.4832562899
C,5.4349975327,4.8691410796,0.8969916369
C,7.2611128293,3.4450145739,0.1342623275
C,6.2604451093,5.9512876709,1.0098475209
H,4.3809328939,4.9384726133,1.1272717553
C,8.1574281403,4.4783309398,0.2095575867
H,7.5891971045,2.4882151655,-0.2401984407
C,7.6859867778,5.7922592355,0.6781846135
O,8.4820361409,6.7515166157,0.7826480304
C,5.7445470018,7.3180569899,1.4736726101
C,5.9893112121,8.3734922792,0.3762311783
C,4.2374217397,7.2889646709,1.7640777605
C,6.4643625348,7.7424593858,2.769687854
H,7.0526600572,8.4771845816,0.1600792071
H,5.467022016,8.0977057812,-0.5459347654
H,5.6001068565,9.344168935,0.7043843833
H,3.9852067109,6.5861890788,2.5636187706
H,3.916630348,8.2849463617,2.0852796314
H,3.65098236,7.0208851328,0.8806782587
H,6.077423342,8.7111393123,3.1065786578
H,6.2830314818,7.0135417227,3.5668583471
H,7.5393904459,7.8296739754,2.6121058633
C,9.6278640634,4.3075510239,-0.1878534811
C,9.9806850039,5.2698945375,-1.3399566569
C,10.538117528,4.5944961771,1.0236828975
C,9.9321030688,2.8805849776,-0.6672514736
H,9.357910282,5.0659256523,-2.2174970918
H,9.8334528931,6.307296443,-1.0406832382
H,11.0285899259,5.1317727061,-1.6307112773
H,10.3165831452,3.9039625523,1.8445555174

H,11.5878184863,4.455210988,0.7398545916
H,10.4022002177,5.6153325915,1.379698742
H,10.9900075586,2.8133380666,-0.9396808135
H,9.7461969586,2.1362858622,0.1138371667
H,9.3459046436,2.6082258641,-1.5505132122
C,-1.7599200722,-4.0914171307,-1.0995070547
C,-0.4716290838,-4.6605537401,-0.8844647145
C,-2.752646425,-4.9071448403,-1.709599349
C,-0.1564571567,-5.9455963094,-1.22070985
H,0.2773894928,-4.0579267906,-0.385992919
C,-2.5245309806,-6.2002642468,-2.0860092374
H,-3.7082648965,-4.4539551681,-1.9234105506
C,-1.1950541191,-6.7873143374,-1.8441792432
O,-0.9515507509,-7.9683853106,-2.1604923315
C,1.2302870918,-6.5400879991,-0.9524048774
C,1.8740495029,-6.9880309189,-2.2804548987
C,2.1768506474,-5.5229899356,-0.2984756559
C,1.1108535448,-7.7461760534,0.0011457085
H,1.2729046478,-7.7537525114,-2.7697178293
H,1.9857864655,-6.1386656362,-2.9626079646
H,2.8722686851,-7.3953508622,-2.0862248555
H,1.8106805618,-5.1818221982,0.6751377937
H,3.1486623075,-5.9965139765,-0.1323687319
H,2.3468315042,-4.6481139227,-0.9346109849
H,2.1064558395,-8.1572483874,0.2010826218
H,0.6754180995,-7.4410729021,0.9586051706
H,0.4901322085,-8.5297010612,-0.43250835
C,-3.6039339871,-7.0486674365,-2.7671682164
C,-3.1253802093,-7.4913090995,-4.1645199569
C,-3.9159060039,-8.2902288571,-1.9069568207
C,-4.9149466209,-6.2704793236,-2.9502323635
H,-2.9195154576,-6.6210429489,-4.7966659052
H,-2.2215701564,-8.0961490506,-4.096678636
H,-3.9077918895,-8.0833608038,-4.6522165874
H,-4.2779989881,-7.9936785061,-0.9167461051
H,-4.7009397688,-8.8843410267,-2.3879861827
H,-3.0311894565,-8.9140423841,-1.7833934788
H,-5.6493145367,-6.9188677319,-3.4372070988
H,-5.3424935318,-5.9534446121,-1.9933807313
H,-4.7868761139,-5.3866881586,-3.5831407748
C,-2.8940315849,-0.6796016024,-0.1758702235
H,-0.0471985664,-2.0527125243,-0.6303802281
C,5.1758824935,1.0645485032,0.2664912237
C,3.7532108676,-0.9122578783,0.1426299154
C,6.3131830081,0.2478817075,0.258080005
H,2.7830786815,-1.3937619573,0.1534804257
C,6.1661984771,-1.1276475207,0.1676076343
H,7.3085241059,0.6644181495,0.3484576211
C,4.8903150818,-1.7038810522,0.1104871616
H,7.0459243695,-1.7638003494,0.1611324823
H,4.7895339489,-2.7841480985,0.0615715663
C,-3.2476221051,-2.0191613109,-0.5211955359
C,-4.6078968492,-2.3936994106,-0.5254086603
C,-3.8857863059,0.2831726604,0.1121601427
C,-5.5599839609,-1.4413579884,-0.2394413587
H,-4.9134295862,-3.4122362245,-0.7274613424
C,-5.2025328579,-0.1065578312,0.0675349651
H,-3.5943044471,1.2936096897,0.3671065062
H,-6.6087854695,-1.7214510539,-0.2412376226
H,-5.983216482,0.6154432572,0.2838549967

1c$_{NH}$$^{-}$-4 (triplet state)
–2636.8822643 hartree
C,-2.8195555931,6.6964090753,-0.9395670064
C,-2.8806745354,6.8633361316,0.4388701884
C,-2.1885552593,5.57611579,-1.5061247612
C,-2.3129897671,5.9159366785,1.3078748036
H,-3.3723439748,7.7377877732,0.8543106844
C,-1.6339522189,4.6501307891,-0.6472477732
H,-2.1329463694,5.4353240904,-2.5802781878
C,-1.6959528307,4.8192215703,0.7444470622
H,-2.3518132671,6.0346776933,2.3855147368
O,-0.9816801308,3.5055021855,-0.9493391551
O,-1.0855546909,3.7846450431,1.3692636171
C,1.5152702276,1.644532402,0.1360097103
C,-0.5796444332,0.4609929769,0.1269327668
C,0.7742455577,0.4411405984,-0.0646830699
H,1.3283764296,-0.4592035412,-0.2873057625
B,-0.5990744272,2.8902130654,0.3283907654
O,-1.2292847588,1.557812794,0.4707113573
O,0.874575967,2.7547556978,0.4093772734
C,-2.0319543135,-2.8145873843,-0.6309174222
C,4.9473689193,0.9074696341,-0.1582160853
N,3.6549042589,0.5619448403,-0.1184497152
C,3.7786557686,2.8442541834,0.2023408186
C,-1.4332766223,-0.7058573145,0.0035088674
N,-1.0335848183,-1.8679350558,-0.5835760569
C,2.918198781,1.6890506155,0.0793983242
H,-3.2640913618,7.4417312679,-1.5921958189
C,5.9643747902,-0.0879273797,-0.3606345968
C,5.6667955692,-1.4555867993,-0.1100069873
C,7.2671291696,0.2430633111,-0.8132242622
C,6.5923389665,-2.4517126958,-0.2432435771
H,4.6606451431,-1.6662178868,0.2246681729
C,8.2474094004,-0.6941172162,-1.0036212199
H,7.4638543094,1.2758497025,-1.0574816669
C,7.9458860586,-2.103818507,-0.7066530502
O,8.8201674799,-2.9865961061,-0.8520726795
C,6.2665682513,-3.9127651437,0.0852613074
C,7.196717763,-4.4247075287,1.2036208351
C,4.8212098707,-4.0757098414,0.5751660114
C,6.4382980369,-4.7883671698,-1.172248192
H,8.2409909486,-4.3834849645,0.8949572382
H,7.0742932526,-3.8241904386,2.111390637
H,6.9423393584,-5.4620697566,1.4498622901
H,4.0954286157,-3.7592580457,-0.1793198131
H,4.6332743264,-5.1318406854,0.7923507213
H,4.6328889895,-3.5084432814,1.4918141689
H,6.188439691,-5.8292608482,-0.936377465
H,5.7666898457,-4.4534873818,-1.9699001447
H,7.4639098958,-4.7486530408,-1.5389704638
C,9.6391849623,-0.31913758,-1.5247826135
C,10.7120246657,-0.6999075113,-0.4842139204
C,9.9222849343,-1.0512795899,-2.8520673735
C,9.766291024,1.1879698527,-1.7904075687
H,10.5389736428,-0.1712507741,0.4593379198
H,10.7044775399,-1.7721923911,-0.2905024275
H,11.7038239945,-0.4142096874,-0.8534441165
H,9.1820834072,-0.7750579482,-3.6105706719
H,10.9119355083,-0.7672229236,-3.2281502574

H,9.8962076399,-2.132085862,-2.7152749689
H,10.7738908198,1.4015566859,-2.1604220594
H,9.0562766499,1.5352238415,-2.5473906444
H,9.6180016477,1.7796888975,-0.8814051959
C,-1.8580046803,-4.0725858344,-1.2689516342
C,-0.5600651771,-4.5791373132,-1.5649483693
C,-2.9819517314,-4.8480385097,-1.6695980719
C,-0.3517100856,-5.7730584212,-2.1952165338
H,0.2991372975,-4.0088865332,-1.2336359248
C,-2.868181039,-6.0487159569,-2.3098565683
H,-3.9618037638,-4.4296618626,-1.5000064506
C,-1.5228897173,-6.5755192387,-2.5960996646
O,-1.3770468895,-7.6735736416,-3.1683054573
C,1.0550101517,-6.3079215516,-2.4827125843
C,1.2445752493,-6.4931572753,-4.0021738027
C,2.1458041766,-5.3472157611,-1.9917757335
C,1.2641551926,-7.6562944524,-1.7636873393
H,0.5299770065,-7.2110355665,-4.4036998889
H,1.1193900128,-5.5399729464,-4.5267117862
H,2.2585431002,-6.8552138878,-4.2037386311
H,2.1036938003,-5.1920137049,-0.909506862
H,3.127868303,-5.7712257862,-2.2183177892
H,2.0924640808,-4.3718161717,-2.4863643528
H,2.2779614455,-8.022484094,-1.9585737899
H,1.153560839,-7.5379989627,-0.6805325867
H,0.5498200192,-8.4025746472,-2.1101039061
C,-4.096337003,-6.8498235883,-2.7557397491
C,-4.0802985349,-7.0353580286,-4.2863410473
C,-4.1019828368,-8.2289010727,-2.0652284594
C,-5.408207688,-6.1418427096,-2.3874617886
H,-4.097227283,-6.0650022127,-4.7937855175
H,-3.1934309777,-7.5818178465,-4.6059854344
H,-4.9690323192,-7.5943687891,-4.5999834413
H,-4.1356650079,-8.1159954742,-0.9763106536
H,-4.9904066749,-8.7919079282,-2.3726891364
H,-3.2146120607,-8.8031224365,-2.3298111901
H,-6.2503480466,-6.7539078571,-2.7239028192
H,-5.513359058,-6.0069455517,-1.3059674198
H,-5.5001785693,-5.1634813844,-2.8695316892
C,-2.7725028387,-0.8956319285,0.4124154364
H,-0.1516401471,-1.9634136551,-1.0643390536
C,5.0947579848,2.3488604981,0.0487644277
C,3.5400695035,4.2019862217,0.4419015025
C,6.182083264,3.2222184991,0.1789599559
H,2.5269867205,4.5662339061,0.5573577025
C,5.9392423543,4.5669285713,0.4173929864
H,7.2050959068,2.8708553693,0.119117341
C,4.6284063753,5.0542245161,0.5393170502
H,6.7766603578,5.2509488697,0.5188440433
H,4.4662589822,6.1117111108,0.7256899653
C,-3.150206833,-2.2191579061,0.03624037
C,-4.4219214718,-2.7046835276,0.4094289942
C,-3.6696727441,-0.0507422724,1.1023229584
C,-5.2811517178,-1.8680242153,1.08487205
H,-4.7222199715,-3.722884411,0.1978853169
C,-4.9121278756,-0.5428037884,1.4203198434
H,-3.3664103349,0.9547635681,1.3639141578
H,-6.2608869589,-2.2344821244,1.375282767
H,-5.6204874824,0.0877430613,1.9479244312

$1c_{OH}^{-}$-1 (closed-shell singlet state)
–2636.8663152 hartree
C,-2.644299331,7.1914845122,-2.2407891119
C,-2.7758789368,7.5727873386,-0.9110447206
C,-2.060141867,5.9602306059,-2.5857321311
C,-2.3287169651,6.7380265624,0.1280001684
H,-3.2313696466,8.5279541814,-0.6666789905
C,-1.6231103424,5.1457718574,-1.5614982979
H,-1.9538158835,5.6500114748,-3.6199863253
C,-1.756233475,5.5320213348,-0.2173249627
H,-2.4268445542,7.0224126926,1.1703571464
O,-1.0377180953,3.9330529725,-1.642341514
O,-1.259126362,4.576331446,0.5973387037
C,1.2176622802,2.1501960736,-0.3065583648
C,-0.9382669051,1.059749624,-0.154319302
C,0.4779449494,1.0090928891,-0.4054410021
H,0.9621295258,0.0667959786,-0.5827518191
B,-0.7682954277,3.5037367711,-0.2554861507
O,-1.4712195296,2.2418413146,0.0416561617
O,0.6945908097,3.3280749139,-0.0622189493
C,-3.6573620044,-1.1297454068,0.1082408363
C,4.7729401839,2.6122794565,0.0440072669
N,3.4319094524,2.9336744355,0.2724817765
C,3.4942438845,1.1636759391,-1.1953059703
C,-1.747626663,-0.0748189119,-0.0850671267
N,-3.111830684,0.0613326539,0.0911425439
C,2.6858157691,2.1058084581,-0.3922038659
H,-2.9977952619,7.8509961431,-3.0280172626
C,5.7562825393,3.3151666015,0.6963498728
C,5.3826816284,4.4309240228,1.5414087173
C,7.1596759137,2.9918817627,0.5930555059
C,6.2840217402,5.1881221419,2.2035329397
H,4.3224265653,4.6308309392,1.6018608986
C,8.1250350954,3.6918974547,1.2268738043
H,7.432294537,2.1372088795,-0.0033575368
C,7.7277068835,4.8591714521,2.0691655017
O,8.5885998722,5.5296432935,2.6436252944
C,5.8683733061,6.3734291513,3.0814440098
C,6.4861923136,7.6769022967,2.5364842607
C,4.3439115323,6.5507252248,3.1013018163
C,6.3337760767,6.1440078953,4.5335070497
H,7.5754224812,7.6370318552,2.5475548251
H,6.1502593415,7.8635162917,1.511135136
H,6.1618762844,8.5223374949,3.1531174171
H,3.830840724,5.6729413876,3.5048597537
H,4.0924781231,7.4026306312,3.7400533582
H,3.9372789794,6.7536528763,2.1064357032
H,6.0087362095,6.9821756177,5.1595771364
H,5.8887377893,5.2303852962,4.9414889846
H,7.4190459297,6.064501391,4.5967606406
C,9.6091705787,3.3280437889,1.1163959312
C,10.3960111025,4.499737284,0.4947943767
C,10.1794522219,2.9985381685,2.5106686034
C,9.8274069927,2.0979548288,0.2236501625
H,10.0147952174,4.7348168945,-0.5045608105
H,10.3271286133,5.3935637669,1.1138760856
H,11.4514830632,4.2228286316,0.3956366357
H,9.6430551552,2.1555939546,2.9590313999
H,11.2341085982,2.7158841747,2.4198288922
H,10.1058313438,3.8551861049,3.1798632762

H,10.8975934642,1.8758861568,0.17708309
H,9.3243339926,1.2086818153,0.6169629524
H,9.4836031909,2.2682801375,-0.8018967787
C,-5.1131939251,-1.2850084154,0.2729727852
C,-5.9622796343,-0.2599508173,-0.1491663067
C,-5.6819523205,-2.4045176039,0.872151774
C,-7.3434125821,-0.3259136069,-0.0081514244
H,-5.4910741788,0.6080329004,-0.5883122187
C,-7.0618589094,-2.5410994308,1.0477523538
H,-5.0216892729,-3.1738635787,1.2434902096
C,-7.8745595224,-1.4874749413,0.5898554835
O,-9.247745609,-1.5357200487,0.7088037427
C,-8.2438209899,0.8285329446,-0.4876182585
C,-9.0354999765,1.4173349259,0.6973020407
C,-7.4203597034,1.9746890534,-1.0948462658
C,-9.2144216013,0.3366871462,-1.5799285818
H,-9.6876099286,0.6755508985,1.1583330702
H,-8.3505198429,1.8001614489,1.4604014793
H,-9.6531216484,2.2536598974,0.3507707388
H,-6.8524733583,1.6542610766,-1.9728401078
H,-8.1010538173,2.7701557173,-1.4137514917
H,-6.7188399806,2.4074039827,-0.376792732
H,-9.8302027939,1.1723779786,-1.9314308905
H,-8.6572091795,-0.0533604633,-2.4379914966
H,-9.87582989,-0.4478336537,-1.2124540489
C,-7.6422637161,-3.7989497706,1.7304283384
C,-8.3920696188,-3.4288672198,3.0306136198
C,-8.5617089015,-4.5806234789,0.7640652891
C,-6.539453039,-4.7855846715,2.1488795712
H,-7.7051232028,-2.9475761559,3.7321505328
H,-9.2287250399,-2.7386793986,2.8929990719
H,-8.7886595466,-4.3322456954,3.50642018
H,-7.9935846685,-4.9003515167,-0.1140098526
H,-8.9555386975,-5.4743513642,1.2599143068
H,-9.4179081957,-4.0131205095,0.3890718754
H,-7.0001489333,-5.6560120733,2.6264565264
H,-5.96475065,-5.1444305452,1.2908827926
H,-5.8445383564,-4.3422455714,2.8666009306
C,-1.412279471,-1.4868832248,-0.1822333168
C,4.8362737334,1.4859809399,-0.917568769
C,3.1591068829,0.192581737,-2.128687313
C,5.8553420277,0.8018727094,-1.5747924013
H,2.1248909493,-0.0139922491,-2.3766429077
C,5.519254252,-0.195140109,-2.4902498262
H,6.9013688481,1.0284050976,-1.4169236627
C,4.1878977399,-0.4957699983,-2.7688779714
H,6.3106986297,-0.7312039416,-3.0044969119
H,3.9481134391,-1.2609892616,-3.4999918124
C,-2.6547639678,-2.1685024953,-0.0731402193
C,-2.7236868304,-3.5604225659,-0.1911670274
C,-0.2398972457,-2.2420857913,-0.3437450546
C,-1.5557842083,-4.2847203747,-0.3719494458
H,-3.6783403929,-4.0741217794,-0.1584610918
C,-0.320345047,-3.6236312942,-0.4332446018
H,0.7386410472,-1.7809188602,-0.3922847799
H,-1.595432695,-5.3659317714,-0.4652308146
H,0.5912204894,-4.2012743278,-0.5576113763
H,-9.5127669105,-2.3575870448,1.1263270543

1c$_{OH}$$^{-}$-2 (closed-shell singlet state)

–2636.8774604 hartree
C,-2.0428185053,7.1081306117,-2.0509146373
C,-2.3301971314,7.4231167867,-0.7283295891
C,-1.4696619359,5.8706107638,-2.3919717533
C,-2.0558046964,6.5125209279,0.3072936495
H,-2.7746497257,8.3842991352,-0.4872362999
C,-1.2028692748,4.9808045694,-1.3713902067
H,-1.2429751215,5.6120259567,-3.4210134032
C,-1.4934986895,5.3002367359,-0.0339772824
H,-2.2778524976,6.7440857291,1.343791028
O,-0.661130104,3.7492637358,-1.4501891194
O,-1.1426188064,4.280669773,0.7786975183
C,1.3359164569,1.776573298,0.1208663726
C,-0.857013373,0.7875464484,-0.1065082479
C,0.5750967621,0.6745206862,-0.116431769
H,1.0694493063,-0.2690128786,-0.2620711817
B,-0.5908092809,3.2261952429,-0.0686413089
O,-1.3682484667,1.9890582393,0.0633588591
O,0.8265795455,2.9846027851,0.3028808507
C,-3.6813671611,-1.2644053355,-0.3249555462
C,4.8014752319,0.8103416823,0.2343065855
N,3.43773184,0.5642015525,0.0963295732
C,3.7095848476,2.820696993,0.4557483793
C,-1.7157127207,-0.3028372663,-0.2236725672
N,-3.0839929238,-0.105775567,-0.1986077717
C,2.7936230731,1.6918868951,0.2169844604
H,-2.2641294312,7.8253784848,-2.8359004258
C,5.6947343113,-0.2340595588,0.1465952598
C,5.1980568803,-1.5712654846,-0.0941739617
C,7.1205038762,-0.0690221307,0.2845383408
C,6.0011387831,-2.6535035256,-0.1940946287
H,4.1260250146,-1.6570105192,-0.1919543414
C,7.9953256295,-1.0961024571,0.1995954555
H,7.4926933157,0.9250870438,0.4651149998
C,7.4681045754,-2.4681006286,-0.0490175024
O,8.2410550812,-3.427117442,-0.131131038
C,5.4501181303,-4.060424889,-0.4509281755
C,5.8225308351,-4.9957866815,0.7169384132
C,3.9198220774,-4.0532295466,-0.5729545315
C,6.0225003156,-4.6200963241,-1.7687558066
H,6.9034039131,-5.0868962069,0.8233373865
H,5.4059485611,-4.6224387649,1.6583420003
H,5.4042008891,-5.9924254005,0.5379002814
H,3.575788528,-3.4337464259,-1.4067165382
H,3.5720608047,-5.0743939754,-0.7557552964
H,3.434898945,-3.6988598252,0.3416735936
H,5.6049056717,-5.6151488081,-1.9580588808
H,5.7496459667,-3.9769157927,-2.6120007937
H,7.1085558639,-4.7016397469,-1.7269425844
C,9.5073326266,-0.8987594537,0.350624562
C,10.0304721871,-1.7153233453,1.5496013865
C,10.2304849003,-1.3424126925,-0.9371041536
C,9.8666107239,0.5731143781,0.6000229839
H,9.5409070223,-1.3982925014,2.4765102025
H,9.8536270327,-2.7810547916,1.4079252867
H,11.1077211659,-1.5517174421,1.6652447298
H,9.8841708486,-0.7579640837,-1.7960676365
H,11.3083471294,-1.1768520534,-0.8302794691
H,10.0592528528,-2.3988392394,-1.1413998781
H,10.9522266685,0.6636410788,0.7005743528

H,9.5592203014,1.2179379204,-0.2297293665
H,9.4187248004,0.955490118,1.5230337141
C,-5.1522941965,-1.3419357212,-0.3387440517
C,-5.8802896951,-0.2213418086,-0.7366280677
C,-5.8565616822,-2.4746095073,0.06558111
C,-7.2715213651,-0.1999227156,-0.7550345806
H,-5.305588001,0.6479583384,-1.0222395732
C,-7.2505864378,-2.5275667068,0.0711089071
H,-5.2961840838,-3.3269838898,0.4194458084
C,-7.9385935167,-1.3755504753,-0.3503680693
O,-9.3152605569,-1.4513235166,-0.3455576473
C,-8.0244698713,1.0717807616,-1.2027742779
C,-8.8954473797,1.6309496657,-0.0545394235
C,-7.0539368982,2.2014124444,-1.5850685056
C,-8.8785298309,0.7972594198,-2.4614206561
H,-9.6647824775,0.9456745281,0.3141424537
H,-8.2647539969,1.8831629207,0.8021905954
H,-9.4044845034,2.5433989482,-0.382229704
H,-6.4092320456,1.9210279377,-2.4216190544
H,-7.6304216761,3.0803650756,-1.8896852779
H,-6.4147743653,2.4957666327,-0.7494843908
H,-9.3887722525,1.7144903356,-2.7738366248
H,-8.2359807995,0.4697941143,-3.2834781021
H,-9.6436695509,0.0256478185,-2.33578751
C,-7.9959121864,-3.7949885739,0.5317927198
C,-8.8799216228,-3.4853332751,1.7563674381
C,-8.8601372927,-4.3565692076,-0.6150396398
C,-7.0242474825,-4.9099396247,0.9492696248
H,-8.2674974043,-3.1195988005,2.5868393196
H,-9.6343685014,-2.7329298841,1.5283274222
H,-9.3893702298,-4.3972975502,2.0885006371
H,-8.2332505539,-4.6140677688,-1.4750233792
H,-9.37070102,-5.2685392697,-0.2846103133
H,-9.6126631923,-3.6392280252,-0.9413073269
H,-7.6016939344,-5.7845769456,1.2647395393
H,-6.3773076625,-5.2227456746,0.1241480548
H,-6.390409825,-4.6122604674,1.7896256919
C,-1.4364309902,-1.7242369136,-0.3840763821
C,5.0021763922,2.2631248674,0.4671891154
C,3.4899438151,4.1785156267,0.6425734934
C,6.1002543232,3.0943163051,0.6698440004
H,2.4825560439,4.5730192718,0.6283079683
C,5.884851534,4.4601534047,0.8582503966
H,7.1169411323,2.7252938848,0.6864624289
C,4.5996071986,4.9974380972,0.8455526648
H,6.738449641,5.1120972253,1.0163830403
H,4.459229252,6.0633420981,0.9935182718
C,-2.7117352188,-2.3436260109,-0.4628459853
C,-2.8295880791,-3.7185492774,-0.6874360999
C,-0.28723917,-2.5213911393,-0.4862367954
C,-1.6807595999,-4.4874513752,-0.794635671
H,-3.8024732262,-4.1849090597,-0.7954600837
C,-0.4185528101,-3.8880799803,-0.6836874971
H,0.7091312103,-2.1037449404,-0.4198239471
H,-1.757657938,-5.5564528528,-0.9691769707
H,0.475288328,-4.5000904057,-0.7629104372
H,-9.6879473941,-0.6088765957,-0.6148169902

$1c_{OH}^{-}$-3 (closed-shell singlet state)

–2636.8802059 hartree

C,-2.8988447493,7.0373320408,-1.2616839586
C,-2.9550052998,7.2113898308,0.1158137214
C,-2.2883761318,5.9024489658,-1.8232861806
C,-2.4028372305,6.2568911232,0.9879283283
H,-3.4312486725,8.0958837722,0.5285422852
C,-1.7477345133,4.9695740263,-0.9622500121
H,-2.2389433225,5.7547148066,-2.8970170576
C,-1.8053195039,5.1460538162,0.4304446641
H,-2.4393070639,6.3802495639,2.0652331441
O,-1.1192065646,3.814410493,-1.2606491639
O,-1.2150176609,4.1068979821,1.0598000514
C,1.2926487139,1.9195086454,-0.2825675467
C,-0.8166201312,0.7532114596,-0.1920090483
C,0.5907659712,0.7700973187,-0.4791229801
H,1.0597355581,-0.1620149263,-0.7472632636
B,-0.7376082287,3.195630697,0.0255713719
O,-1.399222644,1.8769227767,0.1705282001
O,0.7290929087,3.0459274493,0.1057274437
C,-1.9052753279,-2.549612415,-0.5879290256
C,4.8243051213,2.560366854,-0.0206536428
N,3.4772601017,2.7967379551,0.2598672877
C,3.5891347429,1.085010077,-1.2742646197
C,-1.558608483,-0.4163727175,-0.2501570959
N,-0.9730691784,-1.6242151533,-0.600075085
C,2.7547929148,1.9524936631,-0.4127362307
H,-3.3320203608,7.786880065,-1.9175304849
C,5.7882188348,3.2947370384,0.6277329032
C,5.379986366,4.3533115863,1.5277677339
C,7.2032711341,3.0598263972,0.467689219
C,6.2580681628,5.1356395284,2.1926219531
H,4.3125491888,4.488349357,1.6287370863
C,8.147502327,3.790698576,1.0993408852
H,7.5053515063,2.247724829,-0.1723380614
C,7.7125153859,4.8971144493,2.0017939655
O,8.5524170133,5.5929379741,2.5784710084
C,5.8039520004,6.2597263665,3.130164543
C,6.3293236572,7.6171123994,2.6211006196
C,4.2733105054,6.3478102725,3.2022489378
C,6.3274273066,6.0012912404,4.5573385747
H,7.4188763745,7.6393265759,2.5976590116
H,5.9503833137,7.824138542,1.6148206582
H,5.9781185892,8.4176945639,3.2814318432
H,3.8236880953,5.426859785,3.5847042466
H,3.9945034241,7.1584714385,3.8820949685
H,3.8243906099,6.5650578806,2.2288290841
H,5.9759117434,6.7944175545,5.2264209822
H,5.9477184587,5.0483365631,4.9406750938
H,7.4169298209,5.9817242557,4.5848270973
C,9.6457945873,3.5210604422,0.9256523845
C,10.3407719701,4.7625289131,0.3305664887
C,10.2824989992,3.169081211,2.2852313167
C,9.9048888762,2.3443558033,-0.0261710256
H,9.9123021369,5.0151182272,-0.6451136977
H,10.2402306911,5.6242194632,0.9896236303
H,11.4068003976,4.5542706718,0.1861049308
H,9.8126495312,2.2772742501,2.7133240119
H,11.3483765985,2.9549624129,2.1487440274
H,10.1809790804,3.9912342722,2.9929593759
H,10.9840532624,2.1893762816,-0.1170209319
H,9.469015981,1.4108884493,0.3443187412

H,9.5157472887,2.5358502122,-1.0315705834
C,-1.5841655918,-3.9488055201,-0.917017707
C,-0.3044087646,-4.4384760769,-0.6633001896
C,-2.5114531558,-4.8078741349,-1.5050233246
C,0.0722671258,-5.7450208553,-0.965091371
H,0.3972450035,-3.748074042,-0.2178927957
C,-2.2071833525,-6.128267748,-1.834569234
H,-3.4907200818,-4.4162514824,-1.738097804
C,-0.9050673619,-6.5777750171,-1.5488064135
O,-0.6281052779,-7.8889852271,-1.8739611023
C,1.5038666066,-6.2353925538,-0.6575043707
C,2.2363230035,-6.6536295585,-1.9529859342
C,2.3625193571,-5.1263993264,-0.0274911492
C,1.4862355278,-7.392948843,0.3666692425
H,1.7551657202,-7.4624298058,-2.5103760639
H,2.3021744764,-5.8015851342,-2.6351821068
H,3.2544515802,-6.9816368781,-1.7183254503
H,1.9497080474,-4.7803669458,0.9233442637
H,3.3656556046,-5.5179532517,0.1678627853
H,2.4649772432,-4.2626156553,-0.6888764548
H,2.509658657,-7.7159203272,0.5848414694
H,1.0294724731,-7.0563933861,1.3014710993
H,0.927490687,-8.2773138353,0.0474536899
C,-3.2565971331,-7.0450529272,-2.4920911067
C,-2.7689107096,-7.5152727146,-3.8770476474
C,-3.5451116034,-8.2630284947,-1.5916872212
C,-4.5935946946,-6.3192554328,-2.7088836921
H,-2.5962766955,-6.6560681788,-4.5331954018
H,-1.8432974313,-8.0859179434,-3.8065767026
H,-3.5313441206,-8.1487127252,-4.3447036044
H,-3.9266186515,-7.9370648193,-0.6185734924
H,-4.3078855554,-8.8975542834,-2.0573384734
H,-2.6511691651,-8.8638362052,-1.4266890614
H,-5.3013970201,-7.0112181411,-3.1757813422
H,-5.036623888,-5.9808002038,-1.76755601
H,-4.4917597996,-5.454634357,-3.3710735105
C,-2.9629375487,-0.6126234133,0.0104180242
C,4.9191843141,1.4743184725,-1.0251955569
C,3.2827134696,0.1252299222,-2.2293335927
C,5.9554109061,0.8735143762,-1.7348934319
H,2.2566212115,-0.1406108269,-2.451152479
C,5.6494839576,-0.1102411943,-2.6746445357
H,6.9909575844,1.1553296504,-1.598727881
C,4.3295237642,-0.4792453587,-2.9229372397
H,6.4540340379,-0.5815111839,-3.2304461962
H,4.1109058592,-1.233778839,-3.6716691121
C,-3.1963838901,-1.9928862101,-0.2001952838
C,-4.4680435952,-2.5323192693,0.0252830906
C,-4.001127894,0.2361175657,0.4122743524
C,-5.4912750496,-1.6855057332,0.4259437738
H,-4.6588327909,-3.5939762115,-0.090987872
C,-5.2592283925,-0.3114950317,0.6097317535
H,-3.8120211619,1.2912155545,0.5693009738
H,-6.4834872778,-2.0894012516,0.6055395071
H,-6.0785673583,0.3297091293,0.9217638528
H,0.2818002404,-8.0960249691,-1.650717044

1c$_{OH}^{-}$-4 (closed-shell singlet state)
–2636.8880433 hartree
C,-2.4315798009,6.9818954735,-0.8228408207
C,-2.5858075487,7.069787751,0.555395126
C,-1.8467107404,5.8492454673,-1.4151218449
C,-2.1607302803,6.0272834415,1.3976178877
H,-3.0418721725,7.953531422,0.9917893713
C,-1.4312175628,4.8291888407,-0.5834938727
H,-1.7227255456,5.768196443,-2.4898863132
C,-1.5873914581,4.9189529374,0.8101220455
H,-2.2770313583,6.0815431889,2.4750576939
O,-0.8500797222,3.6591227609,-0.9143591499
O,-1.1072058726,3.8067940552,1.4081098037
C,1.394661588,1.5651389457,0.1353649091
C,-0.7627220119,0.5070882875,-0.0062128495
C,0.6633685059,0.4615308635,-0.1696549157
H,1.1360941938,-0.4559001952,-0.4842700005
B,-0.6016310081,2.9323903775,0.3491520338
O,-1.3233574584,1.6475532187,0.3570512964
O,0.845454187,2.7079825648,0.5360392099
C,-2.0210794726,-2.7043278518,-0.6003339752
C,4.904030492,0.8808293808,-0.2766600062
N,3.550342747,0.5748389099,-0.3870794228
C,3.7239591499,2.6625054283,0.5683287526
C,-1.564166625,-0.6093894027,-0.1740637084
N,-1.0312098442,-1.8490974423,-0.500451225
C,2.8555602177,1.5708231888,0.0892473353
H,-2.7680614684,7.7979608907,-1.4555681808
C,5.8468456509,-0.0173124977,-0.7256079558
C,5.412123108,-1.245633721,-1.3535186295
C,7.2666748523,0.2044467491,-0.6099751034
C,6.2689811982,-2.1716268996,-1.8392929238
H,4.342120749,-1.3769415655,-1.424011363
C,8.1917323923,-0.6744416635,-1.0567562104
H,7.592618949,1.112761606,-0.1325392148
C,7.7288963548,-1.9326034268,-1.7068741886
O,8.5486617654,-2.7570959758,-2.1233868059
C,5.7852221486,-3.4557371646,-2.5217847673
C,6.2784558503,-4.6894398553,-1.7393560464
C,4.2531299643,-3.5196918521,-2.5791715358
C,6.310032661,-3.5151161267,-3.9705180156
H,7.3672223907,-4.7288899863,-1.7062578844
H,5.8951053924,-4.6738576242,-0.7136203101
H,5.9104150923,-5.6022086806,-2.2210453643
H,3.8254818041,-2.6894322258,-3.1486997517
H,3.9528183515,-4.4482843898,-3.0738952381
H,3.8029009253,-3.5154559321,-1.582266296
H,5.9372765463,-4.4217973509,-4.4598214287
H,5.9530665044,-2.654166136,-4.5454312081
H,7.3994814891,-3.5281387291,-3.9983655261
C,9.696603312,-0.4224676586,-0.9151670649
C,10.3417552744,-1.5335031223,-0.0624036084
C,10.359844751,-0.3826789356,-2.3067469914
C,9.9865121494,0.9184691635,-0.2255388346
H,9.8959358378,-1.5614230068,0.9375238014
H,10.2163811283,-2.5104448664,-0.5282898418
H,11.4133263155,-1.3353262282,0.0524543098
H,9.9266148428,0.4154857988,-2.918792974
H,11.4313268892,-0.180097362,-2.1990728613
H,10.2354410331,-1.3304455836,-2.829623012
H,11.0694230037,1.0536029237,-0.1480678167
H,9.589943085,1.7668137698,-0.7928886518
H,9.5777018523,0.9564815534,0.7894262993

C,-1.775611182,-4.119645917,-0.9247254903
C,-0.5886201016,-4.7246256259,-0.5173334822
C,-2.6894930906,-4.8829666584,-1.649657312
C,-0.2927310837,-6.0566650271,-0.7977903625
H,0.1053832695,-4.1068564658,0.0342960879
C,-2.4584419563,-6.2188151291,-1.9751870583
H,-3.5925079755,-4.4014145052,-1.9957659182
C,-1.2492860453,-6.7867509102,-1.5335162372
O,-1.0447598348,-8.1110095163,-1.8571092783
C,1.0282709451,-6.6847654322,-0.302816726
C,1.9081280875,-7.1374868981,-1.4904711516
C,1.8759739273,-5.6751012544,0.4880622769
C,0.7540925083,-7.8600821419,0.6627172285
H,1.4549732319,-7.8945059446,-2.1381421554
H,2.1428053987,-6.2815640544,-2.1286935594
H,2.850689261,-7.554025167,-1.1206060127
H,1.3537143811,-5.312823413,1.377132555
H,2.798406837,-6.1626697435,0.8180140829
H,2.1559851952,-4.8113736642,-0.1193456911
H,1.7008789291,-8.2848975448,1.01264497
H,0.1990636579,-7.5037112908,1.5350441882
H,0.16838251,-8.6755728225,0.2302426709
C,-3.4848893088,-7.0269520961,-2.7920231316
C,-2.8592841511,-7.5099931089,-4.1157375884
C,-3.991426058,-8.232953794,-1.975711163
C,-4.7155731605,-6.1825983637,-3.1578828294
H,-2.5286374271,-6.6563415389,-4.7160704296
H,-2.0030111456,-8.1612498431,-3.9427445862
H,-3.6036609021,-8.0642793112,-4.6988094082
H,-4.4703902125,-7.8946615223,-1.0510478097
H,-4.7357953007,-8.7892713398,-2.5568774914
H,-3.1808838086,-8.9125351129,-1.7139234222
H,-5.4118547064,-6.8007387309,-3.7331310201
H,-5.248225986,-5.8243974612,-2.2718803893
H,-4.4547409085,-5.3178927543,-3.774868812
C,-2.999704208,-0.7117911588,-0.0504337388
C,5.04205793,2.2241575581,0.3420315141
C,3.4457085148,3.8970125441,1.1389673867
C,6.1049836248,3.0519627962,0.6924183296
H,2.4202894374,4.2044824418,1.2955454492
C,5.8305857592,4.2933777621,1.267703716
H,7.1383554841,2.7743799146,0.5341040437
C,4.520833017,4.7119635259,1.4902738799
H,6.6566914808,4.941893186,1.5428367899
H,4.3335782725,5.6831111519,1.9371202856
C,-3.3047305927,-2.0658203423,-0.3224675014
C,-4.6238778317,-2.5217664914,-0.2367818399
C,-4.0123414791,0.1971332647,0.2753998154
C,-5.6232950359,-1.6151196426,0.0889424255
H,-4.871249443,-3.5647945631,-0.4039897651
C,-5.3184565796,-0.2663330575,0.3350731164
H,-3.7696953585,1.2323291292,0.4817874225
H,-6.6531702373,-1.9530297521,0.15980775
H,-6.1191180036,0.4233533701,0.5863434483
H,-0.1652900303,-8.3815653264,-1.5842250869

$1c_{OH^-}$-1 (open-shell singlet state)
–2636.8663152 hartree
C,-2.5736817834,7.1880480866,-2.4148008102
C,-2.729141048,7.5818558912,-1.0912928841
C,-1.9878806475,5.9515400085,-2.7374406147
C,-2.3051911027,6.7548563804,-0.0364318692
H,-3.1854622678,8.5408979578,-0.8642703781
C,-1.573695829,5.1447468237,-1.6977632566
H,-1.8630369018,5.6315800337,-3.7666452864
C,-1.7309556059,5.5436366096,-0.3599042835
H,-2.4220948353,7.0490267943,1.0012625159
O,-0.9916317725,3.9291550936,-1.7565002426
O,-1.2532569282,4.5934916477,0.4726634563
C,1.2308245684,2.1499890645,-0.3616935324
C,-0.931878021,1.0690587948,-0.240899237
C,0.4886722762,1.0107949942,-0.4643183007
H,0.9724701365,0.0651030585,-0.6236867652
B,-0.7504442585,3.5113947349,-0.3609562198
O,-1.4638568618,2.2548861664,-0.0660047565
O,0.7077980383,3.3320070015,-0.1381606416
C,-3.6641036432,-1.107723247,-0.010641036
C,4.7805287655,2.6018974948,0.0527944708
N,3.4366438779,2.9304245986,0.2525636877
C,3.5201055269,1.1468075202,-1.1975896391
C,-1.7468693072,-0.0617881028,-0.176865985
N,-3.1136526013,0.0810901531,-0.0281512504
C,2.7001620638,2.0993093327,-0.4187559156
H,-2.9094825184,7.841734683,-3.2145349511
C,5.7539644502,3.3070130068,0.7174734161
C,5.3686700963,4.4319114925,1.5449805633
C,7.1577833126,2.9774620031,0.644103943
C,6.2601560807,5.1917424822,2.2173509472
H,4.308254902,4.6363969749,1.5832278195
C,8.1135899913,3.6796062817,1.289932942
H,7.4383810082,2.1163021538,0.0608668984
C,7.7048285255,4.8560596716,2.1137775562
O,8.5572176743,5.5285448615,2.6984748539
C,5.8324705278,6.3866598709,3.0762469061
C,6.4658484439,7.682690378,2.5313974335
C,4.3086301824,6.5699584565,3.0651540235
C,6.2689853193,6.1688336778,4.5390277451
H,7.5544971057,7.6387582053,2.5637788953
H,6.1504112108,7.8611455835,1.4981162255
H,6.1331394014,8.5349982033,3.1339370322
H,3.7843962786,5.6978907005,3.466753143
H,4.0483861854,7.4286552077,3.6911555797
H,3.9219930815,6.7652844818,2.0608392973
H,5.9353438135,7.013966499,5.1510521471
H,5.8125405569,5.2607153413,4.9466962406
H,7.3525135688,6.0857581783,4.6238678073
C,9.5981162419,3.3091299488,1.2113043767
C,10.4013655508,4.4720899646,0.5943077136
C,10.1402431736,2.990273628,2.6192172517
C,9.8285680637,2.0700876035,0.334144727
H,10.0403302973,4.6994292055,-0.4142897892
H,10.3241481986,5.3718129423,1.2037910083
H,11.4574434887,4.1902994302,0.5179855527
H,9.5920215692,2.1535044797,3.064836404
H,11.1953245891,2.7028241028,2.5512371145
H,10.057177048,3.8532926683,3.2790549166
H,10.8985641731,1.8435576559,0.3101742123
H,9.3145318716,1.1863663047,0.7257914776
H,9.5051756627,2.232296843,-0.6993269467
C,-5.1234235817,-1.2559825348,0.1275410053

C,-5.9602174032,-0.2315832238,-0.3199961106
C,-5.707976122,-2.3678642364,0.7257108785
C,-7.3440424396,-0.2910411389,-0.2049120435
H,-5.4772609701,0.6305966241,-0.757806313
C,-7.0915270563,-2.4976231422,0.8760453998
H,-5.058003062,-3.1363346478,1.1165015213
C,-7.8911340135,-1.4451141554,0.3932222875
O,-9.2665305817,-1.4870846522,0.4861939296
C,-8.2305272342,0.8624017214,-0.7119387923
C,-9.0424313322,1.4649238451,0.4522189397
C,-7.391044756,1.9998657381,-1.3135427783
C,-9.1819627018,0.3643287639,-1.818202297
H,-9.7061655907,0.7298210874,0.9073251843
H,-8.3706897097,1.8520896089,1.2248382239
H,-9.6500027268,2.300383569,0.0863733078
H,-6.8076834244,1.6693182974,-2.1775277273
H,-8.0623442589,2.7949634713,-1.6526406032
H,-6.7017288079,2.4364459501,-0.5860709894
H,-9.7875990008,1.1991118872,-2.1889604687
H,-8.6099625638,-0.0355870071,-2.6618714591
H,-9.8533775771,-0.4143273177,-1.4564436857
C,-7.6898662136,-3.7470736378,1.5586613399
C,-8.4629090056,-3.3624844946,2.8409032724
C,-8.5937782405,-4.5338854149,0.5818677909
C,-6.5991912622,-4.7341107026,2.0069116492
H,-7.7875706239,-2.8775980201,3.5511782945
H,-9.2939814919,-2.6703220262,2.6811724187
H,-8.8721685002,-4.2600321143,3.3170173804
H,-8.0102452158,-4.8636785284,-0.2822680775
H,-9.0006027199,-5.4215927426,1.078038472
H,-9.4403612084,-3.9665007956,0.1854711561
H,-7.0723972635,-5.5984711145,2.4832737478
H,-6.0096063346,-5.1027925919,1.1633045168
H,-5.916386366,-4.2870159788,2.7338670665
C,-1.4152761511,-1.4759354598,-0.2546613769
C,4.8578278068,1.4666035306,-0.8971046426
C,3.199102153,0.1684809984,-2.1283837231
C,5.8866075595,0.772700395,-1.5284169963
H,2.1690265658,-0.0364597805,-2.394277651
C,5.5642058189,-0.2313843533,-2.4410628157
H,6.9302954189,0.9967741688,-1.3525798351
C,4.2372595054,-0.5295509938,-2.7424428125
H,6.3632406807,-0.7750993874,-2.9351435484
H,4.0085152815,-1.300519509,-3.4710501495
C,-2.6622963258,-2.1518540067,-0.1632266194
C,-2.7344101779,-3.5445331827,-0.2698422155
C,-0.2429914913,-2.2369889654,-0.386736581
C,-1.56611273,-4.2748429044,-0.421545344
H,-3.6915221262,-4.0543311989,-0.2507820244
C,-0.3271374457,-3.6189880879,-0.4651452789
H,0.7381048486,-1.7799715849,-0.4206851214
H,-1.6082182447,-5.3567100743,-0.5056960097
H,0.5843693958,-4.201165317,-0.5667085448
H,-9.5427678772,-2.304030856,0.9060824729

1$_{\mathrm{COH}^-}$-2 (open-shell singlet state)
–2636.8759727 hartree
C,-2.2190803839,7.3621033342,-1.2501502045
C,-2.3729910765,7.531210112,0.1205296847
C,-1.6954908659,6.1690101536,-1.7778102201
C,-2.0092673237,6.5132419913,1.0195127344
H,-2.7822450103,8.4601603078,0.5066157769
C,-1.3404867259,5.1734516428,-0.8906044735
H,-1.5736693823,6.0236277223,-2.8460628645
C,-1.4960885411,5.34533356,0.495341414
H,-2.127836689,6.6301863532,2.0916483831
O,-0.8224077556,3.9572545576,-1.1549868708
O,-1.0788446532,4.2428933706,1.1545010215
C,1.3173499573,1.8486741784,-0.0153508727
C,-0.8882168865,0.8676580618,-0.1253017902
C,0.5353647543,0.7716378465,-0.2947150815
H,1.0117635418,-0.1452340759,-0.5912605972
B,-0.6176166687,3.2906913359,0.1482103153
O,-1.378910292,2.038075511,0.2228793196
O,0.8303952345,3.0234151175,0.3497074667
C,-3.7108782433,-1.1958707332,-0.2416683242
C,4.7760861057,0.9112254309,-0.3572585512
N,3.4051254714,0.6671400736,-0.3822379907
C,3.7136000249,2.8749894451,0.1880775274
C,-1.7526208748,-0.216100726,-0.2654892406
N,-3.1108744623,-0.0450353474,-0.067744006
C,2.7774301886,1.7687550366,-0.0766526154
H,-2.5087039247,8.1603080681,-1.9273302726
C,5.6551167495,-0.1086611445,-0.6460794988
C,5.135687553,-1.4175949868,-0.9772018638
C,7.0876282251,0.0552783826,-0.6360747896
C,5.9237989624,-2.474867656,-1.272554355
H,4.0591968488,-1.5032581435,-0.9750984002
C,7.9486326853,-0.9480371065,-0.9179972041
H,7.4768539142,1.0283730478,-0.3891240552
C,7.3980246981,-2.2912302852,-1.2566550771
O,8.1580233143,-3.2284938037,-1.5173360504
C,5.3485876622,-3.8519442896,-1.6216397078
C,5.8357291531,-4.9023653607,-0.603404775
C,3.8137857726,-3.8469332431,-1.5885187426
C,5.7850803159,-4.2627585396,-3.0422816957
H,6.9217710979,-4.9932949058,-0.6158507007
H,5.5160054761,-4.6353923731,0.4093540651
H,5.4009377291,-5.8785253668,-0.8451636401
H,3.3883148475,-3.145719032,-2.3127860683
H,3.4485620748,-4.8462810831,-1.8436379793
H,3.4234621872,-3.5965705148,-0.5974465231
H,5.3496296965,-5.2361005294,-3.2940091627
H,5.4294874615,-3.5362791916,-3.7805007195
H,6.8697590773,-4.337637205,-3.1182547403
C,9.4682914203,-0.7523954701,-0.9005139807
C,10.1101110785,-1.6857294052,0.1458816977
C,10.0565276101,-1.0500776203,-2.2945658593
C,9.8519698576,0.6884490268,-0.533854847
H,9.7173830162,-1.4735356298,1.1459238419
H,9.9192445286,-2.7322645706,-0.0896277792
H,11.1936187422,-1.5241683403,0.168803827
H,9.6250910985,-0.3816373613,-3.0471459728
H,11.139776405,-0.8858189441,-2.2803361336
H,9.8647082574,-2.0806724414,-2.5915167091
H,10.9422353847,0.7789378057,-0.5347453424
H,9.4620598101,1.4142250199,-1.2549545547
H,9.5006206919,0.9664673202,0.4651546673
C,-5.1716965531,-1.3093163792,-0.0918504937
C,-5.9725988822,-0.1907500163,-0.3313112573

C,-5.7905218635,-2.4855661209,0.3197357468
C,-7.355017214,-0.2164411046,-0.1907353132
H,-5.4621327276,0.7152412535,-0.6261363668
C,-7.1747142074,-2.586120183,0.4841103175
H,-5.1658337733,-3.3349112237,0.5521081479
C,-7.9382934144,-1.4354599804,0.2129940559
O,-9.3116371887,-1.4399697395,0.3356768189
C,-8.2021557124,1.0405199925,-0.4659561718
C,-8.9640291281,1.4616298519,0.8066867858
C,-7.3276575889,2.2324050713,-0.8843802532
C,-9.1960046541,0.7792884381,-1.6153932446
H,-9.6484511138,0.6841500295,1.1459907804
H,-8.2609419733,1.6829874913,1.6158825172
H,-9.5435308954,2.3702212741,0.6076107635
H,-6.7762929431,2.0350781815,-1.8079961661
H,-7.9714889512,3.0993427643,-1.0625151308
H,-6.6060134888,2.5091350779,-0.111277905
H,-9.7735186751,1.6882081854,-1.8189968497
H,-8.6586530255,0.5113496455,-2.530977137
H,-9.8920315249,-0.0245524688,-1.3759419717
C,-7.8114679319,-3.9112351773,0.9572246091
C,-8.5410952545,-3.7250745209,2.3071814596
C,-8.7678948227,-4.4819379235,-0.1151496919
C,-6.7544757367,-5.0018461056,1.1981210385
H,-7.8310713847,-3.3984349653,3.0719647561
H,-9.3437958578,-2.9830515554,2.2915563554
H,-8.979609626,-4.6748036482,2.6319356431
H,-8.2178659756,-4.6753620019,-1.0403469142
H,-9.1983765296,-5.4279549152,0.2303335466
H,-9.600723076,-3.825120223,-0.3815363809
H,-7.2537057355,-5.9166889671,1.5323666509
H,-6.1992482606,-5.2417078876,0.2874275525
H,-6.037912633,-4.7133927162,1.9713529301
C,-1.4852521955,-1.6087188173,-0.6016975933
C,5.0003113832,2.3328286006,0.008655203
C,3.5147583336,4.202726158,0.5406529568
C,6.1137650898,3.1489753077,0.1869181468
H,2.511356249,4.5860080209,0.6708749619
C,5.919351314,4.4846198896,0.5415247523
H,7.1266700368,2.7909117352,0.0607071942
C,4.6398009984,5.0068033834,0.7167520595
H,6.784946464,5.1248386884,0.6812776544
H,4.5157413601,6.0493310919,0.9914899466
C,-2.7577339661,-2.2384737649,-0.5980049685
C,-2.8950419771,-3.5847501216,-0.9491824851
C,-0.3522878708,-2.3729532387,-0.9176562826
C,-1.7633104693,-4.3201882951,-1.2663492351
H,-3.8731063896,-4.0512393721,-0.9942404852
C,-0.499453894,-3.7142440736,-1.2390256515
H,0.6433099372,-1.9484662041,-0.9252645163
H,-1.8548258948,-5.3665520812,-1.5420503554
H,0.3820332878,-4.2997461387,-1.4838960551
H,-9.614734376,-2.3094940892,0.6043035653

1c$_{OH^-}$-3 (open-shell singlet state)

–2636.8802059 hartree

C,-2.8988447834,7.0373320369,-1.2616838615
C,-2.9550053246,7.2113898124,0.1158138207
C,-2.2883761641,5.902448971,-1.8232860999
C,-2.4028372439,6.2568910984,0.9879284136
H,-3.4312486989,8.095883747,0.5285423971
C,-1.7477345344,4.9695740253,-0.962249945
H,-2.2389433621,5.7547148232,-2.8970169788
C,-1.8053195158,5.1460538003,0.4304447335
H,-2.43930707,6.3802495278,2.0652332309
O,-1.1192065817,3.8144104984,-1.2606491134
O,-1.2150176627,4.1068979627,1.0598001055
C,1.2926487078,1.9195086433,-0.2825675284
C,-0.8166201348,0.7532114533,-0.192009027
C,0.5907659665,0.7700973165,-0.4791229656
H,1.0597355528,-0.1620149268,-0.747263256
B,-0.7376082362,3.1956306893,0.0255714129
O,-1.3992226479,1.8769227666,0.170528232
O,0.7290929025,3.0459274437,0.1057274727
C,-1.9052753268,-2.5496124208,-0.5879290199
C,4.8243051167,2.5603668562,-0.0206536432
N,3.4772600986,2.7967379507,0.2598673013
C,3.5891347315,1.0850100856,-1.2742646206
C,-1.5586084849,-0.4163727248,-0.2501570791
N,-0.9730691796,-1.6242151571,-0.6000750783
C,2.754792908,1.9524936642,-0.41273622
H,-3.3320204037,7.7868800657,-1.9175303768
C,5.7882188339,3.2947370421,0.6277328957
C,5.3799863707,4.3533115768,1.5277677448
C,7.2032711326,3.0598264169,0.4676891838
C,6.2580681715,5.135639515,2.1926219633
H,4.3125491941,4.4883493399,1.6287371131
C,8.1475023294,3.7906985943,1.0993408458
H,7.5053515017,2.2477248656,-0.1723381194
C,7.7125153936,4.8971144461,2.0017939545
O,8.5524170255,5.592937963,2.5784710004
C,5.8039520132,6.2597263376,3.1301645739
C,6.329323661,7.6171123803,2.6211006669
C,4.2733105185,6.3478102373,3.2022489813
C,6.3274273305,6.0012911919,4.5573385981
H,7.418876378,7.639326562,2.5976590523
H,5.95038331,7.8241385359,1.614820711
H,5.9781185938,8.4176945336,3.2814319045
H,3.8236881145,5.4268597434,3.5847042817
H,3.9945034395,7.1584713936,3.8820950245
H,3.824390615,6.5650578561,2.2288291337
H,5.9759117683,6.794417494,5.2264210202
H,5.9477184893,5.048336507,4.940675105
H,7.416929845,5.9817242115,4.5848271128
C,9.64579459,3.5210604823,0.9256523128
C,10.3407719465,4.7625289757,0.3305664337
C,10.2824990288,3.1690812307,2.285231227
C,9.9048888797,2.3443558678,-0.0261711274
H,9.9123020947,5.0151183054,-0.6451137405
H,10.2402306658,5.6242195099,0.9896235958
H,11.4068003746,4.5542707523,0.1861048547
H,9.8126495808,2.2772742534,2.7133239101
H,11.3483766292,2.9549624515,2.1487439161
H,10.1809791087,3.9912342749,2.9929593058
H,10.9840532667,2.1893763646,-0.1170210565
H,9.469016006,1.410888499,0.3443186274
H,9.515747271,2.5358502922,-1.031570674
C,-1.5841655891,-3.9488055236,-0.9170177097
C,-0.3044087594,-4.4384760781,-0.6633002009
C,-2.511453154,-4.8078741381,-1.5050233264

C,0.0722671327,-5.7450208546,-0.9650913898
H,0.3972450094,-3.7480740425,-0.2178928094
C,-2.2071833494,-6.1282677494,-1.8345692423
H,-3.4907200817,-4.4162514866,-1.7380977996
C,-0.9050673563,-6.5777750164,-1.54880643
O,-0.6281052698,-7.8889852243,-1.873961126
C,1.5038666172,-6.2353925506,-0.6575044033
C,2.2363230014,-6.6536295506,-1.9529859751
C,2.3625193707,-5.1263993237,-0.027491185
C,1.4862355512,-7.392948843,0.3666692068
H,1.755165708,-7.4624297933,-2.5103761024
H,2.3021744696,-5.8015851232,-2.6351821442
H,3.2544515798,-6.9816368733,-1.7183255027
H,1.9497080707,-4.7803669499,0.9233442346
H,3.365655622,-5.5179532463,0.1678627354
H,2.464977246,-4.2626156485,-0.6888764869
H,2.5096586839,-7.7159203199,0.5848414278
H,1.0294724978,-7.0563933926,1.3014710666
H,0.9274907176,-8.2773138408,0.0474536558
C,-3.2565971305,-7.0450529287,-2.4920911145
C,-2.7689107119,-7.5152727083,-3.8770476596
C,-3.5451115929,-8.2630285015,-1.5916872338
C,-4.5935946957,-6.3192554381,-2.7088836908
H,-2.5962767036,-6.6560681688,-4.5331954107
H,-1.8432974315,-8.0859179345,-3.806576722
H,-3.5313441232,-8.148712719,-4.3447036161
H,-3.9266186388,-7.9370648321,-0.6185735021
H,-4.3078855441,-8.897554291,-2.0573384863
H,-2.6511691516,-8.8638362093,-1.4266890801
H,-5.3013970197,-7.0112181458,-3.1757813439
H,-5.036623888,-5.9808002179,-1.7675560051
H,-4.491759806,-5.4546343571,-3.3710735032
C,-2.9629375496,-0.6126234248,0.0104180455
C,4.919184304,1.4743184795,-1.0251955629
C,3.282713453,0.1252299364,-2.2293335975
C,5.9554108927,0.8735143826,-1.7348934421
H,2.2566211934,-0.1406108099,-2.4511524812
C,5.6494839392,-0.1102411832,-2.6746445492
H,6.9909575724,1.1553296518,-1.5987278909
C,4.3295237441,-0.4792453428,-2.9229372514
H,6.4540340169,-0.5815111733,-3.230446213
H,4.1109058348,-1.2337788193,-3.6716691264
C,-3.1963838889,-1.9928862207,-0.2001952696
C,-4.4680435918,-2.5323192838,0.025283107
C,-4.0011278947,0.2361175497,0.4122743833
C,-5.4912750464,-1.6855057521,0.4259437999
H,-4.6588327861,-3.5939762256,-0.0909878607
C,-5.2592283913,-0.3114950514,0.6097317869
H,-3.812021164,1.2912155379,0.5693010103
H,-6.483487273,-2.0894012737,0.605539535
H,-6.078567357,0.3297091061,0.9217638937
H,0.2818002402,-8.0960249747,-1.6507170411

1c$_{OH}$$^{-}$-4 (open-shell singlet state)

–2636.8880433 hartree

C,-2.4586494711,6.9756041499,-0.7578867878
C,-2.5970805927,7.0570083647,0.622422912
C,-1.877517837,5.847244473,-1.3619214235
C,-2.1592953653,6.0120456831,1.4550357167
H,-3.0505284381,7.9375158332,1.0679899432
C,-1.4495032286,4.8247488477,-0.5396925515
H,-1.7658140655,5.7712651262,-2.4384015461
C,-1.589703009,4.9079513443,0.8560289694
H,-2.2632093044,6.0612480624,2.5339834248
O,-0.8689739813,3.6578129507,-0.8824570197
O,-1.0994690484,3.7945666108,1.4434746529
C,1.3944402148,1.566432922,0.1319891335
C,-0.7610955749,0.5020985551,0.0109115206
C,0.6631296546,0.4618088785,-0.1692620264
H,1.13504833,-0.4527613196,-0.493282847
B,-0.603557571,2.9263571215,0.3748701131
O,-1.3210647528,1.6392246469,0.3855457181
O,0.8463238772,2.7057897284,0.5439240835
C,-2.0161281118,-2.7107619054,-0.5823629321
C,4.9008961562,0.8951509559,-0.3238182794
N,3.5469987157,0.5852748134,-0.4197105372
C,3.7251481869,2.6694248837,0.5425052911
C,-1.5609036382,-0.6162070311,-0.1524299631
N,-1.0278674286,-1.8528124743,-0.490291188
C,2.8546721747,1.57700445,0.0689171546
H,-2.8047675198,7.7934661507,-1.383051445
C,5.8412390368,0.0019748396,-0.7876211882
C,5.4031017725,-1.225134235,-1.4155259578
C,7.2616088457,0.2279206135,-0.6878514898
C,6.2571528442,-2.1463944069,-1.9150482546
H,4.3327703044,-1.3595736286,-1.4741066857
C,8.1841636125,-0.6461675503,-1.1489982263
H,7.590238457,1.1354496984,-0.210761697
C,7.7177509313,-1.9033240769,-1.7985014917
O,8.5351940652,-2.7236794437,-2.2275670132
C,5.7695299397,-3.4292388975,-2.5971782096
C,6.2753402426,-4.6645351647,-1.8253673342
C,4.2370804468,-3.4976316112,-2.6374380825
C,6.2780097482,-3.4812356993,-4.0520053027
H,7.3645279953,-4.7007599345,-1.8047825607
H,5.9036159824,-4.65421621,-0.7952963049
H,5.904618909,-5.5765029928,-2.3065203764
H,3.8005130779,-2.66638162,-3.1986926066
H,3.9339564535,-4.4251325833,-3.1324957897
H,3.7981815297,-3.4987902931,-1.6354847507
H,5.9024360182,-4.387090248,-4.5406847036
H,5.911940266,-2.619094021,-4.6193595878
H,7.3671046844,-3.4908290129,-4.092286963
C,9.6897778102,-0.3897812873,-1.0241955838
C,10.3485411409,-1.5018565388,-0.1832721124
C,10.3364458193,-0.3426090744,-2.4233234385
C,9.9834287435,0.9495225308,-0.3329805399
H,9.9146180632,-1.5349740743,0.8217168715
H,10.2208593019,-2.477458855,-0.6513316925
H,11.4207401932,-1.300574806,-0.0802802278
H,9.8934397218,0.4563983271,-3.0272180162
H,11.4084563072,-0.1368910327,-2.3275056678
H,10.208988214,-1.288825329,-2.9482666092
H,11.066731285,1.0879763101,-0.2678275706
H,9.5773570061,1.7986550375,-0.8923731168
H,9.5865647532,0.9823607651,0.6868954275
C,-1.7699922992,-4.1238977304,-0.9156484031
C,-0.5764499164,-4.7268093005,-0.524646218
C,-2.6898603064,-4.8870808487,-1.6331107416
C,-0.279680999,-6.0567144103,-0.8141429704

H,0.1219703271,-4.1091529334,0.0215069094
C,-2.4584059703,-6.2207951123,-1.9670003482
H,-3.5983685081,-4.4069767794,-1.9666309468
C,-1.2423993649,-6.7867184204,-1.5418697993
O,-1.0374744737,-8.1089327936,-1.8734653501
C,1.0488762015,-6.6827359868,-0.3370671405
C,1.9164722806,-7.1276299121,-1.5366431755
C,1.9024781122,-5.6737836473,0.4483522642
C,0.7894265449,-7.8630239656,0.6264637838
H,1.4584890651,-7.8836045173,-2.1821531681
H,2.1408945931,-6.2683487,-2.17403774
H,2.8646229127,-7.5424771004,-1.1793775846
H,1.3893346539,-5.3169352028,1.344892819
H,2.8301312882,-6.1598593586,0.7656285658
H,2.1728301679,-4.8066084233,-0.1585207094
H,1.7414689931,-8.2864933046,0.9635312323
H,0.2435322527,-7.5120877172,1.5067241648
H,0.2011122956,-8.6783301463,0.1972133461
C,-3.4917565532,-7.0287575033,-2.7752571193
C,-2.8801509622,-7.5041039127,-4.1082662638
C,-3.9848424473,-8.2398731662,-1.9582779294
C,-4.7293332558,-6.1868460985,-3.1230487112
H,-2.5592784299,-6.6468245017,-4.7087333763
H,-2.0198391625,-8.1533149224,-3.9480955255
H,-3.6295275478,-8.0583021048,-4.6849821045
H,-4.4540266923,-7.9070843786,-1.0266298072
H,-4.7341843351,-8.7961198035,-2.5330882282
H,-3.1691269393,-8.9179343722,-1.7089124598
H,-5.4303329776,-6.8047809104,-3.6927615971
H,-5.2527049252,-5.8341562316,-2.2293375253
H,-4.4785137174,-5.3186742358,-3.7393185227
C,-2.9945835373,-0.7237215444,-0.0126266503
C,5.041897899,2.2362602552,0.2990076304
C,3.4496784571,3.9005974341,1.1216349261
C,6.1062189886,3.0659432804,0.6406041782
H,2.4251897167,4.2041102521,1.2914609781
C,5.8346328185,4.3040182509,1.2243589373
H,7.1385536948,2.7923197574,0.469168326
C,4.5262475781,4.7174774551,1.463914185
H,6.6618463753,4.9539774316,1.4926722266
H,4.3411478059,5.6861096755,1.9170735595
C,-3.2984858913,-2.0775557038,-0.2869126285
C,-4.6151019332,-2.5380767946,-0.1878910817
C,-4.0062241352,0.1805674259,0.3288153586
C,-5.6135217519,-1.6360185939,0.1532763352
H,-4.8611256262,-3.5811686934,-0.3566887706
C,-5.3100961212,-0.2873243498,0.4016352289
H,-3.7644564425,1.2156431506,0.5368228578
H,-6.6414331553,-1.9775289439,0.2346189732
H,-6.1099485088,0.3987298222,0.6651274288
H,-0.1537185506,-8.3775584909,-1.6127888716

1c^{2-}-1 (closed-shell singlet state)

–2636.2760415 hartree

C,0.1055825828,9.0708763695,1.4324187836
C,0.2402547655,8.5285128381,2.704349164
C,-0.0101621731,8.2409971658,0.3023352068
C,0.2644357434,7.1354442658,2.8974724785
H,0.3292009297,9.1861689517,3.5647533807
C,0.0146305459,6.8755967054,0.4972459642
H,-0.1160211741,8.6505889893,-0.6969554713
C,0.1501781357,6.3251007759,1.7857188304
H,0.3702363122,6.6994178949,3.8855615225
O,-0.0777168602,5.8982007125,-0.4236829514
O,0.1469682641,4.9831175173,1.7230411754
C,1.15957635,2.5318278941,-0.1434197539
C,-1.2412202136,2.5424186431,0.211908883
C,-0.0329559846,1.8469767538,0.108248077
H,-0.0250430665,0.7774727523,0.1766877285
B,-0.0044355734,4.620317272,0.288767822
O,-1.2494845563,3.8580392327,0.0883424719
O,1.166667508,3.8525245358,-0.1688887571
C,-4.1199085639,0.2795187415,0.600666444
C,-4.6544345208,1.5838606718,0.1535132444
C,4.5650927206,1.6047429157,-0.4903103801
C,3.9986590137,0.262714785,-0.7454920532
N,3.5638424865,2.4893764068,-0.3176390289
C,-2.7217757099,0.4598489859,0.7125464292
C,2.5953636333,0.4204951525,-0.6652530079
C,-2.4837748211,1.862918592,0.3332981724
N,-3.6471568802,2.4740218501,0.0602985385
C,2.3858349611,1.8477083442,-0.3652170554
H,0.0892620255,10.1496934941,1.3041819427
C,1.7543022086,-0.6536148895,-0.9600566394
C,4.543776188,-0.9687207371,-1.1069646044
C,3.696522891,-2.0432379269,-1.3619107344
H,4.1182810588,-3.0035781771,-1.643820882
C,2.3120304964,-1.8851744998,-1.2892650621
H,1.6582752688,-2.7220365009,-1.5167434118
H,5.6107669026,-1.1035380524,-1.2252655031
H,0.6773825887,-0.5474925205,-0.9792430055
C,-4.7007646135,-0.9289739268,0.9836184223
C,-1.922580864,-0.5667620865,1.21747589
C,-2.510885148,-1.7784704247,1.5660641125
H,-1.8902956594,-2.5795400738,1.9567701723
C,-3.8898072675,-1.9597395631,1.449700681
H,-4.3392945359,-2.9022128112,1.7485232854
H,-0.8612555809,-0.4335171604,1.3843594427
H,-5.7724211078,-1.0761715452,0.9575858006
C,5.9209924524,2.0105058815,-0.4151424793
C,6.2309251276,3.4011588656,-0.3771486269
C,7.0107221795,1.1012237855,-0.3445842045
C,7.5089198968,3.8816367981,-0.3204379221
H,5.381684504,4.0707202377,-0.4069585042
C,8.3178047403,1.5012534699,-0.2740944916
H,6.7869358804,0.0468243252,-0.3021491071
C,8.6315027007,2.9359202981,-0.279471715
O,9.8223985832,3.3350324333,-0.2410221846
C,7.8031934364,5.3878777887,-0.3015457085
C,8.6542031919,5.7754503135,-1.5275373209
C,6.5181607974,6.2261468515,-0.3426799375
C,8.562429262,5.7608112608,0.9876908753
H,9.5974902154,5.2283040041,-1.5311894182
H,8.1127902407,5.5527542686,-2.4536501192
H,8.8654363461,6.8522171103,-1.5113458224
H,5.8681558671,6.0289562226,0.5146164381
H,6.7819529567,7.289152877,-0.3225433196
H,5.9372933996,6.0454677895,-1.2520368627
H,8.7737639711,6.8375318936,0.999410599
H,7.9550183955,5.5278748443,1.8689733404

H,9.503392205,5.2136379725,1.0536577525
C,9.469040087,0.4907018097,-0.1794211322
C,10.4095434912,0.6464716431,-1.3915278799
C,10.2673421638,0.7151811955,1.1203524149
C,8.9701028442,-0.9617066081,-0.1681872759
H,9.8684734653,0.4482827213,-2.323503048
H,10.8149812011,1.6576892052,-1.4305565849
H,11.2380905559,-0.0701696888,-1.3208054661
H,9.6244789004,0.5664536191,1.9949459602
H,11.0965845549,-0.0013454652,1.182495004
H,10.6698561232,1.727928156,1.1500291237
H,9.8301517628,-1.6366435815,-0.0975160805
H,8.3171593214,-1.1660098069,0.6866156693
H,8.4262997913,-1.2151605959,-1.084473684
C,-5.9876229468,1.9513774268,-0.1565491627
C,-6.3035751534,3.3269533962,-0.3539615139
C,-7.0433700766,1.0139971551,-0.3173596828
C,-7.5629080176,3.7711188019,-0.644385066
H,-5.47685979,4.0167836583,-0.2483516079
C,-8.3282959205,1.3762649782,-0.6196629043
H,-6.8037541748,-0.0349047535,-0.2386604632
C,-8.6549122958,2.7989334271,-0.7801151299
O,-9.8296664632,3.1656908278,-1.0343840727
C,-7.8677626895,5.2638907837,-0.8298627137
C,-8.9027758682,5.7260256132,0.2153894523
C,-6.6141816288,6.1329305289,-0.6594716122
C,-8.4219950461,5.5171600923,-2.2463128035
H,-9.8289139927,5.1585833255,0.1183459381
H,-8.5089631107,5.5894783184,1.2285709647
H,-9.1212106525,6.7928954001,0.0798249328
H,-5.8371139718,5.8841234277,-1.3880565193
H,-6.8836961369,7.1846101607,-0.8057094071
H,-6.1790796004,6.0378848334,0.3397544691
H,-8.6414056368,6.5843782129,-2.377151732
H,-7.6826338752,5.2306110342,-3.0021374467
H,-9.3349638281,4.9442405574,-2.4118336853
C,-9.4396017625,0.3343981414,-0.8071207888
C,-10.5589621062,0.563793078,0.2284583527
C,-10.0283091168,0.4358139326,-2.2284530236
C,-8.9289012698,-1.1020503352,-0.6217265873
H,-10.1675049518,0.4529462984,1.2458417827
H,-10.9764403833,1.5651393631,0.1213962714
H,-11.3585781184,-0.1754140511,0.089769263
H,-9.2555825016,0.2330869467,-2.978203146
H,-10.8299270239,-0.3026847483,-2.3586268901
H,-10.4323304838,1.4332344487,-2.4030777248
H,-9.7599322961,-1.8007203047,-0.7681972231
H,-8.1487787527,-1.3582323013,-1.3459710653
H,-8.5313214651,-1.2687376932,0.3850901286

1c^{2-}-2 (closed-shell singlet state)
–2636.2906303 hartree
C,0.2405647252,9.1855845955,0.1919239804
C,0.2367033426,8.7915767715,1.5241421728
C,0.197309718,8.2323864439,-0.8423078662
C,0.1896350158,7.4303436572,1.8758045236
H,0.2725740857,9.5422876328,2.3089198779
C,0.1516446452,6.8984925004,-0.4923569791
H,0.2023895912,8.5253405008,-1.8873227797
C,0.1478374507,6.498036451,0.8579337434
H,0.1880452785,7.1100296728,2.9126233979
O,0.1032261293,5.8240374923,-1.3005235652
O,0.0976087975,5.1602514718,0.9490724521
C,1.1535526654,2.4600171379,-0.5407768084
C,-1.2612324727,2.585609342,-0.4919253408
C,-0.0717692296,1.8374852169,-0.3973204186
H,-0.1708609024,0.7833243298,-0.2112197496
B,0.0607380276,4.6292471394,-0.4429952613
O,-1.2003530784,3.9044498058,-0.6743669094
O,1.2245810209,3.7764892402,-0.7019543883
C,-4.7522901566,1.5357778327,-0.4916774247
C,-3.9477290387,0.3010572666,-0.313011731
C,4.5670788923,1.4666638247,-0.4443351328
C,4.0031980182,0.1000710368,-0.5422554037
N,3.5542972748,2.3734894222,-0.4775080054
C,-3.8156220432,2.5887157913,-0.5702450937
C,2.602571819,0.2720052927,-0.5986418583
C,-2.5096961095,1.9583546596,-0.4524251381
N,-2.6507276842,0.6068652686,-0.3132932055
C,2.3898024554,1.7361307372,-0.5326059619
H,0.2797194849,10.2420469932,-0.0590580949
C,1.7656231722,-0.8293822879,-0.7633661741
C,4.5536215829,-1.175811925,-0.6471531282
C,3.7077462653,-2.2736685601,-0.7841344645
H,4.1325700607,-3.269943956,-0.8626897684
C,2.326031066,-2.1016639027,-0.8405486536
H,1.6741376552,-2.9613801453,-0.960207443
H,5.6228071247,-1.3397991163,-0.6476400138
H,0.6928046599,-0.7283060431,-0.8548712473
C,-6.1099668119,1.8170195442,-0.6426591685
C,-4.2141367566,3.9110229653,-0.7572033625
C,-5.5743155969,4.1762836313,-0.8779198236
H,-5.911318382,5.1987292774,-1.0235849385
C,-6.5104545702,3.138734565,-0.8293064426
H,-7.5675022044,3.3622767153,-0.9448486465
H,-3.4715220301,4.6971479528,-0.8140137076
H,-6.8590389133,1.0356760371,-0.6377645994
C,5.8989468352,1.8944952133,-0.3267833801
C,6.1864594165,3.2961457974,-0.3863335684
C,7.0021816121,1.0108351301,-0.1300286783
C,7.4473374265,3.8037723137,-0.3016934398
H,5.3279620648,3.9413109857,-0.5142177529
C,8.292784998,1.438532566,-0.0273330662
H,6.7929235712,-0.0414250174,-0.0276611139
C,8.5820135541,2.8810343904,-0.1287164021
O,9.7575063221,3.3021300338,-0.0613140878
C,7.7188983621,5.3116971244,-0.3848034625
C,8.6261111599,5.6199926731,-1.5928413191
C,6.4239966371,6.1175492404,-0.5564964534
C,8.4031867143,5.7972235027,0.9088445347
H,9.5804194202,5.0996939708,-1.5059542622
H,8.1395064704,5.3119356684,-2.5247115667
H,8.8136345435,6.6992509174,-1.6521972586
H,5.7335940882,5.9746093615,0.279637492
H,6.6689472919,7.1838033332,-0.6061672176
H,5.89396158,5.8558909424,-1.4769714251
H,8.5905321416,6.8763900308,0.8487743271
H,7.7564871203,5.6166120703,1.7741765691
H,9.3516212294,5.2823327052,1.0647365429
C,9.4553297871,0.4630659391,0.1988562457

C,10.4519707199,0.5466059551,-0.9749089247
C,10.1826391394,0.7999660461,1.515924612
C,8.9783794821,-0.9934579081,0.2951173038
H,9.9602407408,0.2744193452,-1.9153106376
H,10.8510589812,1.5565615233,-1.0686498793
H,11.2824826852,-0.1518419818,-0.8122635683
H,9.497920509,0.7095137268,2.3661949823
H,11.0140506434,0.1017846739,1.6747111511
H,10.5749654388,1.8166217612,1.4894584169
H,9.8437185326,-1.6445202805,0.4585750768
H,8.2872885117,-1.1459636396,1.1304175569
H,8.4856751957,-1.3251825633,-0.6248935753
C,-4.4019237185,-1.0526299362,-0.1770882784
C,-3.4757307761,-2.1172358375,-0.3053907706
C,-5.7418233975,-1.4007850056,0.1091242282
C,-3.8267006745,-3.4394184002,-0.1986352632
H,-2.4526877176,-1.8307983423,-0.5092846459
C,-6.1715449541,-2.7005210316,0.2429025921
H,-6.4485646137,-0.6010441521,0.2706804489
C,-5.2185979335,-3.7950399797,0.0757954968
O,-5.5832930277,-5.0021151166,0.1713595652
C,-2.7925193089,-4.5618891792,-0.366208464
C,-3.1836084376,-5.4684780506,-1.5502773567
C,-1.3836823891,-4.0179123316,-0.6442311692
C,-2.7177030486,-5.4070531537,0.9211011097
H,-4.1734951758,-5.8966085576,-1.3884756814
H,-3.1953161497,-4.8937407534,-2.4831913767
H,-2.4537180332,-6.2811563525,-1.6618687961
H,-1.0206652895,-3.3860344048,0.1724758234
H,-0.6888162697,-4.858884739,-0.7507522844
H,-1.3456760655,-3.4341653101,-1.569610747
H,-1.9895144062,-6.2195660965,0.7983050886
H,-2.3946990775,-4.7881309635,1.7654765926
H,-3.6944467871,-5.834068173,1.1510935514
C,-7.6333107829,-3.0347844633,0.5736857418
C,-8.2491888007,-3.8827496351,-0.5574402001
C,-7.7105080831,-3.8189905741,1.8988229111
C,-8.5016833796,-1.7779449522,0.7311859238
H,-8.2486273468,-3.3215497303,-1.4987434572
H,-7.6751744638,-4.7996043902,-0.6952428519
H,-9.28901647,-4.1404551452,-0.3156277671
H,-7.3222990065,-3.2123591061,2.7246378698
H,-8.7531794307,-4.0772052019,2.1272109405
H,-7.122443035,-4.734710811,1.8293891944
H,-9.5290980065,-2.0775340706,0.9671864853
H,-8.1489737973,-1.1331966377,1.5428209702
H,-8.5335647962,-1.1842977054,-0.1887217048

1c^{2-}-3 (closed-shell singlet state)
-2636.296186 hartree
C,-0.0191189691,9.2028072374,0.229300681
C,-0.0105296236,8.8562342886,1.5746414755
C,-0.0237699912,8.2122924251,-0.7703891082
C,-0.0063017074,7.507472847,1.9744116561
H,-0.0069003594,9.6350569582,2.3323709238
C,-0.0194827504,6.8904795957,-0.3735696571
H,-0.0305592107,8.4679773495,-1.8252105808
C,-0.0108583556,6.5378238247,0.9905344956
H,0.0005338175,7.2244160212,3.0220108078
O,-0.0228729117,5.7877551472,-1.1432816768
O,-0.0087332141,5.2044144471,1.1296194888
C,1.1971591614,2.5003578021,-0.2597606875
C,-1.2130846352,2.4882918171,-0.2864758663
C,-0.0059627344,1.8010920918,-0.1565742137
H,-0.0024272694,0.7312477949,-0.0062918674
B,-0.014804083,4.6157131349,-0.2448951375
O,-1.2210323363,3.8176492531,-0.4545763986
O,1.1919935031,3.8241028139,-0.4715867665
C,-4.7254296882,1.4739307913,-0.3175335949
C,-3.9407015477,0.2189405376,-0.192226921
C,3.9445377036,0.2682223044,-0.0129695176
C,4.7167264047,1.5146922522,-0.2501955177
N,2.6298498778,0.5497066886,0.0182697115
C,-3.7701307871,2.5088021093,-0.3655003245
C,3.7519989566,2.5342148691,-0.374840739
C,-2.4688963489,1.8433581709,-0.2775928182
N,-2.62619059,0.5057990814,-0.1932013042
C,2.4585802267,1.8709173563,-0.200393402
H,-0.0222505524,10.2504218369,-0.0590166961
C,4.1171542494,3.8614173557,-0.58697874
C,6.0707530464,1.8425713189,-0.293110197
C,6.4383646745,3.1704359173,-0.5056706991
H,7.4927073806,3.4302555155,-0.5427546917
C,5.473281266,4.167844326,-0.6596683875
H,5.7822950125,5.196382995,-0.8225140503
H,6.8446370877,1.100849553,-0.1449606331
H,3.3554112146,4.6240314454,-0.680224528
C,-6.0778922517,1.7824914131,-0.4539333763
C,-4.1464842374,3.8426774281,-0.5027050191
C,-5.5032572459,4.1372015543,-0.605612179
H,-5.820704082,5.1704350715,-0.7130246554
C,-6.4566969307,3.1171845731,-0.5903427808
H,-7.5101318079,3.3623159252,-0.6937859922
H,-3.3917412328,4.6169441277,-0.5383444219
H,-6.8410873672,1.0158140839,-0.4785206631
C,4.4369790144,-1.0462342268,0.1930221538
C,3.5796259329,-2.0493749345,0.727189328
C,5.7684734674,-1.4305911771,-0.1191971139
C,3.9953664131,-3.3246074728,0.9941845596
H,2.5656529075,-1.7385246982,0.9404935623
C,6.2539264992,-2.693360976,0.0899955246
H,6.4052455184,-0.69861371,-0.591275919
C,5.3787736963,-3.708884013,0.6872207275
O,5.8025264146,-4.8693494657,0.9189099616
C,3.0555296114,-4.3666124627,1.6153982929
C,3.6238481162,-4.8542390184,2.9634427855
C,1.6574130762,-3.793712669,1.8823387105
C,2.8941848487,-5.5677410916,0.6622110256
H,4.608318086,-5.3029619722,2.8278775721
H,3.7104243667,-4.0179668443,3.6662107122
H,2.9492803346,-5.5979665903,3.4058941675
H,1.1721065441,-3.4498747364,0.964938099
H,1.0241216528,-4.5741441393,2.3170325045
H,1.6848111771,-2.9560425616,2.5862091855
H,2.2254950593,-6.3136713475,1.1092738804
H,2.4506985988,-5.2456783261,-0.285998064
H,3.8601986808,-6.0317090643,0.4603872002
C,7.6891489026,-3.0819742576,-0.2906703135
C,8.4659182183,-3.5300172212,0.9638981422
C,7.6724355209,-4.230127961,-1.3194450772

C,8.4628704965,-1.9128317584,-0.9174529491
H,8.5254676183,-2.7112221227,1.6896600889
H,7.9700603995,-4.3793193911,1.4343426353
H,9.4897649028,-3.8174693997,0.6912258886
H,7.161616841,-3.9152869677,-2.2360907895
H,8.6989984575,-4.5156950618,-1.5834589542
H,7.1554324697,-5.0990720343,-0.9116602481
H,9.475005259,-2.2466698374,-1.1714768304
H,7.9912227755,-1.5533411095,-1.8377677792
H,8.5577792957,-1.0675287278,-0.2275445965
C,-4.4179543736,-1.1140573657,-0.1098328012
C,-3.5210244742,-2.2023323272,-0.3068633284
C,-5.7726647649,-1.434295855,0.1746998904
C,-3.9161496797,-3.5112645295,-0.2849590242
H,-2.491387426,-1.9351467128,-0.5035982371
C,-6.2428601568,-2.7180369186,0.2405693026
H,-6.4453948737,-0.6208488538,0.3972091545
C,-5.3227292301,-3.8332130513,-0.0126208563
O,-5.7291597604,-5.0221226624,0.0118230426
C,-2.9291051081,-4.6568932567,-0.5460235222
C,-3.3764197067,-5.4699164061,-1.7775981395
C,-1.5101361301,-4.1420960567,-0.8206815444
C,-2.8592840188,-5.5848347987,0.6834140217
H,-4.3710506237,-5.8892498218,-1.6232184599
H,-3.3958977653,-4.8329541358,-2.6690056072
H,-2.6681899301,-6.287179013,-1.96282907
H,-1.1086865033,-3.5746189097,0.0233249881
H,-0.8431753567,-4.9934293271,-0.9918138402
H,-1.4696111001,-3.5033525098,-1.7084424916
H,-2.1556707813,-6.4048513515,0.4941384112
H,-2.5020866374,-5.0320968015,1.5589062795
H,-3.8412362721,-6.0031193427,0.9068753253
C,-7.7073336938,-3.0270518173,0.5804646464
C,-8.3731029541,-3.7816259019,-0.588044704
C,-7.7853683726,-3.8883713105,1.856749786
C,-8.5281432195,-1.7538647246,0.8327989814
H,-8.3655887062,-3.1660768792,-1.4945482991
H,-7.8415112537,-4.711326611,-0.7918815406
H,-9.4180317226,-4.0115882675,-0.342190234
H,-7.3556158307,-3.3500302912,2.7086769527
H,-8.8322526156,-4.1187228258,2.0934412556
H,-7.2382052846,-4.821458059,1.7204058859
H,-9.5600164114,-2.033091224,1.0728744161
H,-8.1374973504,-1.1721355875,1.6740077597
H,-8.5587335474,-1.1046613267,-0.0487534036

1c^{2-}-1 (open-shell singlet state)

–2636.2956417 hartree

C,0.052772051,9.0104305422,0.9536546768
C,0.1997679837,8.5851894146,2.2682327598
C,-0.0604523557,8.0830015759,-0.0975362937
C,0.2392143553,7.2156956142,2.5852127104
H,0.2865392413,9.3180070495,3.0656044067
C,-0.0204898062,6.7414050945,0.2196362418
H,-0.1760197503,8.4007846961,-1.1285082974
C,0.1272980495,6.3104947122,1.5499512117
H,0.354634829,6.8701624399,3.6072516283
O,-0.1076372357,5.6815771647,-0.6087183924
O,0.1373283822,4.9652098512,1.6054837498
C,1.1640461231,2.3756292984,-0.1318258776
C,-1.2314969996,2.3745648301,0.2073528186
C,-0.0206381116,1.6823507091,0.1297102201
H,-0.0071881894,0.6116052263,0.1966949494
B,-0.0143467446,4.4802757342,0.2188705036
O,-1.2474221588,3.6773718037,0.0931598503
O,1.1650955338,3.6819143318,-0.1749423655
C,-4.1915921534,0.2024769062,0.6699917439
C,-4.6830972612,1.4906763488,0.1855155482
C,4.6050357009,1.5218050019,-0.4862993167
C,4.0778027768,0.1979616275,-0.8108171973
N,3.5776479763,2.3753908923,-0.2681037252
C,-2.7805949601,0.3450291398,0.7675199894
C,2.663781543,0.321816291,-0.7343607576
C,-2.5064500305,1.7096496667,0.344509595
N,-3.6430842993,2.3451529579,0.0428770625
C,2.4226197397,1.7104385505,-0.374467055
H,0.0246588096,10.0732394392,0.7302405352
C,1.8436961513,-0.7645446648,-1.0654396508
C,4.6449219677,-1.0189914257,-1.2113543701
C,3.8199347796,-2.0935958819,-1.510115921
H,4.2601598034,-3.036841011,-1.8212993089
C,2.4257801609,-1.9664598791,-1.4384545903
H,1.7923570909,-2.8104304302,-1.6960285193
H,5.7156300879,-1.1367511003,-1.3201123763
H,0.7640518483,-0.6778702219,-1.0670097978
C,-4.7964766578,-0.9923831679,1.0812726761
C,-2.0021278411,-0.7018907523,1.2775577541
C,-2.6182015994,-1.8844853735,1.6577715762
H,-2.0169362594,-2.6979947896,2.0534417151
C,-4.0089383039,-2.0295032157,1.5595774392
H,-4.4784766607,-2.9557721565,1.8787402078
H,-0.9324189237,-0.5979263823,1.4119481377
H,-5.8714165579,-1.1195986327,1.0604054283
C,5.9511938414,1.9790343432,-0.3743045094
C,6.2054049086,3.3741412699,-0.2329683444
C,7.0742312811,1.1106683273,-0.3732430647
C,7.4637788669,3.8985947407,-0.1345212652
H,5.3298555664,4.0087496562,-0.2153404425
C,8.366180942,1.5563185901,-0.2714198514
H,6.8893203749,0.0493619742,-0.4218406572
C,8.6210468091,2.9965721104,-0.1623482267
O,9.7968363032,3.4389825671,-0.0873585035
C,7.697324678,5.4093713656,0.0013486699
C,8.5325320914,5.9237374166,-1.1883177483
C,6.3793357878,6.1958501004,0.0198673257
C,8.4397823424,5.7117293308,1.3185412604
H,9.4996696782,5.421730888,-1.2284757065
H,8.0033912194,5.7470475664,-2.1311631029
H,8.6948960746,7.0045228264,-1.0908354472
H,5.7375416664,5.907317803,0.8571447993
H,6.6002150106,7.2637640044,0.1225171064
H,5.8065839634,6.0624234427,-0.9024909297
H,8.6015580702,6.7925293497,1.4160985737
H,7.844202907,5.3823873859,2.1768336945
H,9.4048604029,5.2048675397,1.3445767655
C,9.5558504565,0.5867457947,-0.2588592096
C,10.4861056796,0.8770328221,-1.4539347002
C,10.3473991406,0.7359573032,1.0557891652
C,9.1123174676,-0.8797045795,-0.3660257523
H,9.9493483793,0.7398097434,-2.3991460645

H,10.8582936559,1.9005794402,-1.4084383313
H,11.3378746614,0.1849006801,-1.4439736949
H,9.7110103106,0.4981416132,1.9152211586
H,11.1990838631,0.0437518531,1.0625117931
H,10.7174312443,1.7554002088,1.1660367068
H,9.9979047639,-1.5243758677,-0.3507864812
H,8.4706418348,-1.1775517974,0.4693480243
H,8.5749928043,-1.0781955452,-1.2991820607
C,-6.0068572215,1.9147513346,-0.1323995915
C,-6.2530449794,3.292240738,-0.4019388393
C,-7.1118750021,1.0279696164,-0.2208252894
C,-7.4919621053,3.7855226388,-0.7021685413
H,-5.3890392958,3.9403876381,-0.3466508159
C,-8.3827930876,1.4418769103,-0.5236047057
H,-6.9253466448,-0.0240827835,-0.0744631652
C,-8.6339748138,2.8662244484,-0.7665577209
O,-9.7929766389,3.2806949441,-1.0292621384
C,-7.719755014,5.2793241798,-0.9707935677
C,-8.7130607376,5.8556501991,0.0581001674
C,-6.4191255122,6.0876005755,-0.8655791763
C,-8.2802548307,5.4796896555,-2.3932273804
H,-9.6717475857,5.3391788808,0.0020827738
H,-8.31458677,5.7518230151,1.0732461996
H,-8.8712576303,6.9243625435,-0.133731821
H,-5.6674687879,5.7561066896,-1.5877253075
H,-6.6347101869,7.1421146296,-1.0691151732
H,-5.9748109771,6.0262777189,0.1320711382
H,-8.4387896414,6.5483489731,-2.5849076259
H,-7.5707269992,5.1059469765,-3.139443428
H,-9.2275722787,4.9534002924,-2.5139792743
C,-9.5513898424,0.4521055885,-0.6251619907
C,-10.6354735167,0.8073574557,0.4123367484
C,-10.1615481909,0.4954185154,-2.0404141211
C,-9.1131793303,-0.9950647049,-0.3562028577
H,-10.2285239596,0.745723809,1.4276992228
H,-11.007335762,1.8183466353,0.2453407277
H,-11.472121628,0.1007635872,0.3387308578
H,-9.4141729192,0.2096419317,-2.7887145209
H,-10.9989660859,-0.2105358628,-2.1106380785
H,-10.5219429095,1.4980378624,-2.2710975706
H,-9.9830508014,-1.6553040385,-0.4428780962
H,-8.363075869,-1.338334147,-1.0758041259
H,-8.7035834069,-1.1185783491,0.6517084456

$1c^{2-}$-2 (open-shell singlet state)
–2636.3065915 hartree
C,0.2072053958,9.1351560413,0.0020029097
C,0.2150081049,8.8111915921,1.353130707
C,0.1704140286,8.1301745566,-0.9814762925
C,0.1863708568,7.4702108708,1.7757969866
H,0.2465408243,9.6022390178,2.0971779676
C,0.1427363626,6.8166755751,-0.5609789238
H,0.1674229002,8.3683578154,-2.0401401638
C,0.1506401914,6.4881056324,0.8068117632
H,0.1951618808,7.2037110772,2.8275339065
O,0.1033645867,5.6977934934,-1.3106471818
O,0.1172554943,5.1528096854,0.9660553995
C,1.1751895628,2.3793865923,-0.4775487717
C,-1.2311329724,2.5006025416,-0.4059395601
C,-0.0616141587,1.7518289478,-0.3136679081
H,-0.1502300312,0.6940687347,-0.1415565375
B,0.0762975026,4.5576955324,-0.3880590714
O,-1.1854168514,3.8067515504,-0.5691632377
O,1.2342828363,3.6849714462,-0.61188688
C,-4.7860410035,1.5418333789,-0.355347096
C,-4.0217856655,0.2946642468,-0.2355922039
C,4.6076678944,1.4416667668,-0.4773218222
C,4.0743317442,0.0893105446,-0.6497110473
N,3.5868491505,2.3274636355,-0.4391926472
C,-3.8165296632,2.573274137,-0.4334560098
C,2.6621172233,0.2373140932,-0.6795133608
C,-2.5356009918,1.9022977684,-0.3658109737
N,-2.6976725063,0.5756708702,-0.2605700037
C,2.4260413882,1.6678108774,-0.5292214158
H,0.232883239,10.177474913,-0.3033477556
C,1.8426276729,-0.8775720931,-0.8933047793
C,4.6423599326,-1.177054762,-0.8361261928
C,3.8162587288,-2.2758519094,-1.0256440312
H,4.2561448656,-3.2588681725,-1.1684829631
C,2.4237048629,-2.1267363644,-1.0529739261
H,1.789104241,-2.9932055755,-1.2140476131
H,5.7151094265,-1.3204819356,-0.8590873601
H,0.7660313525,-0.7868040931,-0.9593586368
C,-6.1436603355,1.8777334119,-0.4393400311
C,-4.1878902537,3.9149600808,-0.5642815311
C,-5.5381223106,4.2243847169,-0.6264630927
H,-5.8483497727,5.2605226094,-0.7278697147
C,-6.507066671,3.2116519961,-0.5704252673
H,-7.5601742625,3.4718077137,-0.6349524202
H,-3.4277173488,4.6838140481,-0.6212424392
H,-6.9223817474,1.1259282678,-0.4200668415
C,5.9525655344,1.8973993543,-0.3525148025
C,6.2191448645,3.2974444823,-0.3776492564
C,7.0623812733,1.028492854,-0.182217722
C,7.4778304325,3.8210871072,-0.2812224832
H,5.3524805019,3.9349993268,-0.4871523271
C,8.3534326204,1.4742870783,-0.071176951
H,6.866188314,-0.0290392135,-0.1064880751
C,8.6225127609,2.9147992764,-0.1331392075
O,9.7988458569,3.3550419085,-0.0539040574
C,7.7251983246,5.3351292001,-0.3272042142
C,8.6275193571,5.6867661525,-1.5270454327
C,6.4191751313,6.1271805197,-0.4787357515
C,8.4033030872,5.7985201558,0.9777902649
H,9.5880113431,5.1760916446,-1.4517334587
H,8.1449506875,5.3946814486,-2.4662163475
H,8.8002739944,6.769930211,-1.559721071
H,5.7312821683,5.9529784919,0.3535249097
H,6.6495200167,7.1978716571,-0.5018228178
H,5.892661515,5.8804565661,-1.4053239749
H,8.5761057305,6.881590954,0.9447208216
H,7.7595785703,5.5869982632,1.8384372898
H,9.3579953694,5.2910481511,1.1192820942
C,9.5276408489,0.5056008825,0.1252134733
C,10.5203937797,0.6349150916,-1.0476609462
C,10.2544484699,0.815050318,1.4492738407
C,9.0700309879,-0.9592968612,0.1818692548
H,10.0295438476,0.3826738454,-1.9941365678
H,10.9040530613,1.6531751046,-1.1129765107
H,11.3608771498,-0.0567395918,-0.9066464267

H,9.5728270247,0.6923529998,2.2980922872
H,11.0954941703,0.1235010527,1.5870502313
H,10.631935954,1.8377437318,1.4485869487
H,9.9447540209,-1.6031944937,0.3245154799
H,8.3830399232,-1.1439146587,1.0138608279
H,8.5777984784,-1.2710657898,-0.7451385237
C,-4.4700788704,-1.0536414168,-0.1147783646
C,-3.5245463512,-2.116771615,-0.182316335
C,-5.8288746174,-1.4110166166,0.0856038427
C,-3.8702955919,-3.4363250971,-0.0918733767
H,-2.4946777695,-1.8192151734,-0.3212609147
C,-6.2565810708,-2.708858424,0.1941484723
H,-6.5487976709,-0.6146265538,0.1833205626
C,-5.2797504169,-3.7971585203,0.096182721
O,-5.6412531607,-5.0004668011,0.1768389934
C,-2.8208404569,-4.5523593562,-0.1857580424
C,-3.1307572609,-5.4693952918,-1.3858622389
C,-1.4030340115,-3.9964489551,-0.3802427776
C,-2.8201664185,-5.3864136398,1.1110199164
H,-4.1180083823,-5.9202426714,-1.2816553719
H,-3.1001820293,-4.8990289621,-2.3207335223
H,-2.379182339,-6.2661231707,-1.4520654319
H,-1.0937790686,-3.356007285,0.4510631167
H,-0.6953522356,-4.8305648778,-0.4378204663
H,-1.3124505999,-3.4189846938,-1.305490019
H,-2.0694609593,-6.1837203615,1.0429951356
H,-2.5654854957,-4.7567204921,1.9703942003
H,-3.7994365078,-5.8345421125,1.2814924432
C,-7.7344555363,-3.0543989089,0.4230387522
C,-8.2630709978,-3.9114523875,-0.7447464026
C,-7.8982826715,-3.8310442211,1.7448110906
C,-8.6182940777,-1.801578609,0.5117766018
H,-8.1893438288,-3.3596545519,-1.6883932158
H,-7.688048533,-4.8334034985,-0.8325551371
H,-9.3189192604,-4.1617558903,-0.5797528464
H,-7.5623446365,-3.2217954738,2.591082033
H,-8.9546749906,-4.0816962172,1.9055087927
H,-7.3142521615,-4.7512819706,1.7223930216
H,-9.657596326,-2.1065700855,0.6760101105
H,-8.3280596725,-1.1515233971,1.3434046315
H,-8.588374578,-1.2126802558,-0.4108457688

1c^{2-}-3 (open-shell singlet state)

–2636.3118934 hartree
C,-0.0162053524,9.2092099909,0.1696496932
C,0.012792513,8.929613554,1.5303603898
C,-0.0350247587,8.1718528823,-0.7804240401
C,0.0240555157,7.6025994907,1.9962533022
H,0.0270862496,9.744948801,2.2483107441
C,-0.0237244777,6.8719324564,-0.3179360131
H,-0.0578810904,8.3753356053,-1.8461255077
C,0.0056046921,6.5881027029,1.0601985609
H,0.0468108337,7.3708611659,3.0560338651
O,-0.0374345791,5.7296264552,-1.0303926077
O,0.0108044136,5.2595778167,1.2632034478
C,1.1854014377,2.4800503546,-0.1585074675
C,-1.2168800738,2.4758318455,-0.1372307576
C,-0.0136062693,1.776267095,-0.065367187
H,-0.0110363888,0.6998780213,0.0096977502
B,-0.0164272525,4.6114987937,-0.0715576746
O,-1.2263866581,3.7963696707,-0.2184867288
O,1.1863145285,3.7985897181,-0.2735674061
C,-4.755515297,1.4771862664,-0.1627840482
C,-3.9824326893,0.2311984501,-0.1616963633
C,3.9649078903,0.2544030081,-0.0840694465
C,4.7231072575,1.4897382545,-0.3053879614
N,2.6452467984,0.5403919031,-0.0039039491
C,-3.7947521256,2.5182444026,-0.153612649
C,3.7550676141,2.5223612772,-0.3635023714
C,-2.5068352404,1.8510460158,-0.1524300859
N,-2.6604004069,0.5178983376,-0.1694276888
C,2.4781716798,1.8613457377,-0.1727972104
H,-0.0245247715,10.2413613511,-0.1694375942
C,4.1307531154,3.8566599755,-0.5522107411
C,6.0817623852,1.8183559802,-0.396245923
C,6.4482943687,3.1446686878,-0.5820050316
H,7.5018477997,3.4013792267,-0.6536997644
C,5.480022559,4.1559079964,-0.6679463105
H,5.790671377,5.1868552073,-0.8130002956
H,6.8570411437,1.0679977008,-0.3053295083
H,3.376062475,4.631098053,-0.5959265643
C,-6.1166942098,1.8019966232,-0.2275096175
C,-4.1836247832,3.8619919951,-0.1667401052
C,-5.5374196881,4.1607328725,-0.202803076
H,-5.8579338734,5.1987700454,-0.2140608417
C,-6.4960945238,3.1375584145,-0.2415874436
H,-7.5517041762,3.3911288243,-0.2906645809
H,-3.4345116853,4.6429050862,-0.1591985183
H,-6.8828832215,1.039334882,-0.2873648729
C,4.4368648357,-1.0823235332,0.0675979419
C,3.5702486487,-2.0858785153,0.5867302472
C,5.754332803,-1.4787659215,-0.2832030905
C,3.9662941057,-3.3768385273,0.8025936515
H,2.5667900105,-1.7620183904,0.8269862959
C,6.2216456627,-2.7561550882,-0.1191584007
H,6.3944287457,-0.7398780863,-0.7392522277
C,5.3375932857,-3.7728647319,0.460017105
O,5.7445196866,-4.9484920058,0.64794962
C,3.0163852447,-4.4222191081,1.4022579232
C,3.5947003798,-4.9649657543,2.7247273778
C,1.6330042588,-3.8329442909,1.7085791328
C,2.8193735085,-5.5867283499,0.4110905611
H,4.5659138583,-5.4323262116,2.5602947878
H,3.7117745229,-4.1539616245,3.452122286
H,2.9094079613,-5.7062901422,3.1541351085
H,1.141438586,-3.4493978815,0.8105153262
H,0.9920284675,-4.6163847315,2.1261196735
H,1.6856679781,-3.0202561854,2.4395088007
H,2.1380483462,-6.3313826954,0.8404046306
H,2.3739992448,-5.2237684494,-0.5212485622
H,3.7718567273,-6.0663138819,0.1835750301
C,7.644520358,-3.1555113404,-0.5331460492
C,8.4317262438,-3.6537807116,0.6956937358
C,7.5937007722,-4.2706312223,-1.5965826265
C,8.4268361857,-1.9790819975,-1.1351996447
H,8.5097001074,-2.8608182789,1.4477505521
H,7.9346728792,-4.5138954948,1.1444904884
H,9.4486637676,-3.9414242456,0.399244694
H,7.0691528083,-3.9217336909,-2.4928179992
H,8.6117670933,-4.5577006021,-1.8894563452

H,7.0756427382,-5.1472742889,-1.2073654122
H,9.4297450988,-2.3204893798,-1.4141750401
H,7.9476068577,-1.5845177179,-2.0367976976
H,8.5442356455,-1.1568741535,-0.4216892223
C,-4.4400511841,-1.1190052944,-0.1757318461
C,-3.5302235069,-2.173302071,-0.4734398579
C,-5.7843986332,-1.4801821744,0.1040953247
C,-3.9053557171,-3.4862905034,-0.5462668021
H,-2.5087103784,-1.873133448,-0.6629768433
C,-6.2358716664,-2.773265738,0.0715081621
H,-6.4634658687,-0.6944273363,0.3953666636
C,-5.3028935067,-3.848486057,-0.2807798278
O,-5.691770603,-5.0433262315,-0.3443197178
C,-2.9043237507,-4.592069028,-0.9066567058
C,-3.3552205806,-5.3149705014,-2.191930194
C,-1.4972437589,-4.0337571621,-1.1575834228
C,-2.8040747836,-5.6098953827,0.2472363073
H,-4.3385163853,-5.7666013117,-2.0580750206
H,-3.4010951657,-4.6110708094,-3.0303323419
H,-2.633537617,-6.0997772815,-2.4502574247
H,-1.0936616511,-3.5262126248,-0.2772713339
H,-0.8199671658,-4.858633026,-1.4018962761
H,-1.4782192226,-3.3280154636,-1.9936024549
H,-2.0853979379,-6.3971843554,-0.0108923613
H,-2.4495694263,-5.119070924,1.1598618138
H,-3.7743005842,-6.0659157293,0.4464245949
C,-7.691650453,-3.1294402433,0.4020814109
C,-8.3611051014,-3.7976867922,-0.8158280969
C,-7.7419026953,-4.0904529183,1.6066162117
C,-8.5253489764,-1.8916225876,0.7642898484
H,-8.3674579471,-3.1141451333,-1.6720935721
H,-7.8252697033,-4.7046228993,-1.0962153714
H,-9.4016517867,-4.0544371191,-0.5789224402
H,-7.3031586016,-3.6179538138,2.4922667302
H,-8.7832750458,-4.3470720771,1.8397299733
H,-7.1907626842,-5.0055451537,1.3893046823
H,-9.5504366428,-2.2036929866,0.9923555597
H,-8.1321450747,-1.373839465,1.6449182057
H,-8.5742306723,-1.1744071217,-0.0616150287

1c^{2-}-1 (triplet state)

–2636.2937283 hartree
C,0.0441101,9.0130742105,0.8723653322
C,0.2045002971,8.6050282088,2.1908608925
C,-0.0762335155,8.0722591934,-0.165928234
C,0.2507246608,7.2399191467,2.5249836589
H,0.2966443006,9.3482061393,2.9779213963
C,-0.0295302156,6.7350978879,0.168225703
H,-0.2022847414,8.3765522347,-1.1997026856
C,0.1317219898,6.3217656645,1.5021895533
H,0.3766404432,6.9076534783,3.5501380108
O,-0.1213104519,5.6638501379,-0.6456395446
O,0.1459832842,4.9766361734,1.5743833389
C,1.1632265306,2.369704832,-0.1467618306
C,-1.2275184347,2.3644861865,0.2128085711
C,-0.0157155715,1.674096993,0.133206078
H,0.0016161473,0.6031229896,0.2043991951
B,-0.0166242364,4.4753457809,0.1970488389
O,-1.2474857573,3.6644072029,0.0923964807
O,1.1614868171,3.6732677834,-0.1975976157
C,-4.1933887223,0.2076735075,0.7327808594
C,-4.6836179387,1.4829903625,0.2236256796
C,4.6068232187,1.5191734708,-0.5223221996
C,4.0794074622,0.2069504499,-0.8770451266
N,3.5787226758,2.3692786833,-0.2823611802
C,-2.780105768,0.3497965904,0.8202695723
C,2.6640464008,0.3308439703,-0.796995318
C,-2.5063473451,1.700952516,0.3672135358
N,-3.6409802296,2.3323781399,0.0552873892
C,2.4252790283,1.7075255566,-0.4068106933
H,0.0109381181,10.0728592884,0.6358254279
C,1.8403955705,-0.7473005527,-1.1488763933
C,4.6424333533,-1.0046141431,-1.303440986
C,3.8143930818,-2.0695075915,-1.6241201112
H,4.2518815456,-3.0074182946,-1.9548903518
C,2.4191635929,-1.9412347489,-1.5484364597
H,1.7843199279,-2.7789585367,-1.8224794521
H,5.7128818138,-1.1244888922,-1.4138832333
H,0.7607894689,-0.6569381476,-1.1427121462
C,-4.7960340373,-0.9803813881,1.1703630532
C,-1.9991224803,-0.68888071,1.3453731985
C,-2.6139443654,-1.8621720026,1.7524605494
H,-2.011647721,-2.6690870871,2.1599457929
C,-4.006575219,-2.0075395146,1.6645174729
H,-4.4747383995,-2.9274903462,2.003546753
H,-0.9279821393,-0.5832670649,1.4678590979
H,-5.8710356496,-1.1090330339,1.1560906966
C,5.953197659,1.9775902474,-0.3987003469
C,6.20395854,3.3682201002,-0.2149900141
C,7.0783162547,1.1128512768,-0.4283117586
C,7.4613139383,3.8927283391,-0.1021757458
H,5.326827817,3.9995565189,-0.1760813885
C,8.3696422916,1.5588224859,-0.3156169789
H,6.8957399734,0.0534157773,-0.5125236519
C,8.6206675272,2.9951942274,-0.1601975487
O,9.795708009,3.4383013968,-0.0720974932
C,7.6909258698,5.3990609083,0.0810773317
C,8.5238205773,5.9529172086,-1.0923887723
C,6.3708840183,6.1810854558,0.1252996719
C,8.4335834509,5.6615599327,1.406680107
H,9.4924955495,5.4554876402,-1.1487742097
H,7.9946694131,5.8041818296,-2.0400219543
H,8.6828005033,7.0306179186,-0.9611955261
H,5.7308138458,5.8648690544,0.9538548057
H,6.5890986044,7.2458561958,0.2609429896
H,5.7974692508,6.0749023143,-0.8001507012
H,8.5923541975,6.7391753739,1.5383473867
H,7.8396302407,5.3034367217,2.2545146463
H,9.4001167215,5.1569063276,1.4160072688
C,9.5618619753,0.5926437845,-0.3386975742
C,10.4878407247,0.9254643168,-1.5259855472
C,10.3566432806,0.7000519746,0.9780933873
C,9.121818542,-0.8705821377,-0.4937020482
H,9.9485969283,0.8190005412,-2.473724131
H,10.8580393698,1.9476955235,-1.4470858528
H,11.3410954517,0.235303254,-1.5419048079
H,9.7231486353,0.4324984568,1.8308867158
H,11.2096807858,0.0097529857,0.9595563939
H,10.7249669572,1.7160247879,1.1211109313
H,10.0091747018,-1.5129302324,-0.5027005494

H,8.4834852006,-1.1979841013,0.3330927568
H,8.582076316,-1.0390994028,-1.4312927011
C,-6.0077113202,1.9081329919,-0.0984159726
C,-6.2462002433,3.2789338992,-0.405754776
C,-7.1194176765,1.0273339643,-0.1521122083
C,-7.483842945,3.7721069199,-0.7120845977
H,-5.3773503994,3.9220820234,-0.3752134354
C,-8.3899936081,1.4416153389,-0.4572553839
H,-6.9391925763,-0.0207906974,0.0263222636
C,-8.6324600857,2.859553255,-0.7416143816
O,-9.7904604534,3.2744662288,-1.0095080674
C,-7.7028708484,5.2589132096,-1.0232017849
C,-8.685259158,5.8723863159,-0.0053424769
C,-6.3959111684,6.0605449912,-0.9501786116
C,-8.2713633865,5.4212268859,-2.4473152682
H,-9.6481681129,5.3619688775,-0.0403116634
H,-8.2811125352,5.7950926701,1.0099100223
H,-8.8366353707,6.9362304302,-0.2272517999
H,-5.6516137242,5.7027807467,-1.6673849711
H,-6.6055777823,7.1101905085,-1.1829256782
H,-5.9451898929,6.0250720617,0.0458246119
H,-8.4230117256,6.484926996,-2.6696124733
H,-7.5697403976,5.0199758868,-3.186686445
H,-9.223433152,4.8989980644,-2.5462735025
C,-9.5665176611,0.4580094447,-0.5183542023
C,-10.6393724067,0.8538990541,0.5161085947
C,-10.1878383807,0.4615468133,-1.9294276245
C,-9.1365523977,-0.9832232852,-0.2074717076
H,-10.2242519289,0.8222384998,1.5295161339
H,-11.0063426274,1.8613847184,0.319768115
H,-11.4810145103,0.15081577,0.4722343505
H,-9.448533952,0.1474570107,-2.674376185
H,-11.030316032,-0.2406152827,-1.9708247923
H,-10.5436185001,1.4588524805,-2.1885118836
H,-10.0119415202,-1.6392198036,-0.2661595855
H,-8.3950978295,-1.3546123955,-0.9220154443
H,-8.7192874929,-1.0779861296,0.8003568036

1c^{2-}-2 (triplet state)

–2636.3042724 hartree
C,0.1944504181,9.1404847845,-0.1591517393
C,0.205941831,8.8398431899,1.1973818455
C,0.1579245004,8.118953696,-1.1252427836
C,0.1813342224,7.5064443349,1.6432489357
H,0.2372458589,9.643672024,1.9275411775
C,0.1342415316,6.8130797986,-0.6817590169
H,0.1519653889,8.3388635154,-2.187795726
C,0.1457611533,6.5083177259,0.6910515376
H,0.1930912487,7.2579523168,2.6993063165
O,0.0961748115,5.6804934944,-1.4118293136
O,0.1159052113,5.174958926,0.8729215895
C,1.1732374141,2.3813048507,-0.523487942
C,-1.2306994737,2.4994429598,-0.4477340877
C,-0.0599162507,1.7544483982,-0.3350433537
H,-0.1421224359,0.6996900494,-0.1404998754
B,0.074028721,4.5591435025,-0.4689705533
O,-1.1864597541,3.7987176536,-0.6344865446
O,1.2316202057,3.681411113,-0.6813252776
C,-4.7912126694,1.543255393,-0.3809338097
C,-4.0294298533,0.3010519973,-0.2524796187
C,4.6095144479,1.4373357588,-0.499878186
C,4.077783547,0.0953902033,-0.718295167
N,3.5879311931,2.3253971847,-0.4516006725
C,-3.8206866179,2.5753885628,-0.4720481803
C,2.6647612992,0.248182525,-0.7649931892
C,-2.5427613244,1.905623638,-0.4023616208
N,-2.7026152743,0.5822212171,-0.2836531067
C,2.4303499891,1.6694910034,-0.5794892523
H,0.2169451301,10.1774585342,-0.4823005531
C,1.8430221363,-0.8570555596,-1.0225583397
C,4.6423199213,-1.170122129,-0.9307895982
C,3.8144523115,-2.2581421986,-1.1636188155
H,4.2522061803,-3.2391093639,-1.3263625635
C,2.421379656,-2.1026374219,-1.2090550429
H,1.7870479805,-2.9623573613,-1.4041475051
H,5.7146978238,-1.319237222,-0.9399279532
H,0.7676824956,-0.7558687355,-1.1002510322
C,-6.1505528185,1.8798759324,-0.4621312113
C,-4.1954455772,3.9166240062,-0.6130285758
C,-5.5448650873,4.2246949958,-0.6723502503
H,-5.8560959199,5.2597931455,-0.7814328682
C,-6.5144965223,3.2112258176,-0.6033815438
H,-7.5678523029,3.4710330382,-0.665880488
H,-3.436980196,4.686487987,-0.6797542654
H,-6.9292870109,1.1281765817,-0.4324846896
C,5.9535265658,1.8911241541,-0.3403688082
C,6.2195584391,3.2908546451,-0.3146481659
C,7.0611362805,1.0174087188,-0.1848945795
C,7.4767669133,3.8111044886,-0.1814916806
H,5.3543888776,3.9317618974,-0.4154323834
C,8.3507057672,1.4596375132,-0.040127385
H,6.864295761,-0.0421595431,-0.1507646764
C,8.619456784,2.9008771718,-0.0472008131
O,9.7947310181,3.3388324447,0.0658773061
C,7.7234850822,5.3259200461,-0.171901903
C,8.6440921964,5.7185099489,-1.3448682067
C,6.4194407363,6.1225084079,-0.3166814581
C,8.3808483926,5.7445990543,1.1585836161
H,9.6037293831,5.2059133823,-1.2718621745
H,8.176541424,5.4582273334,-2.3008299342
H,8.8162625266,6.8022477736,-1.3380656698
H,5.7190307651,5.9200457693,0.4985809848
H,6.6494424689,7.1934070366,-0.2999977845
H,5.9073795665,5.9071560491,-1.2590320593
H,8.5530056881,6.8282743487,1.1653819509
H,7.7241112236,5.5032528299,2.0013861929
H,9.3336993483,5.2329315566,1.2973267412
C,9.5224647039,0.4844977495,0.1379819989
C,10.5316928232,0.6560445831,-1.0152306351
C,10.2301202136,0.746421007,1.482530826
C,9.0649633214,-0.9815607408,0.1355355024
H,10.0544874667,0.4380451162,-1.9770563794
H,10.9156889919,1.6760173629,-1.03826189
H,11.3704025075,-0.0402936295,-0.887255453
H,9.5363073056,0.5940117643,2.3165229783
H,11.069030728,0.049891251,1.6077279406
H,10.6074896735,1.7683324177,1.5235514717
H,9.9381313844,-1.6300324357,0.2666898685
H,8.3668720477,-1.1961742192,0.9508899828
H,8.5853560213,-1.2596513344,-0.8085965125

C,-4.4715619234,-1.048956143,-0.1164309498
C,-3.5214208289,-2.1090281814,-0.1726447659
C,-5.8293154129,-1.4086506674,0.0878910066
C,-3.8621059201,-3.4286785438,-0.0673865159
H,-2.4929073047,-1.8086185525,-0.3147880675
C,-6.252201855,-2.7067848321,0.2104736944
H,-6.5515140092,-0.6131963053,0.1754727652
C,-5.2705102513,-3.792154625,0.1248471293
O,-5.6276486966,-4.9955743775,0.219252191
C,-2.8084013047,-4.5415416203,-0.1485970254
C,-3.1150268319,-5.473483745,-1.338055897
C,-1.3930174234,-3.982070517,-0.3498307734
C,-2.8043043211,-5.3604199407,1.1578504501
H,-4.0995495921,-5.9288765887,-1.2281295161
H,-3.0883574461,-4.9135523591,-2.2793057204
H,-2.3590881998,-6.2666019816,-1.3960691333
H,-1.0857343917,-3.3306951087,0.4735519548
H,-0.6817944858,-4.8136374628,-0.3979711915
H,-1.304863722,-3.4149602412,-1.2816383599
H,-2.0491451484,-6.1541449782,1.0992514894
H,-2.5533216565,-4.7195661763,2.009997431
H,-3.7809612092,-5.8122373377,1.3334040428
C,-7.7287783618,-3.055338369,0.4423369611
C,-8.2533596678,-3.9273015965,-0.7162161882
C,-7.890372571,-3.8176240554,1.7727427721
C,-8.6170138385,-1.8046802043,0.5164698295
H,-8.1796094158,-3.386498345,-1.6661677041
H,-7.6765505306,-4.8491113682,-0.7926168013
H,-9.3088453305,-4.1778316567,-0.5497183306
H,-7.5557061817,-3.1983266441,2.6121619158
H,-8.9462123075,-4.0684573291,1.9362678932
H,-7.3047102957,-4.7369791106,1.7608583473
H,-9.6552853758,-2.1115986874,0.6833524079
H,-8.3297104684,-1.1444376577,1.3409932989
H,-8.5884513365,-1.2259389341,-0.412525598

$1c^{2-}$-3 (triplet state)
–2636.3094726 hartree
C,-0.0164930767,9.2129461451,0.121762629
C,0.0173006883,8.9522708015,1.4861483296
C,-0.03817313,8.1628260095,-0.8139323729
C,0.030682611,7.6319641076,1.9704164474
H,0.0337386185,9.7774992353,2.1925995641
C,-0.024756969,6.8696751841,-0.333211098
H,-0.0647920219,8.3515216099,-1.8822134328
C,0.0094125743,6.6052043991,1.0482248055
H,0.0571793736,7.414775828,3.0331493188
O,-0.0404117203,5.7167979765,-1.0294400774
O,0.0157797167,5.2786603458,1.2692256631
C,1.1839179643,2.4806373531,-0.1416206547
C,-1.2163333722,2.4774188458,-0.1106881486
C,-0.0140000134,1.7761541534,-0.0439202553
H,-0.0116280201,0.6996433107,0.0245012748
B,-0.0159720884,4.6146596444,-0.0544606511
O,-1.2261822573,3.7944713926,-0.1852369785
O,1.1858535654,3.7959369965,-0.2489241494
C,-4.7604374554,1.4781353275,-0.1303993976
C,-3.9891432599,0.2370627869,-0.147127943
C,3.9702529609,0.2582520781,-0.0783958548
C,4.7256259249,1.4875698727,-0.3092111858
N,2.6490267244,0.5466658578,0.008180029
C,-3.7992930596,2.5218095443,-0.1144709596
C,3.7571873576,2.5230077136,-0.3655142015
C,-2.514525215,1.8569844748,-0.1269189428
N,-2.6650873042,0.525664205,-0.1571733443
C,2.4843921062,1.865346634,-0.1652459688
H,-0.0263915826,10.2403054847,-0.2314358067
C,4.1363393746,3.8566069591,-0.5638326686
C,6.0854467984,1.8151866716,-0.4126046384
C,6.4524559738,3.1386914182,-0.6069891583
H,7.5058115315,3.3937191504,-0.6878128579
C,5.4840239413,4.1524234692,-0.6906184206
H,5.7957706437,5.1820381769,-0.8430134186
H,6.8603089884,1.0637367588,-0.3248014386
H,3.3838299264,4.6334248886,-0.6064124815
C,-6.123772884,1.8037159093,-0.1829613646
C,-4.1925950789,3.8659789578,-0.1093569214
C,-5.5457376662,4.1627275792,-0.1342454278
H,-5.8680857167,5.200311117,-0.1314665592
C,-6.5045507559,3.137531362,-0.1794334567
H,-7.560688955,3.3907186659,-0.2194434563
H,-3.4456852394,4.6491194918,-0.0964726799
H,-6.8896096747,1.0407138874,-0.2464634464
C,4.435317512,-1.0825105581,0.0758035828
C,3.5655450153,-2.0789951155,0.6029398332
C,5.7488048415,-1.4869370073,-0.2799196494
C,3.9554137886,-3.3715427781,0.8213425778
H,2.5650676732,-1.7484219373,0.8461119755
C,6.2103921467,-2.7662552357,-0.1121477799
H,6.3903301954,-0.7522006689,-0.7406473079
C,5.3231231787,-3.7757439614,0.4744663829
O,5.7247038377,-4.9530109695,0.665298265
C,3.0020988892,-4.409715284,1.427921169
C,3.5822139891,-4.9509739491,2.7502249393
C,1.622938828,-3.8119341299,1.7370174755
C,2.7954727816,-5.576418689,0.4412818966
H,4.5498382767,-5.4251027718,2.5841184164
H,3.7070640528,-4.1380380528,3.474134276
H,2.8938987367,-5.6863354023,3.1849357764
H,1.1302352761,-3.4288801419,0.8393779972
H,0.979437939,-4.5905849111,2.1595714326
H,1.6824564726,-2.9970097243,2.4648729356
H,2.110776257,-6.3152994552,0.8750835286
H,2.3497315734,-5.2140951224,-0.491106181
H,3.7442715936,-6.0627859097,0.2128200658
C,7.6296821399,-3.1742640771,-0.5296831616
C,8.4188606675,-3.6712518643,0.6984273444
C,7.569398828,-4.2934296911,-1.5883957812
C,8.4153094265,-2.0040028131,-1.1393962751
H,8.5023797088,-2.8760010658,1.4474315103
H,7.9203961789,-4.5280247735,1.1519791883
H,9.4336828471,-3.963582823,0.3994892776
H,7.0421258472,-3.9460795859,-2.4836086993
H,8.5850229242,-4.5853391775,-1.8847846416
H,7.0499524961,-5.1667045302,-1.193565762
H,9.4155654337,-2.3513910969,-1.4204387568
H,7.9347434329,-1.6109864803,-2.0409017104
H,8.538980676,-1.1793526066,-0.4298716586
C,-4.439367895,-1.1173658114,-0.1732119053
C,-3.5245989582,-2.1633036879,-0.484460915

C,-5.7808388554,-1.4880859848,0.1068623606
C,-3.8927373076,-3.4777060168,-0.5689652355
H,-2.505397149,-1.8552993845,-0.6733979809
C,-6.2257820347,-2.7833769731,0.0613230919
H,-6.4627969415,-0.7081216925,0.406804249
C,-5.2876901158,-3.8496302225,-0.3039888405
O,-5.6704462945,-5.0460650362,-0.378806941
C,-2.8863518203,-4.5743975292,-0.9417076232
C,-3.3357334579,-5.287516229,-2.2329760964
C,-1.4828161374,-4.0058845134,-1.1898634017
C,-2.7784091422,-5.6024260826,0.2024259715
H,-4.3157857104,-5.7468110946,-2.1015570471
H,-3.3879339704,-4.5758657297,-3.0644077963
H,-2.6096210686,-6.0650980887,-2.5004338883
H,-1.0803956366,-3.504420117,-0.3055571803
H,-0.8015912768,-4.8247591469,-1.4431755412
H,-1.469143191,-3.2922033408,-2.0191737521
H,-2.055000968,-6.3825151087,-0.0640413837
H,-2.4259037127,-5.1181225604,1.1192793033
H,-3.7453392718,-6.0667058899,0.398476896
C,-7.6790781319,-3.1503290698,0.390651738
C,-8.3472007812,-3.8090440226,-0.8331823698
C,-7.7222788004,-4.1241412828,1.5851711913
C,-8.5181434141,-1.920468649,0.7672913634
H,-8.3573839322,-3.1170328552,-1.6825472729
H,-7.8084559228,-4.7111270977,-1.1234083875
H,-9.3864056692,-4.0720773283,-0.5974842746
H,-7.2830886132,-3.6592858408,2.4746153991
H,-8.7620918268,-4.3869121434,1.8181965967
H,-7.168449846,-5.0349589828,1.3571318752
H,-9.5412472217,-2.2400787478,0.9937338681
H,-8.1259536294,-1.4101515372,1.6526627038
H,-8.5718496044,-1.1947566229,-0.0507684996

TBA^{+}-1c^{2-} (open-shell singlet state)

–3305.5763208 hartree

C,8.0814605951,-2.4735169641,0.5829117337
C,8.9989086969,-3.1464875295,-0.4778109262
C,7.9303757259,-3.4205177755,1.8071244066
C,8.7701787696,-1.1743288794,1.0599291679
H,9.9802224634,-3.3631778172,-0.0361566769
H,7.3095838525,-2.9460008847,2.5764033437
H,9.7496901758,-1.4291379713,1.4815440703
H,6.7288783817,-0.1166910983,0.2687472458
H,8.5287993524,-4.0723831753,-0.8098870209
H,9.1386021979,-2.4765436064,-1.3344373023
H,8.9181175011,-3.6355312264,2.2349876032
H,8.9283726682,-0.4778329603,0.2274086684
H,8.1834794723,-0.6723083169,1.838590658
C,4.1174830986,-1.816621904,-1.1354974082
C,4.5925206546,-3.0982381267,-1.0958184788
C,3.781716625,-4.2852089176,-1.6419610592
C,2.4230493928,-3.8422404217,-2.2293649393
C,4.5902818651,-4.988724096,-2.7691804042
C,3.5127825043,-5.2973083695,-0.4924697939
C,5.9262680574,-3.3676201261,-0.5280899017
C,6.6982436625,-2.2113273241,-0.0363532882
C,6.1739047276,-0.9504590812,-0.1308039704
C,4.8804983729,-0.6943103911,-0.6781953691
C,4.3256342288,0.6014313013,-0.7846268138
C,4.9880612053,1.9260264043,-0.6881581884
C,6.304465298,2.3594367191,-0.5088892742
C,6.5729354624,3.7323449237,-0.5020948164
C,5.548617221,4.6705266108,-0.6886788415
C,4.2312974707,4.2559721255,-0.9019569255
C,3.9583176042,2.8877433692,-0.902719869
C,2.7316873543,2.1194713345,-1.1190926063
C,1.4446392776,2.6583930776,-1.3531326776
C,0.307175096,1.8631259527,-1.4805747423
C,-0.9397897572,2.4391730884,-1.7323952852
C,-2.1433460039,1.7085532872,-1.7419196691
C,-3.44478487,2.2175453322,-2.1628613784
C,-3.8476585216,3.4350095193,-2.7172857954
C,-5.1833014365,3.5836623172,-3.0939474433
C,-6.0929319735,2.5275590079,-2.936607449
C,-5.6945915657,1.3084022101,-2.3807428711
C,-4.3681728914,1.1502339185,-1.9638249925
C,-3.5784794754,0.0410926947,-1.372055647
C,-4.0239398725,-1.2315375802,-0.9248881506
C,-5.4019817201,-1.5560258513,-0.7532508421
C,-5.8284054811,-2.7913609761,-0.3362918091
C,-7.3226745107,-3.1275917779,-0.183454834
C,-8.2373477322,-1.9306691146,-0.5302627613
C,-4.8462358708,-3.8435153953,-0.0361161778
C,-3.423634652,-3.5037400274,-0.2078753181
C,-3.0648180575,-2.2473506492,-0.6213718608
C,-2.3830328951,-4.6016731012,0.0699290978
C,-0.9389827487,-4.1210137948,-0.1873873107
C,-2.6541702651,-5.8204371558,-0.8581826554
H,3.1452832292,-1.5888356096,-1.5486207954
H,2.5578333166,-3.1436286555,-3.0631120435
H,5.5468330858,-5.3220585853,-2.3658870755
H,2.9171011017,-4.820577437,0.2953808145
H,7.1218724854,1.6606785527,-0.3973851178
H,7.5926641813,4.0733202753,-0.3600275583
H,5.7835477493,5.7290848478,-0.6844887566
H,3.4293855925,4.9561885522,-1.0803864044
H,0.3957116571,0.7909896314,-1.4003059505
H,-3.1205177348,4.2224928256,-2.8483928323
H,-5.5181035893,4.5195108056,-3.5274815366
H,-7.1206031441,2.6530286475,-3.2598647977
H,-6.4114072195,0.5023567135,-2.3109943591
H,-6.1312229908,-0.7816512041,-0.927526603
H,-2.0277550102,-1.9799449034,-0.7704751426
H,7.4600524723,-4.3471196516,1.4771084027
H,-0.7993644182,-3.8184716269,-1.2312047496
H,-0.6738911586,-3.276410996,0.458578713
H,-0.24483174,-4.9419011429,0.0263286337
H,-2.5485362216,-5.5221124181,-1.9077195436
H,-1.9288610231,-6.6159987848,-0.6449872284
H,-3.6667057214,-6.1824478703,-0.6790998649
H,-8.0539298859,-1.0790997119,0.1363328342
H,-8.0949891581,-1.6072184855,-1.5686055986
H,1.8948665824,-4.726822398,-2.6038041643
H,1.7922203785,-3.3674032537,-1.469724438
H,4.0245321908,-5.8503108042,-3.145984721
H,4.7621559168,-4.2925983082,-3.5986278164
H,2.9541684964,-6.1590999217,-0.8792757012
H,4.4671259251,-5.6282533703,-0.081416937
N,2.9850613553,0.7832448402,-1.046548798

N,-2.2586604939,0.4190127093,-1.2825218896
O,6.3955639802,-4.5551847552,-0.4589191415
O,1.3456112614,4.0223870891,-1.4211303437
O,-1.0325610355,3.7962185406,-1.9457272187
O,-5.2155250128,-5.0058795468,0.3601883058
C,-7.621908424,-3.5603422897,1.2794227538
C,-2.4971748339,-5.0519249324,1.5538102591
H,-6.98046097,-4.4061223645,1.5285469327
H,-1.7764619285,-5.8537722425,1.7585458798
H,-2.2784406522,-4.2077297185,2.2203476691
H,-3.5121637017,-5.408917033,1.7329492264
H,-7.4234600925,-2.7302462476,1.9676471248
C,-7.6749041445,-4.300664795,-1.1428872728
H,-9.2827219549,-2.2389130976,-0.4099631285
H,-7.0440665511,-5.1546448436,-0.8940622649
H,-8.7326053748,-4.5712429745,-1.0278279383
H,-7.4981211493,-4.0026762631,-2.1829995408
H,-8.6767018906,-3.849537857,1.3739386073
C,-0.297825532,0.8521795672,1.6283109697
C,0.6102459887,-0.0961517216,2.4199721745
C,1.3405163683,-1.0582387088,1.4485521544
C,-0.213735657,2.722941606,3.3519539035
C,0.8817375121,3.4749228939,2.5813674639
C,1.6310120026,4.4363177174,3.5398443826
C,-1.9865028375,2.6868011764,1.5276815205
C,-3.0551214399,3.5737789565,2.1817920456
C,-3.6805130057,4.4770606986,1.0852249473
C,-2.0417895798,1.0426694206,3.4682602838
C,-2.9948768069,0.0332653926,2.8141978399
H,-1.0066284904,0.3148278091,0.9924783215
H,0.0341813717,-0.6953460497,3.1354873014
H,1.8516653691,-0.4841781935,0.6664566514
H,0.2295051591,2.0663486514,4.1049057324
H,0.4651589443,4.0608861234,1.7562583454
H,2.1359005887,3.8599559933,4.3269884611
H,0.9053396137,5.099697519,4.0309324
H,-2.4379371491,1.9850429338,0.8267716693
H,-1.2884322967,3.3208236131,0.981211843
H,-3.8465855933,2.9741693404,2.6468423723
H,-2.6130906691,4.2194409036,2.9510305085
H,-4.1396016476,3.8463412937,0.3156254483
H,-2.8759964505,5.0396372466,0.6015891091
H,-3.62791067,0.5170671934,2.0639205907
H,-2.4347253057,-0.7578740972,2.3066726383
H,0.292762083,1.4887316687,0.9704893856
H,1.3634606969,0.4728079921,2.9787130797
H,0.5947746562,-1.694004602,0.9568391396
H,1.6078187895,2.7705852628,2.1615510276
N,-1.1371036996,1.8225971718,2.4985823511
C,-3.8934988577,-0.6079648306,3.9015152489
H,-2.6040620016,1.7964245427,4.0238333462
H,-1.3718536803,0.5291865556,4.1607185585
H,-4.5047222992,0.1736075225,4.3731421794
H,-3.2597736681,-1.0453531225,4.6853054645
H,-0.8785632172,3.430371463,3.8532841644
C,-4.8043924362,-1.7003636592,3.3054376077
H,-5.4545467648,-2.1283161338,4.0761452725
H,-5.432191175,-1.2920928205,2.5072752834
H,-4.2072276222,-2.5071966617,2.8689424616
C,2.3566196256,-1.9462100588,2.1963159294
H,2.8653435972,-2.6121962447,1.4934778394
H,3.1205533375,-1.3292763731,2.6842440705
H,1.858779046,-2.555318044,2.9613346322
C,2.6592996056,5.2909297512,2.7662770617
H,3.2201287861,5.9377347462,3.4502419035
H,3.3696580446,4.6543875486,2.2276141355
H,2.1459895131,5.9206093757,2.0319576467
C,-4.7311368845,5.4400630612,1.6732972616
H,-4.2739049839,6.1064187841,2.4147249247
H,-5.1659496714,6.059827847,0.8822036719
H,-5.5429105397,4.8849475675,2.1594663101
B,0.0192422731,4.6978630347,-1.4169402224
O,0.0659679426,5.9893211074,-2.1373648019
O,-0.3413844008,5.0991673521,0.0300800813
C,-0.162923519,7.0051607251,-1.2309687784
C,-0.40122376,6.4878679901,0.0572936602
C,-0.1766890625,8.3687400846,-1.460881316
C,-0.6520791334,7.3220813749,1.130740882
C,-0.4324184711,9.2249742065,-0.3732974237
H,0.0072591807,8.7555210297,-2.454519504
C,-0.666121692,8.7120791708,0.9023910442
H,-0.8344478009,6.9142542776,2.117374021
H,-0.4460586812,10.297100858,-0.5337400817
H,-0.8603796262,9.3855197916,1.729408069

TATA$^+$-1c^{2-} (open-shell singlet state)

–3885.0165765 hartree

C,-1.8677293879,0.9340511392,-1.5411749542
C,-3.2432094731,1.3863924432,-1.5930236398
C,-3.8439912191,2.650725043,-1.654779861
C,-5.2229420201,2.725272504,-1.7741798916
C,-6.0026321342,1.5590812345,-1.8467117112
C,-5.4158481116,0.3034415251,-1.783055328
C,-4.0248955679,0.2046707097,-1.6350884506
C,-3.0672225581,-0.9000955378,-1.5957632523
C,-3.3099136676,-2.3060328249,-1.6320072187
C,-2.2572603667,-3.1974924705,-1.985062201
C,-2.4243616379,-4.5522146747,-2.0689209611
C,-1.3005172699,-5.4788069946,-2.5496975684
C,-0.044639625,-4.6933402159,-2.9494064501
C,-1.7660182791,-6.2676276372,-3.7905106759
C,-0.906662083,-6.4655108716,-1.4329661592
C,-3.7271044758,-5.134154319,-1.7142857084
C,-4.8194954771,-4.2200705634,-1.3565545423
C,-6.1953739528,-4.8033241792,-1.009587223
C,-6.7443157402,-5.6056958379,-2.2066214823
C,-7.2223495687,-3.7094854047,-0.6816550847
C,-6.088220655,-5.726854962,0.2202831133
C,-4.5778847942,-2.8724903241,-1.3352318425
H,-3.2330877863,3.5437126998,-1.629822286
H,-5.703690142,3.6973122736,-1.8406184789
H,-7.0785866023,1.6397443867,-1.9748410817
H,-6.0400549375,-0.5760443873,-1.8832330949
H,-1.3096085972,-2.7362333878,-2.2286996028
H,-0.2432995961,-3.9943257119,-3.7674220359
H,-2.0333155886,-5.5826541117,-4.6022978582
H,-0.111939367,-7.1326796791,-1.786494019
H,-6.8538020982,-4.9594882067,-3.0841111859
H,-8.1836769466,-4.1781860676,-0.4478723803
H,-5.3966440827,-6.5468092706,0.0272988606

H,-5.3665991557,-2.2008701137,-1.0329440702
H,0.7252796455,-5.3932272733,-3.2887098263
H,0.3748519584,-4.1267354327,-2.1137825906
H,-2.6299848136,-6.8899851413,-3.5581993169
H,-0.9525438492,-6.9099849675,-4.1472570675
H,-1.7626737619,-7.0678300079,-1.1275731552
H,-0.5222965296,-5.9258876058,-0.5604656935
H,-7.7321881841,-6.0139678161,-1.9618730258
H,-6.0755084948,-6.4279386108,-2.4598359162
H,-7.3821084544,-3.0314166012,-1.5261399694
H,-6.9265492216,-3.1110309764,0.1862792371
H,-7.07293068,-6.1425061918,0.4651719661
H,-5.733750469,-5.1673682549,1.0938075123
N,-1.8075434165,-0.4098944455,-1.5530098725
O,-3.9030033059,-6.3760127566,-1.7129960296
C,6.6795018979,-0.791904173,0.187540995
C,7.2915580119,-1.9481785785,0.5889800171
C,8.6953238179,-1.9496189542,1.2068120595
C,9.6491179843,-2.7989443512,0.342472133
C,9.2890568015,-0.5362290735,1.2962153154
C,8.6458848387,-2.5241902409,2.6367482671
C,6.5770538452,-3.2220832088,0.4337056345
C,5.3751109097,-0.7553967475,-0.3761956042
C,4.7330708342,0.462749673,-0.7508035734
C,5.2877809753,1.8158412241,-0.8549184207
C,6.5736633893,2.3697218272,-0.7950739228
C,6.7322267687,3.7365843744,-0.9741729742
C,5.6282104482,4.5647760275,-1.2244939751
C,4.3506311448,4.0346042588,-1.3158842188
C,4.1830693661,2.655893415,-1.1374862614
H,7.1872444898,0.1470897623,0.3375475971
H,9.299959426,-3.8292820134,0.2784279421
H,9.3937842099,-0.0719952974,0.3101847973
H,7.9969248776,-1.9139941406,3.2745730203
H,7.4530756875,1.7574377561,-0.6406893858
H,7.7281776434,4.1676964895,-0.9315579651
H,5.7782834638,5.6310078854,-1.3665096474
H,3.4970372705,4.6628528633,-1.5349355713
H,10.65462811,-2.7915234301,0.7797835163
H,9.7187893682,-2.3881294591,-0.6705224392
H,10.2880846325,-0.5951262806,1.7398442335
H,8.6867399632,0.125722826,1.926688097
H,9.6506305776,-2.5192938265,3.0757779821
O,7.1037734054,-4.3014992332,0.7902463664
C,5.2235493353,-3.1933527081,-0.1419364064
C,4.4638085479,-4.5138925353,-0.3134762883
C,5.2465140618,-5.4607542876,-1.2454529724
C,3.077558381,-4.3000488464,-0.9326570494
C,4.2644988822,-5.1909083408,1.057575674
C,4.6825868885,-1.9939832894,-0.5109688656
C,3.0372083543,1.7697133113,-1.2161062792
C,1.6749204533,2.1546667259,-1.3954039422
C,0.6065704389,1.2615851242,-1.5075218994
C,-0.6944512947,1.7560053665,-1.5069514902
H,5.3757762236,-5.0056341694,-2.2331295018
H,2.4473819205,-3.6504861448,-0.3163458701
H,3.6910794755,-1.9349741341,-0.9392016513
H,0.7862905774,0.1981100988,-1.5441327322
H,4.6909327863,-6.3966789597,-1.3765809486
H,6.2292343321,-5.6889016223,-0.8332334036
H,2.5694529913,-5.2643795535,-1.0263256593
H,3.1343278497,-3.8585054613,-1.9317395867
H,3.6863106486,-4.5428481066,1.7267374598
N,3.4061777353,0.4930494412,-1.0054213221
O,1.454810886,3.4623351145,-1.3846469207
O,-0.9207527467,3.0616187557,-1.4317995836
H,8.2695508141,-3.5470092479,2.6306205634
H,5.2231505475,-5.408109932,1.5283561268
H,3.7097695579,-6.1284533455,0.9336075543
C,1.4674630792,-1.1345602289,2.0220247754
C,0.113162663,-0.804202288,2.0140311414
C,-2.2166391674,-1.4598715559,2.0656250473
C,-4.9408139682,-0.7504913261,2.2409229077
C,-3.9826075349,0.2612245479,2.1477745137
C,-2.6088882679,-0.0951687965,2.1013025232
C,-6.4885772345,2.1644545325,3.2445684333
C,-7.9496285899,2.5375980235,3.0074008143
H,1.8054941059,-2.1591910385,1.9948429298
H,-5.9895772439,2.9474494611,3.825522121
H,-8.474524986,1.7546305127,2.4502175653
H,-6.4264737559,1.2507852501,3.8455082603
H,-8.4783411508,2.6841992824,3.9529726947
H,0.5600548459,-3.4869766739,3.5442923285
N,-0.8770999593,-1.7718516972,1.9482897119
C,-3.2013704871,-2.4497733021,2.1509075086
C,-4.5299353091,-2.0759083,2.2445713776
C,-0.4933111428,-3.1665190147,1.6930363799
C,-0.2329082503,-3.9718746154,2.9648655537
C,0.156457749,-5.4133513141,2.6469910217
H,-2.9520908971,-3.4996824639,2.1444033752
H,-5.2823244801,-2.8545902742,2.3153534579
H,-5.9943577293,-0.527730343,2.3171758376
H,-1.2783261206,-3.6223172316,1.0888369989
H,-1.1291463597,-3.9533679951,3.5946917462
H,1.0691426235,-5.4552435836,2.0446669169
H,0.3886361362,-3.1550588864,1.0525461665
H,-0.6329621408,-5.9253817825,2.087854939
H,0.3385147686,-5.9801609576,3.5639108542
C,-3.385037701,2.6134828546,2.1407872788
C,-3.7498635514,3.9571450415,2.2011363219
C,-2.7563362527,4.9277948019,2.2075480313
C,-1.4099336115,4.6238347905,2.1690339525
C,-1.0039477282,3.2856469947,2.1234557185
C,-2.0036057392,2.2736407582,2.1225186565
C,0.7276057367,1.5910669946,2.0907286654
C,2.073309633,1.2211610393,2.0983098278
C,2.412692748,-0.1211878276,2.0570686649
C,-0.2621468013,0.5678497124,2.0714107704
C,-1.6238184746,0.9156086472,2.0813278081
C,1.376969761,3.9534420565,1.9315437347
C,2.07739774,4.3433365896,3.2349246068
C,1.2157375533,5.0823179067,4.2568684521
C,-5.7424160932,1.9564263211,1.9278814805
H,-4.7836909761,4.2641417142,2.2446037391
H,-3.0500743977,5.9720898026,2.239945025
H,-0.6949008109,5.4284995058,2.1574142329
H,2.8655040991,1.9527089888,2.100028622
H,3.462614516,-0.3911222957,2.033086347
H,0.9333186276,4.8088757828,1.4277913374
H,2.9301583018,4.9717814559,2.953235453

H,0.8553640424,6.0381505354,3.8642855845
H,-6.2119986414,1.1760674197,1.3288308263
H,2.0999477899,3.5693056594,1.2127211653
H,2.4993332444,3.4486965631,3.7036460019
H,1.795170463,5.2947518683,5.1601885975
H,0.3436943323,4.4926832631,4.5538998777
H,-5.7856918162,2.8501647559,1.3050226336
H,-8.0345236439,3.4647999074,2.4310294905
N,0.3308024701,2.9210769641,2.0839325585
N,-4.3317550369,1.6000338963,2.0986281505
B,0.1388246855,4.0528921768,-1.6588145428
O,-0.0662738107,5.2182313783,-0.7751618188
O,0.0713105771,4.5930594841,-3.0276439261
C,-0.2545320189,6.2938497431,-1.575170619
C,-0.1715505882,5.9143117032,-2.9246295032
C,-0.4941819009,7.6019272172,-1.2101865204
C,-0.3291059981,6.8404232869,-3.9356478
C,-0.6556622376,8.5500226879,-2.2356543545
H,-0.5526104943,7.8846069268,-0.1637850677
C,-0.5748083863,8.1749246981,-3.5709653789
H,-0.262726468,6.5364495389,-4.9747694261
H,-0.844575997,9.5874136888,-1.9766249719
H,-0.7020286058,8.9224393094,-4.3481745303

[S14] M. J. Frisch, G. W. Trucks, H. B. Schlegel, G. E. Scuseria, M. A. Robb, J. R. Cheeseman, G. Scalmani, V. Barone, G. A. Petersson, H. Nakatsuji, X. Li, M. Caricato, A. V. Marenich, J. Bloino, B. G. Janesko, R. Gomperts, B. Mennucci, H. P. Hratchian, J. V. Ortiz, A. F. Izmaylov, J. L. Sonnenberg, D. Williams-Young, F. Ding, F. Lipparini, F. Egidi, J. Goings, B. Peng, A. Petrone, T. Henderson, D. Ranasinghe, V. G. Zakrzewski, J. Gao, N. Rega, G. Zheng, W. Liang, M. Hada, M. Ehara, K. Toyota, R. Fukuda, J. Hasegawa, M. Ishida, T. Nakajima, Y. Honda, O. Kitao, H. Nakai, T. Vreven, K. Throssell, J. A. Montgomery, Jr., J. E. Peralta, F. Ogliaro, M. J. Bearpark, J. J. Heyd, E. N. Brothers, K. N. Kudin, V. N. Staroverov, T. A. Keith, R. Kobayashi, J. Normand, K. Raghavachari, A. P. Rendell, J. C. Burant, S. S. Iyengar, J. Tomasi, M. Cossi, J. M. Millam, M. Klene, C. Adamo, R. Cammi, J. W. Ochterski, R. L. Martin, K. Morokuma, O. Farkas, J. B. Foresman, D. J. Fox, *Gaussian 16*, Revision C.01, Gaussian, Inc., Wallingford CT, 2016.

[S15] H. Maeda, Y. Haketa, T. Nakanishi, *J. Am. Chem. Soc.* **2007**, *129*, 13661–13674.

4. Solution-state properties

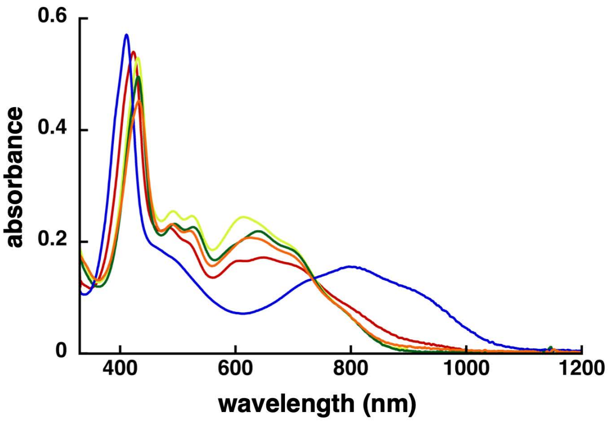

Figure S51 UV/vis absorption spectra of **1c** (1.0×10^{-5} M) in DMF (dielectric constant ε_r: 36.71; blue), acetone (ε_r: 20.56; red), CH_2Cl_2 (ε_r: 8.93; light green), $CHCl_3$ (ε_r: 4.81; dark green), and toluene (ε_r: 2.38; orange). In DMF, **1c** may form a deprotonated species.

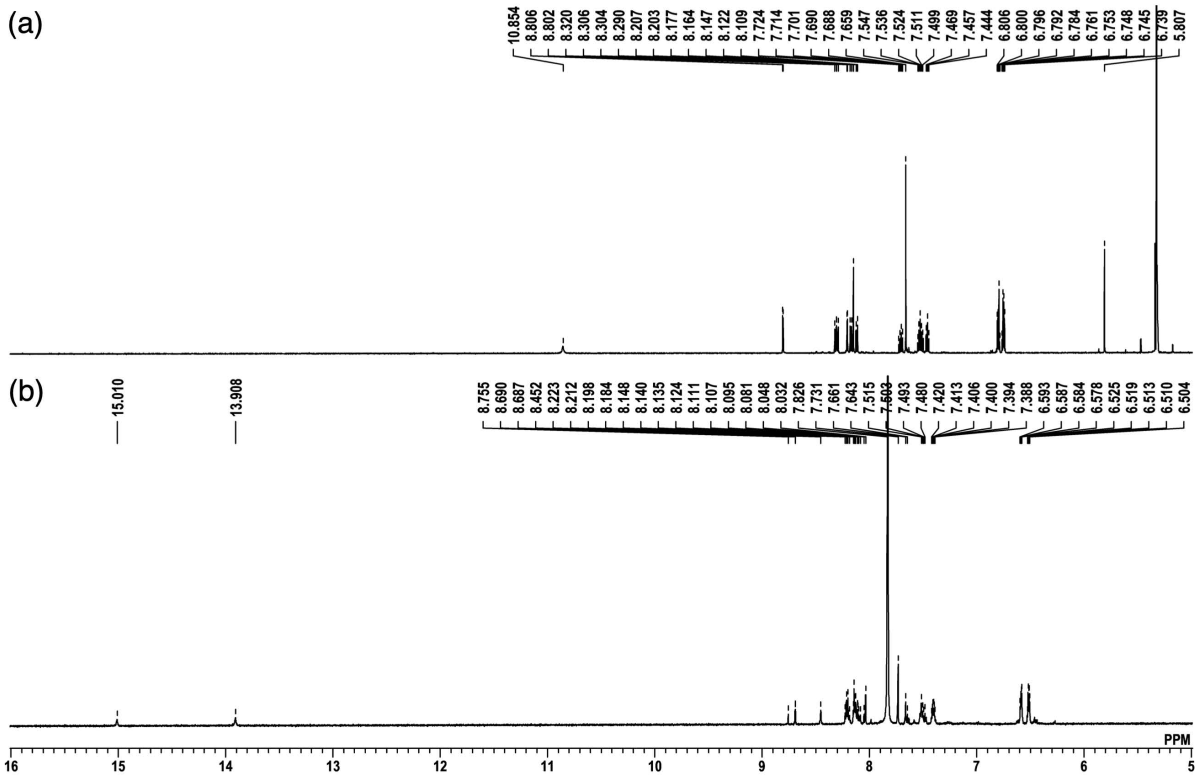

Figure S52 ^{1}H NMR spectra of **1c** in (a) CD_2Cl_2 and (b) DFM-d_7 (1×10^{-3} M). In DFM-d_7, two broad signals were observed in the downfield region (δ = 15.01 and 13.91 ppm), which were not observed in CD_2Cl_2.

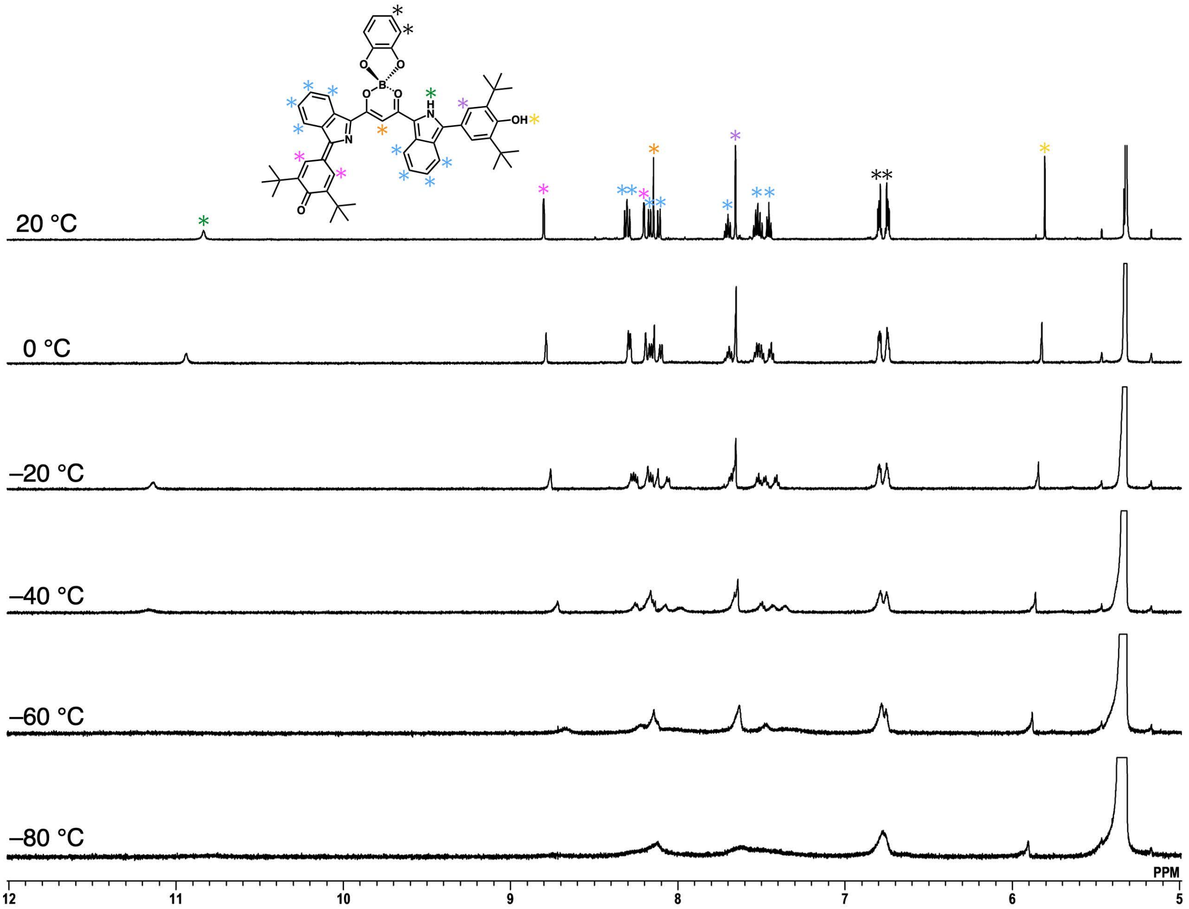

Figure S53 VT ^{1}H NMR spectra of **1c** in CD_2Cl_2 (1×10^{-3} M). As the temperature was decreased, significant broadening and disappearance of certain signals were observed, indicating the formation of aggregates.

5. Deprotonation behaviors and diradical properties

ESR measurements. ESR spectra of TBA^{+}_{2}-**1c**$^{2-}$ and $TATA^{+}$-TBA^{+}-**1c**$^{2-}$ were measured on a JEOL JES-RE1X. All samples were measured using a quartz capillary tube with degassed solvents and N_2 atmosphere. TBA^{+}_{2}-**1c**$^{2-}$ was prepared by the addition of TBAOH (2.0 equiv) to **1c**, whereas $TATA^{+}$-TBA^{+}-**1c**$^{2-}$ was prepared by the addition of TBAOH (1.0 equiv) to $TATA^{+}$-**1c**$^{-}$ (the captions in Figure S59,60 for the detail).

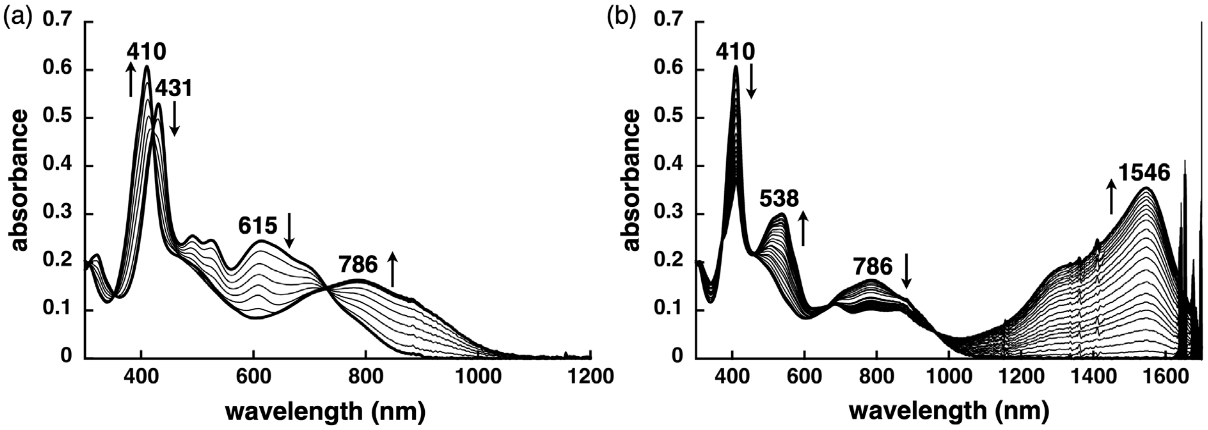

Figure S54 UV/vis absorption spectral changes of **1c** (1.0×10^{-5} M) upon the addition of OH^{-} as a TBA salt (10% in MeOH) ((a) 0–1.6 equiv and (b) 1.6–12.8 equiv) in CH_2Cl_2.

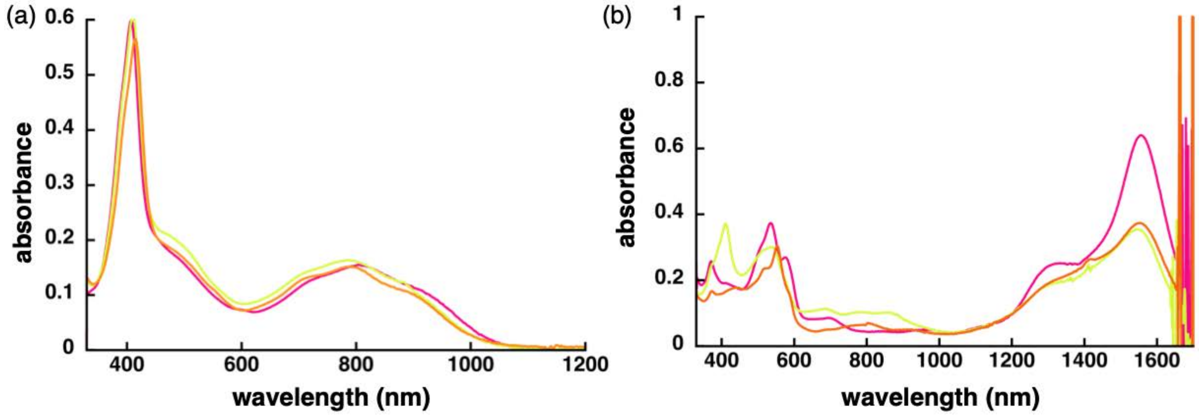

Figure S55 UV/vis absorption spectra of (a) **1c**$^{-}$ and (b) **1c**$^{2-}$ (1.0×10^{-5} M) as TBA^{+} ion pairs, prepared by the addition of TBAOH (10% in MeOH) to **1c** ((a) 5 equiv and (b) 35 equiv), in acetone (magenta), CH_2Cl_2 (green), and toluene (orange). The electronic property of **1c**$^{-}$ did not significantly depend on the solvent polarity, whereas that of **1c**$^{2-}$ was influenced by solvent polarity.

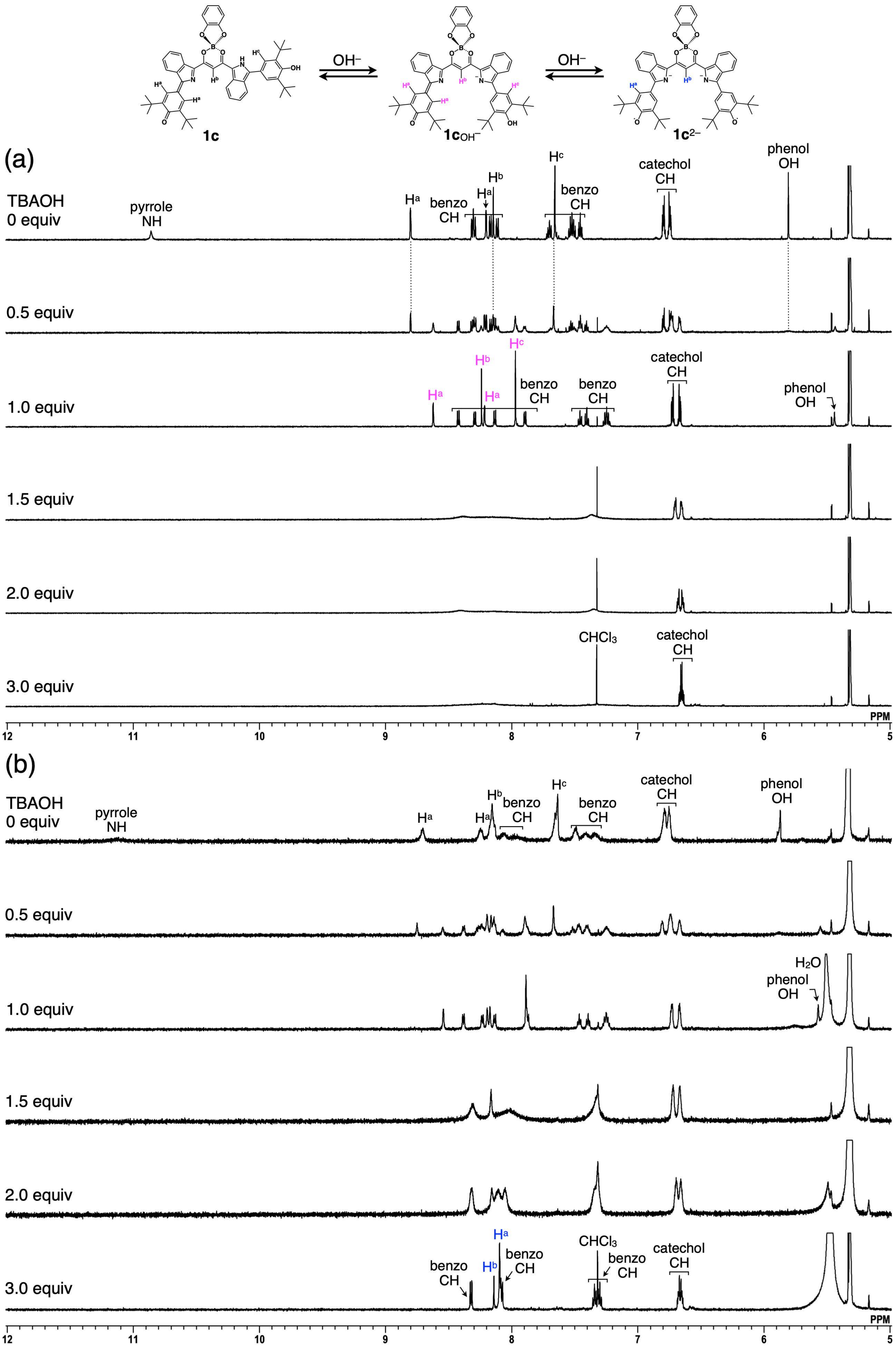

Figure S56 ^{1}H NMR spectral changes of **1c** (1×10^{-3} M) upon the addition of OH^- (0–3.0 equiv) as a TBA salt (10% in MeOH) in CD_2Cl_2 at (a) 20 °C and (b) –50 °C. The downfield shift of the NH signal at 0.5 equiv of TBAOH in (b) is ascribed to hydrogen bonding with MeOH. A signal attributable to $CHCl_3$ could not be excluded after careful repeated examinations.

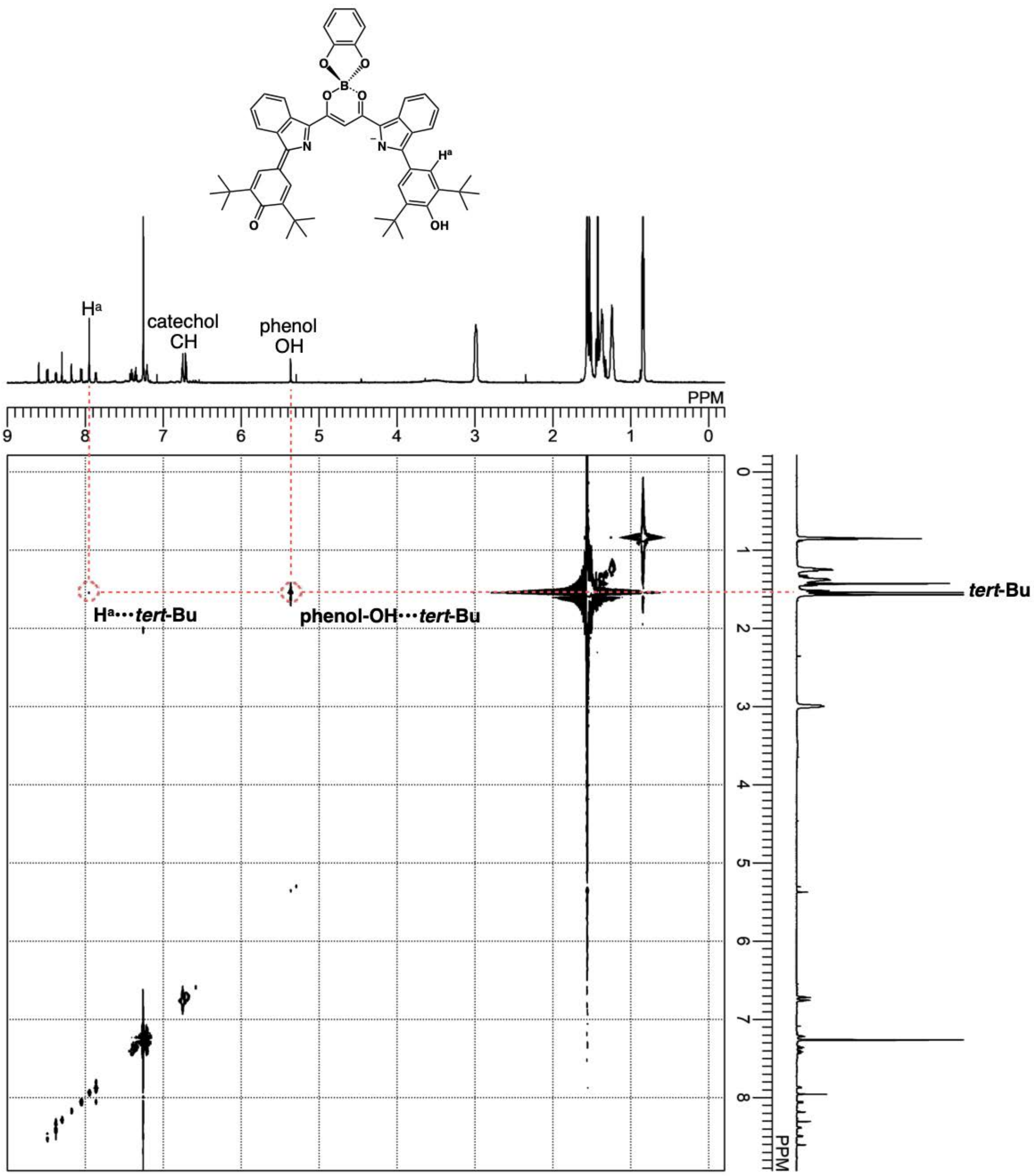

Figure S57 NOESY NMR spectrum of TBA$^+$-**1c**$_{OH}^-$ in $CDCl_3$ at 20 °C. **1c**$^-$ formed a **1c**$_{OH}^-$ tautomer with a **1c**$_{OH}^-$-**4** conformation (Figure S26a) due to the correlation between *tert*-butyl CH and phenol-OH.

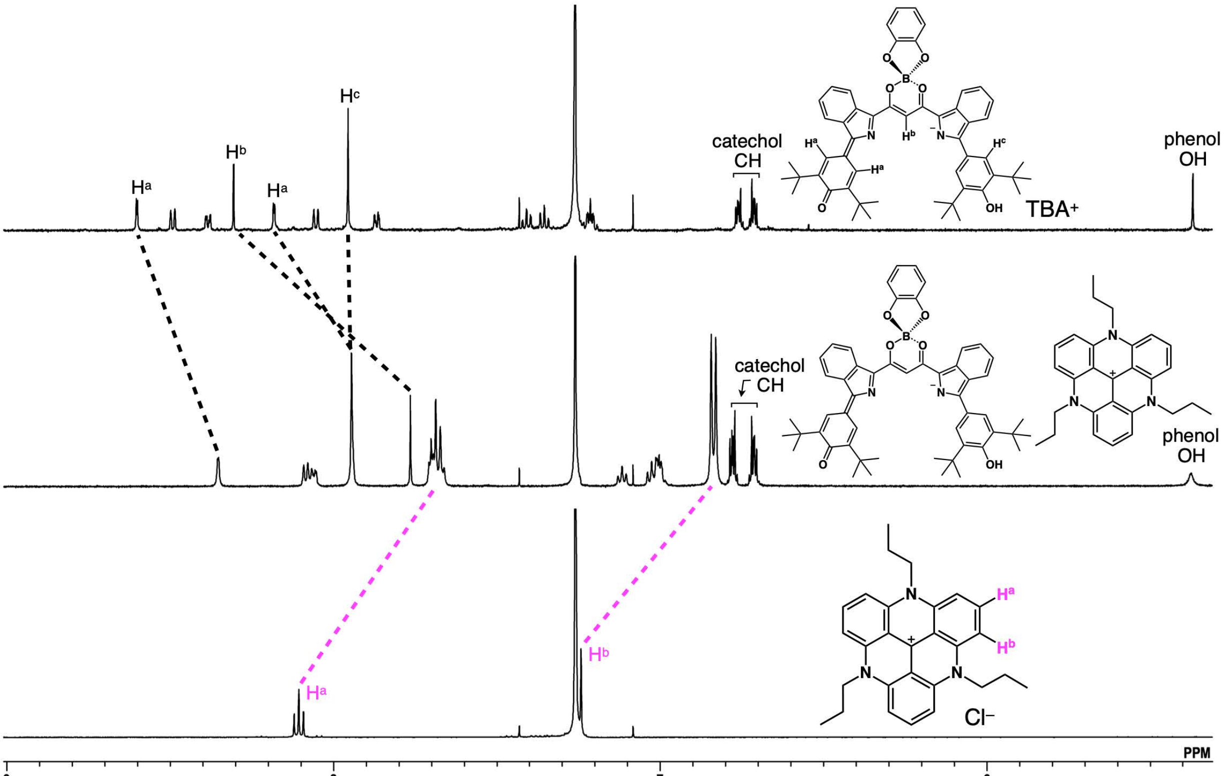

Figure S58 ^{1}H NMR spectra of TBA$^+$-**1c**$_{OH}$$^-$ (top), TATA$^+$-**1c**$_{OH}$$^-$ (middle) and TATA$^+$-Cl$^-$ (bottom) in $CDCl_3$ at 20 °C (1.0×10^{-3} M). TATA$^+$-**1c**$_{OH}$$^-$ was prepared by ion-pair metathesis between Na$^+$-**1c**$_{OH}$$^-$, which was prepared by treating of **1c** with NaOH (1.2 equiv), and TATACl (1.0 equiv). The signals of TATA$^+$-**1c**$_{OH}$$^-$ were shifted upfield from those of TBA$^+$-**1c**$_{OH}$$^-$ due to the shielding effect by stacking in solution. The monoanion was measured in $CDCl_3$ due to its sufficient stability in this solvent, whereas the dianion was less stable in $CDCl_3$, requiring measurement in CD_2Cl_2 (Figure S56).

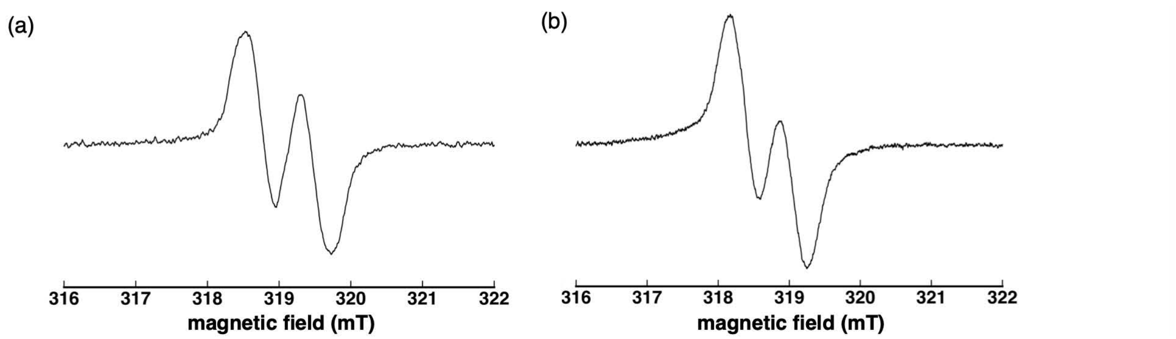

Figure S59 ESR spectra of (a) TBA$^{+}_{2}$-**1c**$^{2-}$ and (b) TATA^{+}-TBA^{+}-**1c**$^{2-}$ in toluene at 250 K and 300 K, respectively. TBA$^{+}_{2}$-**1c**$^{2-}$ was prepared by the addition of TBAOH (2.0 equiv, 10% in MeOH) to **1c** in toluene and TATA^{+}-TBA^{+}-**1c**$^{2-}$ was prepared by the addition of TBAOH (1.0 equiv, 10% in MeOH) to TATA^{+}-**1c**$^{-}$ in toluene. The *g* value of TATA^{+}-TBA^{+}-**1c**$^{2-}$ was estimated to be 2.004, suggesting that their spin density was delocalized at the core unit. The ESR spectra of TBA$^{+}_{2}$-**1c**$^{2-}$ and TATA^{+}-TBA^{+}-**1c**$^{2-}$ show slight deviations from the simulated triplet diradical profiles, including line broadening and asymmetry. These features suggest the presence of minor monoradical impurities. In TBA$^{+}_{2}$-**1c**$^{2-}$, such impurities were observed at 300 K, thus, representing the spectrum at 250 K. Even trace amounts of monoradicals can affect the ESR line shape due to their distinct spin relaxation behavior compared to triplet species. Although detailed spectral simulations would provide further insights, reliable reproduction of the observed spectra has not been feasible due to the existence of triplet diradical and monoradical species.

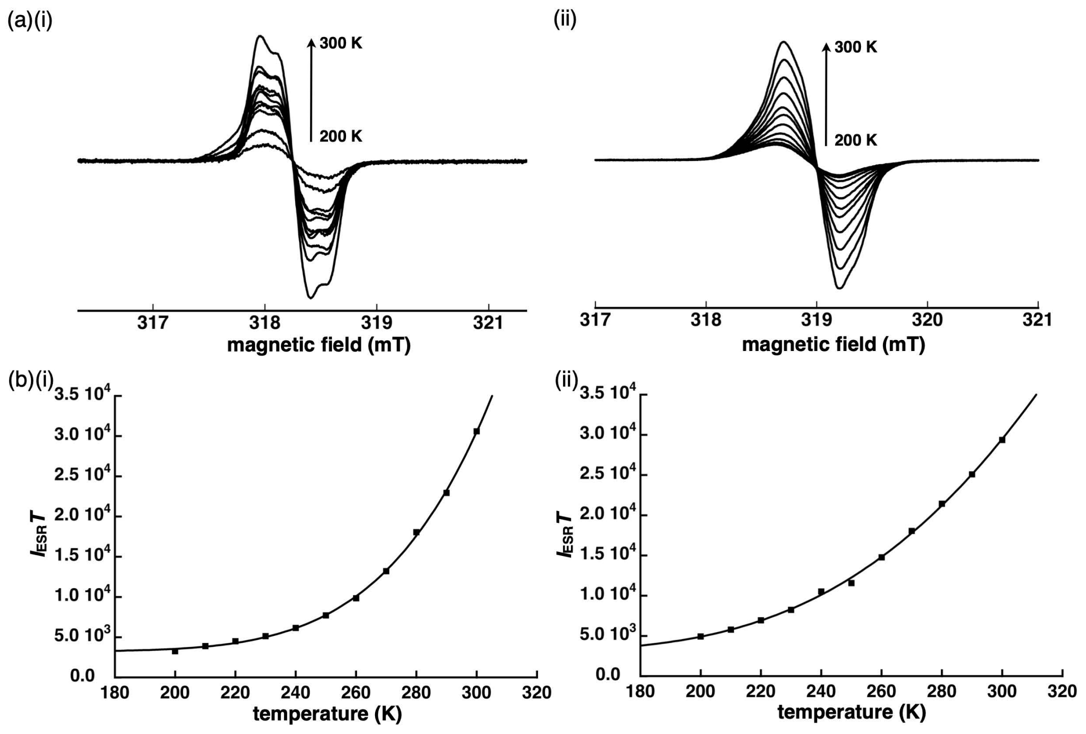

Figure S60 (a) VT ESR spectra and (b) ESR signal intensity of (i) TBA$^{+}_{2}$-**1c**$^{2-}$ and (ii) TATA^{+}-TBA^{+}-**1c**$^{2-}$ in toluene. TBA$^{+}_{2}$-**1c**$^{2-}$ was prepared by the addition of TBAOH (2.0 equiv, 10% in MeOH) to **1c** in toluene, whereas TATA^{+}-TBA^{+}-**1c**$^{2-}$ was prepared by the addition of TBAOH (1.0 equiv, 10% in MeOH) to TATA^{+}-**1c**$^{-}$ in toluene. Enhancement of ESR signal intensity at high temperatures suggested that the singlet-state diradicals were more stable than the corresponding triplet-state diradicals. The plots were fitted using the Bleaney-Bowers equation for the two-site Heisenberg Hamiltonian,[S16] which was described as

$$I_{ESR}T = \frac{C_1}{3 + \exp\left(-\frac{2J}{RT}\right)} + C_2$$

(*J*: exchange interaction constant, $S_{1,2}$: operators for the spin of two unpaired electron, C_1: constant, C_2: constant of the radical impurity, *R*: gas constant). The fitting curves provided the singlet–triplet energy gap (ΔE_{ST}) of –5.4 and –3.2 kcal/mol for TBA$^{+}_{2}$-**1c**$^{2-}$ and TATA^{+}-TBA^{+}-**1c**$^{2-}$, respectively.

[S16] a) B. Bleaney, K. D. Bowers, *Proc. R. Soc. London, Ser. A* **1952**, *214*, 451–465; b) S. Wu, Y. Ni, Y. Han, X. Hou, C. Wang, W. Hu, J. Wu, *Angew. Chem. Int. Ed.* **2022**, *61*, e202115571.
